# Engineered HaloTag variants for fluorescence lifetime multiplexing

**DOI:** 10.1038/s41592-021-01341-x

**Published:** 2021-12-16

**Authors:** Michelle S. Frei, Miroslaw Tarnawski, M. Julia Roberti, Birgit Koch, Julien Hiblot, Kai Johnsson

**Affiliations:** 1 Department of Chemical Biology, Max Planck Institute for Medical Research, Heidelberg, Germany; 2 Institute of Chemical Sciences and Engineering (ISIC), École Polytechnique Fédérale de Lausanne (EPFL), Lausanne, Switzerland; 3 Protein Expression and Characterization Facility, Max Planck Institute for Medical Research, Heidelberg, Germany; 4 Leica Microsystems CMS GmbH, Mannheim, Germany

**Keywords:** Chemical tools, Proteins, Confocal microscopy, Fluorescent dyes, Fluorescence imaging

## Abstract

Self-labeling protein tags such as HaloTag are powerful tools that can label fusion proteins with synthetic fluorophores for use in fluorescence microscopy. Here we introduce HaloTag variants with either increased or decreased brightness and fluorescence lifetime compared with HaloTag7 when labeled with rhodamines. Combining these HaloTag variants enabled live-cell fluorescence lifetime multiplexing of three cellular targets in one spectral channel using a single fluorophore and the generation of a fluorescence lifetime-based biosensor. Additionally, the brightest HaloTag variant showed up to 40% higher brightness in live-cell imaging applications.

## Abstract (web summary)

HaloTag variants offer distinct brightness and fluorescence lifetimes compared with HaloTag7 when labeled with rhodamines. These variants were used for multiplexed imaging with a single fluorophore and to create lifetime-based cell cycle indicators.

## Main

**Fig. 1:**
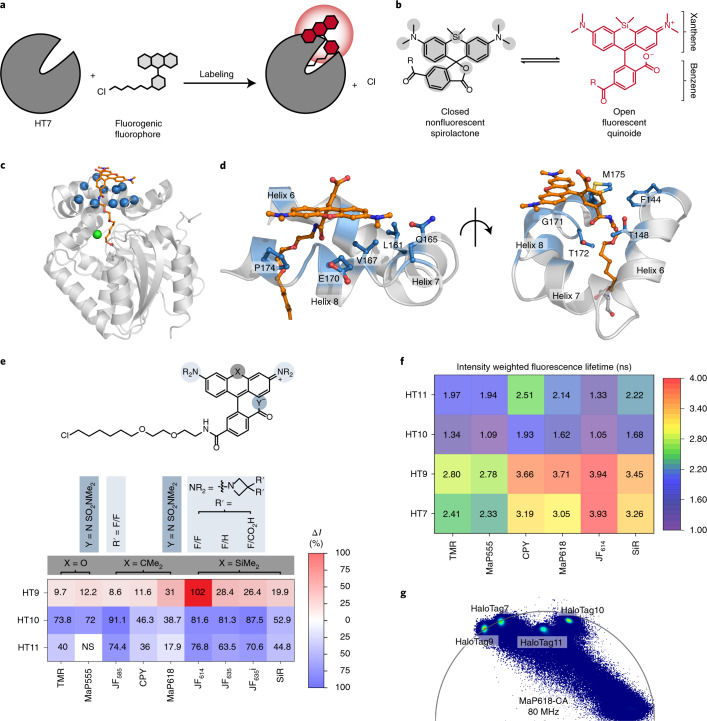
Characterization of HaloTag variants. **a**, Scheme of HaloTag7 labeling with a fluorogenic fluorophore. **b**, Environmentally sensitive open–closed equilibrium of SiR, a fluorogenic rhodamine. The equilibrium position can be tuned through environmental changes or through chemical modifications indicated by the gray areas. R, chloroalkane (CA). **c**, Crystal structure of HaloTag7-TMR (PDB ID: 6Y7A, 1.4 Å). The protein is represented as a gray cartoon and TMR as orange sticks. The Cα of the ten amino acids chosen for site-saturation mutagenesis are highlighted as blue spheres. The chlorine atom is shown as a green sphere. **d**, TMR binding site (helices 6–8) on the HaloTag7-TMR crystal structure. Same structural representation as in **c**, but with the ten amino acid side chains represented by blue sticks. **e**, Relative in vitro fluorescence intensity (Δ*I*) changes of labeled HaloTag variants compared with those of HaloTag7. Unless otherwise stated, R = Me and Y^− ^= O^−^ in the generalized chemical structure (Supplementary Table 4; Δ*I*: mean, for *N* see Supplementary Table 5). **f**, Fluorescence lifetimes (*τ*) of different rhodamines on the four HaloTags (mean, for *N* see Supplementary Table 8). **g**, Overlaid phasor plots of the four HaloTags expressed in the cytosol of U-2 OS cells labeled with MaP618-CA.

Multiplexed fluorescence microscopy based on fluorescence lifetime is an attractive approach to image multiple targets simultaneously. It requires only one spectral channel and therefore frees up other channels for further multiplexing^1–5^. To facilitate the use of fluorescence lifetime multiplexing in living cells, we aimed to establish a method in which different variants of a self-labeling protein (SLP) tag are labeled with a single fluorophore but become distinguishable based on their fluorescence lifetime. To achieve this, we focused on the combination of HaloTag7 with fluorogenic rhodamine-based fluorophores, which increase fluorescence upon target binding (Fig. 1a)^6–9^. The fluorogenicity of rhodamines is based on an environmentally sensitive equilibrium between a closed, nonfluorescent spirocyclic and an open, fluorescent quinoid form (Fig. 1b)^8^. While numerous strategies have been introduced to control the properties of rhodamines via chemical modifications^8,10–12^, little attention has been paid to the influence of the protein^13–16^. However, SLP engineering offers an alternative approach to control not only the fluorogenicity but also the photophysical properties of fluorogenic rhodamines. Here, we describe the engineering of HaloTag7 to modulate the brightness and fluorescence lifetime of bound rhodamines for use in live-cell fluorescence lifetime multiplexing.

## Results

### HaloTag engineering and characterization

**Extended Data Fig. 1:**
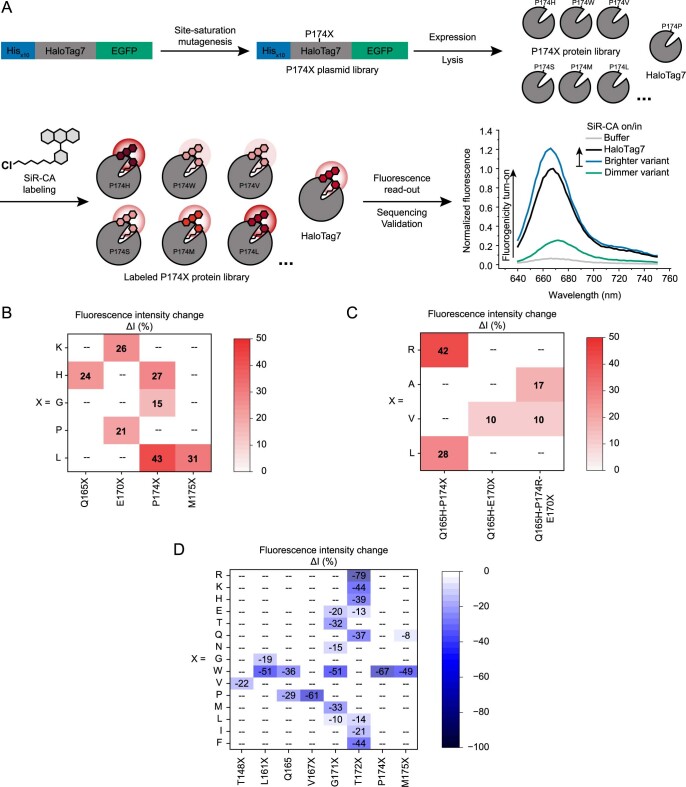
HaloTag7 engineering strategy and results. **a** Schematic representation of the *in vitro* engineering of HaloTag7. Site-saturation mutagenesis was performed onto the plasmid containing His_x10_-HaloTag7-EGFP using degenerate primers and Gibson cloning. The plasmid library (for example P174X) was transformed in *E. coli* for protein production and extraction. The cell lysate (protein concentration estimated via EGFP signal: 50-150 nm) was labeled with a limiting amount of SiR-CA (5 nm) and screened for increases in fluorescence intensity compared to HaloTag7. Brighter or dimmer hits (increased/decreased fluorescence intensity of SiR on variants in comparison to SiR on HaloTag7) were sequenced and validated in a separate fluorescence assay using purified protein. For visibility EGFP is not shown in the expressed and labeled protein libraries. **b** and **d** Outcome of the first round of screening. Brighter variants in (**b**) and dimmer variants in (**d**). Validated hits are given with their fluorescence intensity change (Δ*I*_var-EGFP_ = (*I*_var_ – *I*_Halo_) · *I*_Halo_^−1^, mean, *N* = 3 samples). **c** Outcome of the second and third round of screening. Validated hits are given with their fluorescence intensity change (Δ*I*_var-EGFP,_ mean, *N* = 3 samples, Supplementary Table 1-2).

The rhodamine binding site of HaloTag7 is formed by three helices (6–8), as revealed by the crystal structure of tetramethyl-rhodamine (TMR) bound to HaloTag7 (Protein Data Bank (PDB) identification number (ID): 6Y7A, 1.4 Å)^17^. To engineer the binding site, ten amino acids on helices 6–8 in close proximity to TMR were chosen for randomization by site-saturation mutagenesis (Fig. 1c,d and Supplementary Fig. 1). The resulting libraries were expressed in *Escherichia coli* (cytoplasmic) and the cleared lysates screened for changes in brightness upon labeling with fluorogenic silicon rhodamine (SiR-CA). This led to the identification of both brighter (Δ*I* = (*I*_var_ – *I*_HT7_) × *I*_HT7_^−1^ > 0) and dimmer (Δ*I* < 0) HaloTag variants with similar labeling kinetics and thermostability as HaloTag7 (Extended Data Fig. 1, Supplementary Fig. 3 and Supplementary Tables 1–3). The brightest variant, the double mutant HaloTag9 (HaloTag7-Q165H-P174R, Δ*I* = 19.9 ± 0.5%), as well as the dimmer variants HaloTag10 (HaloTag7-Q165W, Δ*I* = −52.9 ± 1.2%) and HaloTag11 (HaloTag7-M175W, Δ*I* = −44.8 ± 0.8%) were chosen for characterization with a panel of 46 different fluorophores and analysis of their photophysical properties (Supplementary Tables 4–11). HaloTag9 increased the fluorescence intensity of 16 fluorophores, including rhodamines based on the popular SiR, carbopyronine (CPY) and TMR scaffolds. The most pronounced changes for each scaffold were found for the fluorogenic rhodamines: JF_614_-CA^15^ (Δ*I* = 102 ± 11%), MaP618-CA^11^ (Δ*I* = 31 ± 4%), and MaP555-CA^11^ (Δ*I* = 12.2 ± 1.7%). The changes in intensity were found to stem from changes in quantum yield and/or extinction coefficient. HaloTag10 and HaloTag11 decreased the fluorescence intensity of almost all tested fluorophores compared with HaloTag7. The decreases were more pronounced for HaloTag10 than for HaloTag11 but in both cases caused by changes in quantum yield (Fig. 1e, Supplementary Figs. 3–4 and Supplementary Tables 4–11). The changes in brightness were stable over the physiological pH range (Supplementary Fig. 10).

Solving the crystal structures of HaloTag9-TMR (PDB ID: 6ZVY, 1.4 Å), HaloTag10-TMR (PDB ID: 7PCX, 1.4 Å) and HaloTag11-TMR (PDB ID: 7PCW, 2.3 Å) allowed us to rationalize how the protein surface influences the spectroscopic properties of the fluorophore. HaloTag9 showed altered electrostatic surface potential, as well as changes in dihedral angles and rotational energy barriers of the bound TMR compared with HaloTag7, explaining changes in extinction coefficient and quantum yield. For HaloTag10 and HaloTag11, the proximity of TMR to the introduced tryptophans suggests that photoinduced electron transfer (PET) quenching of the fluorophore is responsible for the decreased quantum yields (Supplementary Figs. 5–9 and Supplementary Tables 12–14)^18^.

### Changes in fluorescence lifetimes

**Fig. 2:**
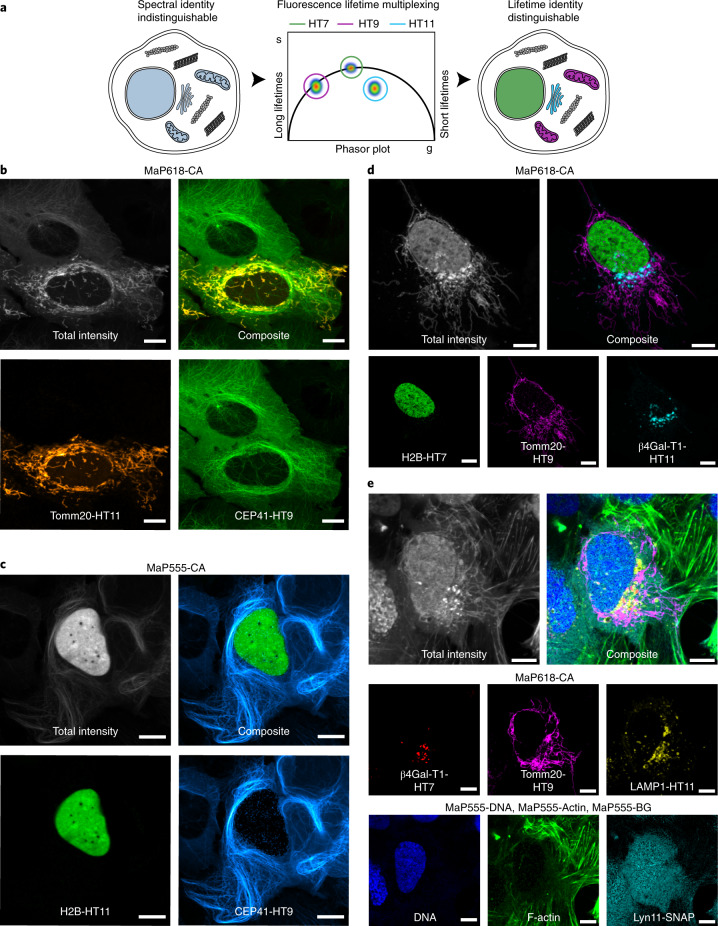
Application of HaloTag variants in fluorescence lifetime multiplexing. **a**, Schematic view of fluorescence lifetime multiplexing using only one rhodamine for three targets (nucleus, mitochondria and Golgi apparatus). **b**–**d**, Fluorescence lifetime multiplexing of U-2 OS cells expressing CEP41 as a HaloTag9 fusion and the outer mitochondrial membrane protein Tomm20 as a HaloTag11 fusion (**b**); CEP41 as a HaloTag9 and histone H2B as a HaloTag11 fusion (**c**); or H2B as a HaloTag7 fusion, Tomm20 as a HaloTag9 fusion, and the membrane-bound glycoprotein of the Golgi apparatus beta-1,4-galactosyltransferase (β4Gal-T1) as a HaloTag11 fusion (**d**). The different HaloTag variants were labeled with MaP618-CA (**b**,**d**) or MaP555-CA (**c**; 1 μM, 3 h). Representative composite, total intensity and individual images with the separated structures are given. Scale bars, 10 μm. **e**, Six-species image acquired by combining fluorescence lifetime multiplexing in both the MaP618 and MaP555 channel. U-2 OS cells expressing β4Gal-T1-HaloTag7, Tomm20-HaloTag9 and the lysosome-associated membrane glycoprotein 1 (LAMP1) as a HaloTag11 fusion, and the tyrosine protein kinase Lyn11 as a SNAP-tag fusion were labeled with MaP618-CA (1 μM, 2 h), MaP555-BG (2 μM, 2 h), MaP555-DNA (0.5 μM, 30 min) and MaP555-Actin (0.5 μM, 30 min). The composite, the total intensity and the six individual images with the separated structures are given. Representative images of two experiments are shown. Scale bars, 10 μm.

**Extended Data Fig. 2:**
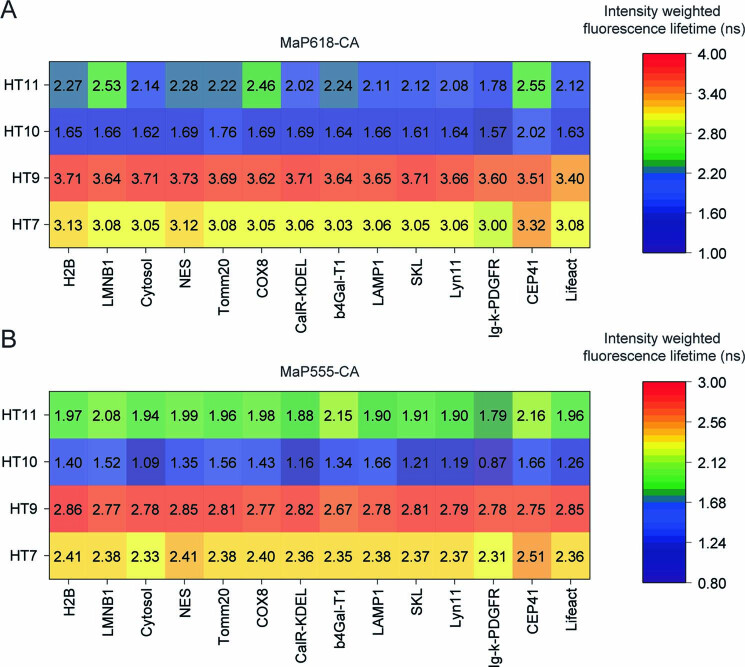
Summary of intensity weighted fluorescence lifetimes of HaloTag variants on different subcellular targets. **a** HaloTag variants or HaloTag7 labeled with MaP618-CA. **b** HaloTag variants or HaloTag7 labeled with MaP555-CA. Overall fluorescent lifetimes were relatively stable over different subcellular targets and most stable for HaloTag7 and HaloTag9 fusions. HaloTag10 and HaloTag11 fusions showed more fluctuations. CEP41 of HaloTag7, HaloTag10, and HaloTag11 show larger changes in fluorescence lifetime for MaP618-CA and MaP555-CA compared to other fusion proteins.

The measured changes in fluorescence lifetime of six fluorophores on the four HaloTags correlated with changes in quantum yield. Hence, HaloTag9 showed the longest and HaloTag10 the shortest fluorescence lifetime. Labeling with MaP555-CA or MaP618-CA resulted in evenly spaced distributions of fluorescence lifetimes, ranging from 1.1 to 2.8 ns and from 1.6 to 3.7 ns, respectively (Fig. 1f,g and Supplementary Table 8). Either of the two MaP fluorophores is therefore particularly well suited for fluorescence lifetime multiplexing of up to three different HaloTag variants in one spectral channel (Fig. 2a)^1–3^. The fluorescence lifetimes of MaP555-CA and MaP618-CA bound to the different HaloTag variants was relatively insensitive to their subcellular localization or fusion partner (Extended Data Fig. 2, Supplementary Figs. 11–12 and Supplementary Table 15).

### Changes in brightness in cellulo

**Extended Data Fig. 3:**
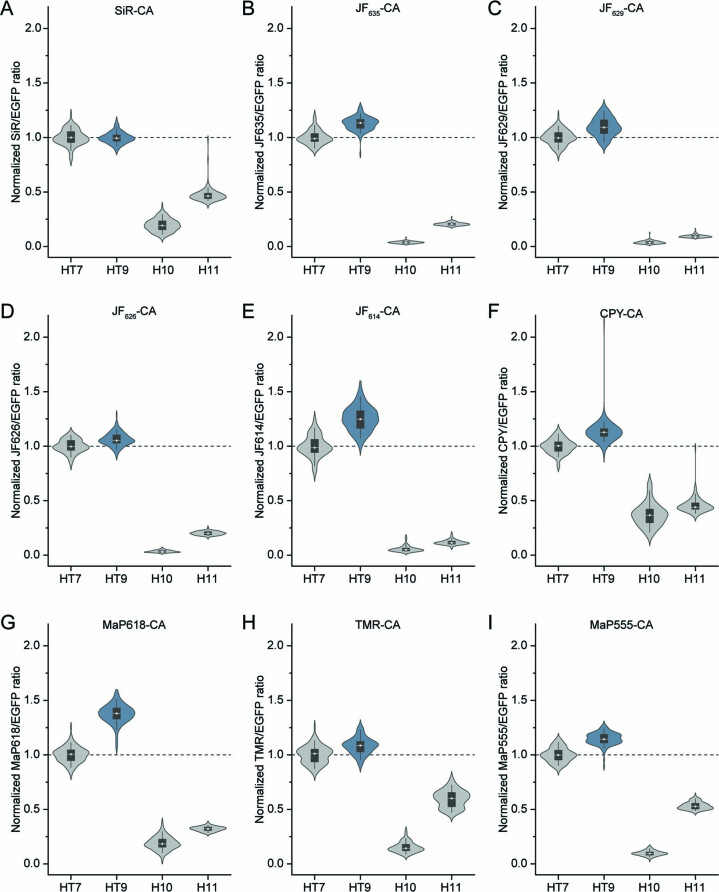
Brightness comparison of HaloTag variants in mammalian cells by confocal microscopy. **a**-**i** Violin plots of normalized fluorescence intensities from living U-2 OS cells stably expressing HaloTag7, HaloTag9, HaloTag10, or HaloTag11 in the cytosol. Cells were labeled with different fluorophores (1 μm, 3 h) and imaged by confocal microscopy. SiR-CA (**a**), JF_635_-CA (**b**), JF_629_-CA (**c**), JF_626_-CA (**d**), JF_614_-CA (**e**), CPY-CA (**f**), MaP618-CA (**g**), TMR-CA (**h**), and MaP555-CA (**i**). Distribution = light gray/blue, box = 25%–75% percentile, whiskers = 5%–95% percentile, white line = median, circle = mean, dashed line = mean of HaloTag7, *N* = 120, 121, 120, 121 (SiR); 120, 121, 120, 120 (JF_635_-CA); 129, 123, 114, 117 (JF_629_-CA); 121, 120, 120, 120 (JF_626_-CA); 120, 124, 120, 120 (JF_614_-CA); all 120 (CPY-CA); 120, 120, 120, 121 (MaP618-CA); 120, 120, 120, 121 (TMR-CA); 121, 120, 120, 120 (MaP555-CA) cells (left to right) from three independent preparations (Supplementary Table 16). Source data

The brightness of the labeled HaloTag variants relative to HaloTag7 was tested in living U-2 OS cells using 9 of the 14 rhodamines showing increased brightness with HaloTag9 compared with HaloTag7 in vitro. All except one fluorophore showed notably higher brightness when bound to HaloTag9 instead of HaloTag7. HaloTag10 and HaloTag11 decreased the brightness of all fluorophores relative to HaloTag7, HaloTag10 having the biggest effect (Extended Data Fig. 3 and Supplementary Table 16). Photostability measurements showed that the higher the fluorescence lifetime, the more photobleaching occurred, making HaloTag9 the least and HaloTag10 and HaloTag11 the most photostable tags (Supplementary Figs. 13–14 and Supplementary Table 17). However, the difference in photostability between HaloTag7 and HaloTag9 is rather small and HaloTag9 is still suitable for stimulated emission depletion (STED) microscopy, which relies on high irradiation intensities (Supplementary Figs. 15 and 16). The increased brightness of HaloTag9 relative to HaloTag7 makes it an attractive tool for applications such as fluorescence correlation spectroscopy (FCS) measurements and fluorescence confocal microscopy (Supplementary Figs. 17 and 18).

### Fluorescence lifetime multiplexing

**Extended Data Fig. 4:**
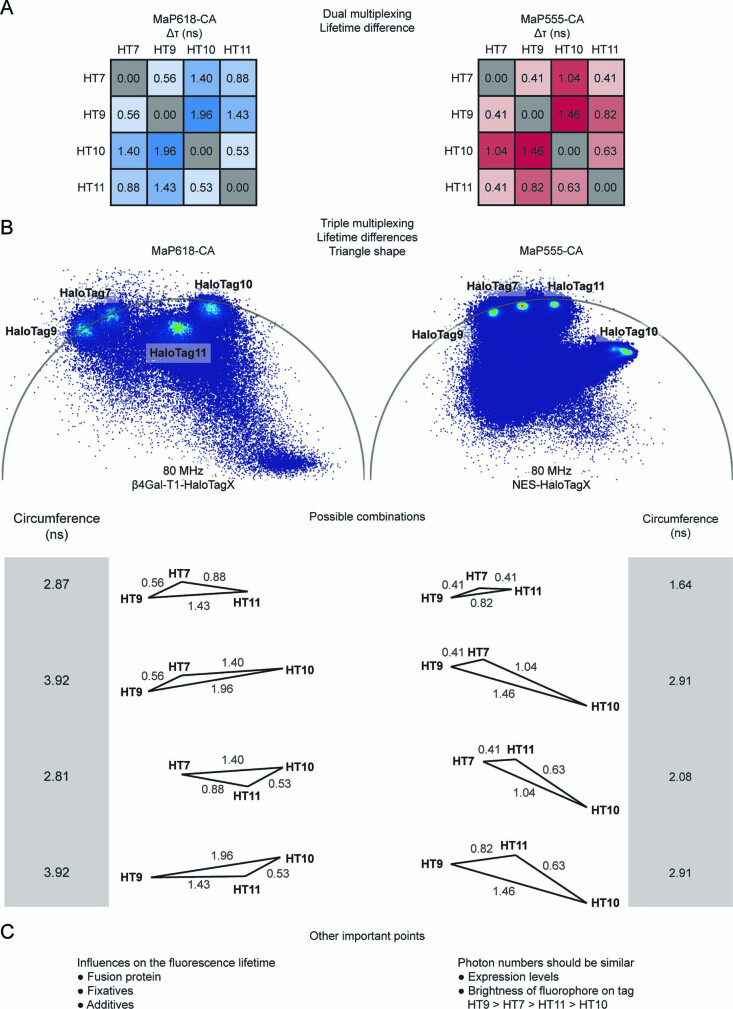
Guidelines for performing fluorescence lifetime multiplexing using HaloTag variants. **a** Overview over the differences in fluorescence lifetime between pairs of HaloTags using either MaP618-CA or MaP555-CA. For multiplexing of two species we generally recommend to use HaloTag9 and HaloTag11 (second/ third largest difference in fluorescence lifetime for MaP618-CA and MaP555-CA, respectively). **b** Representative phasor plots showing the clusters of HaloTag7, HaloTag9, HaloTag10, and HaloTag11 labeled with MaP618-CA or MaP555-CA. The triangles formed by three tags, their circumference, as well as the lifetime difference of the individual sides (ns) are given. In addition to the lifetime difference between the individual species, the shape of the triangle becomes important. Acute triangles (all angles α, β, γ < 90°) facilitate separation compared to obtuse triangles (one angle α > 90°), whose extreme is not a triangle but a straight line (α = 180°) and does not allow for separation of three species. For MaP618-CA the two combinations with the largest circumference correspond to obtuse triangles with α close to 180° (α » 90°) whereas the two combinations with the smaller circumference, even though still obtuse triangles, show α’s closer to 90° (α > 90°). A reasonable starting point would thus be to use HaloTag7, HaloTag9, and HaloTag11. For MaP555-CA the combination of HaloTag9, HaloTag10 and HaloTag11 shows both a large circumference and an α close to 90°. However, other factors are also of importance (see **c**). Separation of four components using the phasor approach solely based on lifetime information only becomes possible using transformation into higher harmonics^3^. **c** The outcome of a multiplexing experiment depends on other factors that influence fluorescence lifetime and fluorophore brightness. Fluorescence lifetime can also be influenced by environmental factors such as the fusion protein, subcellular compartment, or fixatives. We showed that the fluorescence lifetime of all presented HaloTag variants as well as HaloTag7 is relatively robust to change in fusion protein, subcellular localization and also PFA fixation (Extended Data Fig. 2, Supplementary Fig. 11-12 and 29-30). However, it is recommended to prepare single species control samples. A second factor that influences multiplexing experiment is the relative photon number collected for each species. If one species is providing less photons (for example due to brightness or expression level) compared to the other(s), the relative intensity obtained after separation will be more erroneous. It is therefore recommended that the highest expressing species should carry HaloTag10 (least bright) and the lowest expressing species should carry HaloTag9 (the brightest; brightness: HaloTag9 > HaloTag7 > HaloTag11 > HaloTag10, Extended Data Fig. 3).

We then used the tags for live-cell fluorescence lifetime multiplexing. Pairwise combinations of HaloTag7, HaloTag9, HaloTag10, or HaloTag11 were coexpressed as fusion proteins of the histone protein H2B, the outer mitochondrial membrane protein Tomm20 or the microtubule-binding protein CEP41, labeled with MaP618-CA and separated by fluorescence lifetime imaging microscopy (FLIM) using phasor analysis in living cells (Fig. 2b and Supplementary Figs. 19–24)^2^. Similarly, we performed fluorescence lifetime multiplexing in a different spectral window, labeling the two HaloTag variants with MaP555-CA instead of with MaP618-CA (Fig. 2c and Supplementary Figs. 25–28). In addition, separation was also possible in fixed cells (Supplementary Figs. 29–30). We then multiplexed combinations of three HaloTag variants using either MaP555-CA or MaP618-CA, allowing the separation of three species (Fig. 2d and Supplementary Figs. 31 and 32). While larger fluorescence lifetime differences facilitated separation, the relative brightness of the structures, defined by the HaloTag variant and the expression level of the species, also played an important role in separation. We therefore recommend the use of HaloTag9 and HaloTag11 for multiplexing of two species and HaloTag9, HaloTag10 and HaloTag11 for multiplexing of three species (Extended Data Fig. 4). To the best of our knowledge, this is the first example of multi-target FLIM using a single fluorophore on a subcellular level in living cells. Additionally, three-species images can also be accessed by multiplexing two HaloTag variants with the corresponding MaP-based F-actin, microtubule or DNA probes (Supplementary Figs. 33–34)^11^. Super-resolved images were acquired by STED–FLIM using only one depletion laser, while separating two species based on fluorescence lifetime information (Supplementary Fig. 35). Additionally, fluorescence lifetime multiplexing can be combined with spectrally resolved detection, which allowed us to multiplex six species in living cells within two spectral channels each with three lifetime components (Fig. 2e and Supplementary Fig. 36). For this, we expressed HaloTag7 as a fusion with the membrane-bound glycoprotein of the Golgi apparatus beta-1,4-galactosyltransferase (β4Gal-T1), HaloTag9 as a Tomm20 fusion, the lysosome-associated membrane glycoprotein 1 (LAMP1) as a HaloTag11 fusion, and the tyrosine protein kinase Lyn11, which localizes at the plasma membrane, as a SNAP-tag^19^ fusion, and combined them with two noncovalent MaP555 probes targeting F-actin and DNA^11^.

### Lifetime-based biosensors

**Fig. 3:**
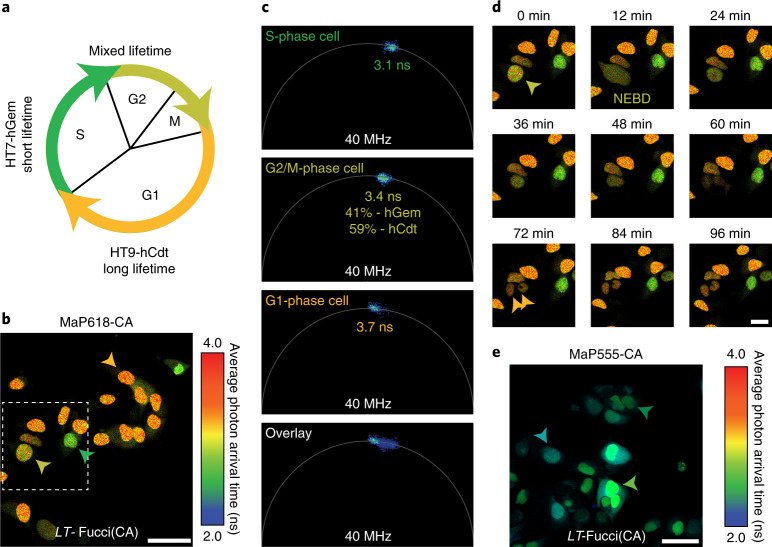
Lifetime-based Fucci biosensor using HaloTag variants. **a**, Schematic overview of the *LT*-Fucci(CA) biosensor. During the G1 phase, mainly HaloTag9-hCdt is present and the nuclei will therefore present long average photon arrival times (3.7 ns, orange). During S phase, HaloTag7-hGem is predominant, resulting in shorter average photon arrival times (3.1 ns, green) and during G2 and M phase a mixture of both will be present (~3.4 ns, light green). **b**, Representative FastFLIM image of U-2 OS cells stably expressing the *LT*-Fucci(CA) biosensor labeled with MaP618-CA (1 μM). Cells in three different cell stages can be found (orange arrowhead: G1, green arrowhead: S, light-green arrowhead: G2 and M). Scale bar, 50 μm. **c**, Fluorescence lifetime analysis of three nuclei from the image of *LT*-Fucci(CA) (**b**). The average fluorescence lifetime of the nuclei indicated was evaluated using phasor analysis and the respective clusters are given in the phasor plots. In addition, the overlay of all three phasor plots was used to evaluate the percentages of hGem and hCdt present in the G2/M-phase cell. As the fluorescence of a G2/M-phase cell shows contributions from the two individual components HaloTag7-hGem and HaloTag9-hCdt, the law of linear addition in phasor space can be applied. **d**, Enlargement of the dotted box in **b** showing the division of a cell over time. The dividing cell (light-green arrowhead), the two daughter cells (orange arrowhead) and the moment of nuclear envelope breakdown (NEBD) are indicated (Supplementary Video 1). Scale bars, 25 μm. **e**, *LT*-Fucci(CA) labeled with MaP555-CA (200 nM) generating a different color variant of the biosensor. The fluorescence lifetimes found for the different cell populations by phasor analysis were 2.9 ns (G1), 2.7 ns (G2/M) and 2.5 ns (S). Due to the lower fluorogenicity of MaP555-CA compared with MaP618-CA, there was also a larger contribution of background fluorescence. Scale bars, 50 μm.

**Extended Data Fig. 5:**
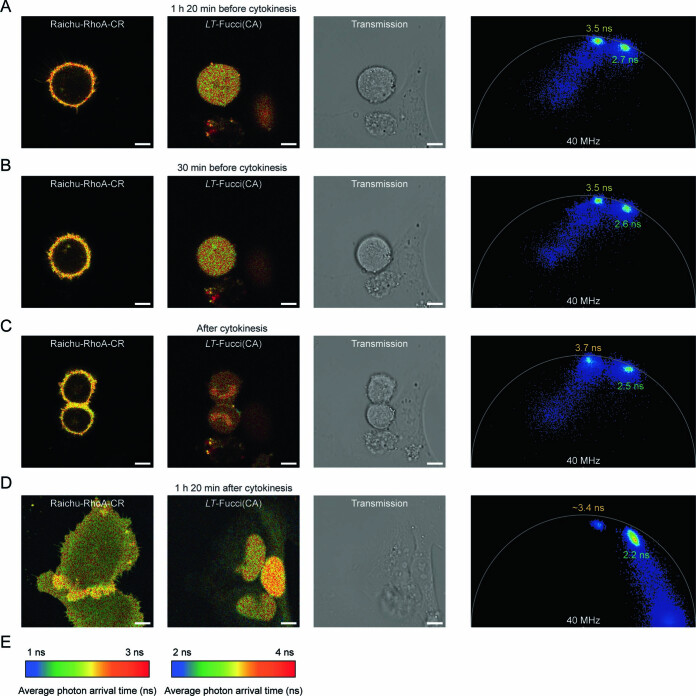
Multiplexed FLIM of the *LT*-Fucci(CA) and Raichu-RhoA-CR biosensors. **a-e** The activity of the GTPase RhoA as well as the cell cycle stage were followed over time for dividing cells. FLIM images are given for both Raichu-RhoA-CR and *LT*-Fucci(CA) labeled with MaP618-CA (1 μm) along with a transmission light image and the phasor plot 1 h 20 min before cytokinesis (**a**), 30 min before cytokinesis (**b**), shortly after cytokinesis (**c**), and 1 h 20 min after cytokinesis (**d**). The FLIM images are color-coded according to the scales in (**e**). The two biosensors could be easily multiplexed measuring fluorescence lifetime in the GFP channel (Clover-Raichu-RhoA-CR) and the CPY channel (*LT*-Fucci(CA)-MaP618). As expected mitotic cells initially showed low RhoA activity indicated by long donor fluorescence lifetimes (2.7 ns). However, closer to cytokinesis RhoA activity started to increase (2.6 ns) until a donor fluorescence lifetime of 2.5 ns was reached directly after cytokinesis. After division the cells reached even higher RhoA activity (2.2 ns)^69^. Scale bar, 10 μm.

Subsequently, we exploited the difference in fluorescence lifetime of HaloTag9 and HaloTag7 for the generation of a fluorescence lifetime-based biosensor to monitor cell cycle progression. Our design is based on Fucci biosensors, which rely on the cell cycle-dependent degradation of hCdt and Geminin (hGem) fragments fused to a green and red fluorescent protein (FP)^20^. Specifically, we developed lifetime-Fucci(CA) (*LT*-Fucci(CA)) by replacing the two FPs with HaloTag7 and HaloTag9. The cell cycle stage of U-2 OS cells stably expressing *LT*-Fucci(CA) was clearly indicated by the average photon arrival time of the nuclei upon labeling with MaP618-CA and could be followed over 24 h when providing the fluorogenic fluorophore continuously (3.7 ns, orange-G1; 3.1 ns, green-S; ~3.4 ns, light-green-G2/M; Fig. 3a–d and Supplementary Video 1). Phasor analysis of cells in the G2/M phase, in which both hCdt and hGem are present, allowed the relative amounts of the two proteins to be attributed (Fig. 3c). Due to the large fluorescence lifetime changes of *LT*-Fucci(CA)-MaP618, TauContrast imaging, a confocal technique without the need for FLIM instrumentation, could be used to assess the cell cycle stage^21^. Two additional *LT*-Fucci biosensors with alternative degradation cycles (*LT*-Fucci(SA) and *LT*-Fucci(SCA)) were developed (Supplementary Fig. 37 and Supplementary Videos 2and 3). Additionally, the color of the biosensor could be switched simply by labeling with MaP555-CA such that a lifetime of 2.9 ns corresponds to G1 phase, 2.5 ns to S phase and 2.7 ns to G2/M phase cells (Fig. 3e and Supplementary Fig. 38). As *LT*-Fucci biosensors occupy only one variable spectral channel, they are ideally suited for combination with other biosensors or probes. *LT*-Fucci(CA)-MaP618, for instance, can be combined with the green spectral region and, due to its narrow emission spectrum, also with the far-red region (Supplementary Fig. 38)^11,22^. We thus simultaneously performed FLIM measurements of *LT*-Fucci(CA)-MaP618 and the RhoA GTPase activity biosensor Raichu-RhoA-CR (Clover-mRuby2) during cell division (Extended Data Fig. 5)^23^. The flexibility to choose the color of the biosensor at the labeling step and the improved multiplexing capabilities sets *LT*-Fucci biosensors apart from the recently published FUCCI-Red (mKate2-mCherry)^22^.

## Discussion

The generation of HaloTag9, HaloTag10, and HaloTag11 from HaloTag7 allowed us to modulate the brightness and fluorescence lifetime of rhodamines for applications in fluorescence lifetime multiplexing. The changes in spectroscopic properties result from single or double point mutations in the direct vicinity of the bound fluorophore. The decreased quantum yields and fluorescence lifetimes of HaloTag10 and HaloTag11 presumably result from PET quenching through the newly introduced tryptophans. On the other hand, the mechanisms behind the increase in fluorescence lifetime observed for HaloTag9 are more complex, involving factors such as changes in electrostatic surface potential or the positioning of the fluorophore on the protein surface. Considering that only a limited number of point mutants was screened in this work, we believe that, by targeting other residues for mutagenesis, further increases in fluorescence lifetime of labeled HaloTag9 should be feasible.

The HaloTag variants developed enable live-cell fluorescence lifetime multiplexing of three species in a single spectral channel, freeing up other spectral channels for further multiplexing. Fluorescence lifetime multiplexing was demonstrated for combinations of various cellular targets, for different fluorophores and by combining two spectral channels to multiplex six species. Another possible advantage of using a single fluorophore for multiplexing is that it could eliminate challenges associated with differences in cell permeability, tissue penetration or metabolic clearance rates commonly encountered when using multiple fluorophores for multicolor experiments.

An additional application of the different HaloTag variants is their use in fluorescence lifetime-based Fucci biosensors, which were generated by fusing HaloTag variants with different fluorescence lifetimes to proteins with different cell-cycle-dependent degradation patterns. These biosensors occupy only one spectral channel that can be freely chosen and they can therefore be multiplexed with other biosensors in a straightforward manner.

Finally, it might be possible to generate other lifetime-orthogonal tags from SNAP-tag-fluorophore combinations or fluorogen activating proteins^6^, further expanding the options for fluorescence lifetime multiplexing. Moreover, SLP engineering might prove beneficial to modulate other photophysical properties of fluorophores, such as excitation and emission spectra, giving access to tailor-made SLPs for specific applications. It is expected that the exploitation of the synergistic interaction between synthetic fluorophores and the proteins to which they are bound will enable the generation of additional tools for bioimaging.

## Methods

### General considerations

Fluorophores-CA were either prepared according to literature procedures by B. Matthes or D. Schmidt (MPI-MR), kindly provided by L. Lavis (Janelia Research Campus), or purchased from commercial vendors (Supplementary Table 4). The synthesis of Cyanine3-CA, Cyanine5-CA, and Alexa647-CA is described in the Supplementary Methods. MaP618-Actin, MaP555-Actin, MaP555-DNA, MaP555-BG and MaP555-Tubulin were obtained from Spirochrome (SPY620 and SPY555). Fluorophores were prepared as stock solutions in dry DMSO and diluted in the respective buffer such that the final concentration of DMSO did not exceed 1% (v/v). Activity buffer (50 mm HEPES, 150 mm NaCl, pH 7.2) was used in all experiments unless otherwise stated, and 96-well plates (black, flat bottom, nonbinding (Corning)) were used unless otherwise stated. Fluorescence intensity was measured on a plate reader (Spark 20M, Tecan) equipped with filters and a monochromator. We performed excitation and emission collection as indicated in Supplementary Table 4. Enhanced green fluorescent protein (EGFP) was excited at 485/20 nm and emission was collected at 535/25 nm.

### Plasmids

A pET51b(+) vector (Novagen) was used for protein production in *E.* *coli*. Proteins were N-terminally tagged with His_x10_, followed by a tobacco etch virus (TEV) protease cleavage site (ENLYFQ | G). For in vitro screening, EGFP was fused to the C-terminus of HaloTag7. A pcDNA5/FRT/TO or a pcDNA5/FRT vector (ThermoFisher Scientific) was used for transient expression as well as stable cell line establishment in mammalian cells. HaloTag7 and its variants were fused to T2A-EGFP for expression in the cytosol. HaloTag7 and its variants were fused to CEP41, H2B, TOMM20, NES, COX8, CalR/KDEL, β4Gal-T1, LAMP1, SKL, Lyn11, Ig-κ-/PDGFR, or Lifeact for expression in mammalian cells. In addition, T2A-EGFP was fused C-terminally of HaloTag7 and its variants. For cotranslational (T2A) expression of two or three HaloTags, plasmids were cloned using three different codon optimizations for HaloTag7, HaloTag9, and HaloTag11; the latter two were obtained from gene synthesis (Eurofins). The Fucci sensors were constructed as follows: Fucci(SA): HaloTag7-Geminin(1–110)-P2A-HaloTag9-Cdt(30–120), Fucci(SCA): HaloTag7-Geminin(1–110)-P2A-HaloTag9-Cdt(1–100)Cy+, Fucci(CA): HaloTag7-Geminin(1–110)-P2A-HaloTag9-Cdt(1–100)Cy– (ref. ^20^), again two different codon optimizations were used for HaloTag7 and HaloTag9. We performed cloning by Gibson assembly^24^. DNA was subsequently electroporated in *E. cloni* 10G (Lucigene) and plated on agar Lysogenic Broth (LB) plates with 100 µg ml^–1^ ampicillin and incubated at 37 °C overnight. EGFP, T2A-EGFP (Addgene plasmid no. 135443)^25^, CEP41 (Addgene plasmid no. 135446)^25^, TOMM20 (Addgene plasmid no. 135443)^25^, H2B (Addgene plasmid no. 135444)^25^, COX8 (Addgene plasmid no. 113916)^26^, Ig-κ-/PDGFR^27^ and SNAPf (Addgene plasmid no. 167271)^17^ were available inhouse and used as template plasmids. mCherry-LaminB1-10 was a gift from M. Davidson (Addgene plasmid no. 55069), pAAV_hsyn_NES-his-CAMPARI2-F391W-WPRE-SV40 was a gift from E. Schreiter (Addgene plasmid no. 101061)^28^, pmTurquoise2-Golgi was a gift from D. Gadella (Addgene plasmid no. 36205)^29^, LAMP1-mGFP was a gift from E. Dell’Angelica (Addgene plasmid no. 34831)^30^, hGeminin(1–110) and hCdt(30–130): pLL3.7m-Clover-Geminin(1–110)-IRES-mKO2-Cdt(30–120) was a gift from M. Lin (Addgene plasmid no. 83841)^31^ and hCdt(1–100): pCSII-EF-miRFP709-hCdt(1–100) was a gift from V. Verkhusha (Addgene plasmid no. 80007)^32^; these were all used as template plasmids. pCAGGS-Raichu-RhoA-CR, a gift from M. Lin (Addgene plasmid no. 40258)^23^, was used directly for transient transfection. For more information see Supplementary Table 18. Plasmids generated in this work have been deposited with Addgene with accession codes listed in Supplementary Table 18.

### Protein production and purification

Proteins were expressed in the *E. coli* strain BL21(DE3)-pLysS. LB cultures were grown at 37 °C to an optical density at 600 nm (OD_600nm_) of 0.8, induced by the addition of 0.5 mm isopropyl-β-d-thiogalactopyranoside (IPTG) and grown at 17 °C overnight in the presence of 1 mM MgCl_2_. The cells were harvested by centrifugation (4,500*g*, 10 min, 4 °C) and lysed by sonication (5 min, cycle 5, 70%, SonoPlus Bandelin). The cell lysate was cleared by centrifugation (70,000*g*, 20 min, 4 °C). Proteins were purified using affinity-tag Ni-NTA (Qiagen) leading to purity higher than 95% (verified by SDS-PAGE Coomassie staining). Proteins were finally concentrated using an Ultra-0.5 ml centrifugal filter device (Amicon) with a molecular weight cut-off according to the protein size, followed by buffer exchange into activity buffer (<0.1 mM Imidazole). The proteins were stored in a glycerol 45% solution at −20 °C or flash frozen and stored at −80 °C. Protein amino acid sequences are listed in the Supplementary Methods.

For X-ray crystallography, proteins were produced as described above but purified using a HisTRAP FF affinity column (GE-Healthcare) on an ÄKTAPure M FPLC (GE-Healthcare). The proteins were concentrated using an Ultra-4mL centrifugal filter device (Amicon) and diluted to a final concentration of around 0.3 mg ml^−1^ (around 40 ml) in TEV-cleavage buffer (25 mM Na_2_HPO_4_, 200 mM NaCl). β-mercaptoethanol (10 μl) and TEV protease (mass ratio substrate:TEV 30:1, TEV protease produced and purified inhouse by A. Bergner) were added and incubated at 30 °C overnight. The solution was filtered (0.22 μm) and the cleaved protein was harvested by reverse purification on a HisTRAP FF affinity column (GE-Healthcare), collecting the flow through. Proteins were concentrated using an Ultra-4mL centrifugal filter device (Amicon) and further purified by size exclusion chromatography on a HiLoad 26/600 Superdex 75 pg column (GE-Healthcare) exchanging the buffer to activity buffer. Proteins were concentrated again and prepared to a final concentration of 5 μM in activity buffer; 3 mg of protein was incubated in presence of TMR-CA (10 μM) at room temperature overnight. The labeled protein was concentrated and an Illustra MicroSPin G-50 desalting column (GE-Healthcare) was employed to remove excess of unreacted fluorophore. The final protein concentration was adjusted to 13.0–15.0 mg ml^−1^ using the absorbance at 280 nm, correcting the extinction coefficient of the protein by *ε*_280, TMR-CA_ = 0.16.

### Protein crystallization

We performed crystallization at 20 °C using the vapor-diffusion method. HaloTag9, HaloTag10, and HaloTag11 were labeled with a TMR-CA fluorophore substrate and were concentrated to 13.0–15.0 mg ml^−1^ in 50 mM HEPES pH 7.3, 150 mM sodium chloride. Crystals of HaloTag9-TMR, HaloTag10-TMR, and HaloTag11-TMR were grown by mixing equal volumes of protein solution and a reservoir solution containing 0.1 M MES pH 6.0, 1.0 M lithium chloride and 20% (m/v) PEG 6000, 21% (m/v) PEG 6000 or 22% (m/v) PEG 6000, respectively. The crystals were washed briefly in cryoprotectant solution consisting of the reservoir solution with glycerol added to a final concentration of 20% (v/v), before flash-cooling in liquid nitrogen.

### X-ray diffraction data collection and structure determination

Single crystal X-ray diffraction data were collected at 100 K on the X10SA beamline at the SLS (PSI). Data were processed with XDS^33^. The structures of HaloTag9-TMR, HaloTag10-TMR, or HaloTag11-TMR labeled with TMR were determined by molecular replacement (MR) using Phaser^34^ and HaloTag7-TMR coordinates (6Y7A) as a search model. Geometrical restraints for TMR were generated using Grade server^35^. The final model was optimized in iterative cycles of manual rebuilding using Coot^36^ and refinement using Refmac5 (ref. ^37^) and phenix.refine^38^. Data collection and refinement statistics are summarized in Supplementary Table 14; model quality was validated with MolProbity^39^ as implemented in PHENIX.

Atomic coordinates and structure factors have been deposited in the Protein Data Bank under accession codes: 6ZVY (HaloTag7-Q156H-P174R-TMR = HaloTag9-TMR), 7PCX (HaloTag7-Q165W-TMR = HaloTag10-TMR) and 7PCW (HaloTag7-M175W-TMR = HaloTag11-TMR).

We performed structural analysis using PyMOL^40^, phenix^38^ and the APBS & PDB2PQR plug-in in Pymol using standard parameters (0.15 M ionic strength in monovalent salt, 310.0 K, protein dielectric of 2 and solvent dielectric of 78.0)^41^. Calculations of electrostatic surface potential at varying pH were performed using the PDB2PQR web service^42^.

### Polyacrylamide gel electrophoresis

HaloTag7 proteins (2 µM, 15 µl) were labeled using SiR-CA (10 µM) in activity buffer for 1 h at room temperature. After labeling, the proteins were separated by PAGE (4–20% ten-well Mini-Protean TGX, Bio-Rad) as recommended by the manufacturer and revealed by in gel fluorescence using a ChemiDoc MD Imaging System (Bio-Rad). SiR-CA labeled proteins were imaged using red epi illumination (695/55 nm), the proteins were revealed by Coommassie staining (Bio-Rad) and colorimetric imaging.

### Library generation

The plasmid libraries consisting of site-saturation mutagenesis performed on specific sites were prepared using degenerated primers according to Kille et al.^43^. The degenerated primers (Eurofins) were mixed in a ratio of NDT:VHG:TGG = 12:9:1 (N = any base, D = A, G, or T, V = A, C, or G, and H = A, C, or T) and used for PCR amplification of two DNA fragments of pET51b(+)-His-tev-HaloTag7-EGFP. The saturation site belonged to an overlapping sequence between two DNA fragments. Plasmid libraries were prepared via Gibson assembly^24^. After electroporation in the *E. coli* strain *E. cloni* 10 G (Lucigen), library diversity was evaluated by serial dilution, plating on selective LB agar plates (100 µg ml^−1^ ampicillin) at 37 °C overnight and verification that more than 1,000 transformants were obtained by colony counting. Concomitantly, the library was isolated by plasmid extraction from a selective liquid LB culture (100 µg ml^−1^ ampicillin) performed at 37 °C overnight (Qiagen kit). The plasmid libraries were sequenced by the Sanger method (Eurofins) to verify proper incorporation of degenerate codons. Libraries were employed to transform *E. coli* strain BL21(DE3)-pLysS that were plated on selective LB agar plates (100 µg ml^−1^ ampicillin) at 37 °C overnight. Single colonies were used to inoculate 400 µl selective LB medium (100 µg ml^−1^ ampicillin) in a 96-deep well plate. Five wells were reserved for parental HaloTag7, five wells for CLIP-tag as a negative control and eight wells for sterility controls. The bacterial cultures were incubated at 37 °C overnight and 500 rpm. Then, 50 µl of the stationary phase cultures were employed to inoculate 950 µl selective LB medium (50 µg ml^−1^ ampicillin) in a 96-deep-well plate and incubated at 37 °C for 4 h at 500 rpm. The remaining culture was centrifuged (5,000*g*, 15 min, 4 °C) and stored at 4 °C. Protein expression was induced by addition of 0.5 mm IPTG and grown at 17 °C overnight at 500 rpm. The cells were harvested by centrifugation (5,000*g*, 15 min, 4 °C). The bacterial pellets were submitted to two cycles of freeze/thawing before resuspension in 300 μl lysis buffer (50 mM K_2_HPO_4_ pH 8, 1 mg ml^−1^ lysozyme, 2 mM MgCl_2_ and 2.5 units ml^−1^ benzonase (Turbo Nuclease, Jena Bioscience)) at 37 °C for 1 h. The cell lysate was cleared by centrifugation (5,000*g*, 20 min, 4 °C). The cleared supernatant was transferred into nonbinding black bottom 96-well plates for the screening assays.

### Screening assay

Cell lysates (20 μl) were diluted in a nonbinding black bottom 96-well plate into activity buffer (100 μl final, 0.5 mg ml^−1^ bovine serum albumin (BSA; Sigma)). Background SiR (620/20 excitation, 680/30 emission) and GFP (485/20 excitation, 535/25 emission) fluorescence intensity were measured before spiking SiR-CA (5 μl) in each well (5 nM final SiR-CA concentration). After incubation at room temperature for 1 h, fluorescence intensities were measured again. We performed an additional second labeling step as previously described, reaching 10 nM final SiR-CA concentration and intensities were again measured. The five control wells allowed to access the mean and s.d. GFP and SiR fluorescence intensities of the parental protein. Wells with GFP intensities lower than 10% of the control were discarded for the screening (expression too low). Wells with SiR fluorescence intensities three or two times s.d. smaller/larger than the control (first round: mean_par_ ± 3 s.d._par_, second and third round: mean_par_ ± 2 s.d._par_) were selected for further characterization. Plasmids of selected wells were obtained from stored bacterial cultures and sequenced. Selected variants for characterization were produced and purified from 50 ml selective LB cultures (as described above).

Each protein (1 μM) was labeled with SiR-CA in 100 μl activity buffer (containing 0.5 mg ml^−1^ BSA (Sigma)) in a nonbinding black bottom 96-well plate and incubated for 2 h at room temperature. The SiR and GFP fluorescence intensities were measured as previously described. Measurements were performed in triplicate, performing the independent labeling reactions in three separate wells. Mean and 90% confidence intervals were calculated for every variant and compared with those of the parental protein. Variants with significant changes in SiR fluorescence intensity compared with the parental protein were picked for further characterization (one-sided *t*-test, *α* = 5%, d.f. = 4).

### Fluorescence intensity characterization

The most promising variants were subcloned into a pET51b(+) vector without the C-terminal EGFP fusion. Variant proteins were produced and purified from selective LB cultures (500 ml) as described above.

Fluorophores (Supplementary Table 4) were distributed into a nonbinding black bottom 96-well plate (100 μl, 100 nM) and incubated at room temperature overnight. The next day, 100 μl protein (2 μM, activity buffer containing 0.5 mg ml^−1^ BSA (Sigma)) was added to the fluorophore and incubated for 4 h at room temperature. The respective fluorescence intensities were measured with a plate reader (TECAN Spark 20M). The labeling and measurements were performed in quadruplicates. Mean and 95% confidence intervals were calculated for every variant and compared with those of the parental protein (one-sided *t*-test, *α* = 5%, d.f. = 6). Fluorescence excitation and emission spectra were measured using a plate reader (TECAN Spark 20M).

### D_50_ measurements

The free acid of the fluorophore (5 μM) was diluted in 200 μl water-dioxane mixtures of 0%, 10%, 20%, 30%, 40%, 50%, 60%, 70%, 80%, 90%, and 100% in clear-bottom polypropylene 96-well plates (Greiner Bio-One). Absorbance spectra were recorded on a plate reader (TECAN Spark 20M) from 400 nm to 700 nm. Measurements were performed in triplicate. Data were baseline corrected and the absorbance values at the maximal absorbance wavelength (*λ*_max_) were plotted against the dielectric constants of water-dioxane mixtures^44^. The data were fitted with a sigmoidal curve (1) and the *D*_50_ was determined as the point of inflection (*x*_c_).

1 $$y\left( x \right) = \frac{a}{{1 + {\mathrm{e}}^{ - k(x - x_c)}}}$$

### Kinetics by plate reader

Labeling kinetics of HaloTag variants (80 nM) were measured by time course fluorescence anisotropy measurements using TMR-CA (20 nM) in 200 µl activity buffer supplemented with 0.5 mg ml^−1^ BSA at room temperature and on a plate reader (TECAN Spark 20M) using the above stated filters for TMR and an injector system. *G*-factor and gain were calculated from three control measurements (buffer only, fluorophore in buffer, fully labeled protein in buffer). The data were fitted with a monoexponential function (2), where *y*_0_ corresponds to the *y* offset, *x*_0_ to the x offset, *A* to the amplitude and *τ* to the time constant. Using equation (3), the apparent second-order rate constant (*k*_app_) was calculated using the initial protein concentration [*P*]_0_. Fitted parameters are reported as means from at least two measurements.

2 $$y(x) = y_0 + A\cdot {\mathrm{e}}^{\frac{{ - (x - x_0)}}{\tau }}$$

3 $$k_{\mathrm{app}} = \frac{1}{{\tau \times [P]_0}}$$

### Kinetics by stopped-flow

Labeling kinetics of HaloTag7 and HaloTag9 with TMR-CA were measured by recording fluorescence anisotropy changes over time using a BioLogic SFM-400 stopped-flow instrument (BioLogic Science Instruments) in single mixing configuration at 37 °C. Monochromator wavelengths for excitation was set to 555 nm and a 570-nm long pass filter was used for detection. Protein and substrates were mixed in a 1:1 stoichiometry in activity buffer supplemented with 0.5 mg ml^−1^ BSA. Concentrations were varied from 0.125 µM to 0.5 µM. The anisotropy of the free substrate was measured to obtain a baseline. The dead time of the instrument was measured according to the manufacturer’s protocol (BioLogic Technical note no. 53) by recording the fluorescence decay during the pseudo first-order reaction of *N*-acetyl-l-tryptophanamide with a large excess of *N*-bromosuccinimide and fitting the data to the first-order reaction rate law. Recorded data were processed removing pretrigger time points and averaging replicates. The data were fit to a two-stage kinetic model (equations (4) and (5)) using the DynaFit software^45^. Baseline anisotropy of the free fluorophore, substrate concentrations and dead time of the instrument were taken into account. The s.d. (normal distribution verified) and confidence intervals of fitted parameters were estimated with the Monte Carlo method^46^ with standard settings (*N* = 1,000, 5% worst fits discarded). The derived parameters *K*_D_ (dissociation constant) and *k*_app_ (apparent second-order rate constant) were calculated according to equations (6) and (7).

4 $$P + S\begin{array}{*{20}{c}} {\mathop { \leftrightarrow }\limits^{k_1} } \\ {k_{ - 1}} \end{array}PS^ \ast$$

5 $$PS^ \ast \mathop { \to }\limits^{k_2} PS$$

6 $$K_\mathrm{D} = \frac{{k_{ - 1}}}{{k_1}}$$

7 $$k_{\mathrm{app}} = k_1\frac{{k_2}}{{k_2 + k_{ - 1}}}$$

### Thermostability

Thermostability of His-tev-HaloTag7, His-tev-HaloTag9, His-tev-HaloTag10, or His-tev-HaloTag11 was measured at 0.5 mg ml^−1^ in activity buffer on a nanoscale differential scanning fluorimeter Prometheus NT 48 (NanoTemper) over a temperature range from 20 °C to 95 °C with a heating rate of 1 °C min^−1^ by monitoring changes in the ratio of the fluorescence intensities at 350 nm and 330 nm. The indicated melting temperature (mean ± s.d., *N* = 2 samples) corresponds to the point of inflection (maximum of the first derivative).

### Quantum yield

Quantum yields were determined using a Hamamatsu Quantaurus QY. Fluorophores (0.5 μM) were added directly to the target protein (2.5 μM) in activity buffer. After incubation for 4 h at room temperature, quantum yields were measured. Except for Cy-3, where proteins (5 μM) were labeled with fluorophores (1 μM) in activity buffer for 12 h at room temperature and an Illustra MicroSPin G-50 desalting column (GE-Healthcare) was employed to remove excess of unreacted fluorophore.

### Extinction coefficient measurements

Proteins (12 μM) were labeled with fluorophores (6 μM) in activity buffer for 3 h at room temperature. The absorbance spectra of labeled proteins (0.5 μM, 1.0 μM, 1.5 μM, 2.0 μM, and 3.0 μM) were recorded in clear-bottom nonbinding 96-well plates (200 μl) on a plate reader (TECAN Spark 20M) from 400 nm to 700 nm. Data were baseline corrected and the maximum absorbance values plotted against concentration. The data were fitted to a linear function (equation (8)) and the extinction coefficients were calculated from the slope *b*.

8 $$y\left( x \right) = a + bx$$

### Computational chemistry

We optimized the quinoid and the spirolactone form of TMR at the B3LYP/6-31G(d,p) level of theory using the software package Gaussian v.16 (ref. ^47^). Solvent effects were modeled using the polarizable continuum model SMD. Molecules were visualized using the Avogadro software^48^.

We performed molecular modeling of TMR on the surface of HaloTag7 or HaloTag9 using MacroModel^49^ and the Protein Preparation Wizard^50^, both part of Maestro^51^ (Schrödinger Software). Relative energies were calculated by molecular modeling using the force field OPLS3e (ref. ^52^) in water constraining the protein as well as the remainder of the ligand apart from NMe_2_.

### Cell culture and transfection

U-2 OS (ATCC) and U-2 OS Flp-In T-REx Cep41-HaloTag7 (ref. ^25^) cells were cultured in high-glucose phenol-red free DMEM (Life Technologies) medium supplemented with GlutaMAX (Life Technologies), sodium pyruvate (Life Technologies) and 10% FBS (Life Technologies) in a humidified 5% CO_2_ incubator at 37 °C. Cells were split every 3–4 days or at confluency. Cell lines were tested regularly for mycoplasma contamination. Cells were seeded on eight-well glass bottom dishes (Ibidi) at three to one days before imaging. Transient transfections were performed using Lipofectamine 2000 reagent (Life Technologies) according to the manufacturer’s recommendations: the DNA (0.3 μg) was mixed with OptiMEM I (10 μl, Life Technologies) and Lipofectamine 2000 (0.75 μl) was mixed with OptiMEM I (10 μl). The solutions were incubated for 5 min at room temperature, then mixed and incubated for an additional 20 min at room temperature. The prepared DNA-Lipofectamine complex was added to one of the wells in an eight-well glass bottom dish with cells at 50–70% confluency. After 12 h incubation in a humidified 5% CO_2_ incubator at 37 °C, the medium was changed to fresh medium. The cells were incubated under the same conditions for 24–48 h before imaging.

### Stable cell line establishment

The Flp-In T-REx System (ThermoFisher Scientific) was used to generate stable cell lines exhibiting tetracycline-inducible expression of the gene of interest (GOI). Briefly, pcDNA5-FRT-TO-GOI or pcDNA5-FRT-GOI and pOG44 were cotransfected into the host cell line U-2 OS Flp-In T-REx^53^. Homologous recombination between the Flp recombination target (FRT) sites in pcDNA5-FRT-TO-GOI and the host cell chromosome, catalyzed by the Flp recombinase expressed from pOG44, produced the U-2 OS Flp-In T-REx cells expressing stable and inducible GOI. Stable cell lines were selected using 100 μg ml^–1^ hygromycin B (ThermoFisher Scientific) and 15 μg ml^–1^ blasticidine (ThermoFisher Scientific), seeded on glass bottom dishes as described in the previous section and induced if necessary using 100 μg ml^–1^ doxycycline (Sigma Aldrich) for 24–48 h before imaging. A list of all established cell lines can be found in Supplementary Table 18.

### Fixation

Fixation was performed as follows: cells were prefixed in 2.4% (w/v) formaldehyde (PFA) in PBS for 45 s, permeabilized in 0.4% (v/v) Triton X-100 in PBS for 3 min and fixed in 2.4% (w/v) PFA in PBS for 30 min. PFA was quenched by 100 mM NH_4_Cl in PBS for 5 min. After washing three times for 5 min in PBS, the cells were labeled as described below.

### Labeling and sample preparation

Cells were labeled with the respective fluorophores (Fluorophore-CA 1–2 μM, 1–3 h, 37 °C; or MaP555-BG (Spirochrome) 2 μM, 3 h, 37 °C; MaP618-Actin (Spirochrome), MaP555-Actin (Spirochrome) 0.5–2 μM, 3 h, 37 °C; or MaP555-DNA (Spirochrome), MaP555-Tubulin (Spirochrome) 1 μM, 3 h, 37 °C) in phenol-red free DMEM medium supplemented with GlutaMAX, sodium pyruvate and 10% FBS (all Life Technologies), washed with the same medium (twice for 1 min, 37 °C) except for actin, tubulin, DNA probes and Fucci experiments for which labeling and imaging were performed in the same medium.

### Confocal microscopy

Confocal fluorescence microscopy was performed on a Leica SP8 FALCON microscope (Leica Microsystems) equipped with a Leica TCS SP8 X scanhead; a SuperK white light laser, Leica HyD SMD detectors, a HC PL APO CS2 ×20/0.75 dry objective, a HC PL APO CS2 ×40/1.10 water objective and a water immersion microdispenser. Emission was collected as indicated in Supplementary Table 19. The microscope was equipped with a CO_2_ and temperature controllable incubator (Life Imaging Services, 37 °C).

For brightness comparison, stable cell lines expressing HaloTag variants or HaloTag7 in the cytosol were seeded and labeled as described above. Cells were focused in the GFP channel and z-stacks were recorded with 1 μm step size over 22 μm. The summed stacks were analyzed as follows: the mean intensity of a rectangular region of interest (ROI) within the cell was normalized by the GFP intensity using a custom written Fiji macro^54,55^.

Images of Cep41 were acquired transiently by transfecting U-2 OS cells with CEP41-HaloTag7-T2A-EGFP or CEP41-HaloTag9-T2A-EGFP and labeling them as described above. Cells were focused in the GFP channel and z-stacks were recorded. The intensity of the MaP618-CA signal was normalized by the EGFP signal, and the resulting values compared for the two HaloTags. Cep41-Halo images were rescaled to the expression levels using the EGFP intensity values from within a ROI over the entire cell area and depicted using the same brightness and contrast settings.

Photostability measurements were performed using a PMT detector to collect emission. Stable cell lines expressing HaloTag7, HaloTag9, HaloTag10, or HaloTag11 as H2B fusions were seeded and labeled as described above. Cells were focused and a z-stack was recorded with 2 μm step size over 22 μm, using a pinhole of 5 Airy Unit (AU) and 2% (630 nm, SiR), 1%, 1.5% or 5% (615 nm, CPY, MaP618, or JF_614_) and 2% (555 nm, TMR, MaP555) laser intensity. This was followed by the acquisition of eight consecutive photobleaching frames in the focal plane at 100% laser intensity. Z-stack and photobleaching was repeated 60 times. The summed stacks were analyzed as follows: the mean intensity of ROIs around the nuclei were normalized to the mean intensity found at t_0_.

Fluorescence excitation and emission spectra of MaP555-CA, MaP555-Actin, MaP555-Tubulin, MaP555-DNA, MaP555-SNAP, MaP618-CA, MaP618-Actin, and MaP618-DNA were measured in live U-2 OS cells expressing HaloTag variants in the cytosol or blank U-2 OS cells. MaP555 excitation: exciting at 475–575 nm in 2-nm steps collecting at 595–700 nm. MaP555 emission: exciting at 520 nm and collecting at 530–627 nm in 3-nm steps with a bandwidth of 10 nm. MaP618 excitation: exciting at 550–650 nm in 2-nm steps collecting at 670–780 nm. MaP618 emission: exciting at 600 nm and collecting at 610–707 nm in 3-nm steps with a bandwidth of 10 nm.

### FCS measurements

FCS was performed on a Leica SP8 FALCON microscope (as described above) using a HC PL APO CS2 ×40/1.10 water objective with a motorized correction collar. FCS traces (30 s) were measured in U-2 OS cells expressing either HaloTag7 or HaloTag9 in the cytosol (no induction). The cells were labeled with MaP618-CA (150 nM, 2 h) and washed twice for 1 min. Excitation and emission collection was performed as indicated in Supplementary Table 19. Five traces per cell and a total of 36 cells from three biological replicates were measured. Data analysis (correlation and fitting) was performed using the LAS X software (Leica Microsystems) fitting a free three-dimensional diffusion model including a triplet component^56^. The diffusion amplitude (*G*(0)) as well as the mean photon counts (PC) over the 30 s trace were used to calculate the molecular brightness (*mB*, equation (9)).

9 $$mB = G\left( 0 \right) \times {{{\mathrm{PC}}}}$$

### STED microscopy

Imaging was performed on an Abberior easy3D STED/RESOLFT QUAD scanning microscope (Abberior Instruments) built on a motorized inverted microscope IX83 (Olympus). The microscope was equipped with a pulsed STED lasers at 775 nm, and with a 640-nm excitation laser. Spectral detection was performed with avalanche photodiodes (APD) in the following spectral window: 650–725 nm. Images were acquired with a ×100/1.40 UPlanSApo Oil immersion objective lens (Olympus). Pixel size was 25 nm for all images. Laser powers and dwell times were kept constant so HaloTag9 and HaloTag7 images were comparable.

For quantification of microtubule diameter, the perpendicular line profile of a 200-nm-wide microtubule section was measured. The profile was fitted with a Gaussian function (equation (10)) and the full-width at half-maximum (FWHM) derived thereof (equation (11)).

10 $$y = y_0 + \frac{A}{{w\sqrt {\frac{\uppi }{2}} }}{\mathrm{e}}^{ - 2\frac{{(x - x_c)^2}}{{w^2}}}$$

11 $$\mathrm{FWHM} = w\sqrt {2{{{\mathrm{ln}}}}(2)}$$

For STED laser only images, cells were focused using excitation only, followed by the sequential acquisition of a STED laser only image (excitation laser: off, STED laser: on) and a normal STED image (excitation laser: on, STED laser: on).

For photobleaching measurements, 31 consecutive STED frames were acquired and the intensity within a rectangular ROI was compared over time. Movement of mitochondria out of, or into, the ROI was neglected.

### Fluorescence lifetime imaging microscopy

FLIM was performed on a Leica SP8 FALCON microscope (as described above) at a pulse frequency of 80 MHz unless otherwise stated. Emission was collected as indicated in Supplementary Table 19.

For determination of fluorescence lifetime, cells stably expressing HaloTag variants in the cytosol were imaged, collecting 1,000 photons per pixel. The acquired images of cells were thresholded to remove background signal from empty coverslip space. Mean fluorescence lifetimes were calculated in the LAS X software (Leica Microsystems) by fitting mono, bi, or triexponential decay models (*n*-exponential reconvolution) to the decay (*χ*^2^ < 1.2).

For determination of average fluorescence lifetimes on different subcellular targets, cells were transiently transfected with the HaloTag constructs and imaged, collecting 500 photons per pixel. The acquired images were processed as described above.

Structural images (species separation) were acquired as indicated in Supplementary Table 19 and species separation was performed via phasor analysis, positioning the cluster-circles on the phasor plot at the position of the pure species (Leica Microsystems)^2,57,58^. Images in Supplementary Fig. 24 were analyzed using Pattern Matching in SymPhoTime64 (PicoQuant).

Long-term cell cycle measurements were performed using the Navigator function of the SP8. Cells were labeled with MaP618-CA (1 μM, no wash) and imaged after 1 h. FLIM images were acquired every 12 min and an autofocus z-stack measurement was performed before every image. Water immersion was controlled using the water immersion microdispenser. Biosensor multiplexing was performed on the same setup, acquiring FLIM images in both channels every 5 min for 2–4 h. Images were analyzed in LAS X (Leica Microsystems) applying pixel binning (equation 2). FastFLIM images (average photon arrival times per pixel) are shown. Additionally, we used phasor analysis to evaluate the different cell populations.

### TauContrast microscopy

TauContrast microscopy was performed on a STELLARIS 8 FALCON microscope (Leica Microsystems, FALCON system only used for comparison). The microscope was equipped with a White Light Laser with tunable excitation wavelengths 440–790 nm operating at 80 MHz. Spectral detection was performed with Power HyD X photon-counting detectors in the following spectral window: 630–700 nm. Images were acquired with a ×86/1.20 STED WHITE water immersion objective lens (Leica Microsystems). Pixel size was 176 nm for all images. TauContrast images were analyzed using the LAS X software. For comparison FastFLIM images were acquired simultaneously using the FALCON system.

### STED–FLIM microscopy

Line sequential, confocal/STED–FLIM imaging was performed on a STELLARIS 8 STED FALCON microscope (Leica Microsystems). The microscope was equipped with a White Light Laser with tunable excitation wavelengths 440–790 nm operating at 80 MHz, and a pulsed STED laser at 775 nm operating at 80 MHz. Spectral detection was performed with Power HyD X photon-counting detectors in the following spectral window: 630–760 nm. Images were acquired with a ×86/1.20 STED WHITE water immersion objective lens (Leica Microsystems). Pixel size was 20 nm for all images. Images were analyzed using species separation via phasor analysis available in FALCON through the LAS X software.

### Software and image processing

Statistical analysis as well as curve fitting was performed using Microsoft Excel 2016, OriginLab^59^ or R^60^ including packages readxl^61^, fBasics^62^, tidyverse^63^, gghighlight^64^, ggplot2 (ref. ^65^), ggrepel^66^, ggpubr^67^ and broom^68^. All images were processed with ImageJ/Fiji^54,55^ and macros written therein unless otherwise stated.

### Statistics and reproducibility

Representative microscopy images are shown and unless otherwise stated experiments were performed on three different days.

### Reporting Summary

Further information on research design is available in the Nature Research Reporting Summary linked to this article.

## Online content

Any methods, additional references, Nature Research reporting summaries, source data, extended data, supplementary information, acknowledgements, peer review information; details of author contributions and competing interests; and statements of data and code availability are available at 10.1038/s41592-021-01341-x.

## Supplementary information

Supplementary Information Supplementary Figs. 1–38, Tables 1–19, Videos 1–3, Methods and References.

Reporting Summary

Supplementary Video 1. *LT*-Fucci(CA) biosensor over 24 h in living U-2 OS cells.

Supplementary Video 2. *LT*-Fucci(SA) biosensor over 24 h in living U-2 OS cells.

Supplementary Video 3. *LT*-Fucci(SCA) biosensor over 24 h in living U-2 OS cells.

## Data Availability

Plasmids encoding HaloTag variants and fusions thereof have been deposited with Addgene. Accession codes can be found in Supplementary Table 18. The X-ray crystal structures of HaloTag9-TMR, HaloTag10-TMR, and HaloTag11-TMR have been deposited with PDB under deposition codes 6ZVY, 7PCX, and 7PCW. HaloTag7-TMR is available at PDB with deposition code 6Y7A. Correspondence and requests for materials should be addressed to K.J. Source data are provided with this paper. The data supporting the findings of this study are available within the paper and its Supplementary Information and are available from the corresponding author upon reasonable request.

## Source data

Source Data Extended Data Fig. 3 Statistical source data for violin plots.

## Code availability

Custom written Fiji macros are available upon request to K.J.
