# Supplementary material for: Engineered HaloTag variants for fluorescence lifetime multiplexing
Source: Nat Methods. 2021 Dec 16;19(1):65–70. doi: 10.1038/s41592-021-01341-x (PMC8748199; doi:10.1038/s41592-021-01341-x)
Supplement: Supplementary file 1 — Supplementary Figs. 1–38, Tables 1–19, Videos 1–3, Methods and References. [file 41592_2021_1341_MOESM1_ESM.pdf]

nature portfolio

https://doi.org/10.1038/s41592-021-01341-x

Supplementary information

Engineered HaloTag variants for fluorescence lifetime multiplexing

In the format provided by the authors and unedited

Supplementary information

Engineered HaloTag variants for fluorescence lifetime multiplexing

Michelle S. Frei[1-2], Miroslaw Tarnawski[3], M. Julia Roberti[4], Birgit Koch[1], Julien Hiblot[1], Kai Johnsson[1-2]

1 Department of Chemical Biology, Max Planck Institute for Medical Research, Jahnstrasse 29, 69120 Heidelberg, Germany

2 Institute of Chemical Sciences and Engineering (ISIC), École Polytechnique Fédérale de Lausanne (EPFL), 1015 Lausanne, Switzerland.

3 Protein Expression and Characterization Facility, Max Planck Institute for Medical Research, Jahnstrasse 29, 69120 Heidelberg, Germany

4 Leica Microsystems CMS GmbH, Am Friedensplatz 3, 68163 Mannheim, Germany.

Corresponding Author: Kai Johnsson email: johnsson@mr.mpg.de

Table of Contents

Supplementary Figures

Figure S1: Library design based on the structure of HaloTag7-TMR. S4
Figure S2: Labeling kinetics of HaloTag variants with TMR-CA. S5
Figure S3: Comparison of the fluorescence brightness of HaloTag variants and HaloTag7. S6-7
Figure S4: Normalized fluorescence excitation and emission spectra of six fluorophores on HaloTags. S8
Figure S5: Crystal structure of HaloTag9-TMR, HaloTag10-TMR, and HaloTag11-TMR. S9-13
Figure S6: Structural analysis of the interactions of TMR at the crystal packing interface. S14-16
Figure S7: Modeled structures of the open (**A**) and the spirolactone form (**B**) of TMR in water together with their chemical structures. S17
Figure S8: Structural analysis of HaloTag7- or HaloTag9-TMR. S18
Figure S9: Electrostatic potential of HaloTag9-TMR and HaloTag7-TMR mapped unto to their solvent unto to their solvent excluded surface (Connolly surface). S19
Figure S10: Normalized fluorescence intensity of three fluorophores on the HaloTags at different pH. S20
Figure S11: Live-cell fluorescence lifetime microscopy of HaloTag7, HaloTag9, HaloTag10, or HaloTag11 fusion proteins labeled with MaP618-CA. S21-23
Figure S12: Live-cell fluorescence lifetime microscopy of HaloTag7, HaloTag9, HaloTag10, or HaloTag11 fusion proteins labeled with MaP555-CA. S24-26
Figure S13: Photostability of different fluorophores on the HaloTags assessed by confocal microscopy. S27-28
Figure S14: Photostability of different fluorophores on HaloTag7 and HaloTag9 assessed by STED microscopy. S29
Figure S15: Confocal and STED fluorescence microscopy images of mitochondria or microtubules labeled via HaloTag7 or HaloTag9. S30-31
Figure S16: STED-laser only and STED fluorescence microscopy images of microtubules labeled via HaloTag7 or HaloTag9. S32-33
Figure S17: Molecular brightness comparison of HaloTag7 and HaloTag9 in mammalian cells by FCS. S34
Figure S18: Brightness comparison of CEP41-HaloTag7 and CEP41-HaloTag9 in mammalian cells. S35
Figure S19: Live-cell fluorescence lifetime multiplexing of H2B and Tomm20 fused to different HaloTag variants labeled with MaP618-CA. S36
Figure S20: Live-cell fluorescence lifetime multiplexing of the inverse combinations of H2B and Tomm20 fused to different HaloTag variants labeled with MaP618-CA. S37-38
Figure S21: Live-cell fluorescence lifetime multiplexing of CEP41 and H2B fused to different HaloTag variants labeled with MaP618-CA. S39-40
Figure S22: Live-cell fluorescence lifetime multiplexing of CEP41 and Tomm20 fused to different HaloTag variants with MaP618-CA. S41-42
Figure S23: Live-cell fluorescence lifetime multiplexing of inverse combinations of fusion proteins with HaloTag variants. S43
Figure S24: Live-cell fluorescence lifetime multiplexing using pattern matching analysis to separate the two components. S44
Figure S25: Live-cell fluorescence lifetime multiplexing of H2B and Tomm20 fused to different HaloTag variants labeled with MaP555-CA. S45
Figure S26: Live-cell fluorescence lifetime multiplexing of the inverse combinations of H2B and Tomm20 fused to different HaloTag variants labeled with MaP555-CA. S46-47
Figure S27: Live-cell fluorescence lifetime multiplexing of CEP41 and H2B fused to different HaloTag variants labeled with MaP555-CA. S48-49
Figure S28: Live-cell fluorescence lifetime multiplexing of CEP41 and Tomm20 fused to different HaloTag variants labeled with MaP555-CA. S50

Figure S29: Fluorescence lifetime multiplexing in fixed cells labeled with MaP618-CA. S51
Figure S30: Fluorescence lifetime multiplexing in fixed cells labeled with MaP555-CA. S52
Figure S31: Live-cell fluorescence lifetime multiplexing of three HaloTags labeled with MaP618-CA. S53-56
Figure S32: Live-cell fluorescence lifetime multiplexing of three HaloTags labeled with MaP555-CA. S57-59
Figure S33: Live-cell fluorescence lifetime multiplexing of two HaloTag variants or HaloTag7 and one non-covalent probe. S60-62
Figure S34: Spectral characterization of MaP555 and MaP618 based fluorescent probes. S63
Figure S35: Fixed cell fluorescence lifetime multiplexing in STED microscopy. S64-65
Figure S36: Six species live-cell fluorescence lifetime multiplexing combined with spectrally resolved detection. S66
Figure S37: Characterization of the *LT*-Fucci biosensors. S67-68
Figure S38: Phasor analysis of the *LT*-Fucci(CA) biosensor. S69-70

Supplementary Tables

Table S1: Summary of HaloTag7 engineering. S71
Table S2: Validation of the engineered variants. S72
Table S3: Characterization of different HaloTag variants. S73
Table S4: Fluorophores used for characterization of the HaloTag variants. S74-79
Table S5: Comparison of the fluorescence intensity of different fluorophores on the three HaloTag variants relative to HaloTag7. S80
Table S6: Quantum yields (ϕ) of fluorophores on HaloTags. S81
Table S7: Extinction coefficients (ε) of fluorophores on HaloTags. S82
Table S8: Intensity weighted fluorescence lifetimes (τ) of fluorophores on HaloTags. S83
Table S9: Comparison of fluorophore properties on HaloTag9 and HaloTag7. S84
Table S10: Comparison of fluorophore properties on HaloTag10 (HaloTag7-Q165W) and HaloTag7. S85
Table S11: Comparison of fluorophore properties on HaloTag11 (HaloTag7-M175W) and HaloTag7. S86
Table S12: Structural analysis of TMR-labeled HaloTags. S87
Table S13: Comparison of the packing interface in HaloTag7-TMR and HaloTag9-TMR. S88
Table S14. Data collection and refinement statistics for the crystal structure of HaloTag9-TMR, HaloTag10-TMR, and HaloTag11-TMR. S89
Table S15: Comparison of intensity weighted fluorescence lifetimes (τ) of MaP618-CA and MaP55-CA on HaloTags fused to different protein of interest. S90
Table S16: Brightness comparison of different fluorophores on new HaloTag variants relative to HaloTag7 *in cellulo*. S91
Table S17: Photostability of HaloTags assessed by confocal microscopy. S92
Table S18: Plasmids used and generated as well as the stable cell lines derived thereof. S93-96
Table S19: Fluorescence microscopy data acquisition parameters. Fluorophores refer to CA analogues unless otherwise stated. S97-109

Supplementary Videos

Videos 1-3. *LT*-Fucci biosensors over 24 h in living U-2 OS cells. S110

Supplementary Methods

Protein sequences S110
Synthesis S110-112

References S112-115

Supplementary Figures

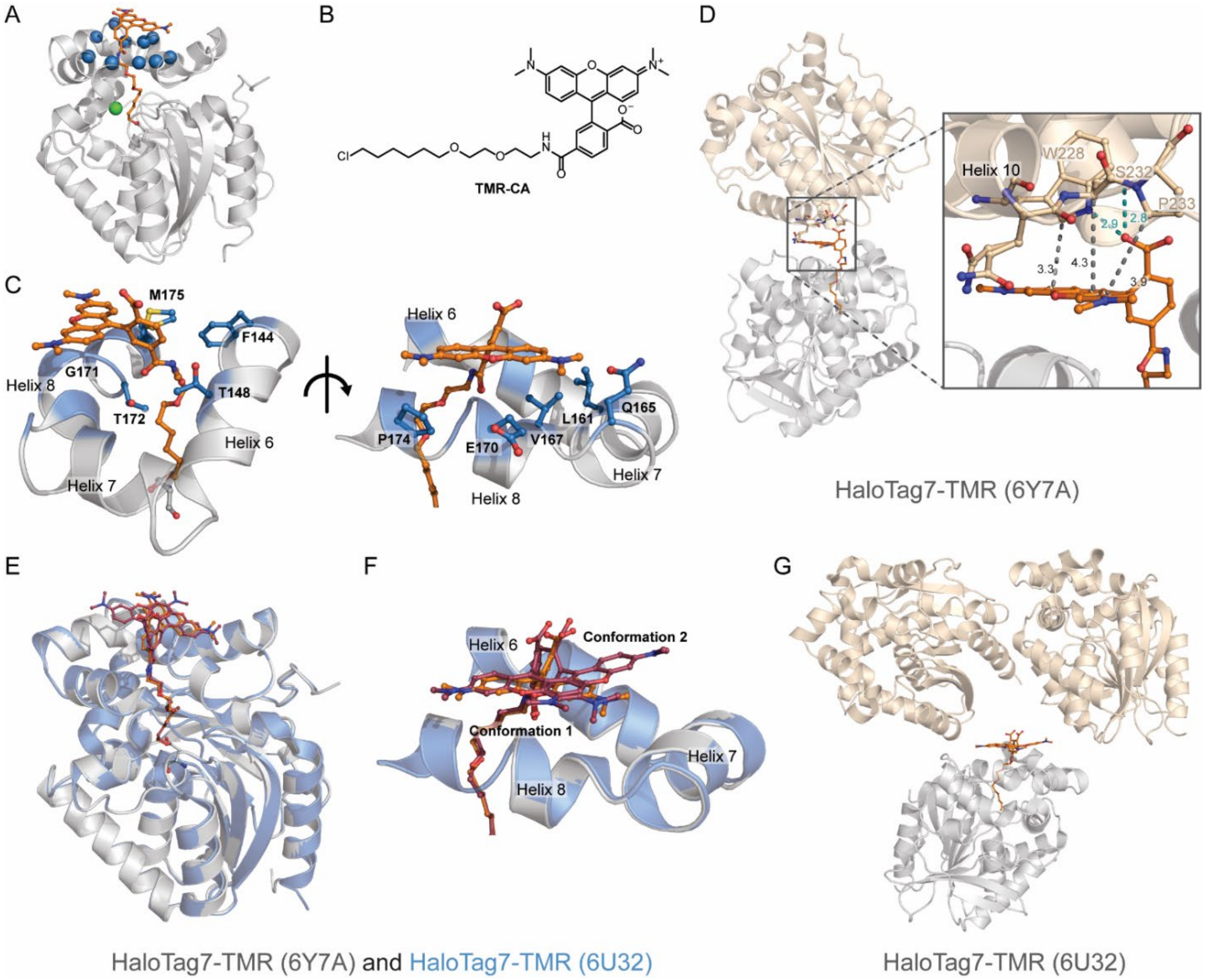

Supplementary Figure S1: Library design based on the structure of HaloTag7-TMR. **A** Crystal structure of HaloTag7-TMR (PDB ID: 6Y7A, 1.4 Å). The protein is represented as grey cartoon and TMR as orange sticks. The Cα of the ten amino acids chosen for site-saturation mutagenesis are highlighted as blue spheres. The chlorine atom is shown as a green sphere. **B** Chemical structure of TMR-CA. **C** TMR binding site (helices 6-8) on the HaloTag7-TMR crystal structure. Same structural representation as in **A**, but the ten amino acids' side chains are represented by blue sticks. **D** Interactions between two HaloTag7-TMR monomers within the crystal packing. The carboxylic acid of TMR is hydrogen bonded (teal dashes) to the next monomer via S232 and W228 (cream). In addition, the distances from W228 (Cδ), S232 (Cα), and P233 (Cδ) to the xanthene ring system (grey dashes) are given in Ångström. Only W228 comes close enough to the xanthene system to be involved in efficient van der Waals interactions. **E-F** Overlay (**E**) and zoom (**F**) of HaloTag7 (grey) and an alternative crystal structure of HaloTag7-TMR (PDB ID: 6U32, 1.8 Å)[1]. TMR is given in stick representation (orange (6Y7A) and violet (6U32)). The 6U32 structure shows two fluorophore conformations: one almost identical to the 6Y7A one (conformation 1), the second (conformation 2) places the xanthene core over the turn between helix 7 and helix 8. **G** Crystal packing of the 6U32 HaloTag7-TMR structure. The fluorophore is not constrained by neighboring monomers, allowing TMR to adopt conformation 2.

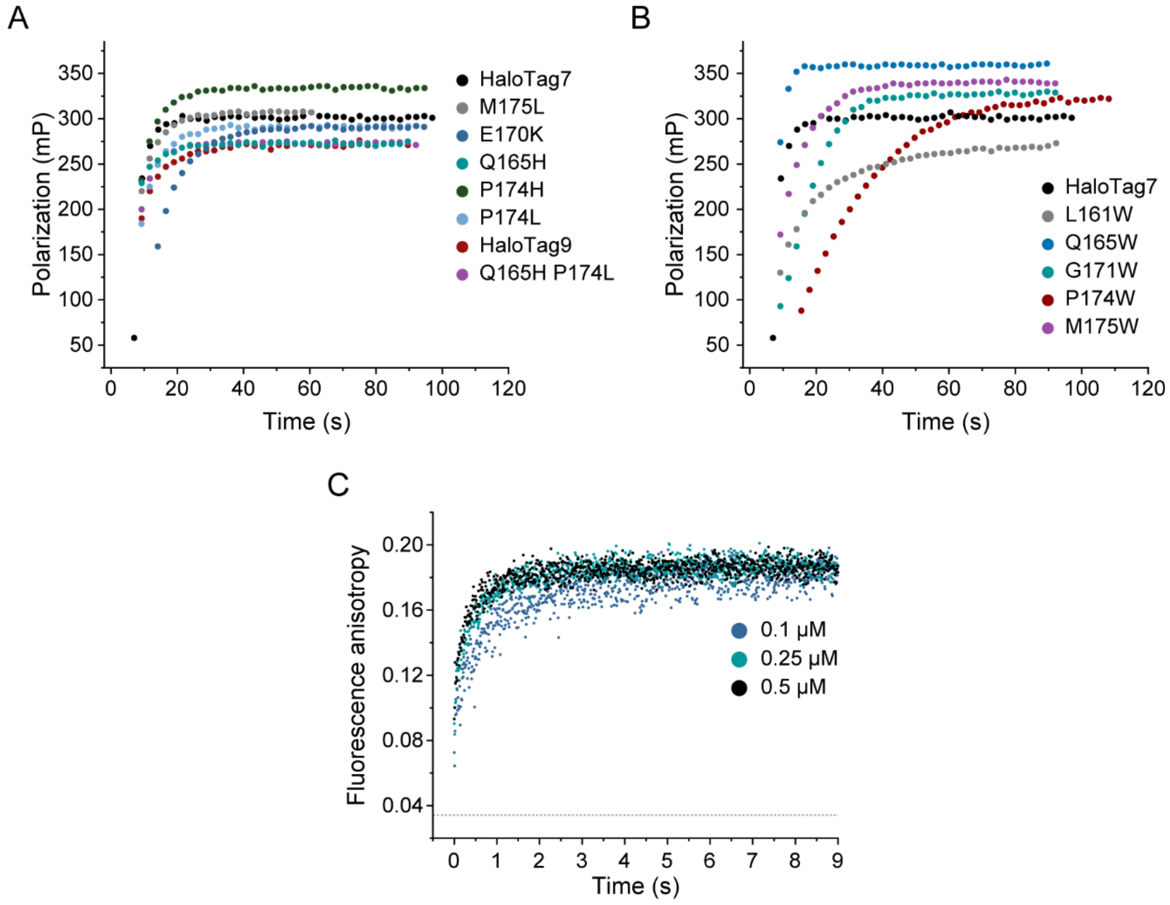

Supplementary Figure S2: Labeling kinetics of HaloTag variants with TMR-CA. **A-B** Plots of labeling kinetics of seven brighter (**A**) and six dimmer (**B**) HaloTag variants compared to HaloTag7. Kinetic measurements were performed by fluorescence polarization on a plate reader. Proteins (80 nM) were reacted with limiting amounts of TMR-CA (20 nM) to allow complete labeling of fluorophores. The data was fitted with a mono-exponential function.

The apparent first order rate constants k_{1app} and the polarization values reached after reaction completion were compared (Supplementary Table S3). Among the brighter variants only HaloTag7-E170K showed slower labeling kinetics than HaloTag7 and three of the dimmer variants did (HaloTag7-L161W, HaloTag7-G171W, and HaloTag7-P174W). Representative measurements of three replicates are displayed. **C** Labeling kinetics of HaloTag9. Kinetic measurements were performed by stop flow measurements. HaloTag9 was reacted with an equimolar amount of TMR-CA and the change in fluorescence anisotropy was followed. The measurement was performed with three different concentrations (0.1, 0.25 and 0.5 µM). The data was globally fit to a reaction model taking an initial association equilibrium (P + S ⇔ PS*) followed by an irreversible labeling reaction (PS* -> PS) into account. From this K_D, and k_{app} were calculated (Supplementary Table S3). Similar measurements were performed for HaloTag7 and attempted for HaloTag10 and HaloTag11. However, the large decrease in fluorescence intensity (decrease in quantum yield) of TMR-CA upon labeling HaloTag10 or HaloTag11 impaired the measurements over a larger concentration range (dynamic range of the detector not sufficient). Averaged measurements from at least 3 measurements are shown. The dashed line at 0.034 mP corresponds to the lower bound measured by a sample of free TMR-CA in solution.

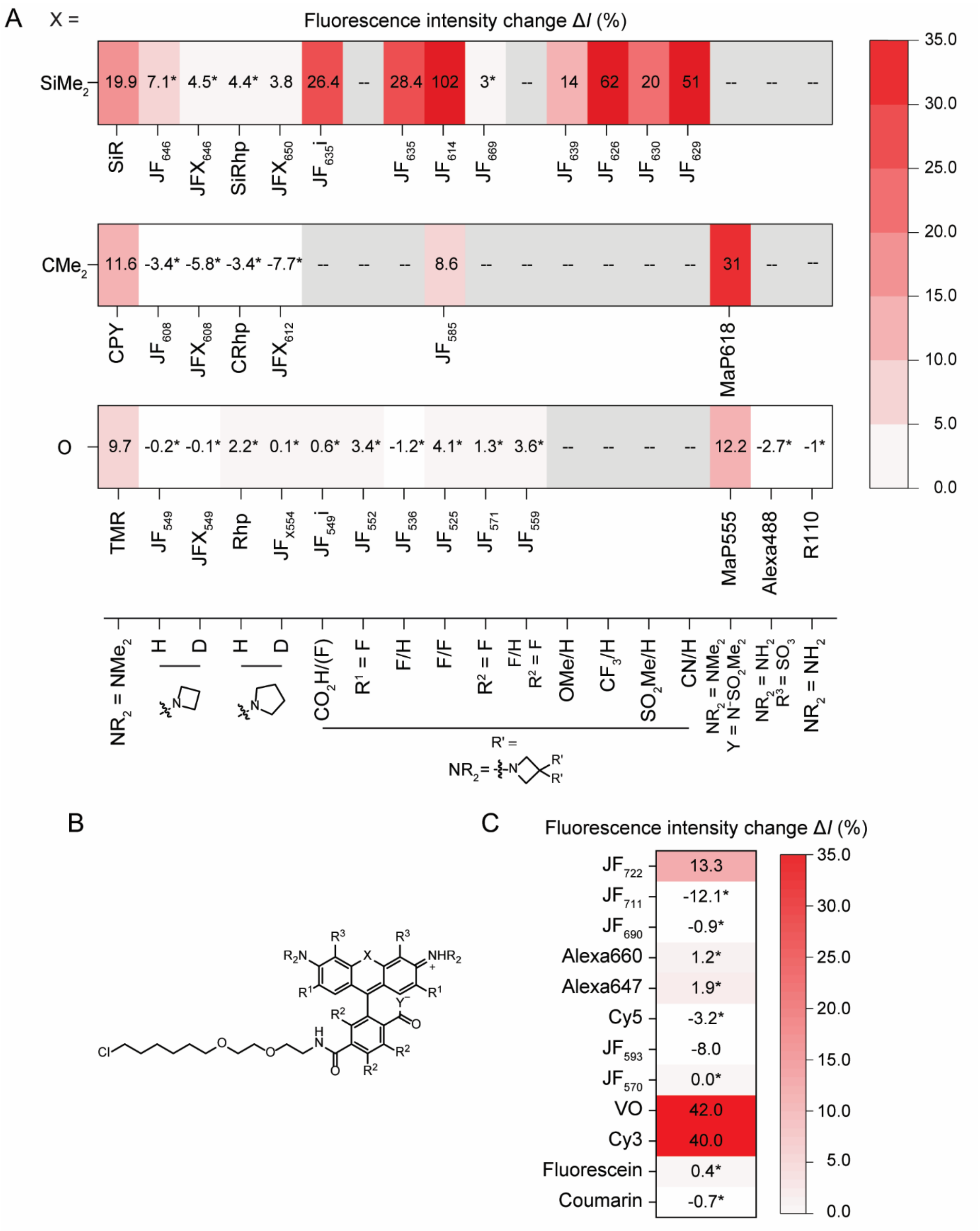
A
X =
Fluorescence intensity change ΔI (%)
SiMe2
19.9 7.1* 4.5* 4.4* 3.8 26.4 -- 28.4 102 3* -- 14 62 20 51 -- -- --
SiR JF646 JFX646 SiRhp JFX650 JF635i JF635 JF614 JF669 JF639 JF626 JF630 JF629
CMe2
11.6 -3.4* -5.8* -3.4* -7.7* -- -- -- 8.6 -- -- -- -- -- -- 31 -- --
CPY JF608 JFX608 CRhp JFX612 JF585 MaP618
O
9.7 -0.2* -0.1* 2.2* 0.1* 0.6* 3.4* -1.2* 4.1* 1.3* 3.6* -- -- -- -- 12.2 -2.7* -1*
TMR JF549 JFX549 Rhp JFX554 JF549i JF552 JF536 JF525 JF571 JF559 MaP555 Alexa488 R110
NR2 = NMe2
H D H D
CO2H/(F) R1 = F F/H F/F R2 = F F/H R2 = F OMe/H CF3/H SO2Me/H CN/H
NR2 = NMe2 Y = N-SO2Me2
NR2 = NH2 R3 = SO3
NR2 = NH2
R' =
NR2 =
35.0 30.0 25.0 20.0 15.0 10.0 5.0 0.0
B
C
Fluorescence intensity change ΔI (%)
JF722 13.3
JF711 -12.1*
JF690 -0.9*
Alexa660 1.2*
Alexa647 1.9*
Cy5 -3.2*
JF593 -8.0
JF570 0.0*
VO 42.0
Cy3 40.0
Fluorescein 0.4*
Coumarin -0.7*
35.0 30.0 25.0 20.0 15.0 10.0 5.0 0.0

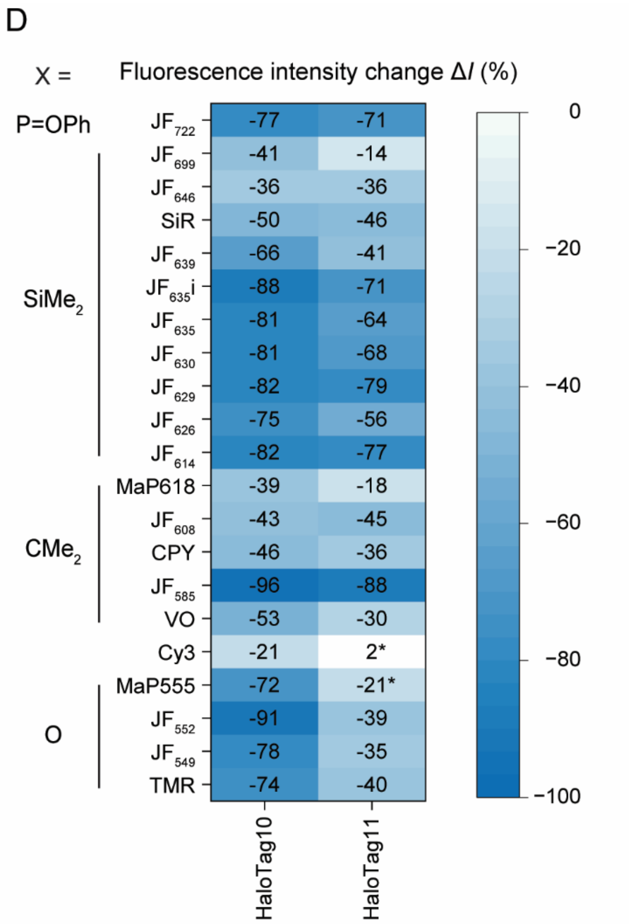

Supplementary Figure S3: Comparison of the fluorescence brightness of HaloTag variants and HaloTag7. **A** Fluorescence intensity change ($\Delta I = (I_{HT9} - I_{HT7}) \cdot I_{HT7}^{-1}$) of rhodamine based fluorophores reacted with HaloTag9 compared to HaloTag7. Fluorophore structures can be found in Supplementary Table S4 or can be inferred using the legend and the generic rhodamine structure in (**B**). Unless otherwise stated R^1, R^2, and R^3 = H, Y^- = O^-, and R' = H. **B** Chemical structure of generic rhodamine. **C** Fluorescence intensity changes for other fluorophores (e.g. X = S (JF_{570} and JF_{593}) or X = P=O(OH)/P=OPh (JF_{690}, JF_{711}, and JF_{722})) as well as fluoresceins (fluorescein and Virginia Orange (VO)), cyanines (Cyanine3, Cyanine5, and Alexa647), and coumarin when reacted with HaloTag9 in comparison to HaloTag7. Mean, N = 2 replicates each 4 samples. **D** Fluorescence intensity change ($\Delta I = (I_{HT\text{-}var} - I_{HT7}) \cdot I_{HT7}^{-1}$) of selected fluorophores reacted with HaloTag10 and HaloTag11 compared to HaloTag7. HaloTag10 is dimmer than HaloTag11. Mean, N = 4 samples. Fluorophore structures can be found in Supplementary Table S4. * Not significant. – Fluorophore not measured.

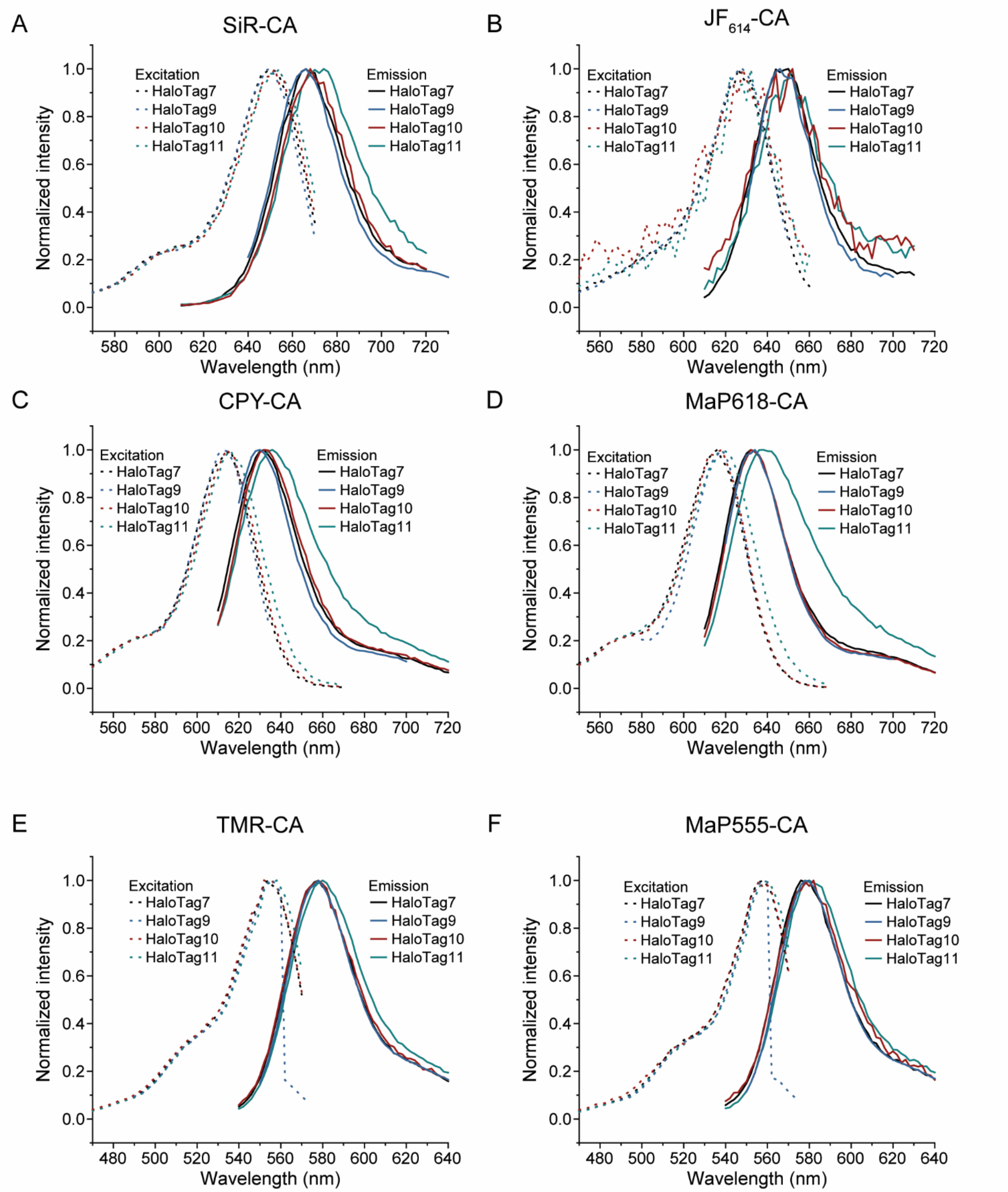

Supplementary Figure S4: Normalized fluorescence excitation and emission spectra of six fluorophores on HaloTags. **A-F** The spectra were measured for SiR-CA (**A**), JF_{614}-CA (**B**), CPY-CA (**C**), MaP618-CA (**D**), TMR-CA (**E**), and MaP555-CA (**F**) reacted to the different HaloTags. Almost no spectral changes were found for the fluorophores on the different tags only HaloTag11 showed a small bathochromic shift of 2-5 nm in emission and 1-3 nm in excitation.

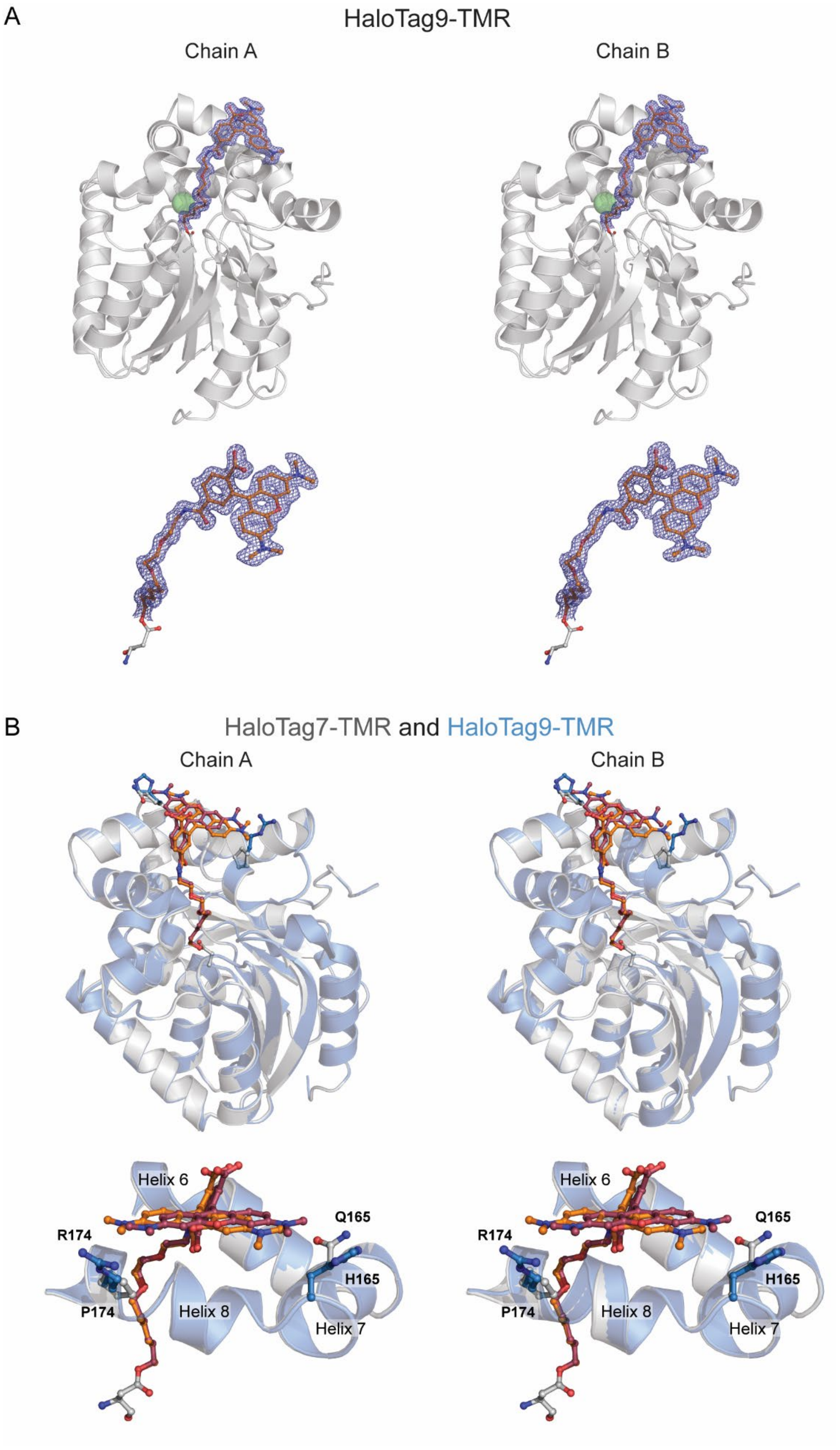
A
HaloTag9-TMR
Chain A
Chain B
B
HaloTag7-TMR and HaloTag9-TMR
Chain A
Chain B
Helix 6
Q165
R174
H165
P174
Helix 8
Helix 7
Helix 6
Q165
R174
H165
P174
Helix 8
Helix 7

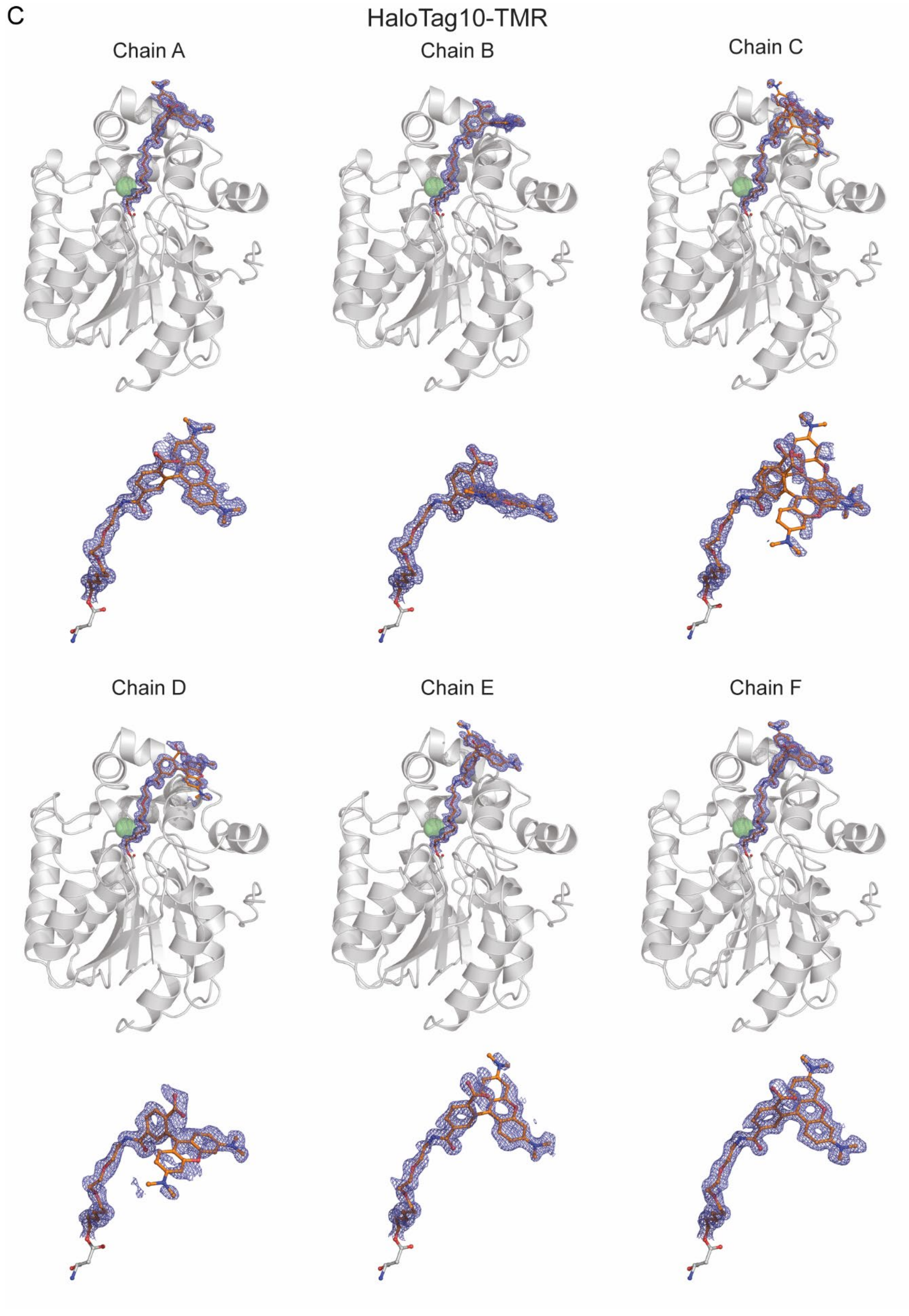
C
HaloTag10-TMR
Chain A
Chain B
Chain C
Chain D
Chain E
Chain F

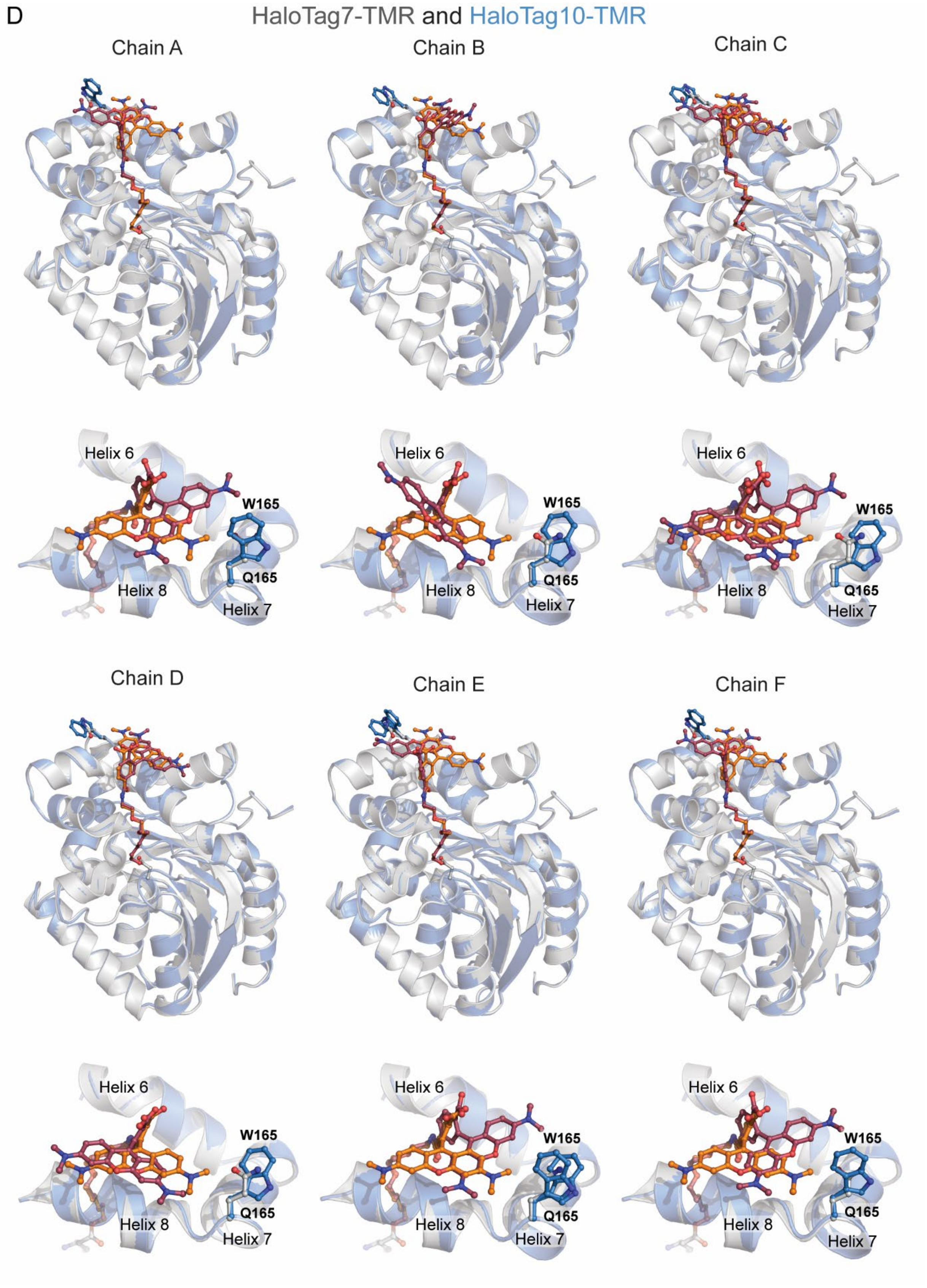
D
HaloTag7-TMR and HaloTag10-TMR
Chain A
Chain B
Chain C
Helix 6
W165
Q165
Helix 8
Helix 7
Chain D
Chain E
Chain F

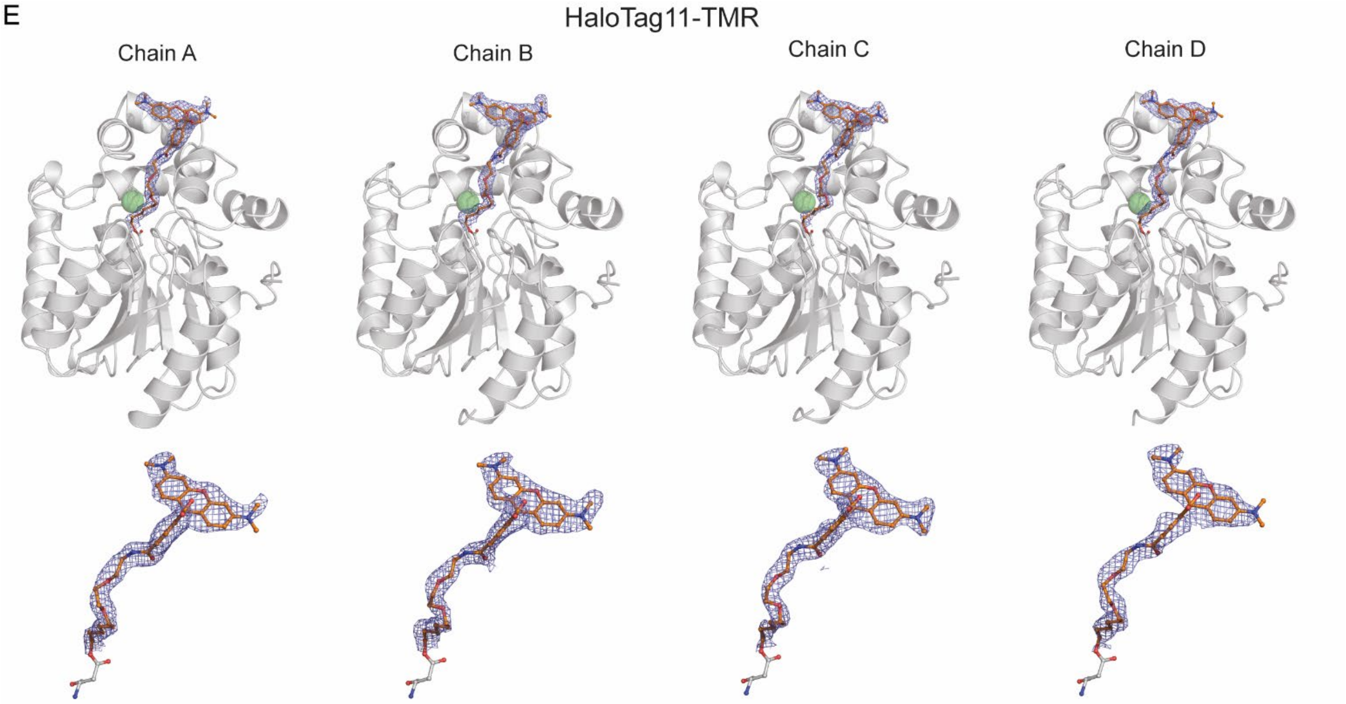

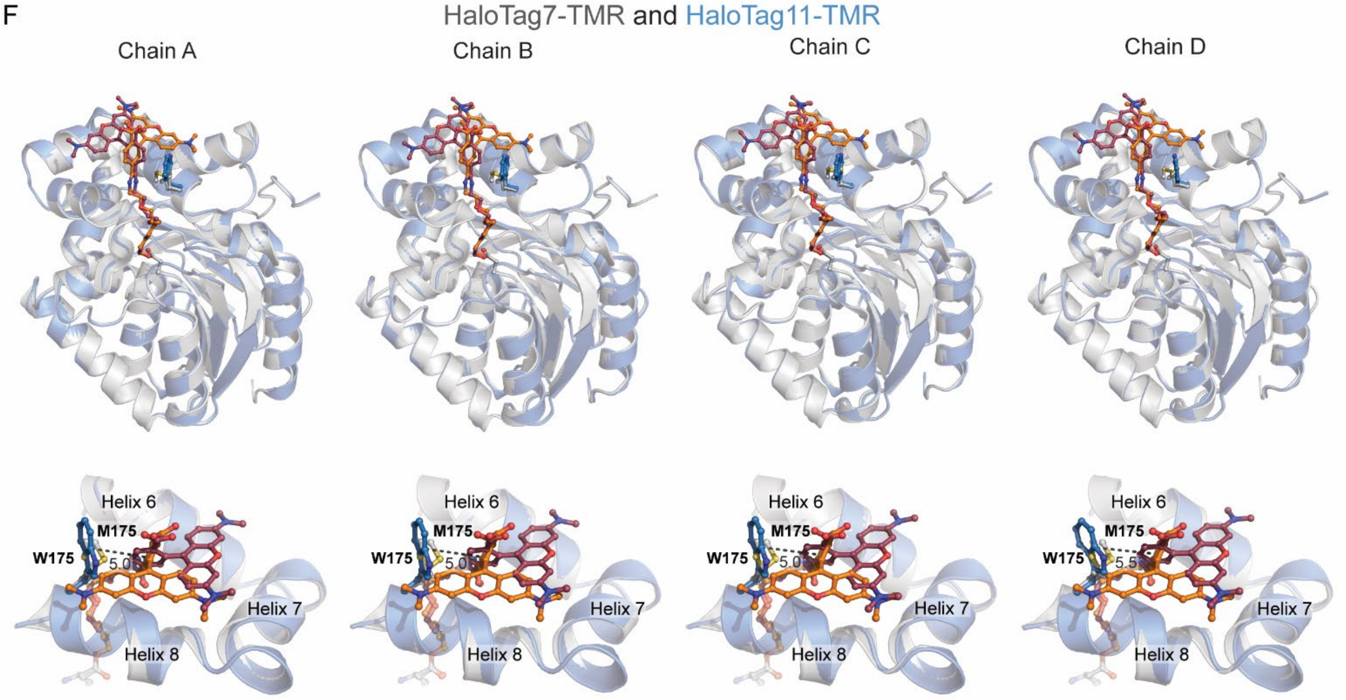

Supplementary Figure S5: Crystal structure of HaloTag9-TMR, HaloTag10-TMR, and HaloTag11-TMR. **A, C, E** OMIT-maps of TMR from the HaloTag9-TMR structure (PDB ID: 6ZVY, 1.4 Å, **A**), HaloTag10-TMR structure (PDB ID: 7PCX, 1.4 Å, **C**), HaloTag11-TMR structure (PDB ID: 7PCW, 2.3 Å, **E**). The protein of each chain is represented as a cartoon (grey) while TMR is represented as sticks (orange). The chlorine atom is represented as a green sphere. The composite OMIT electron-density map for TMR is contoured at 1.0 σ and represented as blue mesh (2mFo-DFc).

In addition, a magnification of the TMR binding site is shown. **B, D, F** Structural comparison of HaloTag9-TMR (PDB ID: 6ZVY, 1.4 Å, **B**), HaloTag10-TMR (PDB ID: 7PCX, 1.4 Å, **D**), HaloTag11-TMR (PDB ID: 7PCW, 2.3 Å, **F**), with HaloTag7-TMR (PDB ID: 6Y7A, 1.4 Å, only chain A). The proteins are given in cartoon representation: HaloTag7 in grey and HaloTag9, HaloTag10, or HaloTag11 in blue. The bound TMR is given in stick representation in orange (HaloTag7) and violet (HaloTag9, HaloTag10 or HaloTag11). In addition, a zoom onto helices 6–8 with the proteins in cartoon representation and the TMR ligand as well as the mutated amino-acids as sticks is given. Distances between the centers of aromatic rings are indicated in Ångström (grey dashes).

Overall, the new HaloTag variants show a similar protein structure as HaloTag7 ($RMSD_{C\alpha}$; Supplementary Table S12).

HaloTag9 structural analysis: The xanthene core of TMR on HaloTag9 is displaced toward α-helix 7 ($RMSD_{xanth}$ = 1.82±0.16 Å). This induces an increase in the rotational energy barriers around the aniline groups of TMR ($\varphi_{Ar\text{-}NMe2}$) and a rise in the dihedral angle ($\varphi_{Ar\text{-}Ar}$) between the xanthene and the benzene ring ($\varphi_{Ar\text{-}Ar\text{-}HT7}$ = 119.0°, $\varphi_{Ar\text{-}Ar\text{-}HT9}$ 122.32±0.21°, Supplementary Fig. S8, Supplementary Table S12). Both should be favorable for quantum yields as non-radiative decay pathways via rotational motion or photoinduced electron transfer (PET) are suppressed[2,3]. Overall also, other factors such as the change in electrostatic surface potential might contribute to the increase in brightness (Supplementary Fig. S9). The proposed mechanisms are in agreement with the observation that for azetidine-bearing rhodamines small effects on quantum yield were observed as non-radiative decay pathways via rotational motion of these fluorophores are already reduced [4].

HaloTag10 structural analysis: The structure of HaloTag10-TMR with six chains in the asymmetric unit revealed different conformations of both TMR and W165 in the different monomers. TMR was either placed over helix 8 (chain C and D) as in the HaloTag7 (PDB ID: 6Y7A) structure, over helix 8 (chain B) but tilted, or over helix 7 (chain A, C, E, F) as also found for HaloTag11 and in the HaloTag7-TMR (PDB ID: 6U32)[1] crystal structure. In none of the monomers were π-stacking interactions between W165 and TMR of the same monomer observed. However, analysis of the crystal packing (Supplementary Fig. S6C) revealed that W165 π-stacks with TMR of the neighboring monomer. It is therefore unclear if the conformations of TMR and W165 found in the crystal structure are representative for the protein in solution.

HaloTag11 structural analysis: The xanthene core of TMR on HaloTag11 is centered over α-helix 7 and not as found for HaloTag7 (PDB ID: 6Y7A) over G171 on helix 8 ($RMSD_{xanth}$ = 7.96±0.12 Å). The xanthene core is shifted to a higher extend over α-helix 7 than observed for the second conformation in the HaloTag7-TMR (PDB ID: 6U32)[1] crystal structure. Additionally, W175 forms π-stacking interactions with the benzene ring of TMR. The proximity between W175 and TMR could lead to PET quenching, rationalizing the reduced quantum yields[5].

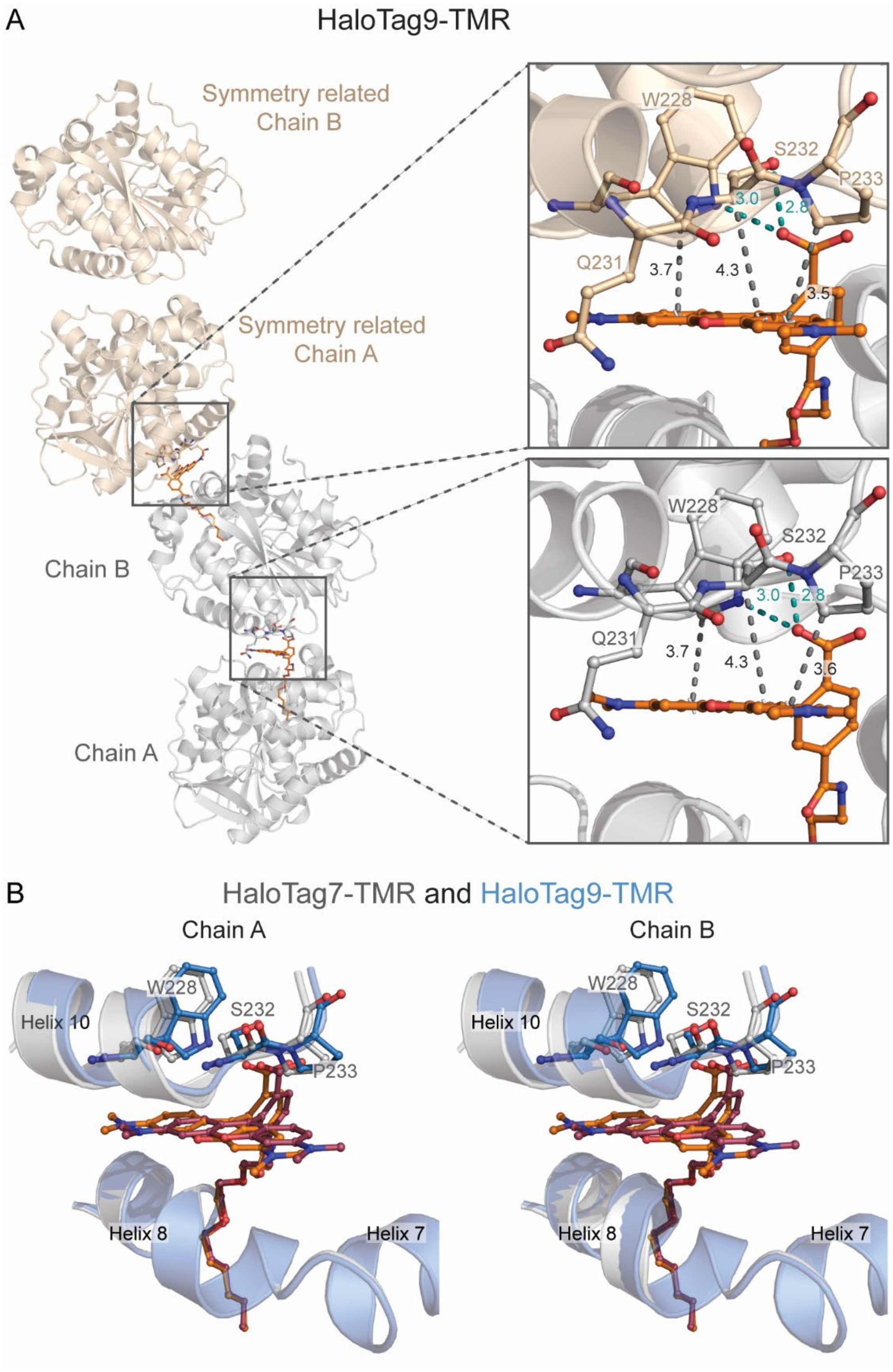
A
HaloTag9-TMR
Symmetry related
Chain B
Symmetry related
Chain A
Chain B
Chain A
W228
S232
P233
Q231
3.0
2.8
3.7
4.3
3.5
W228
S232
P233
Q231
3.0
2.8
3.7
4.3
3.6
B
HaloTag7-TMR and HaloTag9-TMR
Chain A
Chain B
W228
S232
Helix 10
P233
Helix 8
Helix 7
W228
S232
Helix 10
P233
Helix 8
Helix 7

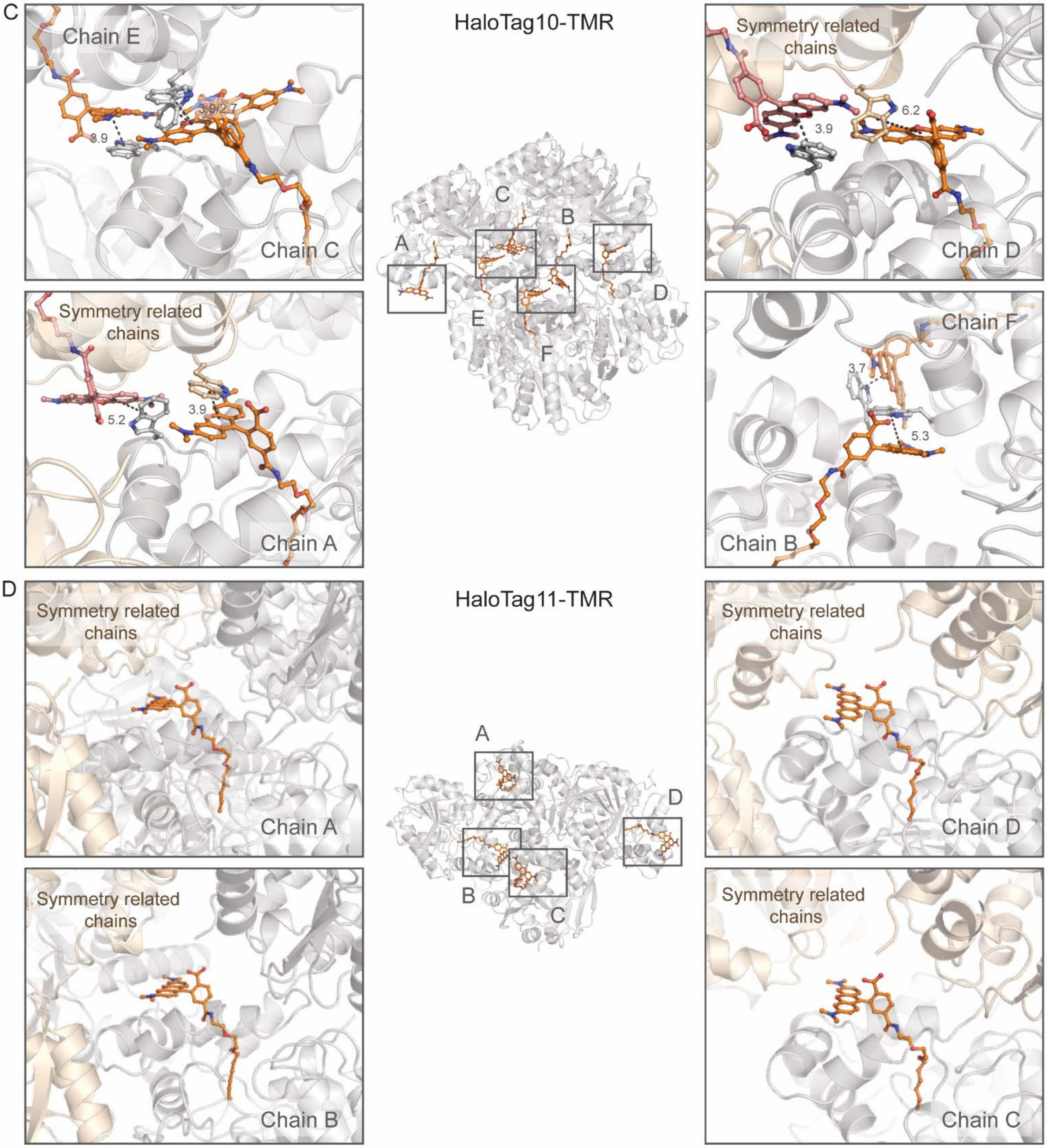

Supplementary Figure S6: Structural analysis of the interactions of TMR at the crystal packing interface. **A** HaloTag9-TMR (PDB ID: 6ZVY, 1.4 Å) crystal structure with the indicated zoom regions. The proteins are represented as cartoon (grey (asymmetric unit) or beige (symmetry related)). The bound TMR at the interface is represented as orange sticks. Important helix residues of the neighboring monomer are represented as sticks together with important distances, which are indicated in Ångström (e.g. hydrogen bonds: carboxylic acid of TMR with S232 and W228 (teal dashes); distances from the xanthene ring to W228 (Cδ), S232 (Cα), and P233 (Cδ) (grey

dashes)). **B** Structural comparison of both chains of HaloTag9-TMR (D, PDB ID: 6ZVY, 1.4 Å) with HaloTag7-TMR at the packing interface. The HaloTag7 and HaloTag9 proteins are represented as cartoons in grey and blue, respectively. The bound TMR is given in stick representation in orange (HaloTag7) and violet (HaloTag9). Helices 7–8 of one monomer are represented with the end of helix 10 (224–234) of the adjacent monomer. Amino acids in close proximity to TMR are represented as sticks. **C-D** HaloTag10-TMR (PDB ID: 7PCX, 1.4 Å, **C**) or HaloTag11-TMR (PDB ID: 7PCW, 2.3 Å, **D**) crystal structure with the indicated zoom regions. The proteins are represented as cartoon (grey (asymmetric unit) or beige (symmetry related)). The bound TMR at the interface is represented as orange/pink sticks (asymmetric unit/symmetry related). Important helix residues of the neighboring protein are represented as sticks together with characteristic distances between aromatic rings, which are indicated in Ångström (grey dashes).

HaloTag9 structural analysis (A and B): Comparing the distances of the hydrogen bonds of the carboxylic acid in the HaloTag9 structure with those in the structure of HaloTag7 reveals that the distances do not change (Supplementary Table S13). The van der Waals distances increase for W228 (Cδ), remain constant for S232 (Cα), and decrease for P233 (Cδ) and the xanthene plane. This demonstrates that the fluorophore shifts from W288 towards S232 albeit the general interface remaining relatively similar. The shift of the xanthene core of the fluorophore toward helix 7 is accompanied by a shift of the neighboring protein as indicated by the $RMSD_{xanth}$ compared to the $RMSD_{pack}$ of the W228, S232, and P233 residues (Supplementary Table 12; due to the two possible confirmations of Q231 in the HaloTag7 crystal structure this residue was omitted for RMSD calculations). Overall, the crystal packing of HaloTag7 and HaloTag9 is similar allowing the comparison of the relative fluorophore orientation.

HaloTag10 structural analysis: HaloTag10-TMR adopts a different crystal packing than HaloTag7-TMR or HaloTag9-TMR. At the interface of different monomers HaloTag10-TMR shows π-stacking interactions of TMR with the neighboring W165.

HaloTag10 structural analysis: HaloTag11-TMR also adopts a different crystal packing than HaloTag7-TMR or HaloTag9-TMR. However, in the individual monomers of HaloTag11-TMR TMR do not come in close contact with any amino acids of the neighboring monomers (distance COO^- – G3 > 4.5 Å).

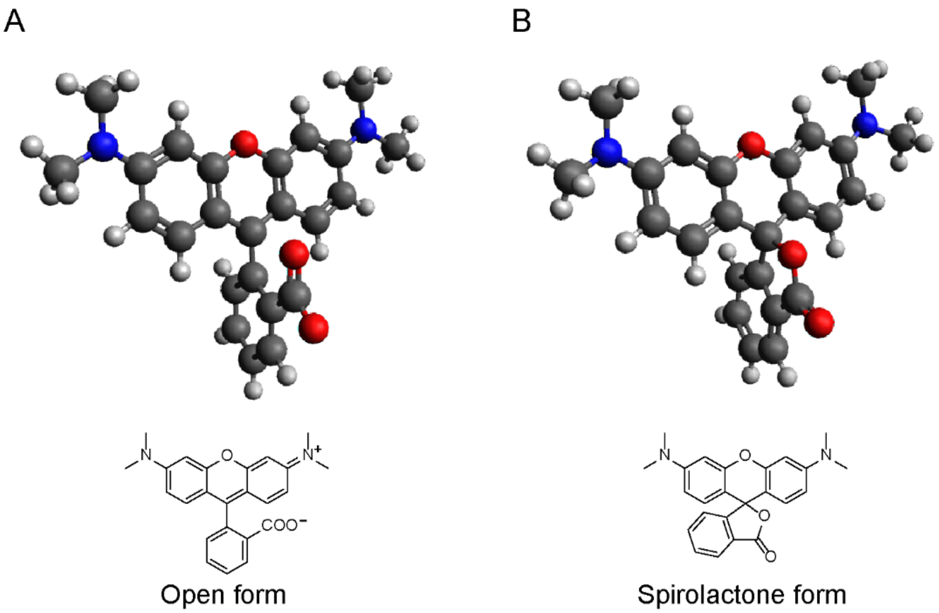

Supplementary Figure S7: Modeled structures of the open (**A**) and the spirolactone form (**B**) of TMR in water together with their chemical structures. The calculated angels $\varphi_{\text{Ar-Ar}}$ and γ correspond to $\varphi_{\text{Ar-Ar}}$ = 94.3° and γ = 5.6° for the open form and $\varphi_{\text{Ar-Ar}}$ = 115.1° and γ = 37.8° for the closed form.

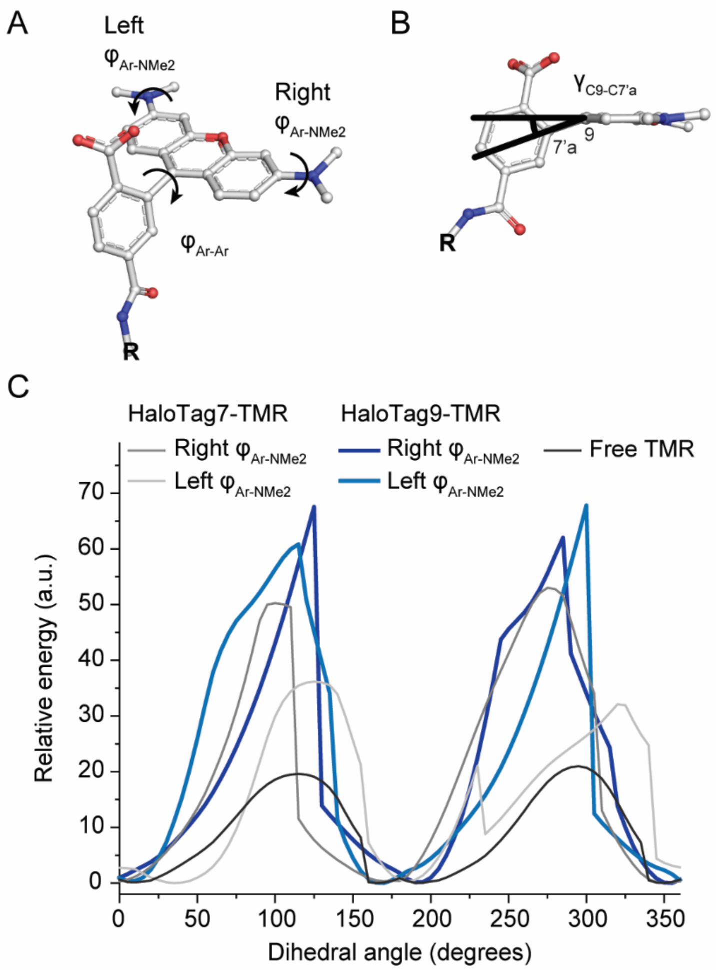

Supplementary Figure S8: Structural analysis of HaloTag7- or HaloTag9-TMR. **A** Dihedral angles $\varphi_{\text{Ar-Ar}}$ and $\varphi_{\text{Ar-NMe2}}$ left and right of TMR. The dihedral angle $\varphi_{\text{Ar-Ar}}$ is influenced by the protein, as it positions the xanthene core with respect to the chloroalkane, which is hold in place by two hydrogen bonds formed between the amid bond and T148 and T172. **B** Angle γ by which the C9-C7'a bond is tilted out of the xanthene plane. TMR on HaloTag9 and HaloTag7 showed angles of γ = 4.1° and 8.7°, respectively, which is in accordance with the calculated structures of the open form of TMR (5.6°) in water (Supplementary Fig. S7). A smaller angle in HaloTag9-TMR might indicate a greater sp^2 character of C9 and therefore a higher contribution of its remaining p orbital to the molecular orbitals relevant to the chromophore. This could positively influence the intrinsic extinction coefficient. **C** Relative energies of TMR when rotating the aniline group around $\varphi_{\text{Ar-NMe2}}$ (right or left). The rotational barriers are higher for TMR bound to HaloTag9 than those for TMR bound to Halotag7 or for TMR free in solution. Energies given were calculated by molecular modeling using the force field OPLS3e.

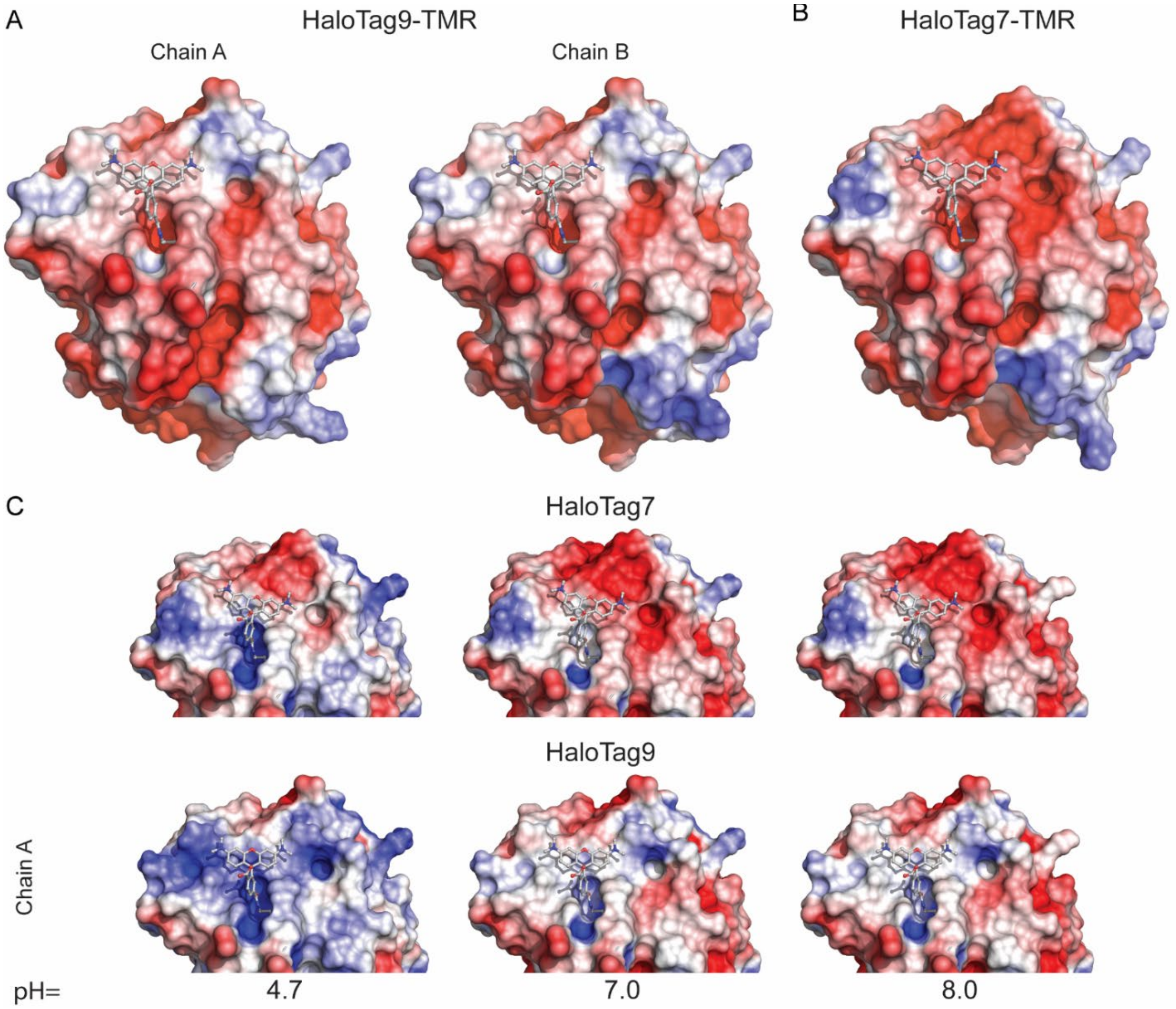

Supplementary Figure S9: Electrostatic potential of HaloTag9-TMR and HaloTag7-TMR mapped unto to their solvent excluded surface (Connolly surface). **A-B** The electrostatic surface potential of HaloTag9-TMR (PDB ID: 6ZVY, 1.4 Å, **A**) and HaloTag7-TMR (PDB ID: 6Y7A, 1.4 Å, **B**). The two mutations of HaloTag9 (Q165H-P174R) influence the local electrostatic potential around the fluorophore, going from predominantly negative on HaloTag7 to slightly positive on HaloTag9. Such a change in the rhodamine's solvation shell might stabilize its carboxylate group, favoring the open form and potentially increasing the extinction coefficient. **C** The electrostatic surface potential of HaloTag7-TMR and HaloTag9-TMR over the physiological pH range (4.7 to 8.0 (lysosomes – mitochondrial matrix))[6]. The surface potential of HaloTag7 close to the fluorophore binding site (excluding the substrate entry channel) changes marginally over pH and never reaches the positive charge observed for HaloTag9 at pH = 7.0. The surface potential of HaloTag9 is more pH sensitive, especially between pH 7.0 and 4.7, where it becomes very positively charged. The potentials were obtained using the adaptive Poisson-Boltzmann Solver (APBS software) with standard parameters (0.15 M ionic strength in monovalent salt, 310.0 K, protein dielectric of 2, and solvent dielectric of 78.0). The surface potentials mapped unto the solvent excluded surface are on a [−3; 3] red–white–blue color map in units of kJ · mol^{-1} · e^{-1}. Calculations were performed using the APBS & PDB2PQR plug-in in Pymol[7] or the PDB2PQR web service[8].

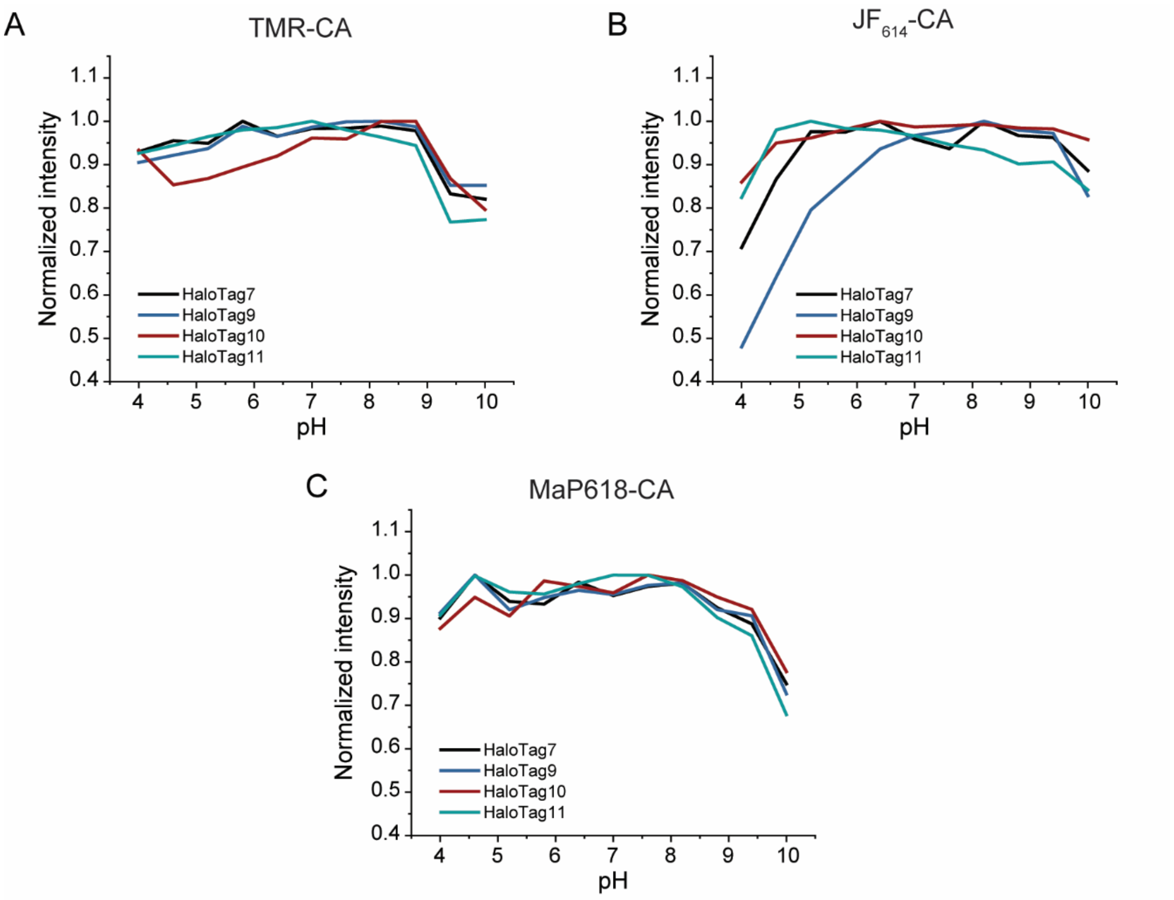

Supplementary Figure S10: Normalized fluorescence intensity of three fluorophores on the HaloTags at different pH. **A-C** Fluorescence intensity was measured for TMR-CA (**A**, non-fluorogenic), JF_{614}-CA (**B**, fluorogenic), and MaP618-CA (**C**, fluorogenic) bound to HaloTag variants or HaloTag7. pH was varied between 4.0 and 10.0 covering the physiological range from 4.7-8.0 (mean; N = 2 measurements).

For all HaloTag variants and HaloTag7 the fluorescence intensity was stable over the physiological range. For HaloTag7 this is in agreement with the rather small changes in electrostatic surface potential in close proximity to the fluorophore binding site over the same pH range (Supplementary Fig. S9). The fluorescence intensity started to drop at higher pH, which is in line with previous reports showing that under basic conditions the open-closed equilibrium is shifted towards the closed form[9]. However, it is difficult to distinguish whether this is due to the attack of the intramolecular nucleophile or an OH^- nucleophile. For JF_{614}-CA an additional drop in intensity is observed below pH 5.5, which most likely corresponds to the protonation of the aniline nitrogen as previously reported[10]. This is more pronounced for JF_{614}-CA than for MaP618-CA and TMR-CA as the two fluorine substituents on each azetidine render the aniline nitrogen of JF_{614}-CA more basic than the one of rhodamines, which do not bear electron withdrawing groups at this position (e.g. TMR-CA or MaP618-CA).

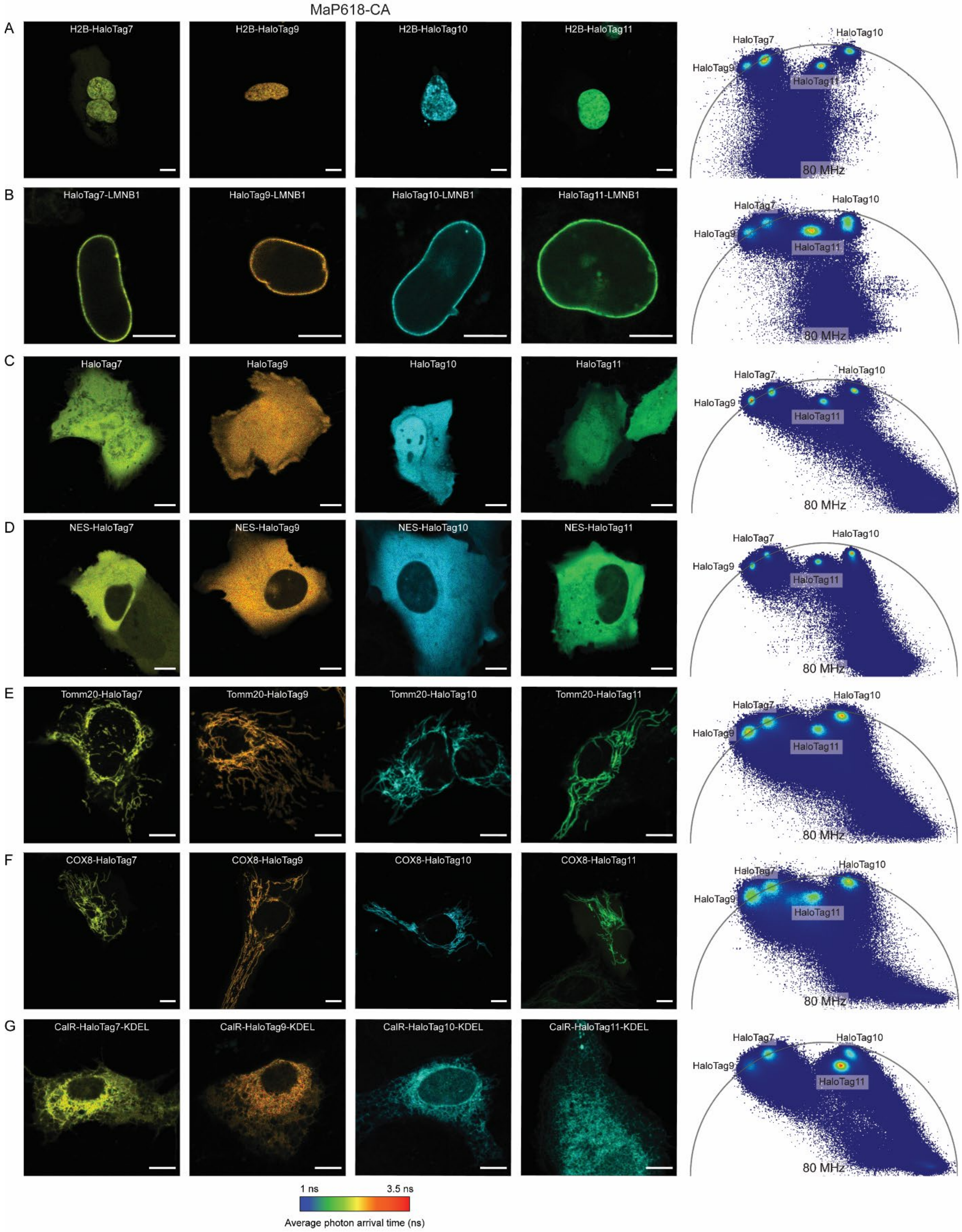
MaP618-CA
A
H2B-HaloTag7
H2B-HaloTag9
H2B-HaloTag10
H2B-HaloTag11
B
HaloTag7-LMNB1
HaloTag9-LMNB1
HaloTag10-LMNB1
HaloTag11-LMNB1
C
HaloTag7
HaloTag9
HaloTag10
HaloTag11
D
NES-HaloTag7
NES-HaloTag9
NES-HaloTag10
NES-HaloTag11
E
Tomm20-HaloTag7
Tomm20-HaloTag9
Tomm20-HaloTag10
Tomm20-HaloTag11
F
COX8-HaloTag7
COX8-HaloTag9
COX8-HaloTag10
COX8-HaloTag11
G
CalR-HaloTag7-KDEL
CalR-HaloTag9-KDEL
CalR-HaloTag10-KDEL
CalR-HaloTag11-KDEL
80 MHz
1 ns
3.5 ns
Average photon arrival time (ns)

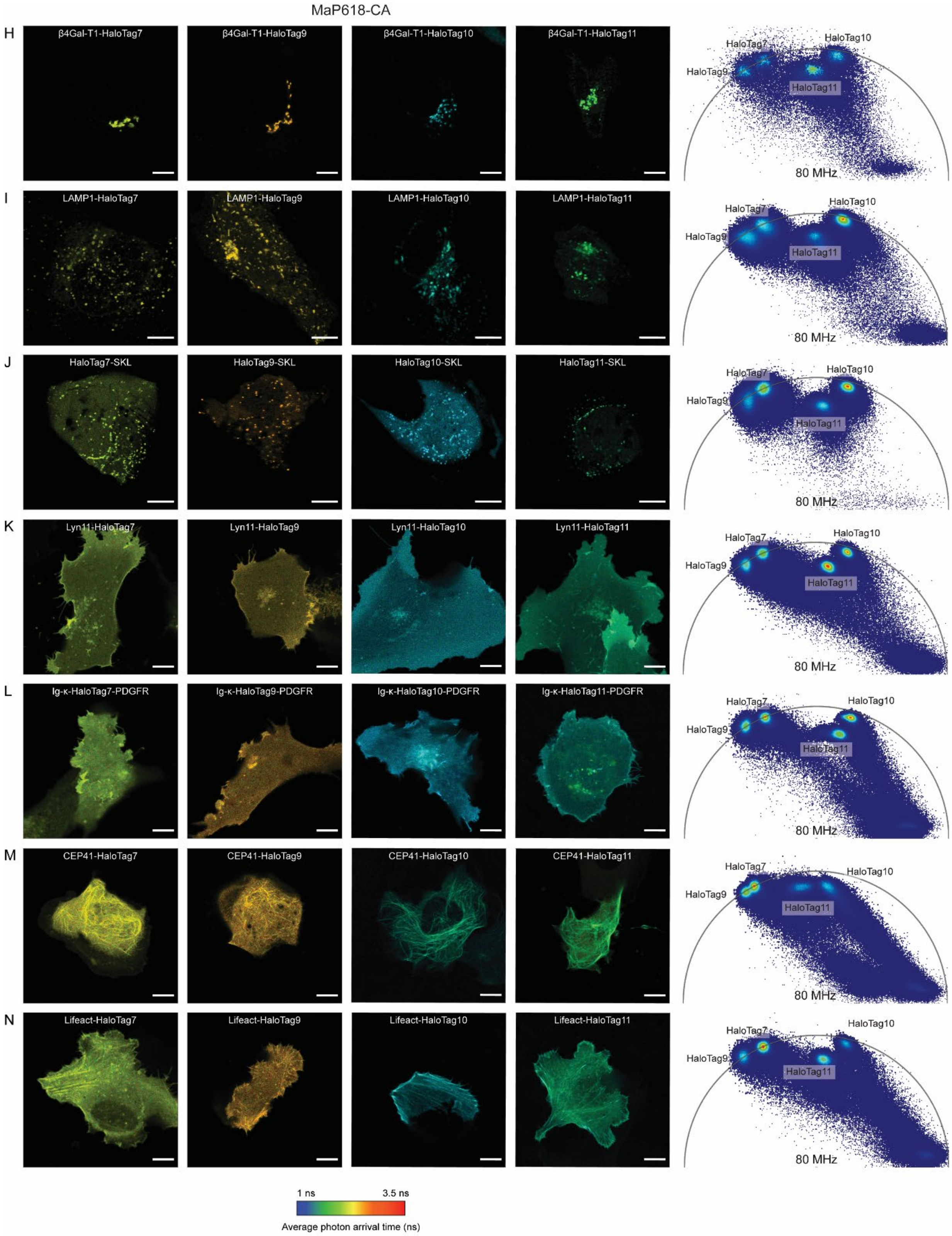

Supplementary Figure S11: Live-cell fluorescence lifetime microscopy of HaloTag7, HaloTag9, HaloTag10, or HaloTag11 fusion proteins labeled with MaP618-CA. FastFLIM image for each fusion protein as well as the phasor

plot of all four are given. **A** Nucleus, labeling histone 2B (H2B). **B** Nuclear lamina, labeling Lamin B1 (LMNB1). **C** Untargeted (no fusion). **D** Cytosol, fusion with a nuclear export signal (NES). **E** Outer mitochondrial membrane, labeling the membrane receptor Tomm20. **F** Inner mitochondrial membrane, labeling the cytochrome c oxidase subunit 8 (COX8). **G** Endoplasmic reticulum, labeling calreticulin (CalR) and additionally fused to a KDEL targeting peptide. **H** Golgi Apparatus, labeling beta-1,4-galactosidase (β-4-Gal-T1). **I** Lysosomes, labeling lysosome-associated membrane glycoprotein 1 (LAMP1). **J** Peroxisomes, targeted via a peroxisomal targeting signal (SKL). **K** Inner leaflet of the plasma membrane, labeling tyrosine protein kinase Lyn11. **L** Outer plasma membrane, labeling platelet-derived growth factor receptor (PDGFR) and additionally fused to an immunoglobulin kappa light chain (Ig-κ) leader sequence. **M** Microtubules, labeling the microtubule-binding protein CEP41. **N** Filamentous actin (F-actin), labeling the actin-binding peptide lifeact. Representative images from two independent experiments. Scale bars, 10 µm.

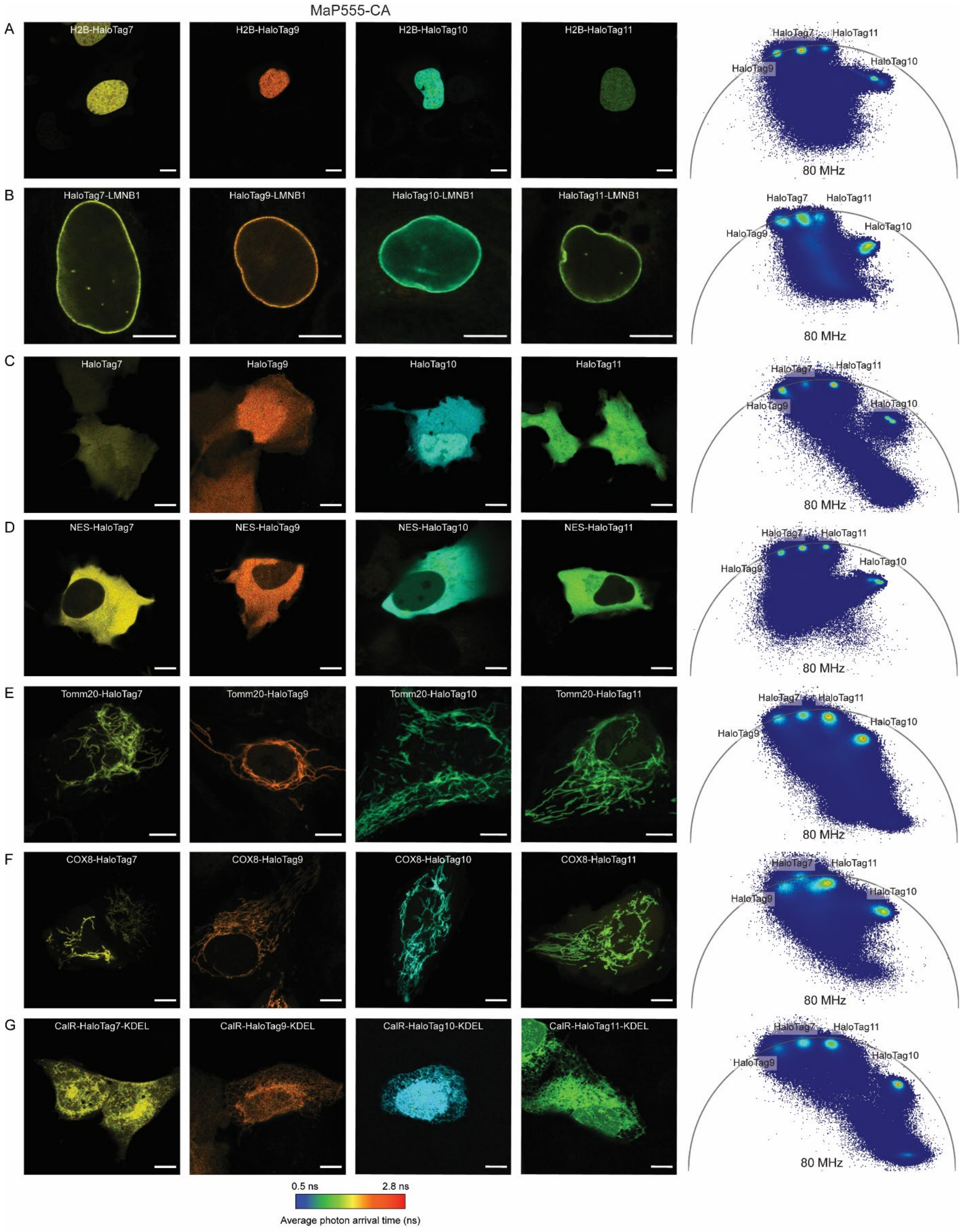
MaP555-CA
A
H2B-HaloTag7
H2B-HaloTag9
H2B-HaloTag10
H2B-HaloTag11
B
HaloTag7-LMNB1
HaloTag9-LMNB1
HaloTag10-LMNB1
HaloTag11-LMNB1
C
HaloTag7
HaloTag9
HaloTag10
HaloTag11
D
NES-HaloTag7
NES-HaloTag9
NES-HaloTag10
NES-HaloTag11
E
Tomm20-HaloTag7
Tomm20-HaloTag9
Tomm20-HaloTag10
Tomm20-HaloTag11
F
COX8-HaloTag7
COX8-HaloTag9
COX8-HaloTag10
COX8-HaloTag11
G
CalR-HaloTag7-KDEL
CalR-HaloTag9-KDEL
CalR-HaloTag10-KDEL
CalR-HaloTag11-KDEL
80 MHz
0.5 ns
2.8 ns
Average photon arrival time (ns)

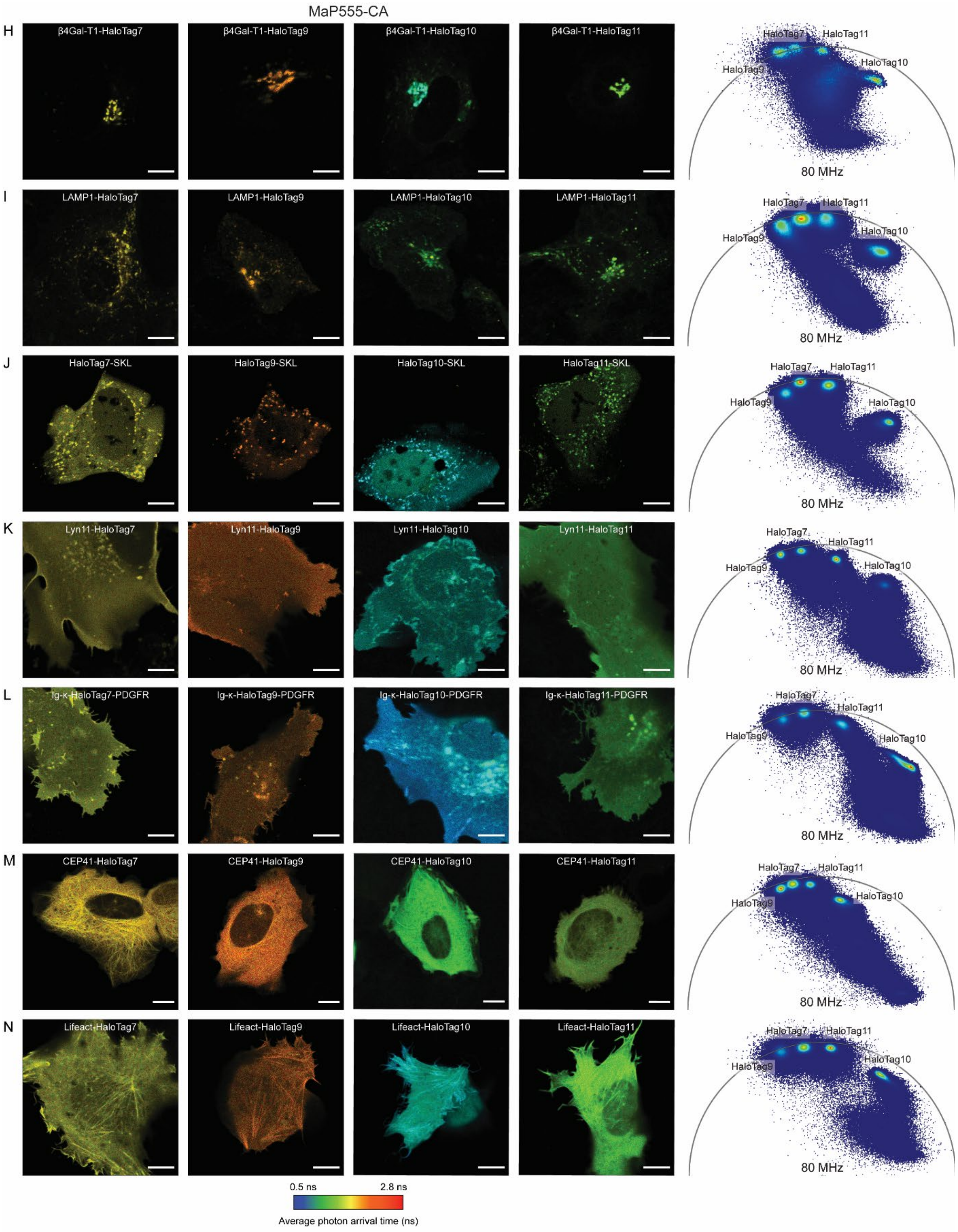

Supplementary Figure S12: Live-cell fluorescence lifetime microscopy of HaloTag7, HaloTag9, HaloTag10, or HaloTag11 fusion proteins labeled with MaP555-CA. FastFLIM image for each protein as well as the phasor plot of all four are given. **A** Nucleus, labeling H2B. **B** Nuclear lamina, labeling LMNB1. **C** Untargeted (no fusion). **D** Cytosol,

fusion with a NES. **E** Outer mitochondrial membrane, labeling Tomm20. **F** Inner mitochondrial membrane, labeling COX8. **G** Endoplasmic reticulum, labeling CalR and additionally fused to KDEL. **H** Golgi Apparatus, labeling β-4-Gal-T1. **I** Lysosomes, labeling LAMP1. **J** Peroxisomes, targeted via SKL. **K** Inner leaflet of the plasma membrane, labeling Lyn11. **L** Outer plasma membrane, labeling PDGFR and additionally fused to Ig-κ. **M** Microtubules, labeling CEP41. **N** F-actin, labeling lifeact. Representative images from two independent experiments. Scale bars, 10 µm.

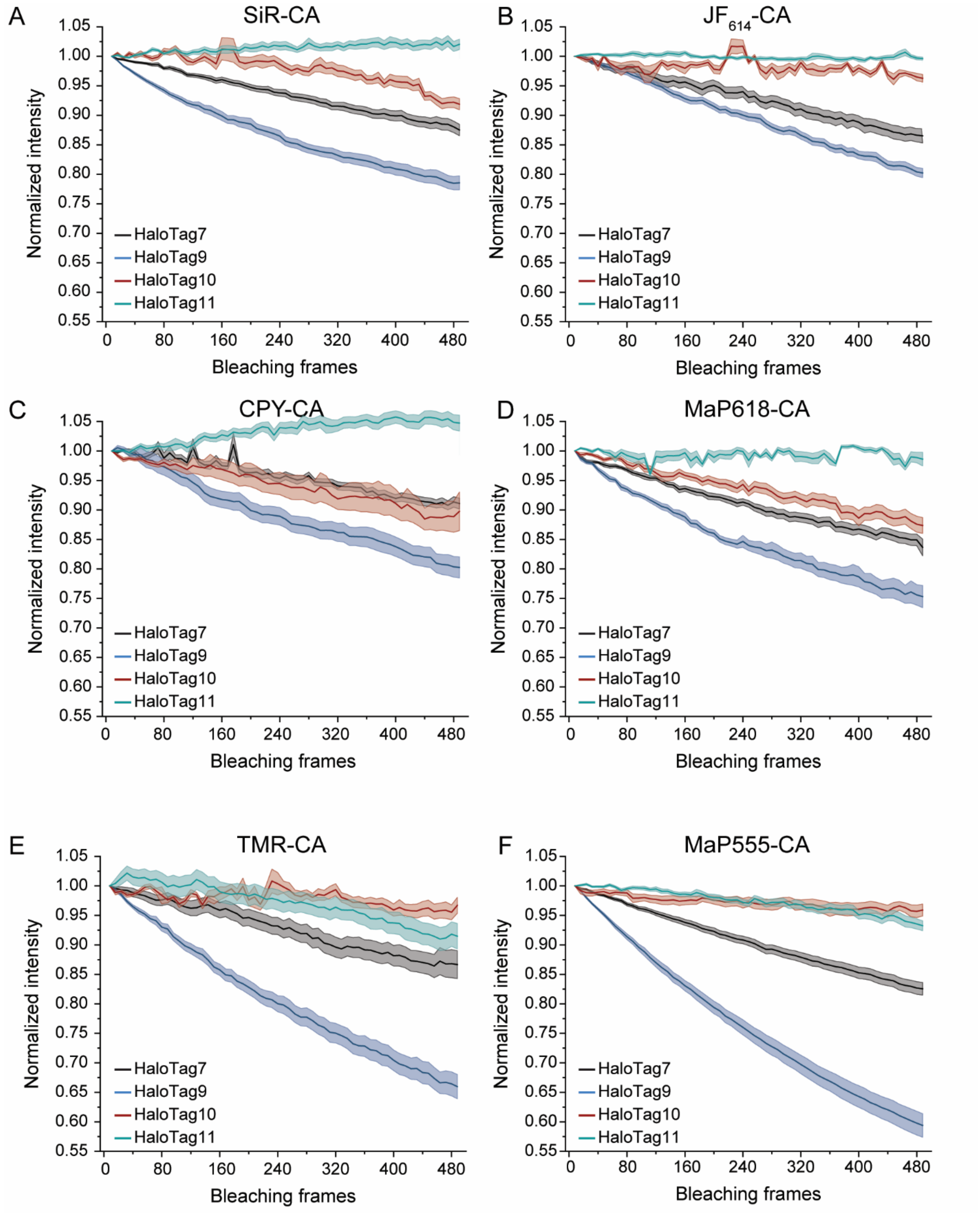

Supplementary Figure S13: Photostability of different fluorophores on the HaloTags assessed by confocal microscopy. **A-F** Plots of normalized fluorescence intensity over time from living U-2 OS cells. U-2 OS cells stably expressing H2B-HaloTag7, H2B-HaloTag9, H2B-HaloTag10, or H2B-HaloTag11 were labeled with SiR-CA (**A**), JF_{614}-CA (**B**), CPY-CA (**C**), MaP618-CA (**D**), TMR-CA (**E**), and MaP555-CA (**F**) (1 μM, 3 h) and imaged by confocal microscopy

over 480 bleaching cycles. Photobleaching was induced by irradiation at maximal laser power and a z-stack to read out the fluorescence intensity was acquired every 8 bleaching cycles (line and shaded area = mean±95% confidence interval, for *N* see Supplementary Table S17). The difference in relative fluorescence intensity in percentage after 480 photobleaching frames are given in Supplementary Table S17.

In all experiments performed, HaloTag9 was less photostable than HaloTag7, and HaloTag10 and HaloTag11 more photostable than HaloTag7. Moreover, photobleaching of the tested rhodamines on HaloTag variants correlates with their quantum yield and fluorescence lifetime: The higher the quantum yield or the fluorescence lifetime the faster the photobleaching. Photobleaching is initiated from the first excited state and can either proceed via the triplet state accessed by intersystem crossing or via higher excited states accessed by two-step excitation. Triplet state bleaching is expected to decrease as quantum yields increase, as a higher quantum yield indicates less non-radiative decay (including intersystem crossing) compared to fluorescence decay. Bleaching via higher excited states on the other hand is expected to increase as fluorescence lifetimes increase, as there is a higher probability of the fluorophore to undergo two-step excitation[11]. Our experiments therefore indicate that bleaching does not primarily proceed via the triplet state but rather via higher excited states.

The relative photostability of labeled HaloTag10 and Halotag11 depends on the nature of the fluorophore: For TMR-CA and MaP555-CA, HaloTag10 is more photostable than HaloTag11, whereas for the SiR and CPY based fluorophores (SiR-CA, JF_{614}-CA, CPY-CA, and MaP618-CA) HaloTag11 is more photostable than HaloTag10.

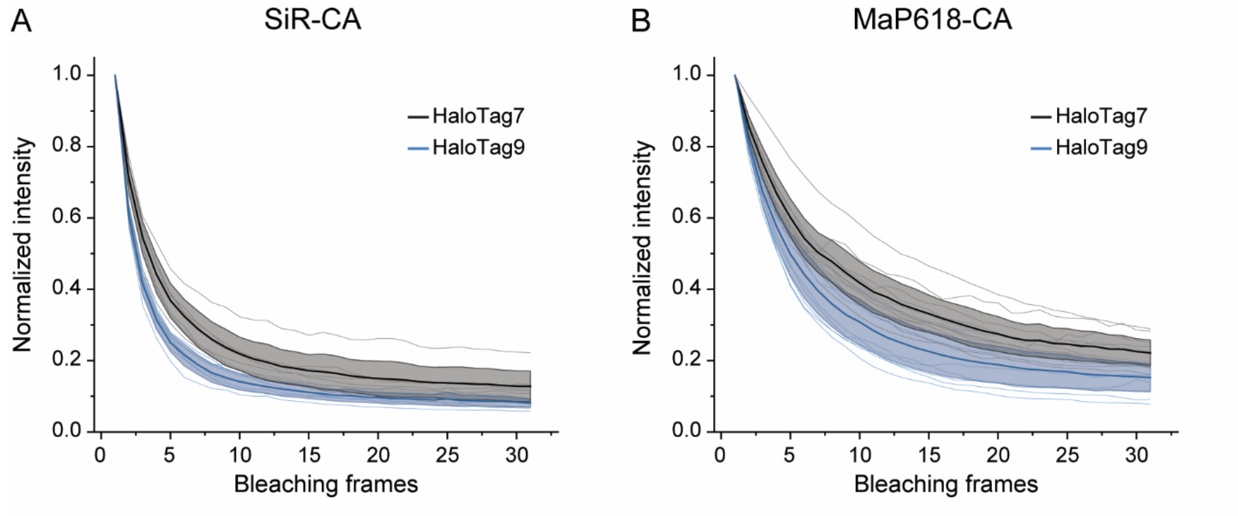

Supplementary Figure S14: Photostability of different fluorophores on HaloTag7 and HaloTag9 assessed by STED microscopy. **A-B** Plots of normalized fluorescence intensity over time from living U-2 OS cells. U-2 OS cells expressing Tomm20-HaloTag7 or Tomm20-HaloTag9 were labeled with SiR-CA (**A**), MaP618-CA (**B**) (1 µM, 3 h) and imaged by STED microscopy for 30 consecutive frames (thin lines = individual measurements; thick line and shaded area = mean±95% confidence interval, *N* = 7 (SiR-CA) or 9 (MaP618-CA) measurements from three independent preparations). Fluorescence intensity is rapidly dropping and whereas there is a small difference between HaloTag7 and HaloTag9 labeled with SiR-CA during the first 10 frames there is no difference for the later 20 frames or for the experiment using MaP618-CA. We therefore conclude that the small difference in photostability of HaloTag7 and HaloTag9 does not impact the usability of HaloTag9 for repeated STED microscopy. Both HaloTag7 and HaloTag9 can be used to acquire multiple (> 2) STED images.

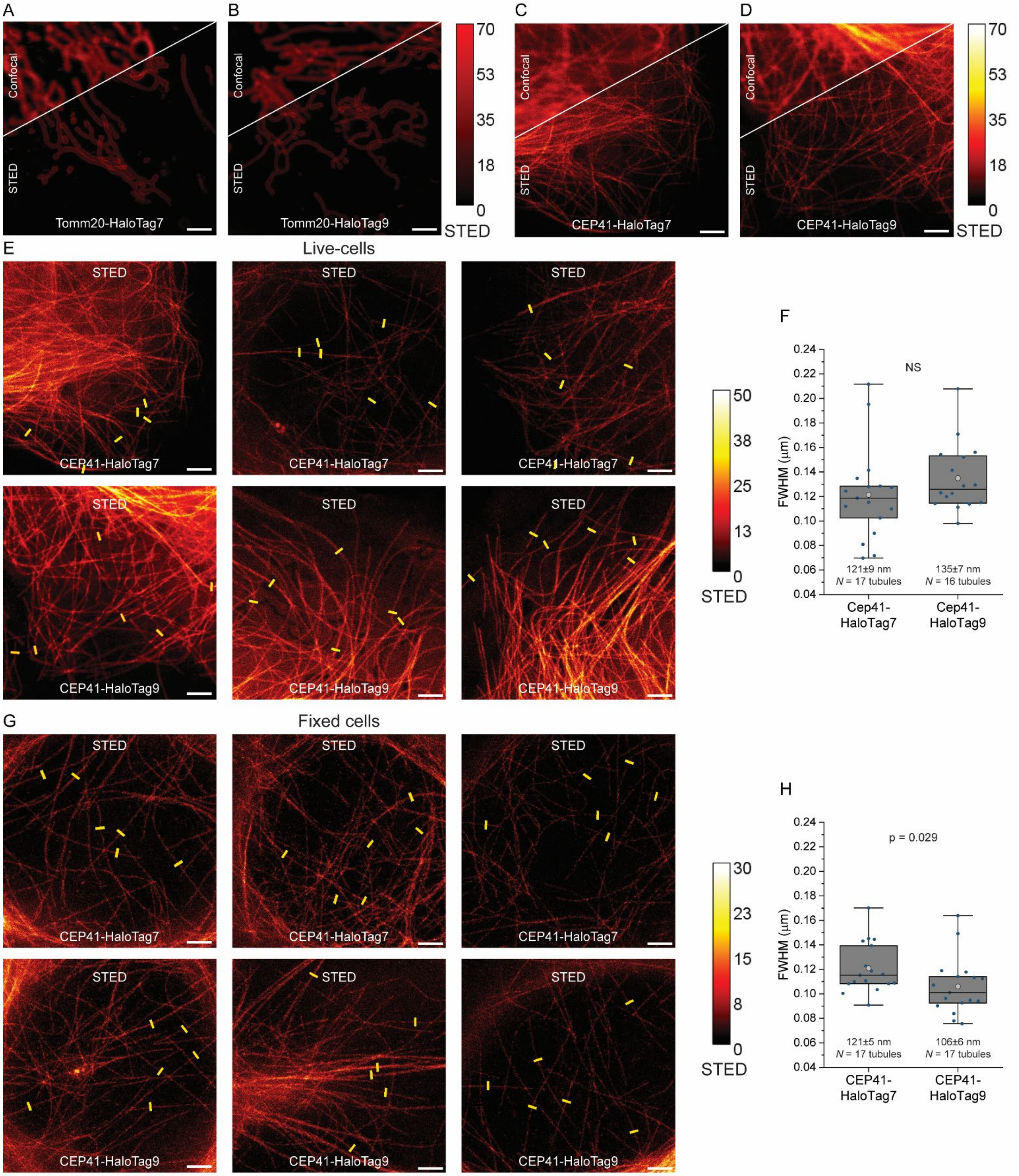

Supplementary Figure S15: Confocal and STED fluorescence microscopy images of mitochondria or microtubules labeled via HaloTag7 or HaloTag9. **A-B** U-2 OS cells stably expressing the outer mitochondrial membrane protein Tomm20 as a fusion with HaloTag7 (**A**) or HaloTag9 (**B**) labeled with MaP618-CA (1 µM, 3 h). **C-D** U-2 OS cells stably expressing the microtubule marker CEP41 as a fusion of HaloTag7 (**C**) or HaloTag9 (**D**) labeled with MaP618-CA

(1 µM, 3 h). **E**-**H** Microtubule diameter quantification in living (**E**-**F**) and fixed (**G**-**H**) U-2 OS cells. Full-width half maximum (FWHM) was measured at the indicated regions (**E** and **G**). In living cells no difference in FWHM between CEP41-HaloTag7 and CEP41-HaloTag9 labeled cells was found (HaloTag7: 121±9 nm vs. HaloTag9: 135±7 nm (mean±s.e.m.; *N* = 17 or 16 microtubules from 3 field of views from 2 preparations)). CEP41 is a microtubule-binding protein and most of the expressed protein localizes to microtubules but some can also be found in the cytosol, contributing to noise. Going from HaloTag7 to HaloTag9 does therefore not only increase the signal from microtubules but also the noise from the cytosol, effectively leaving the signal-to-noise ratio (SNR) unchanged. As the measured width of the microtubules depends on SNR and not the signal alone, no difference in FWHM is expected in living cells. However, in fixed cells, the cytosolic pool can be extracted during fixation, leading to an increase in SNR and therefore a decrease in microtubule width (**H**, HaloTag7: 121±5 nm vs. HaloTag9: 106±6 nm (mean±s.e.m.; *N* = 17 microtubules from 3 field of views from 2 preparations)). p-Value is given based on a one-sided t-test (α = 5%, r = −0.05/0.04, DF = 31/32; live/fixed). Box = 25%–75% percentile, whiskers = 5%–95% percentile, line = median, circle = mean. Representative images from three (**A-B**) or two (**C-G**) independent experiments. Scale bars, 2 µm.

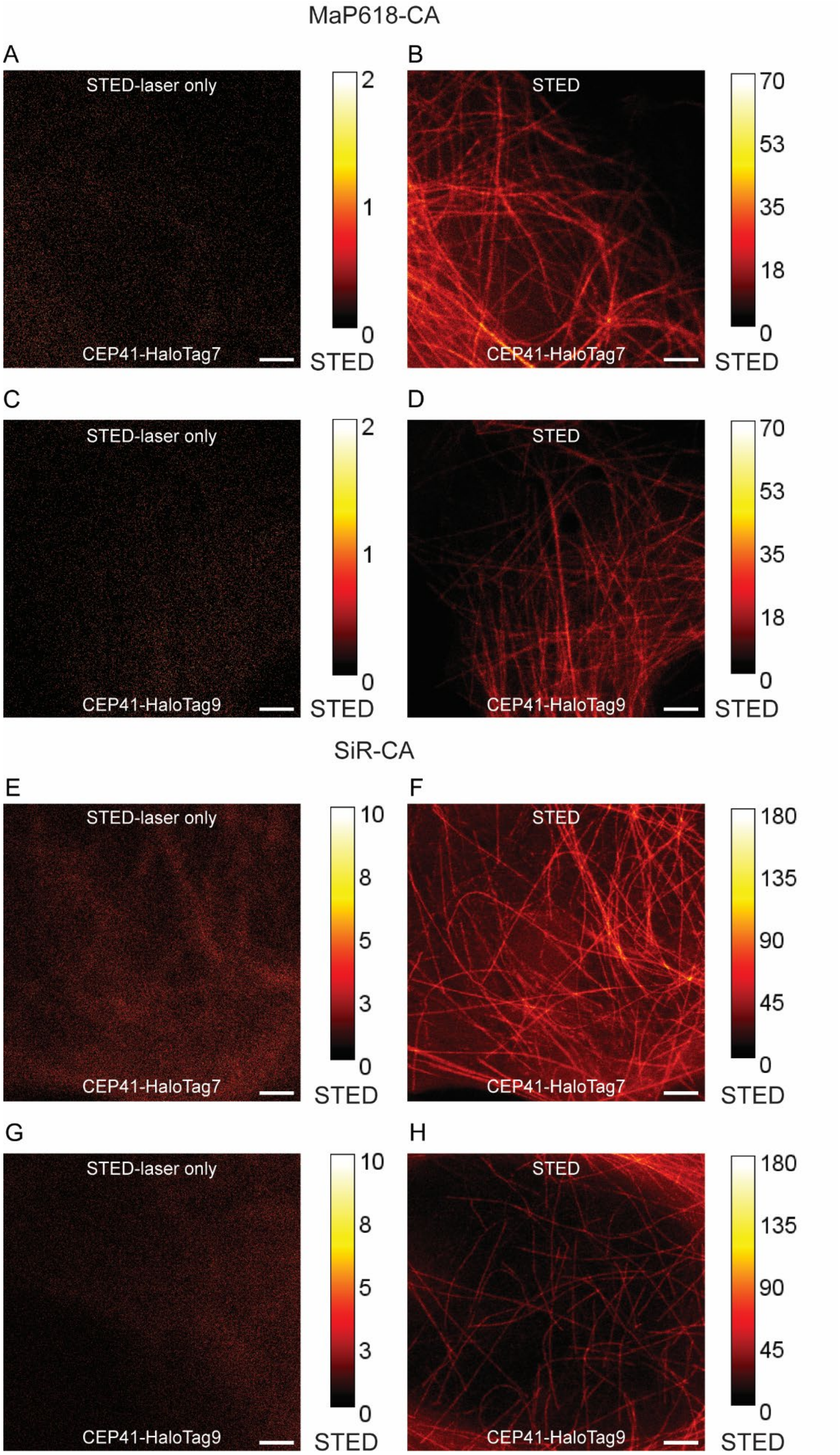
MaP618-CA
A
STED-laser only
CEP41-HaloTag7
2
1
0
STED
B
STED
CEP41-HaloTag7
70
53
35
18
0
STED
C
STED-laser only
CEP41-HaloTag9
2
1
0
STED
D
STED
CEP41-HaloTag9
70
53
35
18
0
STED
SiR-CA
E
STED-laser only
CEP41-HaloTag7
10
8
5
3
0
STED
F
STED
CEP41-HaloTag7
180
135
90
45
0
STED
G
STED-laser only
CEP41-HaloTag9
10
8
5
3
0
STED
H
STED
CEP41-HaloTag9
180
135
90
45
0
STED

Supplementary Figure S16: STED-laser only and STED fluorescence microscopy images of microtubules labeled via HaloTag7 or HaloTag9. **A-H** U-2 OS cells stably expressing the microtubule marker CEP41 as a fusion of HaloTag7 (**A-B**, **E-F**) or HaloTag9 (**C-D**, **G-H**) labeled with MaP618-CA (1 µM, 3 h, **A-D**) or SiR-CA (1 µM, 3 h, **E-H**). Confocal images using the STED laser for excitation (STED-laser only, **A**, **C**, **E**, **G**) and STED images (**B**, **D**, **F**, **H**) are given. Representative images from two independent experiments. Scale bars, 2 µm.

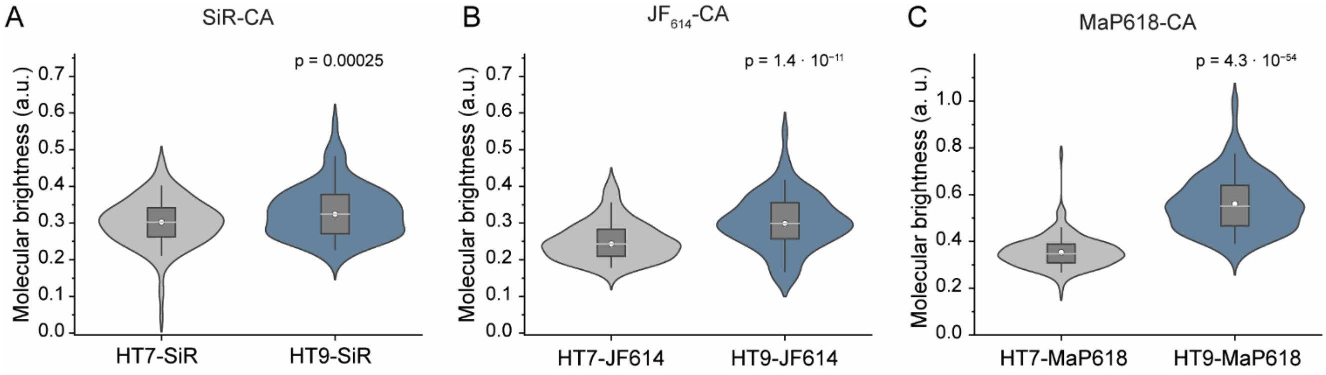

Supplementary Figure S17: Molecular brightness comparison of HaloTag7 and HaloTag9 in mammalian cells by FCS. **A-C** Violin plots of molecular brightness acquired by FCS in living U-2 OS cells expressing either HaloTag7 or HaloTag9 in the cytosol labeled with fluorophore (150 nM, 2 h, SiR-CA (**A**), JF_{614}-CA (**B**) and MaP618-CA (**C**)). 30 s long FCS traces were measured. Molecular brightness was then calculated using the amplitude of the autocorrelation curve as well as the mean fluorescence intensity. SiR-CA labeled HaloTag9 showed a 9±3% (mean±s.e.m.) higher brightness than HaloTag7, for JF_{614}-CA the change was 21±3%, and for MaP618-CA 59±4%. p-Values are given based one-sided t-tests (α = 5%, r = 0.05, 0.18, 0.27, Degrees of freedom (DF) = 296, 291, 323). Distribution = light grey/blue, box = 25%–75% percentile, whiskers = 5%–95% percentile, white line = median, circle = mean. *N* = SiR-CA: 141, 157; JF_{614}-CA: 148, 145; MaP618-CA: 155, 170 traces from 36 cells from three independent preparations (HaloTag7, HaloTag9).

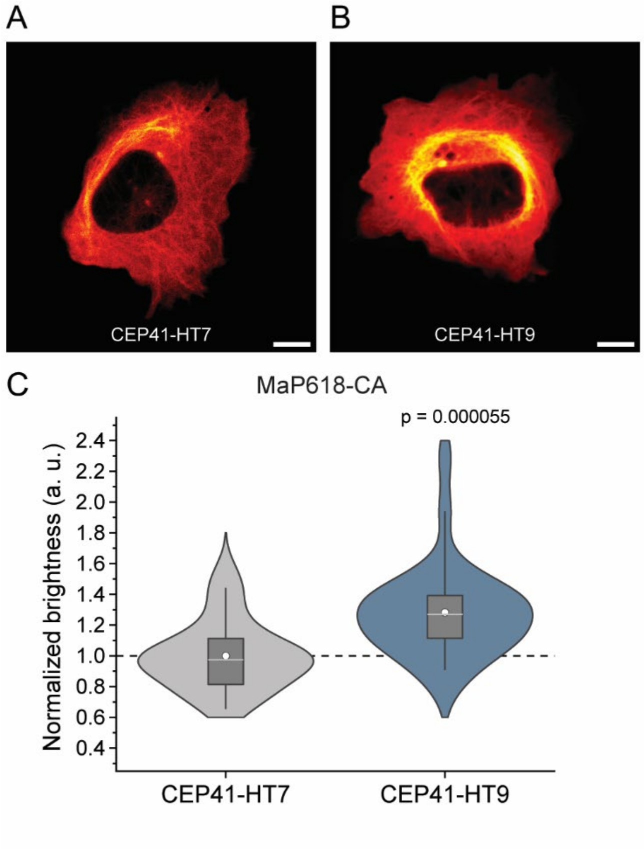

Supplementary Figure S18: Brightness comparison of CEP41-HaloTag7 and CEP41-HaloTag9 in mammalian cells. **A-B** Exemplary confocal fluorescence microscopy images of microtubules labeled with HaloTag7 (**A**) or HaloTag9 (**B**). U-2 OS cells expressing CEP41-HaloTag7 T2A-EGFP or CEP41-HaloTag9 T2A-EGFP labeled with MaP618-CA (1 µM, 3 h). The brightness of the sum projection images was scaled to the expression level (T2A-EGFP) of the two proteins in the two cells. Representative images from three independent experiments. Scale bars, 10 µm. **C** Violin plots of normalized brightness of living U-2 OS cells expressing either CEP41-HaloTag7 T2A-EGFP or CEP41-HaloTag9 T2A-EGFP labeled with MaP618-CA (1 µM, 3 h). z-Stacks were acquired measuring both the MaP618-CA and the EGFP (T2A-EGFP) fluorescence. Sum projections thereof were analyzed for the mean fluorescence intensity within the cell and normalized for expression level variations by the EGFP signal. MaP618-CA labeled CEP41-HaloTag9 showed a 28±8% (mean±s.e.m.) higher brightness than CEP41-HaloTag7. p-Value is given based on a one-sided t-test (α = 5%, r = 0.21, DF = 58). Distribution = light grey/blue, box = 25%–75% percentile, whiskers = 5%–95% percentile, white line = median, circle = mean. *N* = 30, 30 cells from three independent preparations (HaloTag7, HaloTag9).

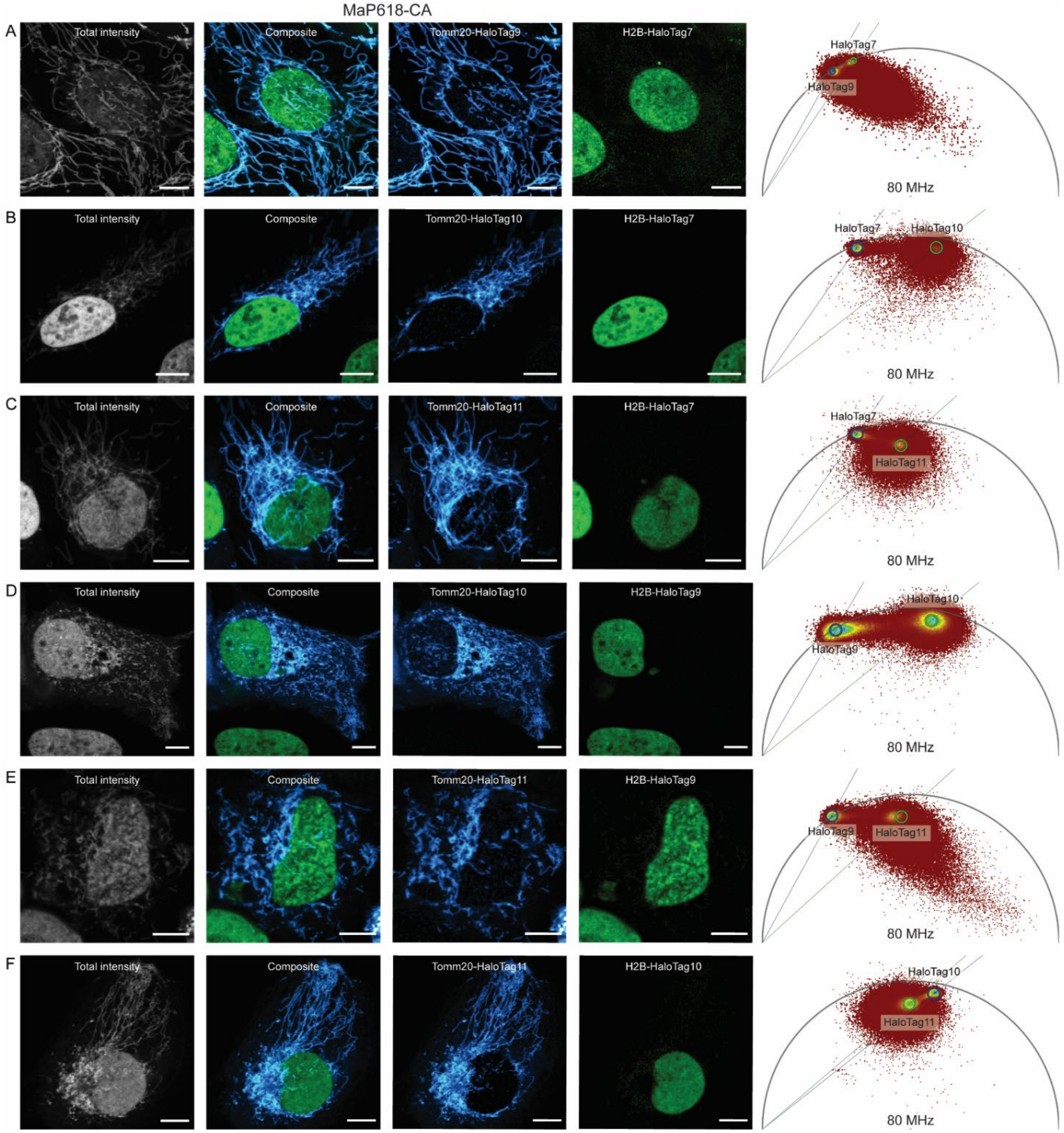

Supplementary Figure S19: Live-cell fluorescence lifetime multiplexing of H2B and Tomm20 fused to different HaloTag variants labeled with MaP618-CA. U-2 OS cells expressing a H2B-fusion (stable) and a Tomm20-fusion (transient) were labeled with MaP618-CA (1 µM, 3 h). **A** H2B-HaloTag7 and Tomm20-HaloTag9. **B** H2B-HaloTag7 and Tomm20-HaloTag10. **C** H2B-HaloTag7 and Tomm20-HaloTag11. **D** H2B-HaloTag9 and Tomm20-HaloTag10. **E** H2B-HaloTag9 and Tomm20-HaloTag11. **F** H2B-HaloTag10 and Tomm20-HaloTag11. The inverse combinations are given in Supplementary Fig. S20. The fluorescence intensity, the composite, the two individual separated species as well as the corresponding wavelet-filtered phasor plot used for separation are given. Species separation was achieved using the phasor approach by positioning the cluster-circles on the phasor plot at the position of the pure species. Scale bars, 10 µm.

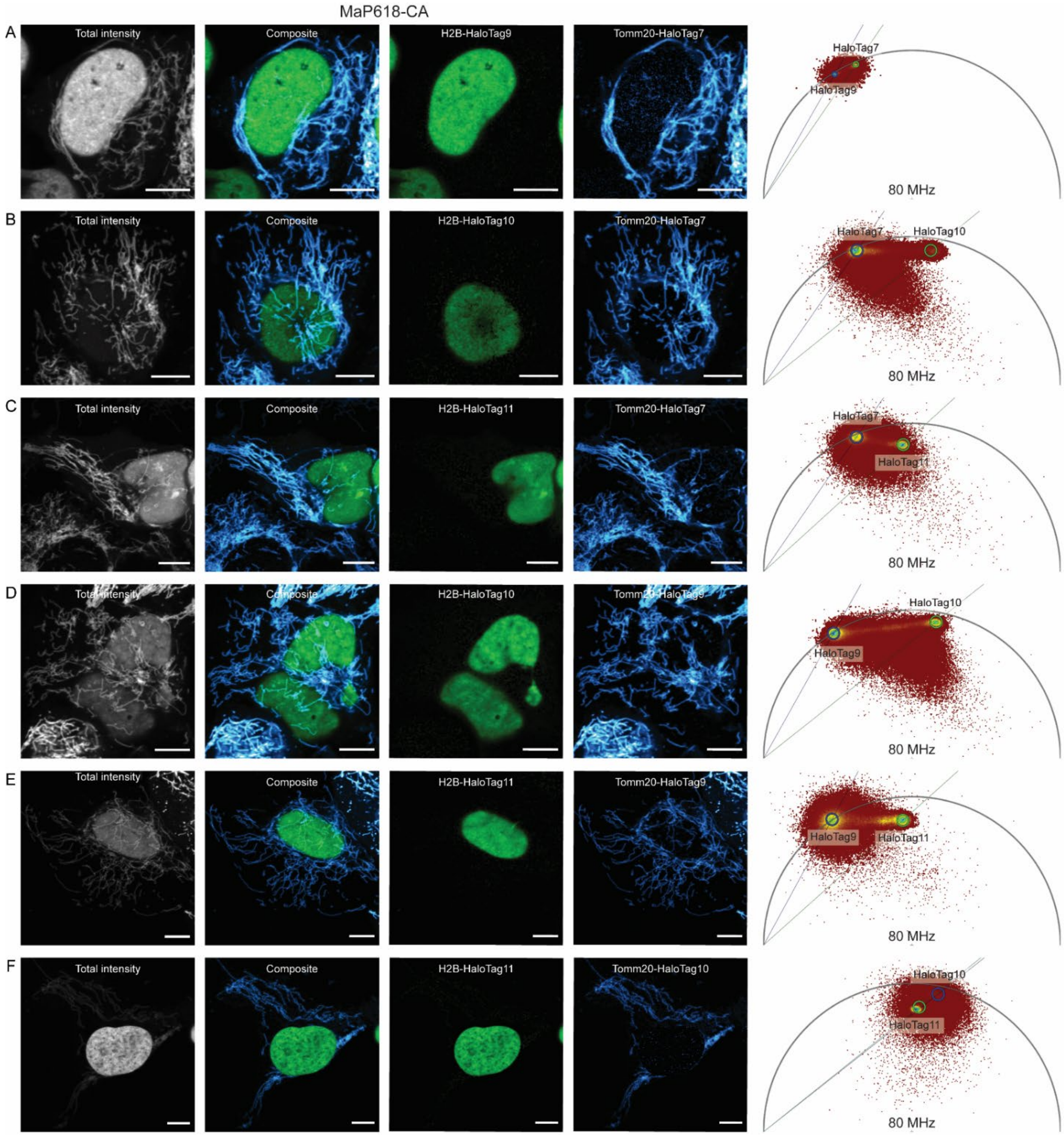

Supplementary Figure S20: Live-cell fluorescence lifetime multiplexing of the inverse combinations of H2B and Tomm20 fused to different HaloTag variants labeled with MaP618-CA. U-2 OS cells expressing a Tomm20-fusion and a H2B-fusion were labeled with MaP618-CA (1 µM, 3 h). **A** Tomm20-HaloTag7 (stable) and H2B-HaloTag9 (transient). **B** Tomm20-HaloTag7 (stable) and H2B-HaloTag10 (transient). **C** Tomm20-HaloTag7 (stable) and H2B-HaloTag11 (transient). **D** Tomm20-HaloTag9 (stable) and H2B-HaloTag10 (transient). **E** Tomm20-HaloTag9 (stable) and H2B-HaloTag11 (transient). **F** Tomm20-HaloTag10 (transient) and H2B-HaloTag11 (stable). The fluorescence intensity, the composite, the two individual separated species as well as the corresponding wavelet-filtered

phasor plot used for separation are given. Species separation was achieved using the phasor approach by positioning the cluster-circles on the phasor plot at the position of the pure species. Scale bars, 10 µm.

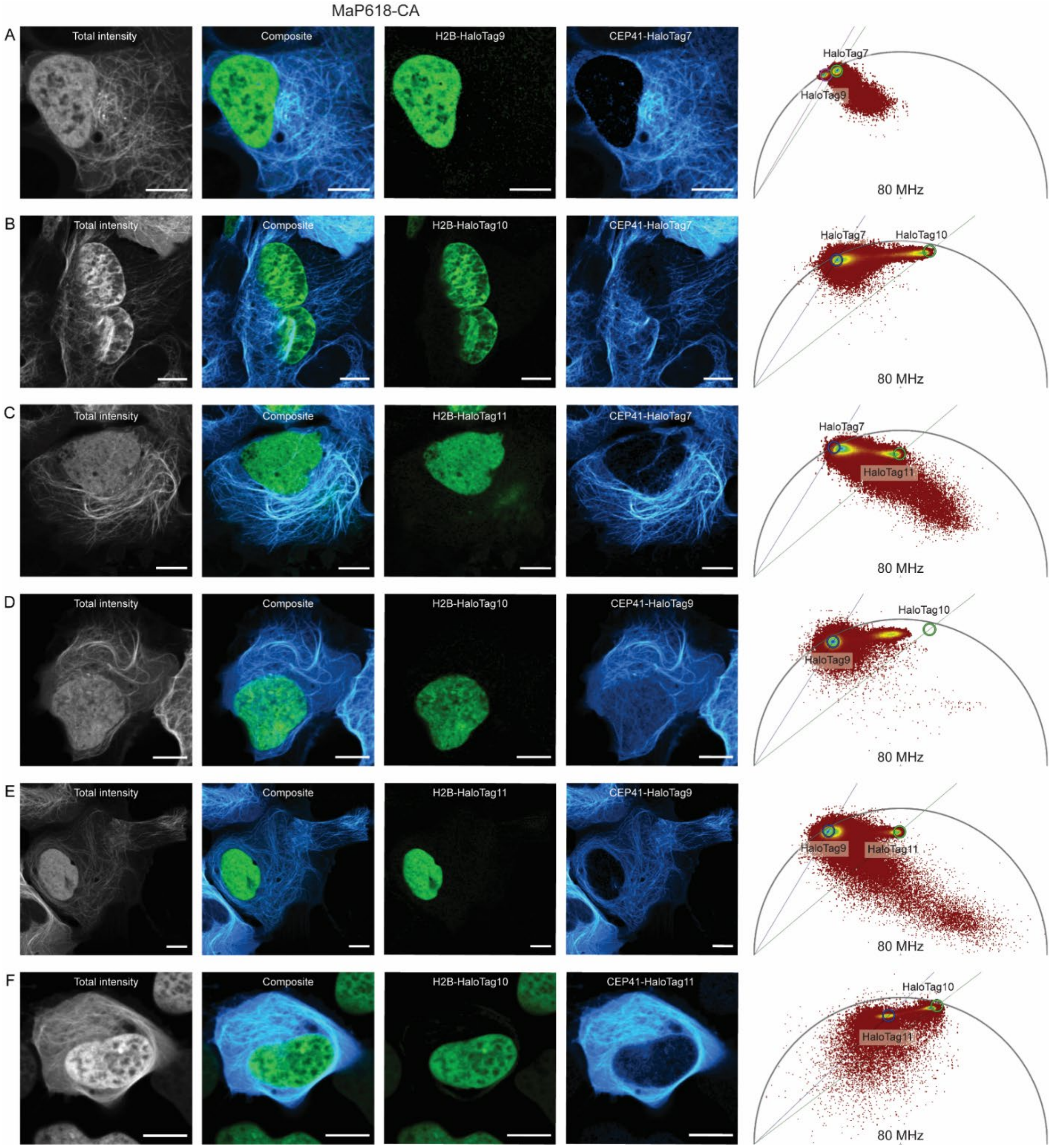

Supplementary Figure S21: Live-cell fluorescence lifetime multiplexing of CEP41 and H2B fused to different HaloTag variants labeled with MaP618-CA. U-2 OS cells expressing a CEP41-fusion and a H2B-fusion were labeled with MaP618-CA (1 µM, 3 h). **A** CEP41-HaloTag7 (stable) and H2B-HaloTag9 (transient). The inverse combination (CEP41-HaloTag9 (stable) and H2B-HaloTag7 (transient)) was also measured and is given in Supplementary Fig. S23A. **B** CEP41-HaloTag7 (stable) and H2B-HaloTag10 (transient). **C** CEP41-HaloTag7 (stable) and H2B-HaloTag11 (transient). **D** CEP41-HaloTag9 (stable) and H2B-HaloTag10 (transient). **E** CEP41-HaloTag9 (stable) and H2B-HaloTag11 (transient). **F** CEP41-HaloTag11 (transient) and H2B-HaloTag10 (stable). The fluorescence intensity, the composite, the two individual separated species as well as the corresponding wavelet-filtered phasor plot used

for separation are given. Species separation was achieved using the phasor approach by positioning the cluster-circles on the phasor plot at the position of the pure species. Scale bars, 10 µm.

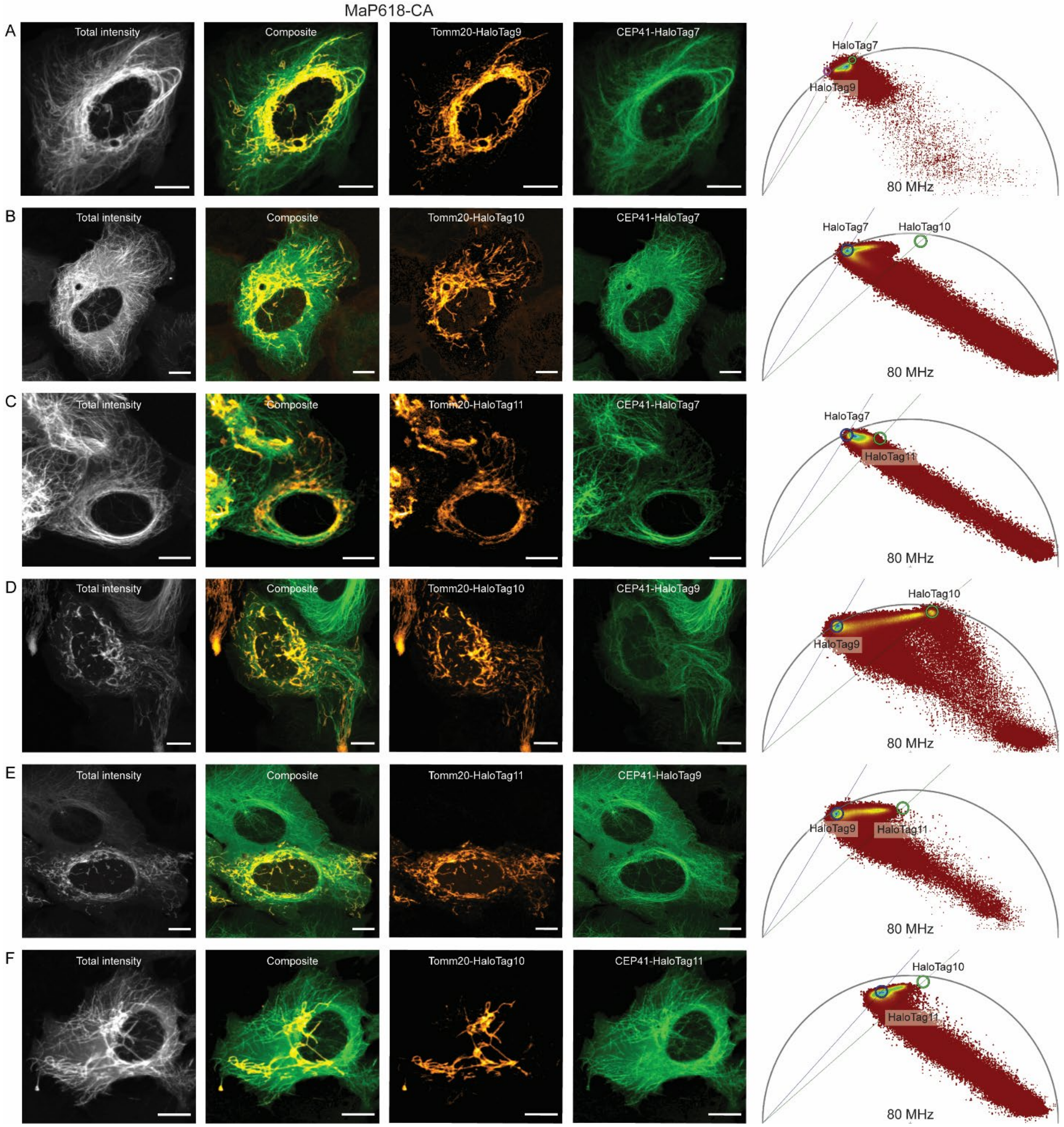

Supplementary Figure S22: Live-cell fluorescence lifetime multiplexing of CEP41 and Tomm20 fused to different HaloTag variants with MaP618-CA. U-2 OS cells expressing a CEP41-fusion and a Tomm20-fusion were labeled with MaP618-CA (1 µM, 3 h). **A** CEP41-HaloTag7 (stable) and Tomm20-HaloTag9 (transient). **B** CEP41-HaloTag7 (stable) and Tomm20-HaloTag10 (transient). **C** CEP41-HaloTag7 (stable) and Tomm20-HaloTag11 (transient). **D** CEP41-HaloTag9 (stable) and Tomm20-HaloTag10 (transient). **E** CEP41-HaloTag9 (stable) and Tomm20-HaloTag11 (transient). **F** CEP41-HaloTag11 (stable) and Tomm20-HaloTag10 (transient). The inverse combination was measured for the combination of CEP41-HaloTag9 (stable) and Tomm20-HaloTag7 (transient) and is given in Supplementary Fig. S23B. The fluorescence intensity, the composite, the two individual separated species as well as the corresponding wavelet-filtered phasor plot used for separation are given. Species separation was achieved

using the phasor approach by positioning the cluster-circles on the phasor plot at the position of the pure species. Scale bars, 10 µm.

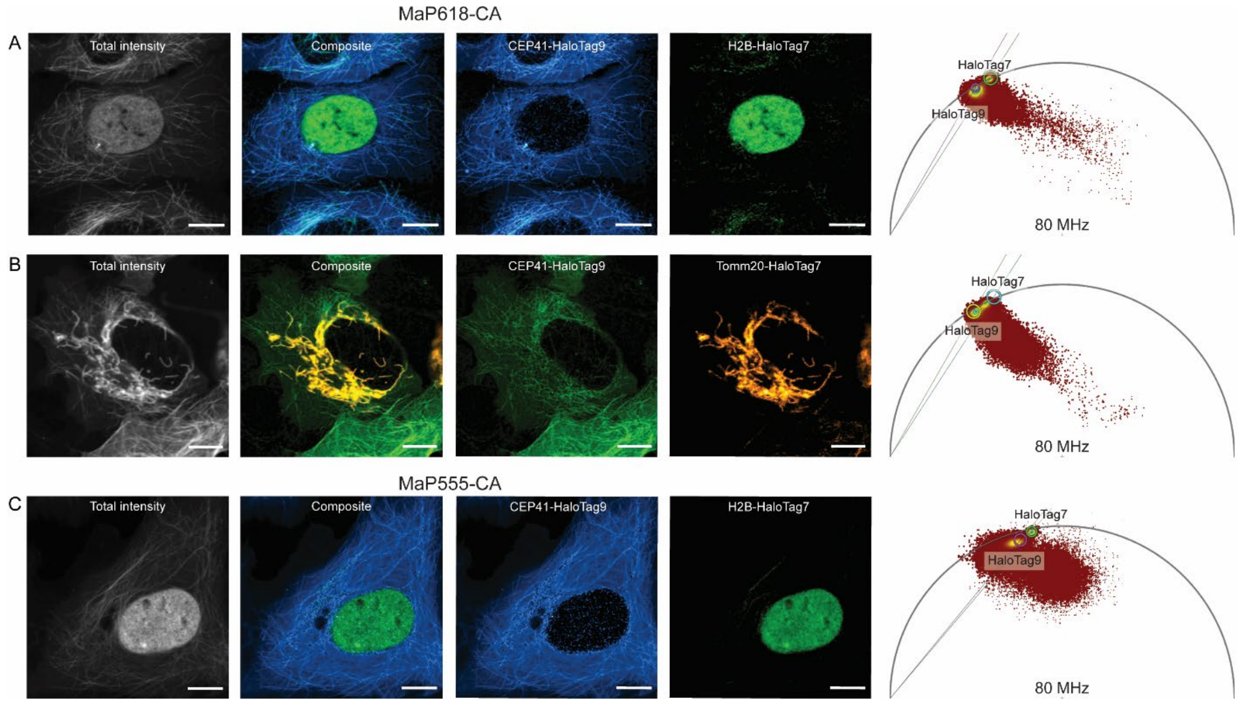

Supplementary Figure S23: Live-cell fluorescence lifetime multiplexing of inverse combinations of fusion proteins with HaloTag variants. **A** U-2 OS cells expressing CEP41-HaloTag9 (stable) and H2B-HaloTag7 (transient) or **B** CEP41-HaloTag9 (stable) and Tomm20-HaloTag7 (transient) labeled with MaP618-CA (1 µᴍ, 3 h). **C** U-2 OS cells expressing CEP41-HaloTag9 (stable) and H2B-HaloTag7 (transient) were labeled with MaP555-CA (1 µᴍ, 3 h). The fluorescence intensity, the composite, the two individual separated species as well as the corresponding wavelet-filtered phasor plot used for separation are given. Species separation was achieved using the phasor approach by positioning the cluster-circles on the phasor plot at the position of the pure species. Scale bars, 10 µm.

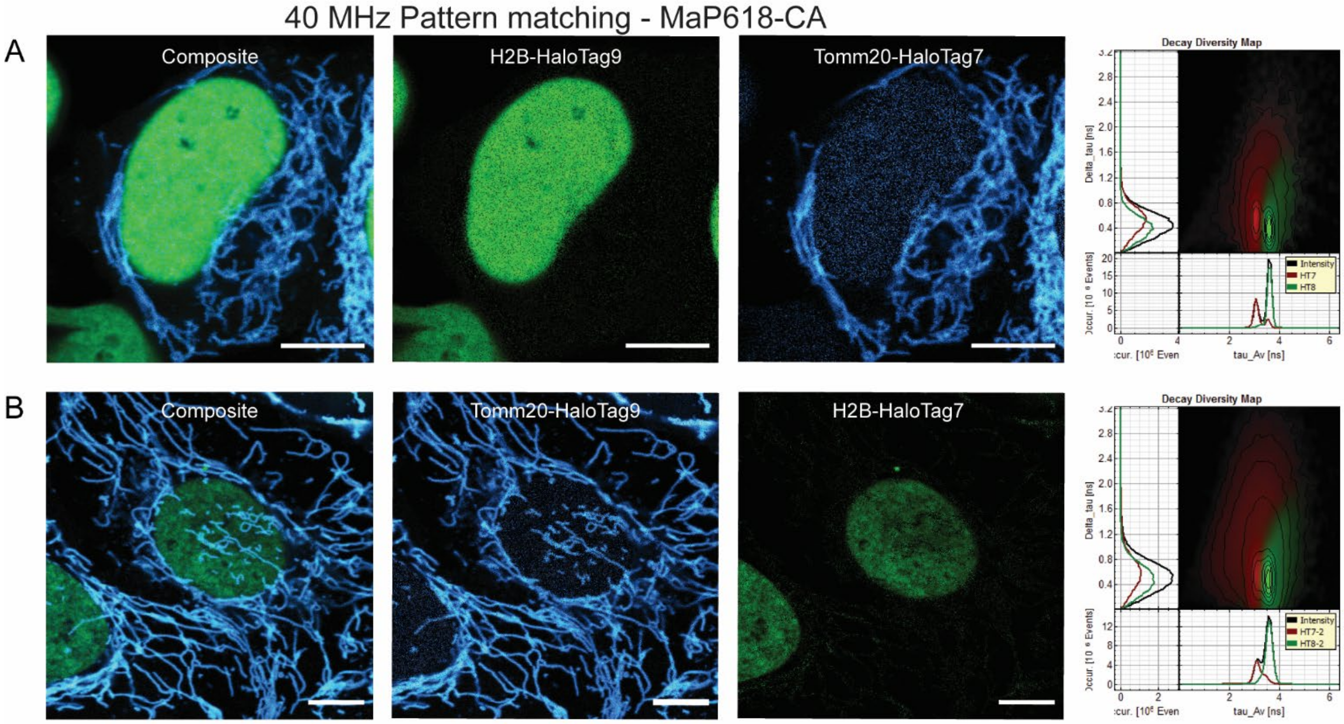

Supplementary Figure S24: Live-cell fluorescence lifetime multiplexing using pattern matching analysis to separate the two components. **A-B** Analysis of the FLIM images given in Supplementary Fig. S19A and 20A via pattern matching gave similar results as the separation using the phasor plot. However, only images acquired at 40 MHz instead of 80 MHz could be separated reliably using pattern matching. The composite, the two individual separated components as well as the corresponding decay diversity map used for separation are given. The presented images were acquired under the same conditions as the images in Supplementary Fig. S19A and 20A apart from the laser repetition rate, which was set to 40 MHz instead of 80 MHz. Scale bars, 10 µm.

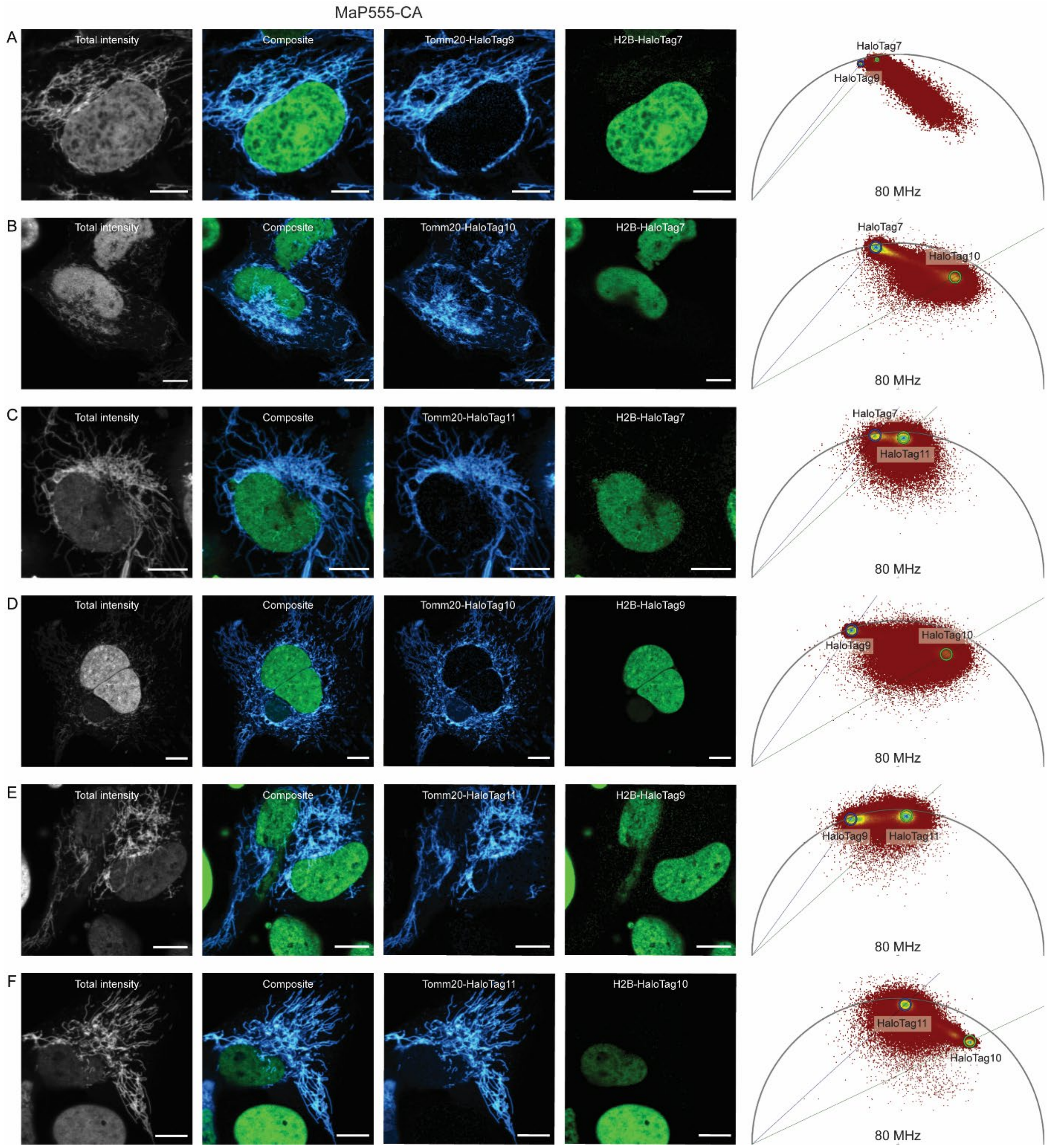

Supplementary Figure S25: Live-cell fluorescence lifetime multiplexing of H2B and Tomm20 fused to different HaloTag variants labeled with MaP555-CA. U-2 OS cells expressing a H2B-fusion (stable) and a Tomm20-fusion (transient) were labeled with MaP555-CA (1 µM, 3 h). **A** H2B-HaloTag7 and Tomm20-HaloTag9. **B** H2B-HaloTag7 and Tomm20-HaloTag10. **C** H2B-HaloTag7 and Tomm20-HaloTag11. **D** H2B-HaloTag9 and Tomm20-HaloTag10. **E** H2B-HaloTag9 and Tomm20-HaloTag11. **F** H2B-HaloTag10 and Tomm20-HaloTag11. The inverse combinations are given in Supplementary Fig. S26. The fluorescence intensity, the composite, the two individual separated species as well as the corresponding wavelet-filtered phasor plot used for separation are given. Species separation was achieved using the phasor approach by positioning the cluster-circles on the phasor plot at the position of the pure species. Scale bars, 10 µm.

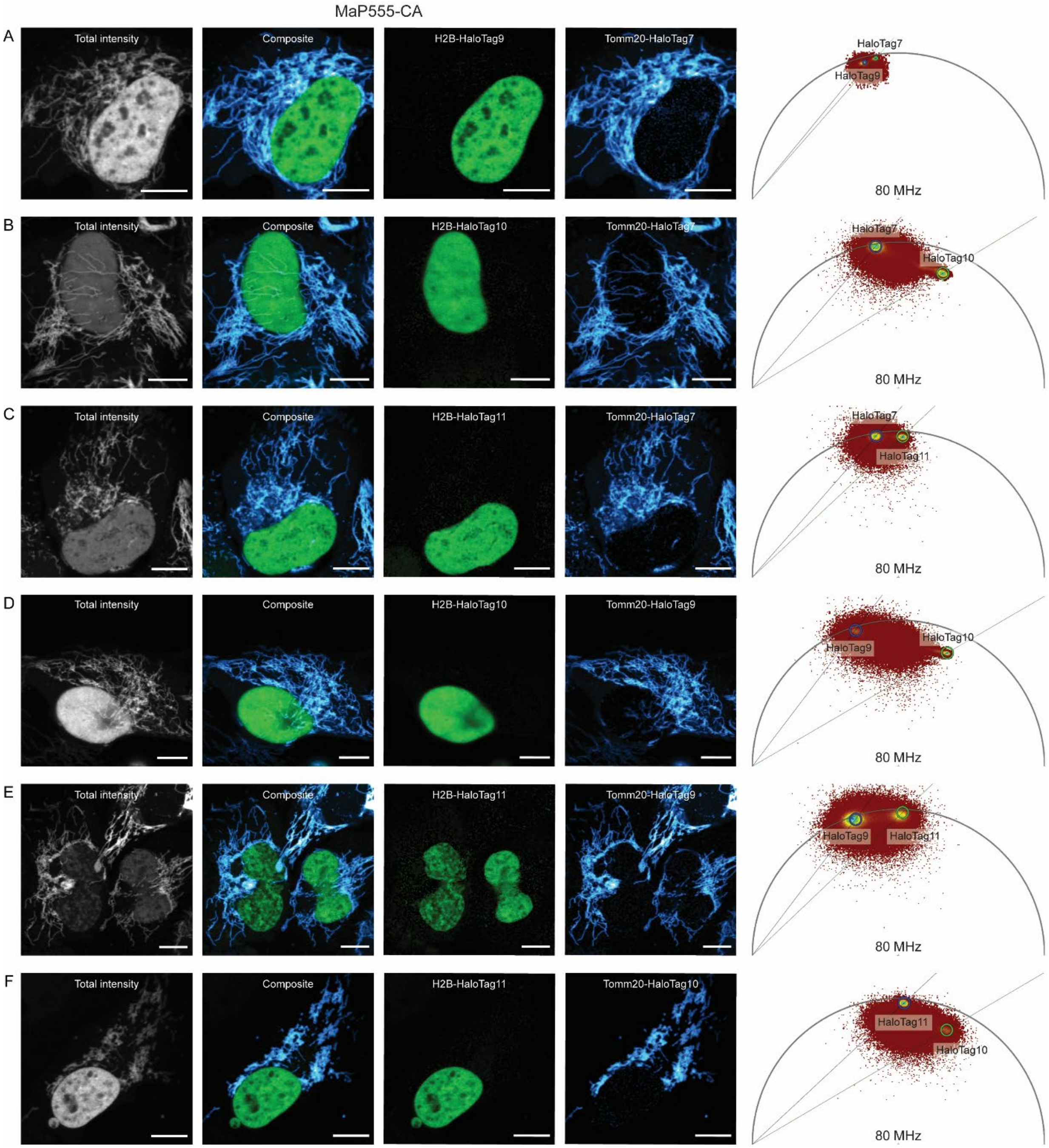

Supplementary Figure S26: Live-cell fluorescence lifetime multiplexing of the inverse combinations of H2B and Tomm20 fused to different HaloTag variants labeled with MaP555-CA. U-2 OS cells expressing a Tomm20-fusion and a H2B-fusion were labeled with MaP555-CA (1 µM, 3 h). **A** Tomm20-HaloTag7 (stable) and H2B-HaloTag9 (transient). **B** Tomm20-HaloTag7 (stable) and H2B-HaloTag10 (transient). **C** Tomm20-HaloTag7 (stable) and H2B-HaloTag11 (transient). **D** Tomm20-HaloTag9 (stable) and H2B-HaloTag10 (transient). **E** Tomm20-HaloTag9 (stable) and H2B-HaloTag11 (transient). **F** Tomm20-HaloTag10 (transient) and H2B-HaloTag11 (stable). The fluorescence intensity, the composite, the two individual separated species as well as the corresponding wavelet-filtered

phasor plot used for separation are given. Species separation was achieved using the phasor approach by positioning the cluster-circles on the phasor plot at the position of the pure species. Scale bars, 10 µm.

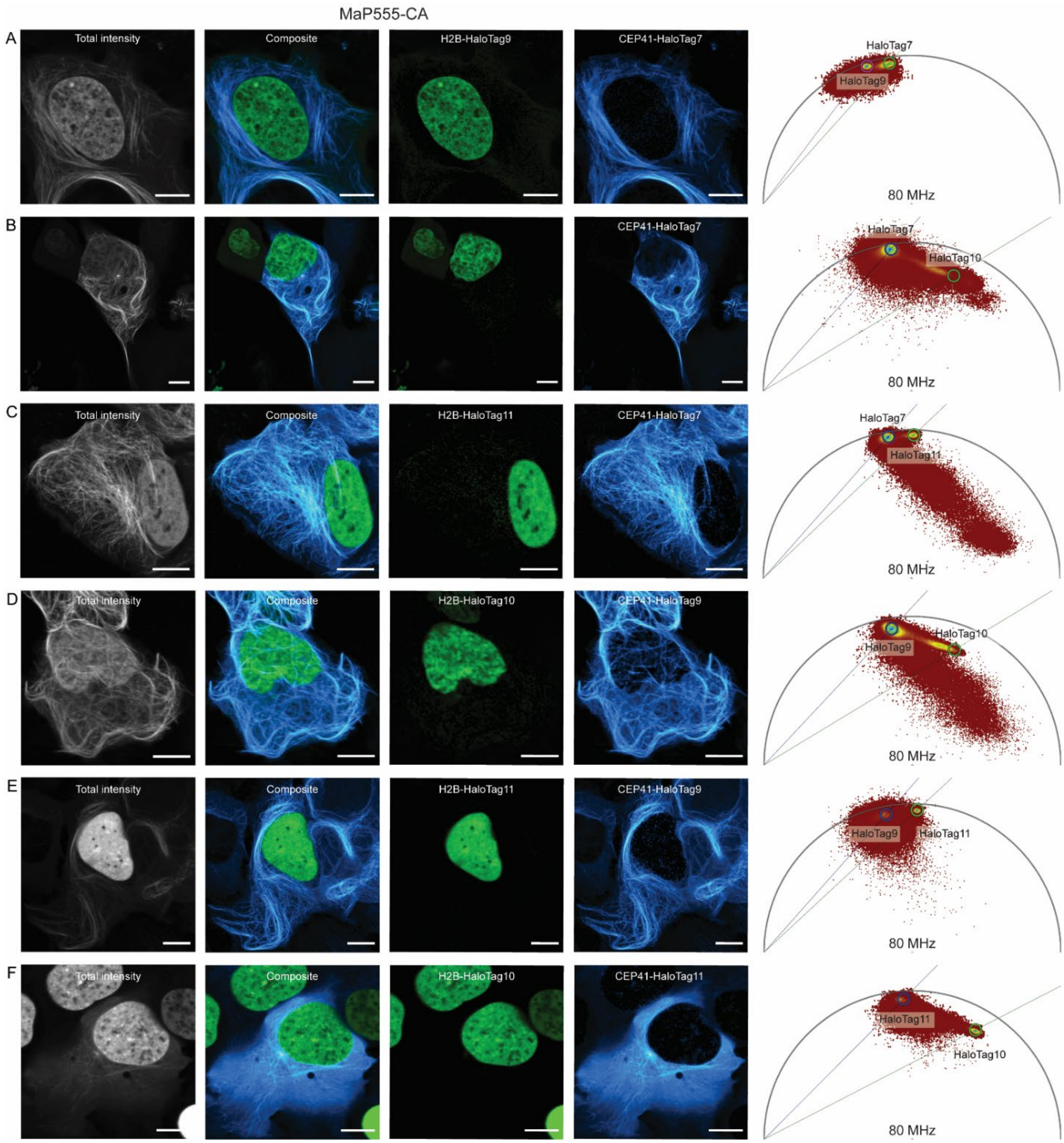

Supplementary Figure S27: Live-cell fluorescence lifetime multiplexing of CEP41 and H2B fused to different HaloTag variants labeled with MaP555-CA. U-2 OS cells expressing a CEP41-fusion and a H2B-fusion were labeled with MaP555-CA (1 µM, 3 h). **A** CEP41-HaloTag7 (stable) and H2B-HaloTag9 (transient). The inverse combination (CEP41-HaloTag9 (stable) and H2B-HaloTag7 (transient)) was measured and is given in Supplementary Fig. S23C. **B** CEP41-HaloTag7 (stable) and H2B-HaloTag10 (transient). **C** CEP41-HaloTag7 (stable) and H2B-HaloTag11 (transient). **D** CEP41-HaloTag9 (stable) and H2B-HaloTag10 (transient). **E** CEP41-HaloTag9 (stable) and H2B-HaloTag11 (transient). **F** CEP41-HaloTag11 (transient) and H2B-HaloTag10 (stable). The fluorescence intensity, the composite, the two individual separated species as well as the corresponding wavelet-filtered phasor plot used

for separation are given. Species separation was achieved using the phasor approach by positioning the cluster-circles on the phasor plot at the position of the pure species. Scale bars, 10 µm.

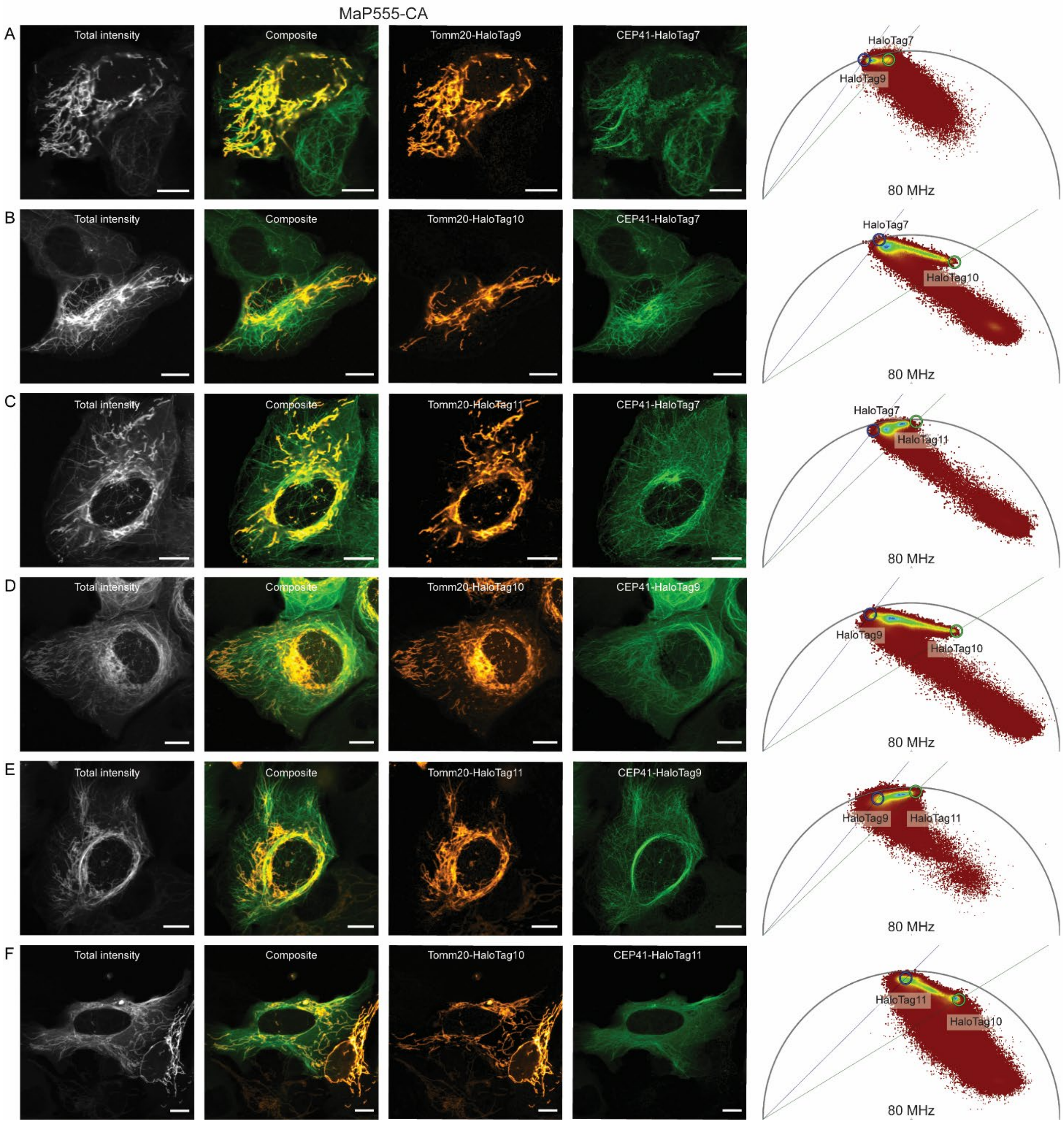

Supplementary Figure S28: Live-cell fluorescence lifetime multiplexing of CEP41 and Tomm20 fused to different HaloTag variants labeled with MaP555-CA. U-2 OS cells expressing a CEP41-fusion and a Tomm20-fusion were labeled with MaP618-CA (1 µм, 3 h). **A** CEP41-HaloTag7 (stable) and Tomm20-HaloTag9 (transient). **B** CEP41-HaloTag7 (stable) and Tomm20-HaloTag10 (transient). **C** CEP41-HaloTag7 (stable) and Tomm20-HaloTag11 (transient). **D** CEP41-HaloTag9 (stable) and Tomm20-HaloTag10 (transient). **E** CEP41-HaloTag9 (stable) and Tomm20-HaloTag11 (transient). **F** CEP41-HaloTag11 (stable) and Tomm20-HaloTag10 (transient). The fluorescence intensity, the composite, the two individual separated species as well as the corresponding wavelet-filtered phasor plot used for separation are given. Species separation was achieved using the phasor approach by positioning the cluster-circles on the phasor plot at the position of the pure species. Scale bars, 10 µm.

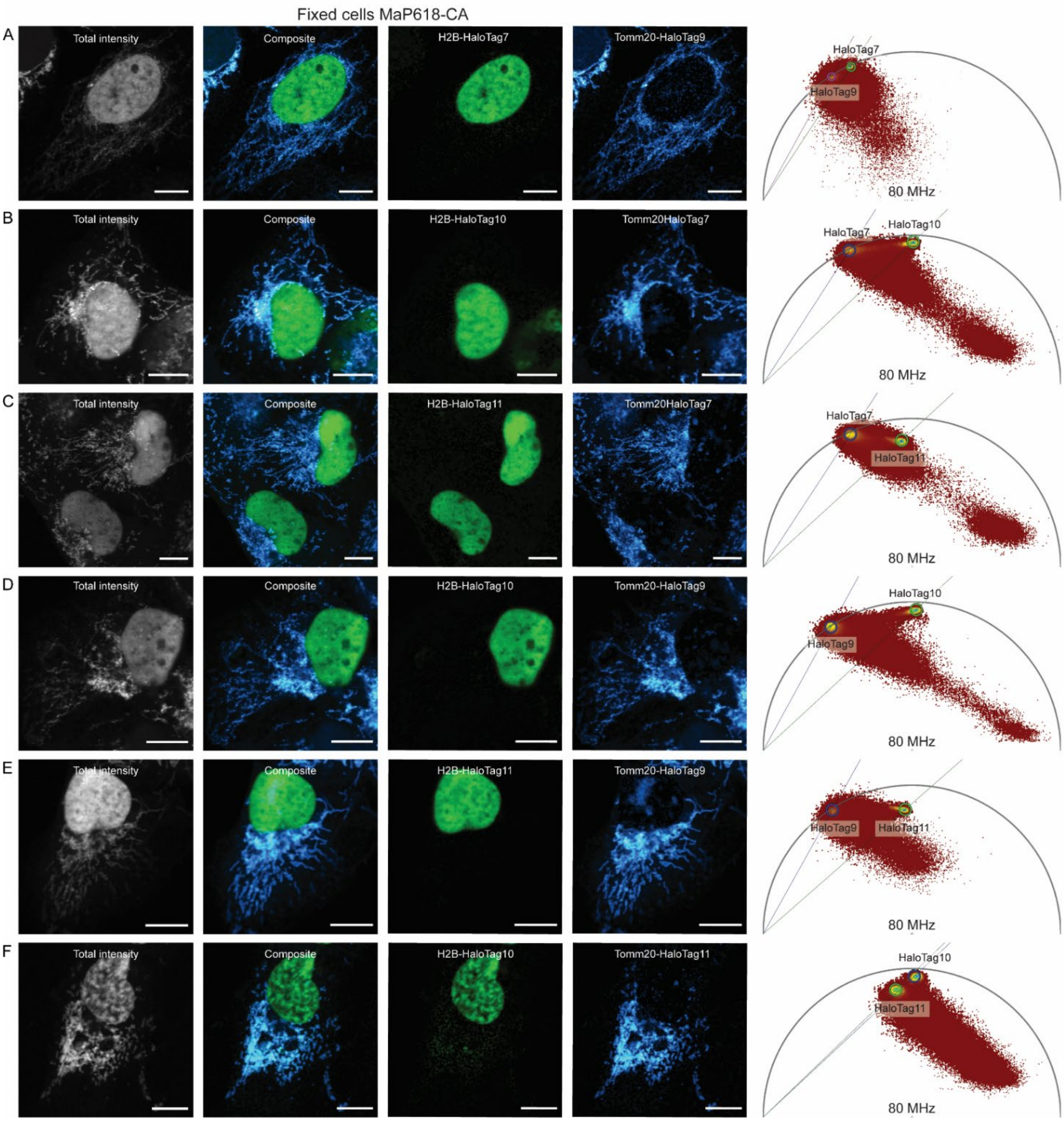

Supplementary Figure 29: Fluorescence lifetime multiplexing in fixed cells labeled with MaP618-CA. U-2 OS cells expressing a Tomm20-fusion and a H2B-fusion were labeled with MaP618-CA (1 µM, 3 h). **A** Tomm20-HaloTag9 (stable) and H2B-HaloTag7 (transient). **B** Tomm20-HaloTag7 (stable) and H2B-HaloTag10 (transient). **C** Tomm20-HaloTag7 (stable) and H2B-HaloTag11 (transient). **D** Tomm20-HaloTag9 (stable) and H2B-HaloTag10 (transient). **E** Tomm20-HaloTag9 (stable) and H2B-HaloTag11 (transient). **F** Tomm20-HaloTag11 (transient) and H2B-HaloTag10 (stable). The fluorescence intensity, the composite, the two individual separated species as well as the corresponding wavelet-filtered phasor plot used for separation are given. Species separation was achieved using the phasor approach by positioning the cluster-circles on the phasor plot at the position of the pure species. Scale bars, 10 µm.

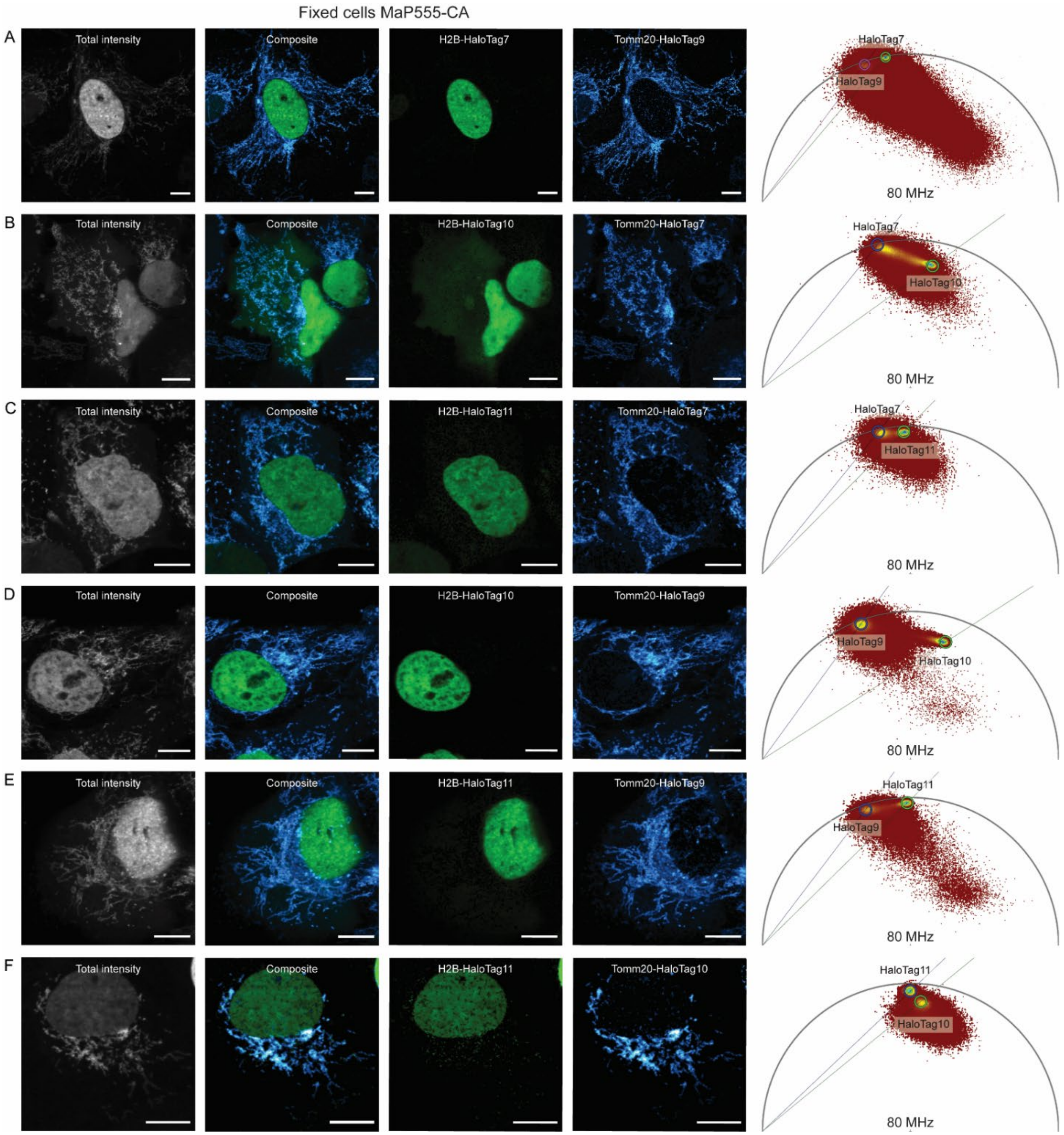

Supplementary Figure S30: Fluorescence lifetime multiplexing in fixed cells labeled with MaP555-CA. U-2 OS cells expressing a Tomm20-fusion and a H2B-fusion were labeled with MaP555-CA (1 µM, 3 h). **A** Tomm20-HaloTag9 (stable) and H2B-HaloTag7 (transient). **B** Tomm20-HaloTag7 (stable) and H2B-HaloTag10 (transient). **C** Tomm20-HaloTag7 (stable) and H2B-HaloTag11 (transient). **D** Tomm20-HaloTag9 (stable) and H2B-HaloTag10 (transient). **E** Tomm20-HaloTag9 (stable) and H2B-HaloTag11 (transient). **F** Tomm20-HaloTag10 (transient) and H2B-HaloTag11 (stable). The fluorescence intensity, the composite, the two individual separated species as well as the corresponding wavelet-filtered phasor plot used for separation are given. Species separation was achieved using the phasor approach by positioning the cluster-circles on the phasor plot at the position of the pure species. Scale bars, 10 µm.

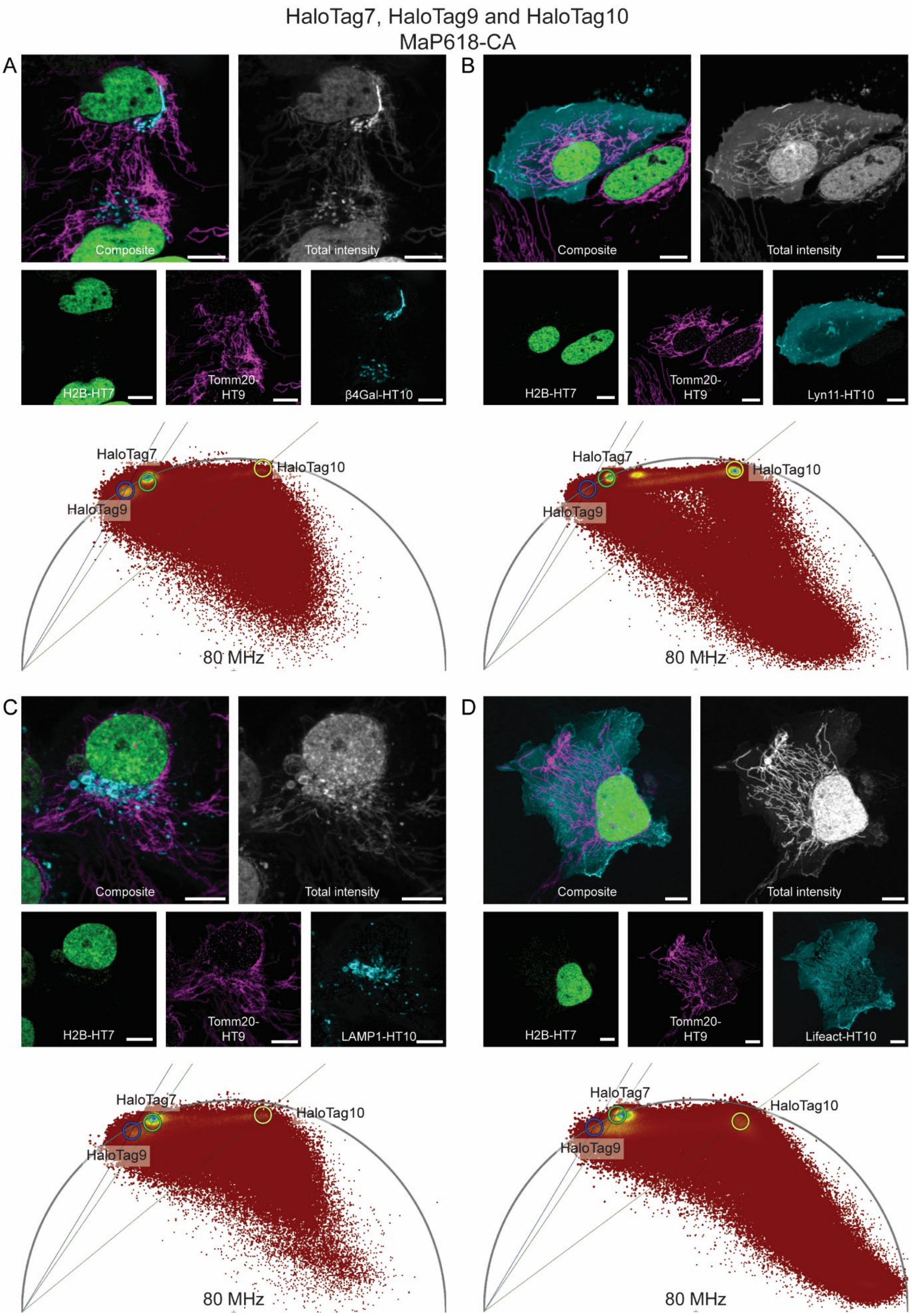
HaloTag7, HaloTag9 and HaloTag10
MaP618-CA
A
Composite
Total intensity
H2B-HT7
Tomm20-HT9
β4Gal-HT10
HaloTag7
HaloTag10
HaloTag9
80 MHz
B
Composite
Total intensity
H2B-HT7
Tomm20-HT9
Lyn11-HT10
HaloTag7
HaloTag10
HaloTag9
80 MHz
C
Composite
Total intensity
H2B-HT7
Tomm20-HT9
LAMP1-HT10
HaloTag7
HaloTag10
HaloTag9
80 MHz
D
Composite
Total intensity
H2B-HT7
Tomm20-HT9
Lifeact-HT10
HaloTag7
HaloTag10
HaloTag9
80 MHz

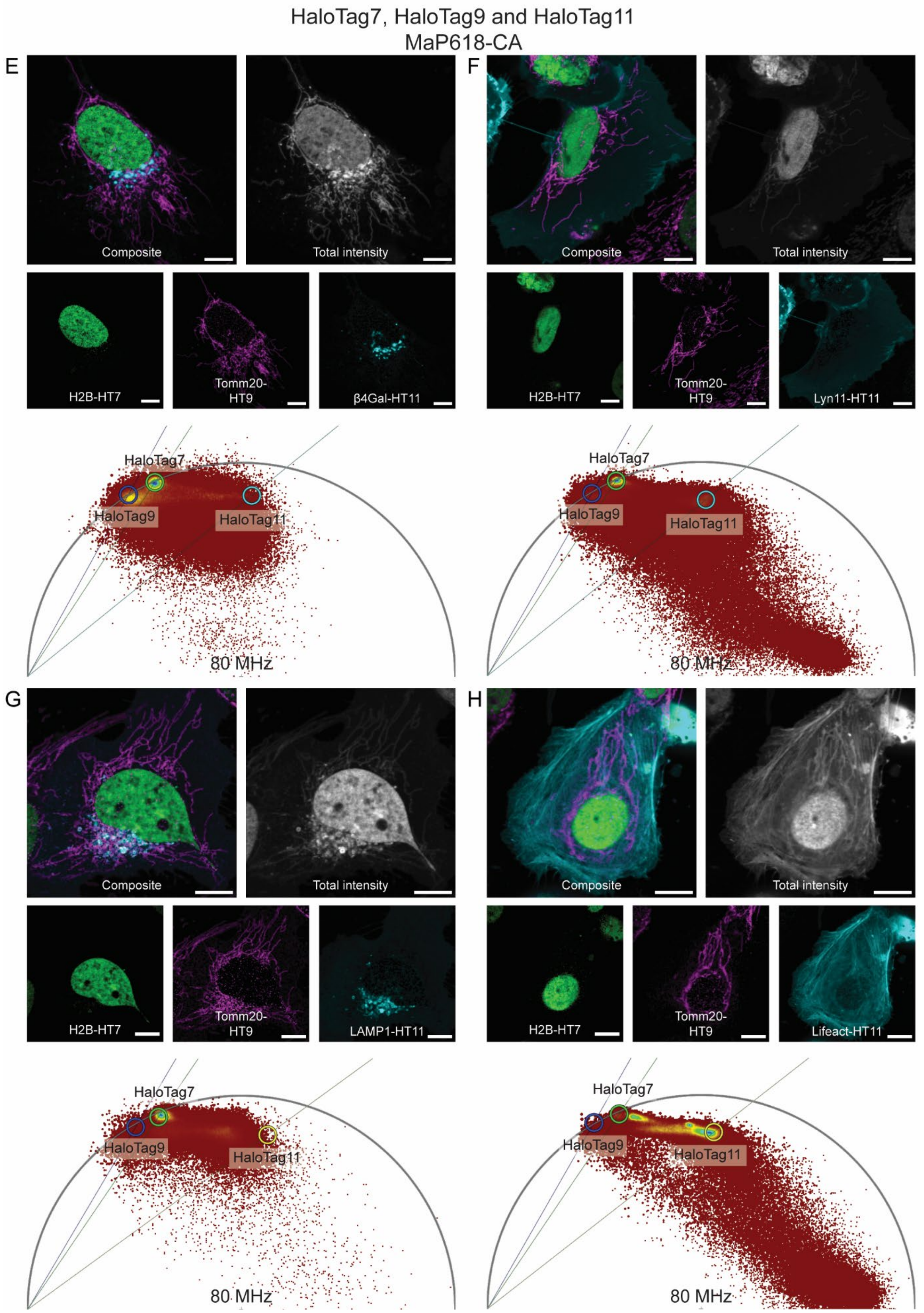
HaloTag7, HaloTag9 and HaloTag11
MaP618-CA
E
Composite
Total intensity
H2B-HT7
Tomm20-HT9
β4Gal-HT11
HaloTag7
HaloTag9
HaloTag11
80 MHz
F
Composite
Total intensity
H2B-HT7
Tomm20-HT9
Lyn11-HT11
HaloTag7
HaloTag9
HaloTag11
80 MHz
G
Composite
Total intensity
H2B-HT7
Tomm20-HT9
LAMP1-HT11
HaloTag7
HaloTag9
HaloTag11
80 MHz
H
Composite
Total intensity
H2B-HT7
Tomm20-HT9
Lifeact-HT11
HaloTag7
HaloTag9
HaloTag11
80 MHz

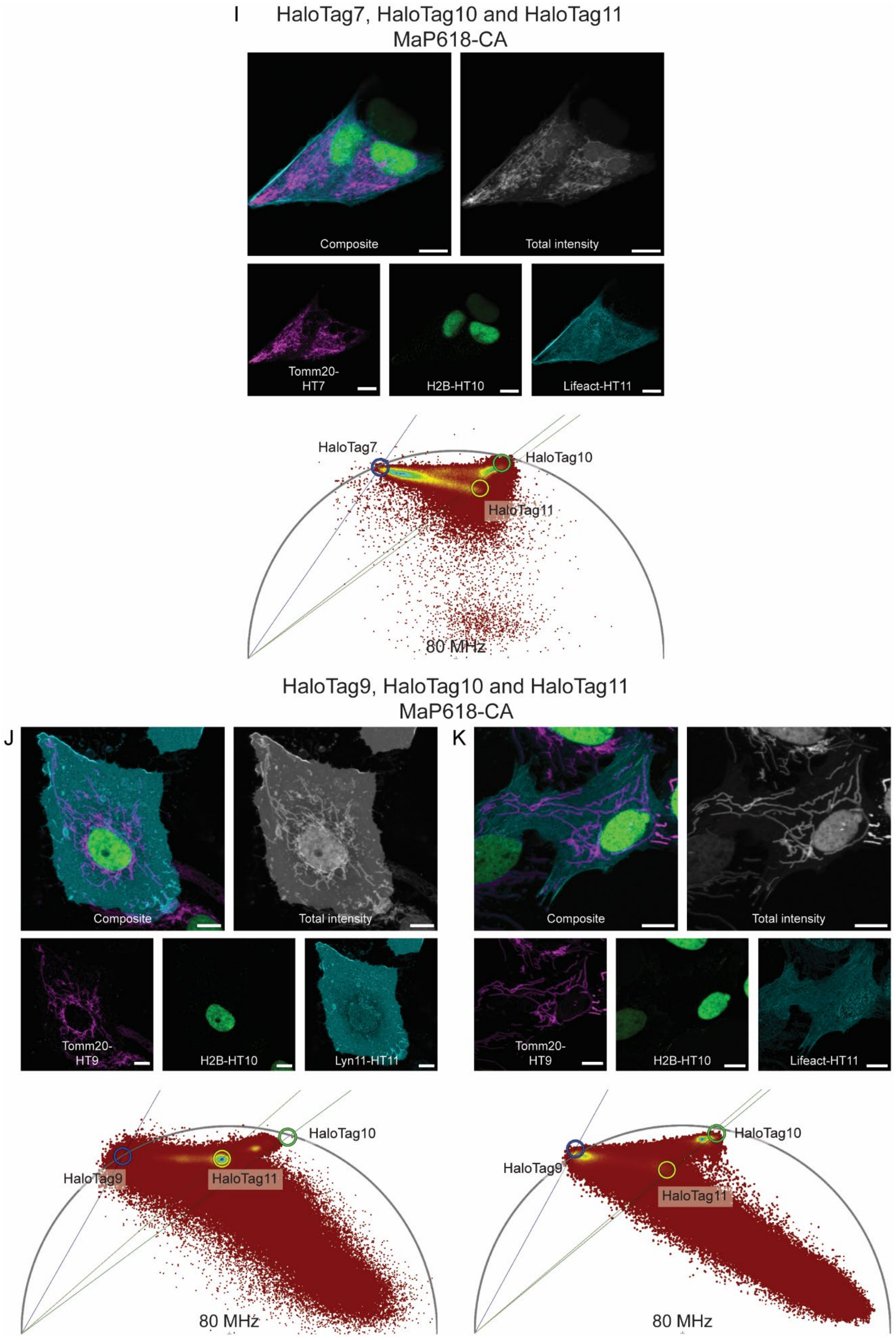
I
HaloTag7, HaloTag10 and HaloTag11
MaP618-CA
Composite
Total intensity
Tomm20-
HT7
H2B-HT10
Lifeact-HT11
HaloTag7
HaloTag10
HaloTag11
80 MHz
HaloTag9, HaloTag10 and HaloTag11
MaP618-CA
J
K
Composite
Total intensity
Tomm20-
HT9
H2B-HT10
Lyn11-HT11
Composite
Total intensity
Tomm20-
HT9
H2B-HT10
Lifeact-HT11
HaloTag10
HaloTag9
HaloTag11
80 MHz
HaloTag10
HaloTag9
HaloTag11
80 MHz

Supplementary Figure S31: Live-cell fluorescence lifetime multiplexing of three HaloTags labeled with MaP618-CA. **A-D** Combination of HaloTag7, HaloTag9 and HaloTag10. Live U-2 OS cells expressing H2B-HaloTag7 and Tomm20-HaloTag9 (stable) and **A** β-4Gal-T1-HaloTag10 **B** Lyn11-HaloTag10 **C** LAMP1-HaloTag10 **D** Lifeact-HaloTag10 (transient). **E-H** Combination of HaloTag7, HaloTag9 and HaloTag11. Live U-2 OS cells expressing H2B-HaloTag7 and Tomm20-HaloTag9 (stable) and **E** β-4Gal-T1-HaloTag11 **F** Lyn11-HaloTag11 **G** LAMP1-HaloTag11 **H** Lifeact-HaloTag11 (transient). **I** Combination of HaloTag7, HaloTag10 and HaloTag11. Live U-2 OS cells expressing H2B-HaloTag10 (stable) and **H** Lifeact-HaloTag11-T2A-Tomm20-HaloTag7 (transient). **J-K** Combination of HaloTag9, HaloTag10 and HaloTag11. Live U-2 OS cells expressing Tomm20-HaloTag9 and H2B-HaloTag10 (stable) and **J** Lyn11-HaloTag11 **K** Lifeact-HaloTag11 (transient). All cells were labeled with MaP618-CA (1 µᴍ, 3 h). The fluorescence intensity, the composite, the three individual separated species as well as the corresponding wavelet-filtered phasor plot used for separation are given. Species separation was achieved using the phasor approach. Scale bars, 10 µm.

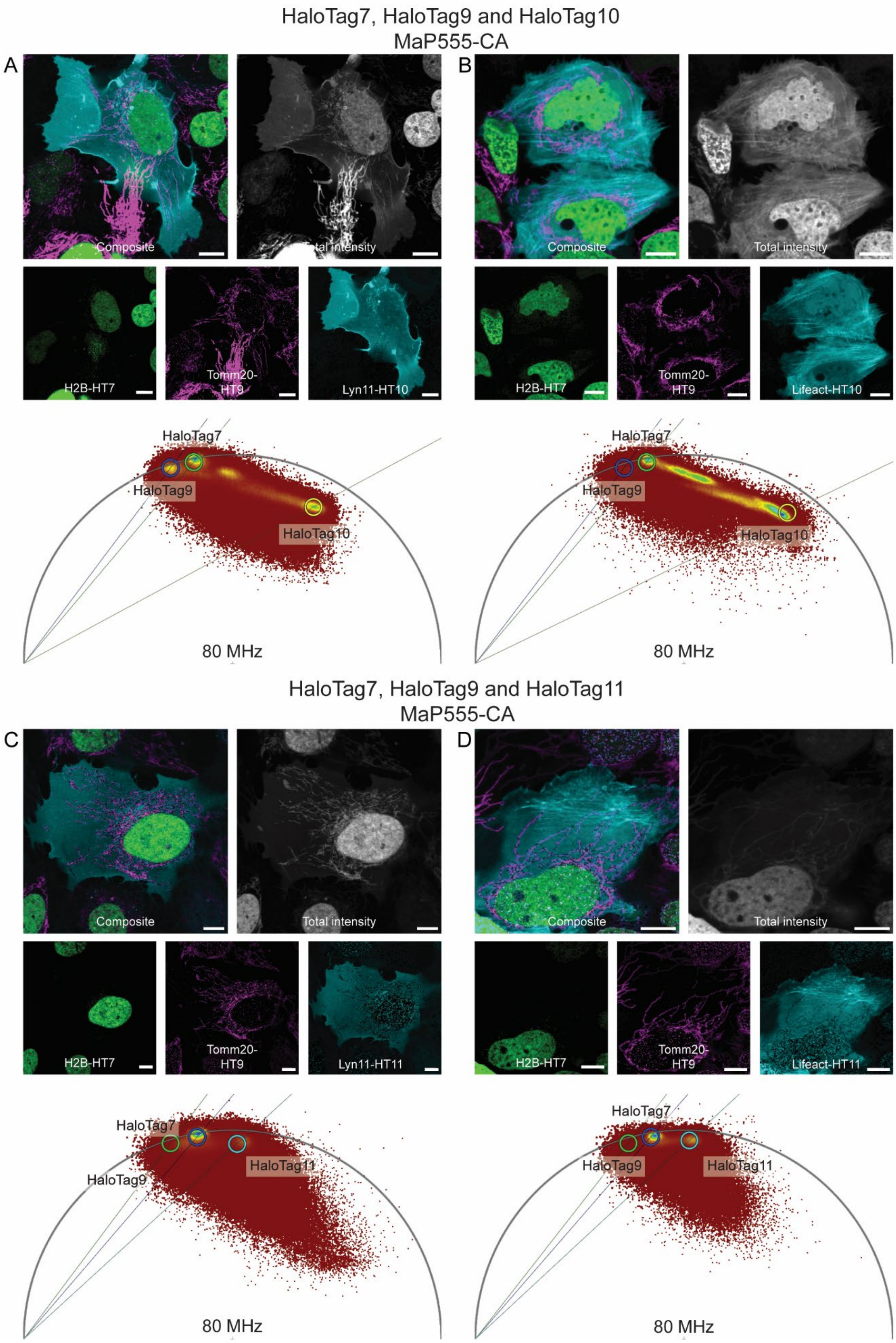

HaloTag7, HaloTag9 and HaloTag10
MaP555-CA
A
Composite
Total intensity
H2B-HT7
Tomm20-HT9
Lyn11-HT10
HaloTag7
HaloTag9
HaloTag10
80 MHz
B
Composite
Total intensity
H2B-HT7
Tomm20-HT9
Lifeact-HT10
HaloTag7
HaloTag9
HaloTag10
80 MHz
HaloTag7, HaloTag9 and HaloTag11
MaP555-CA
C
Composite
Total intensity
H2B-HT7
Tomm20-HT9
Lyn11-HT11
HaloTag7
HaloTag9
HaloTag11
80 MHz
D
Composite
Total intensity
H2B-HT7
Tomm20-HT9
Lifeact-HT11
HaloTag7
HaloTag9
HaloTag11
80 MHz

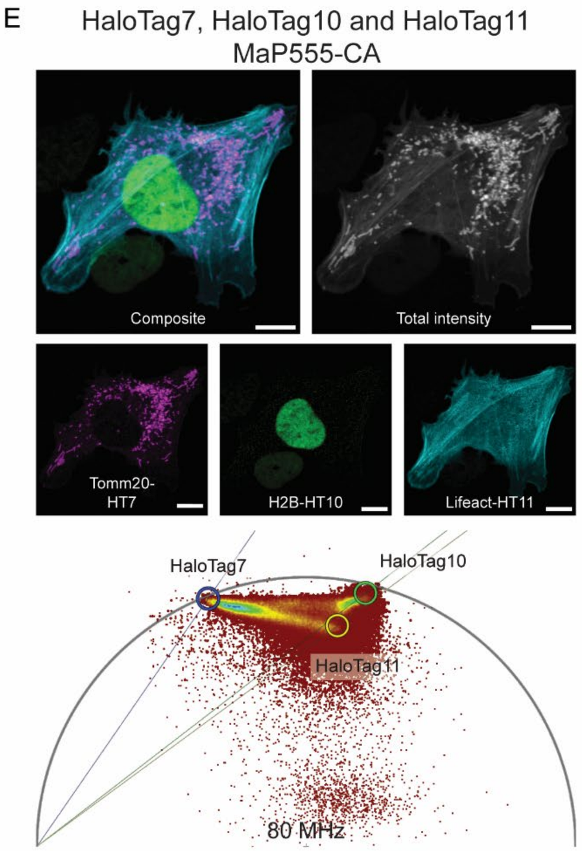
E
HaloTag7, HaloTag10 and HaloTag11
MaP555-CA
Composite
Total intensity
Tomm20-HT7
H2B-HT10
Lifeact-HT11
HaloTag7
HaloTag10
HaloTag11
80 MHz

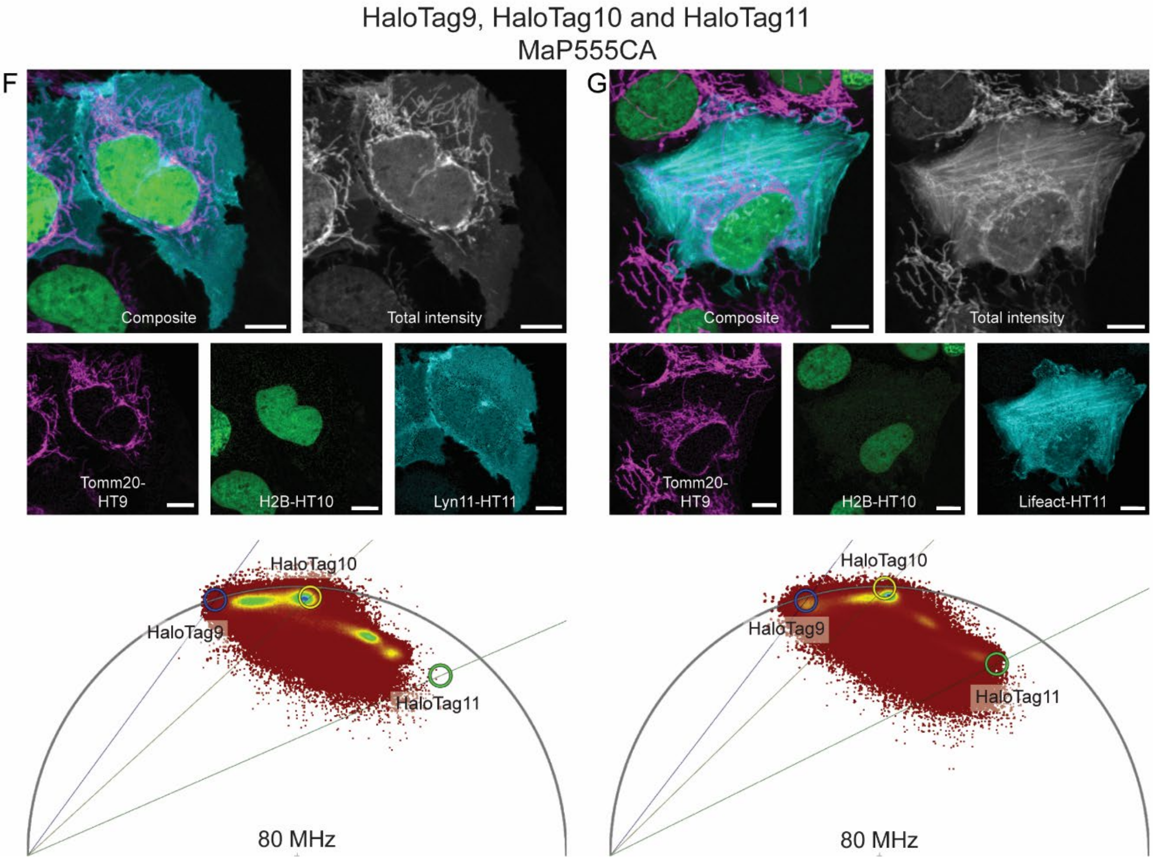
HaloTag9, HaloTag10 and HaloTag11
MaP555CA
F
Composite
Total intensity
Tomm20-HT9
H2B-HT10
Lyn11-HT11
HaloTag10
HaloTag9
HaloTag11
80 MHz
G
Composite
Total intensity
Tomm20-HT9
H2B-HT10
Lifeact-HT11
HaloTag10
HaloTag9
HaloTag11
80 MHz

Supplementary Figure S32: Live-cell fluorescence lifetime multiplexing of three HaloTags labeled with MaP555-CA. **A-B** Combination of HaloTag7, HaloTag9 and HaloTag10. Live U-2 OS cells expressing H2B-HaloTag7 and Tomm20-HaloTag9 (stable) and **A** Lyn11-HaloTag10 **B** Lifeact-HaloTag10 (transient). **C-D** Combination of HaloTag7, HaloTag9 and HaloTag11. Live U-2 OS cells expressing H2B-HaloTag7 and Tomm20-HaloTag9 (stable) and **C** Lyn11-HaloTag11 **D** Lifeact-HaloTag11 (transient). **E** Combination of HaloTag7, HaloTag10 and HaloTag11. Live U-2 OS cells expressing H2B-HaloTag10 (stable) and Lifeact-HaloTag11-T2A-Tomm20-HaloTag7 (transient). **F-G** Combination of HaloTag9, HaloTag10 and HaloTag11. Live U-2 OS cells expressing Tomm20-HaloTag9 and H2B-HaloTag10 (stable) and **F** Lyn11-HaloTag11 **G** Lifeact-HaloTag11 (transient). All cells were labeled with MaP555-CA (1 µM, 3 h). The fluorescence intensity, the composite, the three individual separated species as well as the corresponding wavelet-filtered phasor plot used for separation are given. Species separation was achieved using the phasor approach by positioning the cluster-circles on the phasor plot at the position of the pure species. Scale bars, 10 µm.

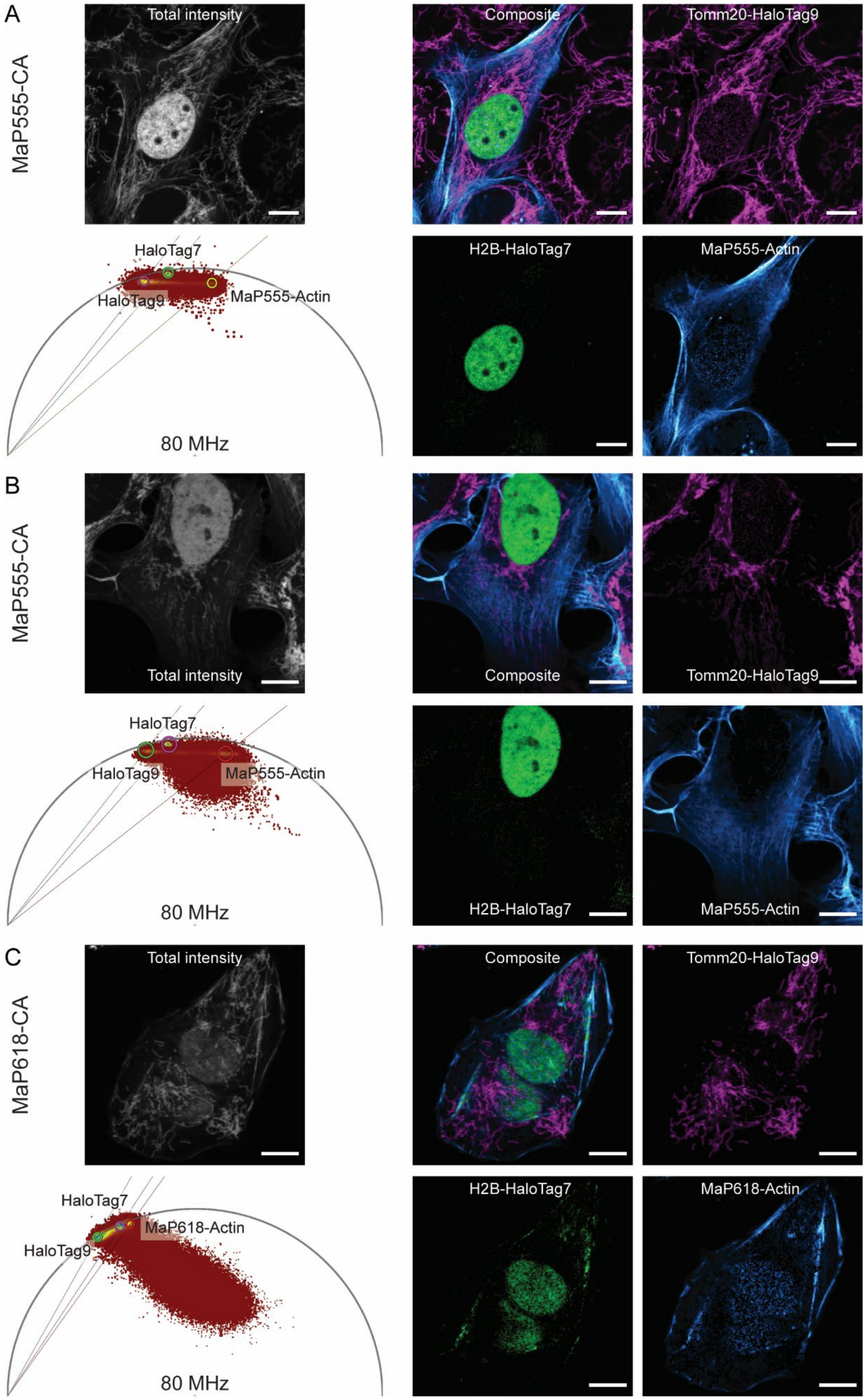
A
MaP555-CA
Total intensity
Composite
Tomm20-HaloTag9
HaloTag7
HaloTag9
MaP555-Actin
80 MHz
H2B-HaloTag7
MaP555-Actin
B
MaP555-CA
Total intensity
Composite
Tomm20-HaloTag9
HaloTag7
HaloTag9
MaP555-Actin
80 MHz
H2B-HaloTag7
MaP555-Actin
C
MaP618-CA
Total intensity
Composite
Tomm20-HaloTag9
HaloTag7
MaP618-Actin
HaloTag9
80 MHz
H2B-HaloTag7
MaP618-Actin

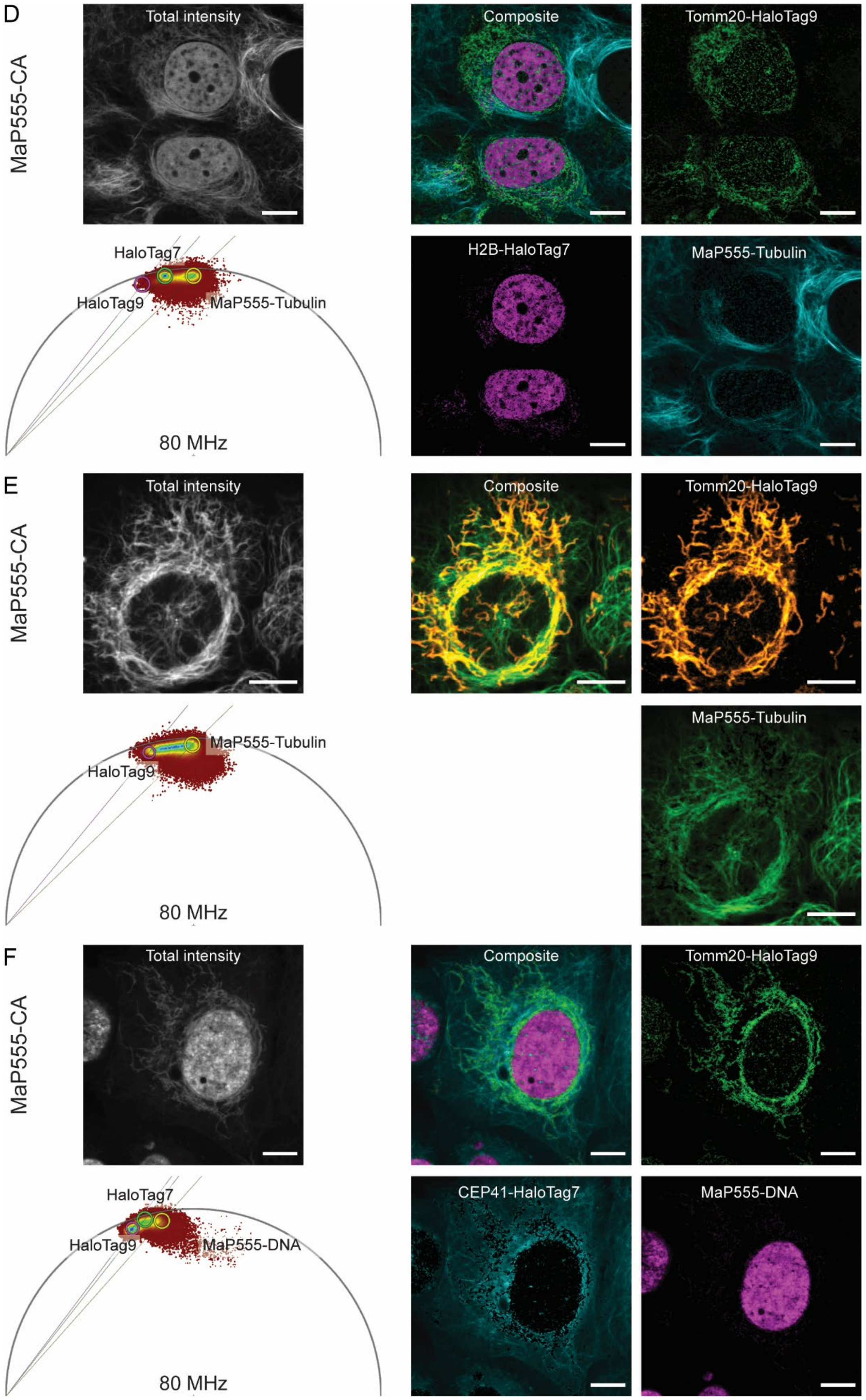
D
MaP555-CA
Total intensity
Composite
Tomm20-HaloTag9
HaloTag7
HaloTag9
MaP555-Tubulin
80 MHz
H2B-HaloTag7
MaP555-Tubulin
E
MaP555-CA
Total intensity
Composite
Tomm20-HaloTag9
MaP555-Tubulin
HaloTag9
80 MHz
MaP555-Tubulin
F
MaP555-CA
Total intensity
Composite
Tomm20-HaloTag9
HaloTag7
HaloTag9
MaP555-DNA
80 MHz
CEP41-HaloTag7
MaP555-DNA

Supplementary Figure S33: Live-cell fluorescence lifetime multiplexing of two HaloTag variants and one non-covalent probe. **A-B** Live U-2 OS cells expressing Tomm20-HaloTag9 (stable) and H2B-HaloTag7 (transient) were labeled with MaP555-CA (1 µM, 3 h) and MaP555-Actin (2 µM, 3 h). The three structures can be clearly separated. **C** Live U-2 OS cells expressing Tomm20-HaloTag9 (stable) and H2B-HaloTag7 (transient) were labeled with MaP618-CA (1 µM, 3 h0 and MaP618-Actin (2 µM, 3 h). Separation was more challenging as the fluorescence lifetime of MaP618-Actin was very similar to HaloTag7-MaP618 and the three clusters were lying almost on a line and not forming a triangle in the phasor space. **D** U-2 OS cells expressing Tomm20-HaloTag9 (stable) and H2B-HaloTag7 (transient) were labeled with MaP555-CA (1 µM, 3 h) as well as MaP555-Tubulin (2 µM, 3 h). MaP555-Tubulin showed a fluorescence lifetime of 2.0 ns and can therefore be separated from the fluorescence lifetimes of MaP555-CA. However, the separation from HaloTag9 (2.6 ns) is performed more easily than from HaloTag7 (2.3 ns). **E** U-2 OS cells expressing only Tomm20-HaloTag9 labeled with MaP555-CA (1 µM, 3 h) as well as MaP555-Tubulin (2 µM, 3 h), demonstrating that separation of these two species works very well. **F** U-2 OS cells expressing CEP41-HaloTag7 (stable) and Tomm20-HaloTag9 (transient) labeled with MaP555-CA(1 µM, 3 h) and MaP555-DNA (1 µM, 3 h). The fluorescence lifetime of MaP555-DNA (2.7 ns) is very close to the fluorescence lifetime of HaloTag9-MaP555 (2.6 ns) and separation was therefore complex. Similar experiments using MaP618-CA and MaP618-DNA were performed but the images acquired were not separable as the fluorescence lifetime of HaloTag9-MaP618 and MaP618-DNA were too similar. The fluorescence intensity, the composite, the three individual separated species as well as the corresponding wavelet-filtered phasor plot used for separation are given. Species separation was achieved using the phasor approach by positioning the cluster-circles on the phasor plot at the position of the pure species. Scale bars, 10 µm.

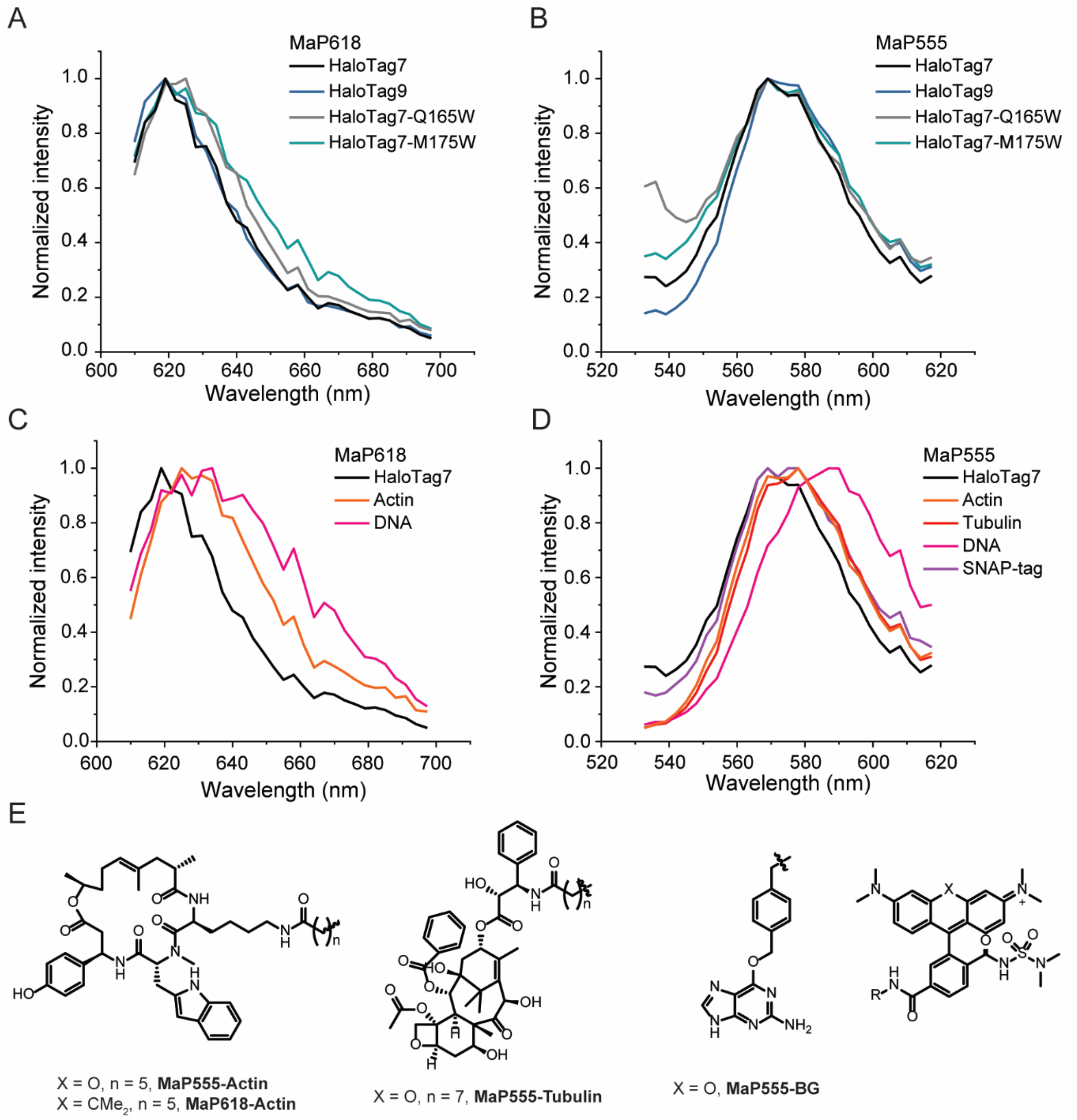

Supplementary Figure S34: Spectral characterization of MaP555 and MaP618 based fluorescent probes. **A-D** Emission spectra of MaP618 (**A**, **C**) and MaP555 (**B**, **D**) fluorophores on different targets as measured by confocal microscopy. The emission spectrum of MaP618-Actin, MaP618-DNA and MaP555-DNA showed a bathochromic shift compared to the other probes. **E** Chemical structures of MaP555-Actin, MaP618-Actin, MaP555-Tubulin, and MaP555-BG. The structures for MaP555-DNA and MaP618-DNA are unknown.

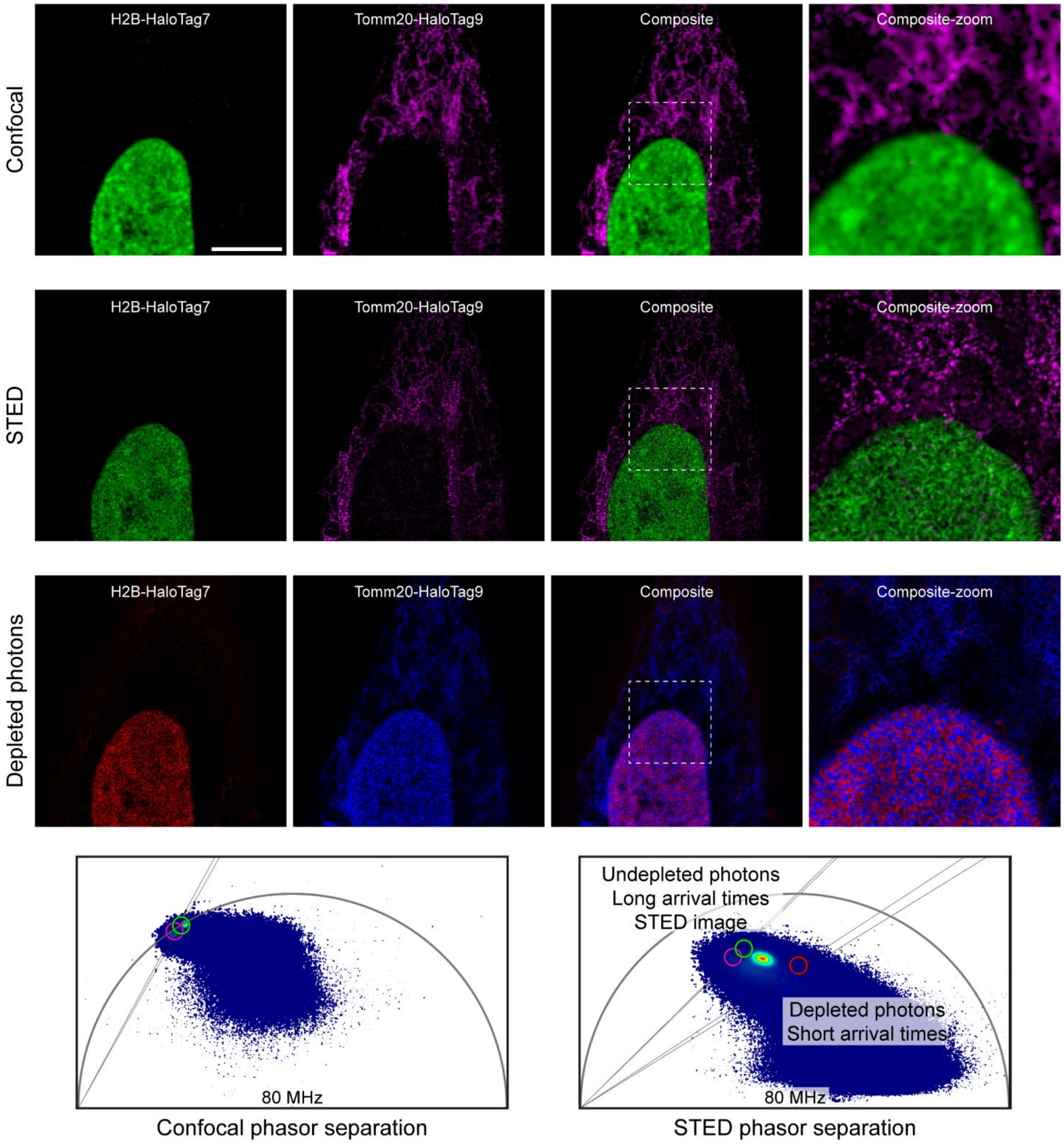
Confocal
STED
Depleted photons
H2B-HaloTag7
Tomm20-HaloTag9
Composite
Composite-zoom
Undepleted photons
Long arrival times
STED image
Depleted photons
Short arrival times
80 MHz
Confocal phasor separation
STED phasor separation

are closer to the (1,0) coordinate, stem from depleted photons and do not show any resolution enhancement. The two individual separated species, the composite, a zoom-in and the corresponding wavelet-filtered phasor plots used for separation both in confocal and STED (magenta and green regions) are given. Additionally, the composite image generated using the depleted photons (blue and red region in the STED phasor plot) is given. Representative images from two independent experiments. Scale bars, 10 µm.

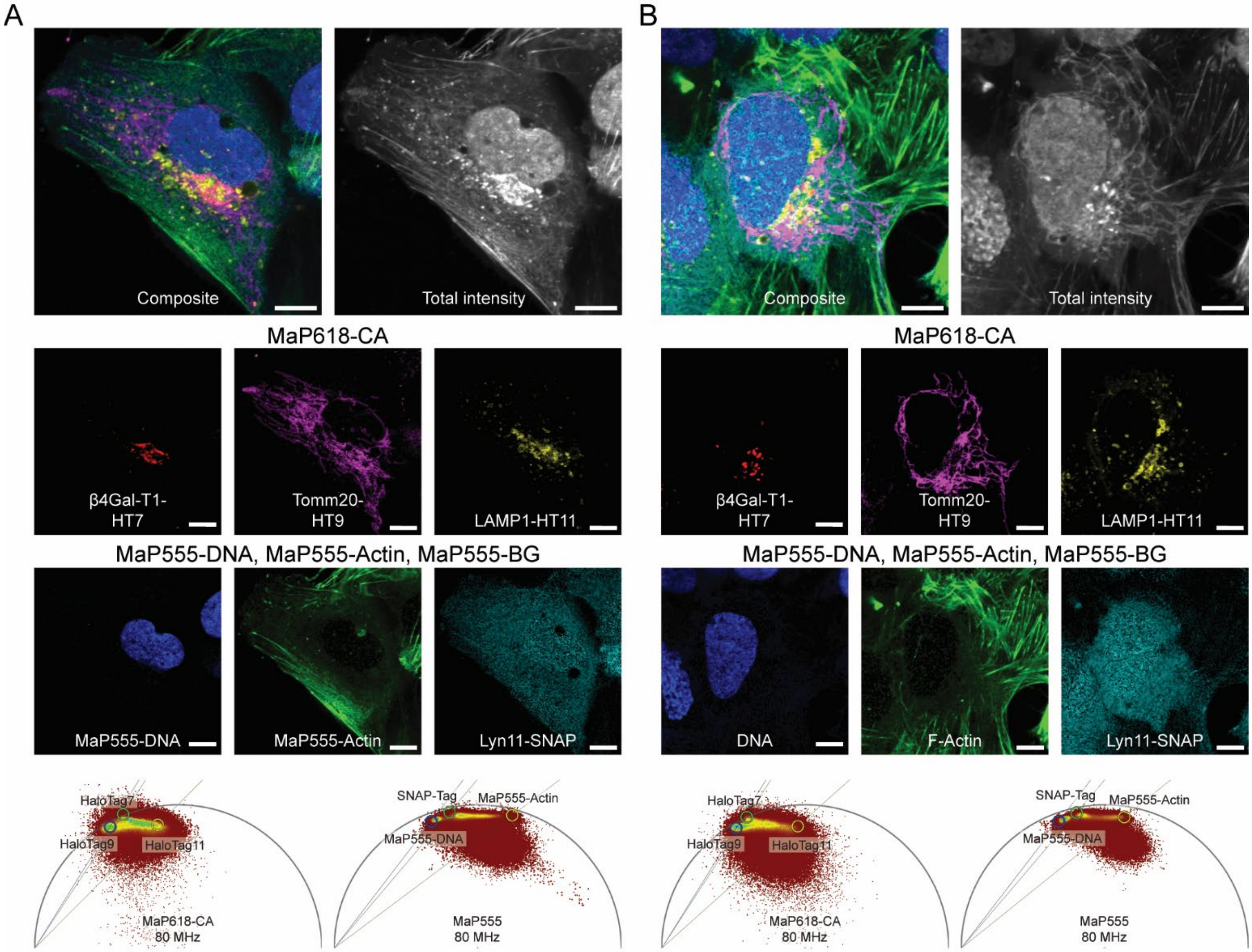

Supplementary Figure S36: Six species live-cell fluorescence lifetime multiplexing combined with spectrally resolved detection. **A-B** Live U-2 OS cells expressing β4Gal-T1-HaloTag7 and Lyn11-SNAP-tag (stable) and Tomm20-HaloTag9 and LAMP1-HaloTag11 (transient) were labeled with MaP618-CA (1 µM, 2 h), MaP555-DNA (1 µM, 1 h), MaP555-Actin (0.5 µM, 1 h), and MaP555-BG (2 µM, 2 h). In each spectral channel a FLIM image was acquired and the three species separated giving access to a six color image. The brightness of the 2x3 species needs to be balanced and only cells with matching expression levels of the four fusion proteins (HaloTags and SNAP-tag) give access to suitable images. The fluorescence intensity, the composite, the six individual separated species as well as the corresponding wavelet-filtered phasor plots used for separation are given. Species separation was achieved using the phasor approach by positioning the cluster-circles on the phasor plot at the position of the pure species. Scale bars, 10 µm.

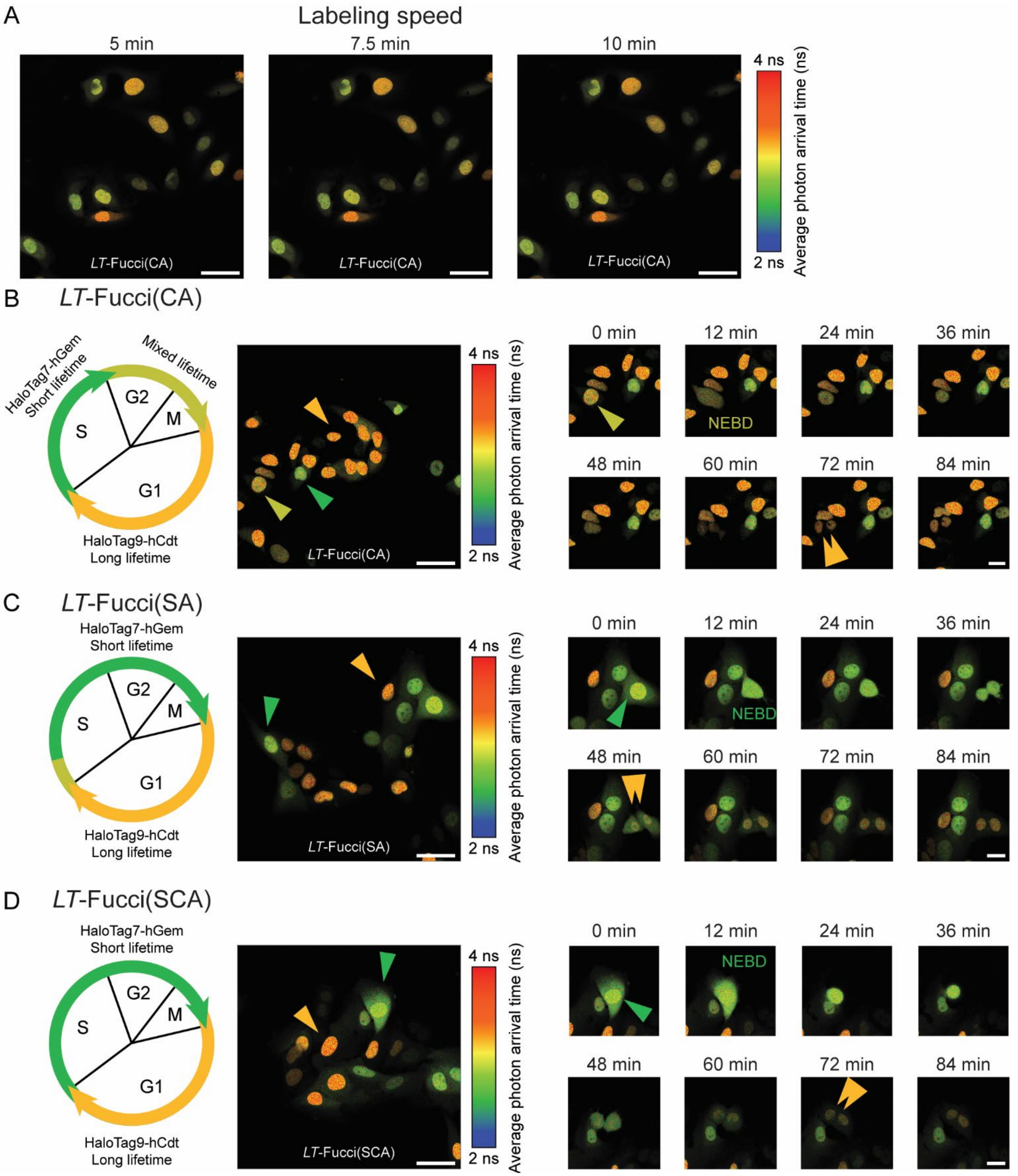

Supplementary Figure S37: Characterization of the *LT*-Fucci biosensors. **A** Assessment of the labeling speed of *LT*-Fucci(CA) with MaP618-CA (1 µM). Within 5 min the biosensor was fully labeled. **B** *LT*-Fucci(CA). **C** *LT*-Fucci(SA). **D** *LT*-Fucci(SCA). For each biosensor a schematic overview of the predominant species present in each phase of the cell cycle is given. For all biosensors HaloTag9-hCdt is present during G1 and the nuclei will therefore present long

average photon arrival times (MaP618-CA: 3.7 ns, orange). During S, G2 and M phase HaloTag7-hGem or a mixture of HaloTag7-hGem and HaloTag9-hCdt is present, resulting in short average photon arrival times (MaP618-CA: 3.1 ns, green) or a gradient of medium average photon arrival times (MaP618-CA: ~3.4 ns, light-green). A representative FastFLIM image of U-2 OS cells stably expressing the lifetime-biosensors labeled with MaP618-CA (1 µM) is given. Colored arrowheads indicate cells in the respective cell cycle phases. Providing 1 µM of MaP618-CA was sufficient to follow the division of cells over 24 h (Supplementary videos 1-3). Representative images of individual cells dividing for each cell line are given. The cells dividing ((light)-green arrowheads), the two daughter cells (orange arrowheads) as well as the moment of nuclear envelope breakdown (NEBD) are indicated. Representative images from three independent experiments. Scale bars, 50 µm and 25 µm.

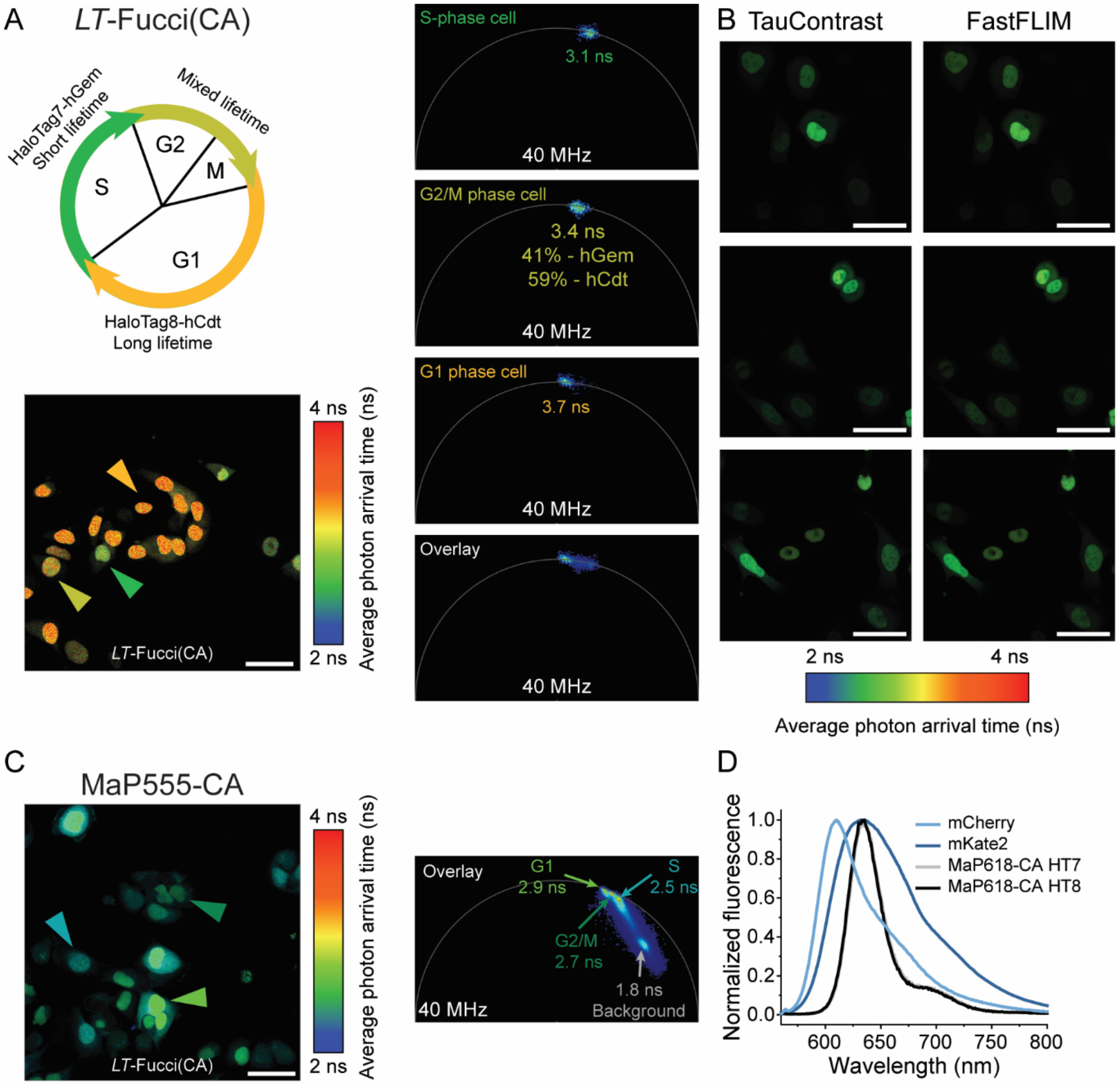

Supplementary Figure S38: Phasor analysis of the *LT*-Fucci(CA) biosensor. **A** Fluorescence lifetime analysis of three nuclei from the image of *LT*-Fucci(CA) (Fig. 3A-D). The average fluorescence lifetime of the nuclei indicated was evaluated using phasor analysis and the respective clusters are given in the phasor plots. In addition, the overlay of all three phasor plots was used to evaluate the percentages of hGem and hCdt present in the G2/M phase cell. As the fluorescence of a G2/M phase cell shows contributions from the two individual components HaloTag7-hGem and HaloTag9-hCdt, the law of linear addition in phasor space can be applied. Scale bars, 50 µm. **B** Lifetime-based biosensing on a confocal microscope without FLIM module. The average photon arrival time of *LT*-Fucci(CA) labeled with MaP618-CA (1 µM) was measured using TauContrast on a STELLARIS microscope[12]. This allows for a direct, lifetime-based readout of the biosensor, without the need for dedicated and complex FLIM instrumentation. TauContrast and FastFLIM images (for comparison) of three field of views are given. Scale bars, 50 µm. **C** *LT*-Fucci(CA) labeled with MaP555-CA (200 nM) generating a different color variant of the biosensor. The fluorescence lifetimes found for the different cell populations by phasor analysis were 2.9 ns (G1), 2.7 ns (G2/M) and 2.5 ns (S). Due to the lower fluorogenicity of MaP555-CA compared to MaP618-CA there was also a larger

contribution of background fluorescence. Scale bars, 50 µm. **D** *In vitro* emission spectra of mCherry, mKate2[13], and MaP618-CA reacted with HaloTag7 or HaloTag9. The spectra of mCherry and mKate2, which compose FUCCI-Red[14], are broader especially toward the far-red region than the spectra of MaP618-CA on HaloTag7 or HaloTag9.

Supplementary Tables

Supplementary Table S1: Summary of HaloTag7 engineering. The mutations that increase (+) or decrease (–) the fluorescence intensity of SiR-CA compared to parental HaloTag7 (first round: $I_{\text{Halo-EGFP}}\pm3\cdot\text{s.d.}_{\text{Halo}}$, second and third round: $I_{\text{Halo-EGFP}}\pm2\cdot\text{s.d.}_{\text{Halo}}$, N = 5 HaloTag7 samples) are given. If more than 20 hits were identified, only a representative number were picked for sequencing and further analysis. The mutations are listed together with the ones that were later validated using purified proteins. The second round was started from the five brightest first round variants (M175L, E170K, Q165H, P174H, and P174L) randomizing the remaining three positions individually. Similarly the third round was performed starting from the two brightest second round variants (Q165H-P174R and Q165H-P174L) randomizing the remaining two positions (E170X and M175X) individually.

	Library	+/–	Hits found (picked)	Mutations (redundancy)	Validated
First round	M175X	+	6 (6)	L(4), M, Q	L
		–	26 (8)	W(3) = HaloTag11, V, E(2), Q , M	W, Q
	V167X	+	6 (5)	V(5)	-
		–	37 (10)	G(2), E(4), D(2), P(2)	P
	E170X	+	1 (1)	K	K, P
		–	7 (7)	T, W(3), P(3)	-
	T148X	+	1 (1)	I	-
		–	2 (2)	V, G	V
	L161X	+	0	-	-
		–	39 (16)	W(7), K, P, T, G(4), A, C	W, G
	Q165X	+	1 (1)	H	H
		–	5 (5)	P, Q, W(3) = HaloTag10	W, P
	T172X	+	0	-	-
		–	48 (18)	E(5), F, K(4), Q(2), R(2), L(2), H, I	E, F, K, Q, R, L, H, I
	F144X	+	0	-	-
		–	11 (11)	S, L(2), F, H(3), N(2), G, I	-
	G171X	+	0	-	-
		–	39 (10)	L(3), H, N, W, T, E, P, M	L,N, W, T, E, M
	P174X	+	4 (4)	H, L, G, Y	H, L, G
		–	23 (12)	Q(2), T(3), V, W(3), P(2), E	W
Second round	E170K, M175X	+	2 (2)	M(2)	-
	E170X, M175L	+	12 (12)	E(5), G, A(2), Q, K, L, V	-
	Q165H, M175X	+	2 (2)	Y, M	-
	Q165X, M175L	+	4 (4)	Q, V, L, A	-
	Q165H, P174X	+	4 (4)	R(2) = HaloTag9, F, L	R, L
	Q165X, P174H	+	2 (2)	V, Q	-
	Q165X, P174L	+	1 (1)	V	-
	P174X, M175L	+	3 (3)	L, H, V	-
	P174H, M175X	+	0	-	-
	P174L, M175X	+	6 (6)	L(3), M(3)	-
	Q165X, E170K	+	0	-	-
	Q165H, E170X	+	2 (2)	V, Q	V
	E170K, P174X	+	0	-	-
	E170X, P174H	+	7 (7)	E(4), K, Q, N	-
	E170X, P174L	+	4 (4)	E(2), Q, A	-
Third round	Q165H, P174R, M175X	+	1 (1)	V	-
	Q165H, E170X, P174R	+	5 (5)	G(2), V, T, A	V, A
	Q165H, P174L, M175X	+	3 (3)	L, V(2)	-
	Q165H, E170X, P174L		10 (10)	E(5), H, V, G, A, Q	-

Supplementary Table S2: Validation of the engineered variants. SiR changes in fluorescence intensity from the variant-EGFP assay are given compared to the parental variant ($\Delta I_{\text{Par-EGFP}} = (I_{\text{var}} - I_{\text{par}})\cdot I_{\text{par}}^{-1}$, $N = 3$ samples) or to HaloTag7 ($\Delta I_{\text{HT7-EGFP}} = (I_{\text{var}} - I_{\text{HT7}})\cdot I_{\text{HT7}}^{-1}$, $N = 3$ samples). Selection criteria: (+) $\Delta I_{\text{HT7-EGFP}} > 23\%$; (–) $\Delta I_{\text{HT7-EGFP}} > 40\%$ and all W bearing variants. * Variants selected for further analysis. s.e.m.: standard error of the mean.

Round	Variant	$\Delta I_{\text{Par-EGFP}}$ [%]	s.e.m. [%]	$\Delta I_{\text{HT7-EGFP}}$ [%]	s.e.m. [%]
First round (+)	M175L*	-	-	31	7
	E170K*	-	-	26	5
	E170P	-	-	21	5
	Q165H*	-	-	24	5
	P174H*	-	-	27	5
	P174L*	-	-	43	11
	P174G	-	-	15	5
First round (–)	M175W* = HaloTag11	-	-	−49	3
	M175Q	-	-	−8	2
	V167P*	-	-	−61	1
	T148V	-	-	−22	3
	L161W*	-	-	−51	3
	L161G	-	-	−19	3
	Q165P	-	-	−29	5
	Q165W* = HaloTag10	-	-	−36	3
	T172E	-	-	−13	2
	T172F*	-	-	−44	2
	T172K*	-	-	−44	5
	T172Q	-	-	−37	4
	T172L	-	-	−14	2
	T172H	-	-	−39	2
	T172I	-	-	−21	2
	T172R*	-	-	−79	1
	G171L	-	-	−10	3
	G171N	-	-	−15	3
	G171W*	-	-	−51	2
	G171T	-	-	−32	2
	G171E	-	-	−20	3
	G171M	-	-	−33	2
	P174W*	-	-	−67	2
Second round	Q165H-P174R* = HaloTag9	32	4	42	7
	Q165H-P174L*	19	3	28	6
	E170V, Q165H	10	3	10	3
Third round	Q165H-E170V-P174R	5	3	10	3
	Q165H-E170A-P174R	12	3	17	2

Supplementary Table S3: Characterization of different HaloTag variants. Comparison of SiR-CA fluorescence intensity changes of all selected variants without EGFP marker (Δ*I*, *N* = 4 replicates each *N* = 4 samples, * *N* = 3 replicates each *N* = 4 samples) as well as the variants' labeling kinetics characterization. The apparent second order rate constant k_{app} measured by either a plate reader or stopped flow, the maximal fluorescence polarization value reached y_0, and the dissociation constant K_D are given (*N* = 3 samples, stop flow *N* = 3 curves from 14 measurements). In addition, the melting temperature (*N* = 2 samples) is given. s.d.: standard deviation. ND not determined.

		Plate reader						Stop flow				Thermostability	
	**Variant**	**Δ*I* [%]**	**s.d. [%]**	**k_{app} [s^{-1} M^{-1}]**	**s.d. [s^{-1} M^{-1}]**	**y_0 [mP]**	**s.d. [mP]**	**K_D [µM]**	**s.d. [µM]**	**k_{app} [s^{-1} M^{-1}]**	**s.d. [s^{-1} M^{-1}]**	**T_m [°C]**	**s.d. [°C]**
**Brighter variants**	**Q165H**	10	6	$12.9 \cdot 10^6$	$0.4 \cdot 10^6$	285.9	0.4	ND	ND	ND	ND	ND	ND
	**E170K**	12	5	$4.4 \cdot 10^6$	$0.1 \cdot 10^6$	288.8	0.7	ND	ND	ND	ND	ND	ND
	**P174H**	16.8	2.0	$7.7 \cdot 10^6$	$0.2 \cdot 10^6$	332.6	0.6	ND	ND	ND	ND	ND	ND
	**P174L**	16	5	$5.7 \cdot 10^6$	$0.1 \cdot 10^6$	289.6	0.5	ND	ND	ND	ND	ND	ND
	**M175L**	9	3	$10.9 \cdot 10^6$	$0.4 \cdot 10^6$	308.9	0.8	ND	ND	ND	ND	ND	ND
	**Q165H-P174L**	15	4	$9.2 \cdot 10^6$	$0.1 \cdot 10^6$	272.1	0.3	ND	ND	ND	ND	ND	ND
	**HaloTag9**	19.9	0.5	$10.1 \cdot 10^6$	$0.3 \cdot 10^6$	271.6	0.5	0.123	0.024	$4.4 \cdot 10^7$	$0.8 \cdot 10^7$	60.3	0.1
	**HaloTag7**	0	-	$9.3 \cdot 10^6$	$0.1 \cdot 10^6$	300.7	0.3	0.23	0.07	$2.4 \cdot 10^7$	$0.7 \cdot 10^7$	61.0	0.4
**Dimmer variants**	**L161W***	−53.3	2.4	$5.4 \cdot 10^6$	$0.4 \cdot 10^6$	325.9	2.0	ND	ND	ND	ND	ND	ND
	**Q165W***	−52.9	1.2	$11.6 \cdot 10^6$	$0.1 \cdot 10^6$	358.5	0.2	ND	ND	ND	ND	58.0	0.1
	**G171W***	−76.51	0.09	$3.12 \cdot 10^6$	$0.07 \cdot 10^6$	329.4	1.0	ND	ND	ND	ND	ND	ND
	**P174W***	−73.1	0.4	$1.16 \cdot 10^6$	$0.02 \cdot 10^6$	329.6	1.4	ND	ND	ND	ND	ND	ND
	**M175W***	−44.8	1.0	$7.3 \cdot 10^6$	$0.3 \cdot 10^6$	339.8	1.0	ND	ND	ND	ND	58.7	0.2

Supplementary Table S4: Fluorophores used for characterization of the HaloTag variants. Their chemical structures, names, excitation and emission maxima, the settings used during plate reader assays (Ex_{used}, Em_{used}, and BW = bandwidth), as well as a reference, the vendor, or the distributor are given. R = CA. [a]HaloTag ligand; [b]NHS ester purchases and coupled to CA-NH_2 – see methods.

#	Structure	Name	Ex_{max} Ex_{used}/BW [nm]	Em_{max} Em_{used}/BW [nm]	Reference, Vendor or Distributor
**1**	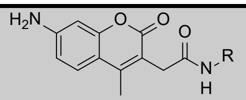	Coumarin-CA	353<br>400/25	434<br>485/20	Promega
**2**	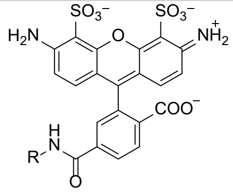	Alexa 488-CA	494<br>490/25	517<br>595/35	Promega
**3**	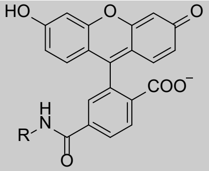	Fluorescein-CA	492<br>490/25	525<br>595/35	[15]
**4**	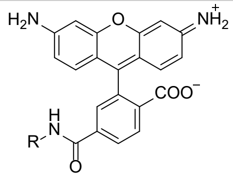	R110-CA	502<br>490/25	527<br>595/35	Promega
**5**	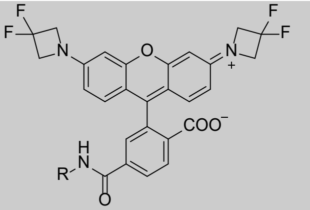	JF_{525}-CA	525<br>490/25	549<br>595/35	[16]
**6**	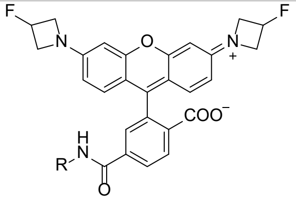	JF_{536}-CA	536<br>490/25	560 595/35	[16]
**7**	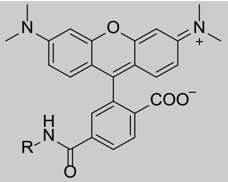	TMR-CA	548<br>535/25	572<br>595/35	[4]
**8**	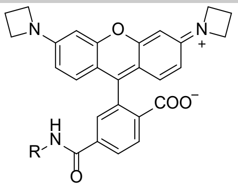	JF_{549}-CA	549<br>535/25	571<br>595/35	[4]

**9**	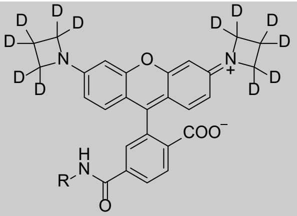	JFX$_{549}$-CA	548<br>535/25	570<br>595/35	[17]<br>Janelia
**10**	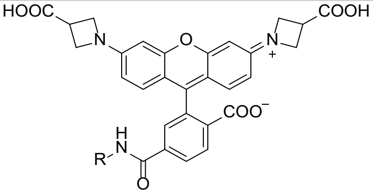	JF$_{549}$i-CA	545<br>535/25	568<br>595/35	[16,18]<br>Janelia
**11**	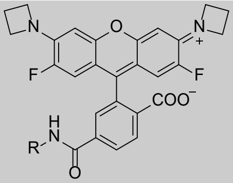	JF$_{552}$-CA	552<br>490/25	575<br>595/35	[19]<br>Janelia
**12**	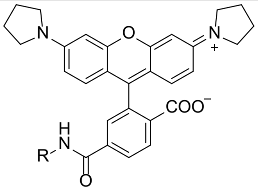	Rhp-CA	553<br>535/25	576<br>595/35	[17]<br>Janelia
**13**	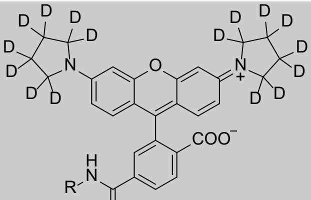	JFX$_{554}$-CA	554<br>535/25	576<br>595/35	[17]<br>Janelia
**14**	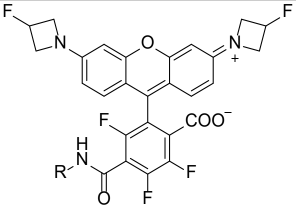	JF$_{559}$-CA	559<br>565/25	579<br>625/35	[20]<br>Janelia
**15**	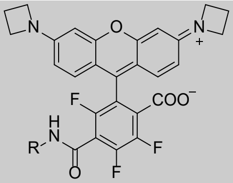	JF$_{571}$-CA	571<br>565/25	590<br>625/35	[20]<br>Janelia
**16**	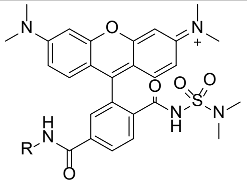	MaP555-CA	558[a]<br>535/25	578[a]<br>595/35	[21]

**17**	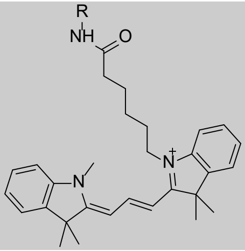	Cyanine3-CA	554<br>490/25	568<br>595/35	See methods
**18**	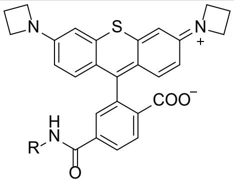	JF_{570}-CA	570<br>565/25	593<br>625/35	[20]<br>Janelia
**19**	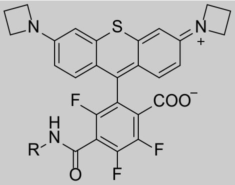	JF_{593}-CA	593<br>565/25	612<br>625/35	[20]<br>Janelia
**20**	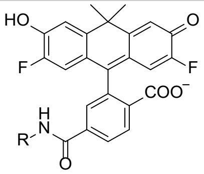	VO-CA	555<br>490/25	581<br>595/35	[22]<br>Janelia
**21**	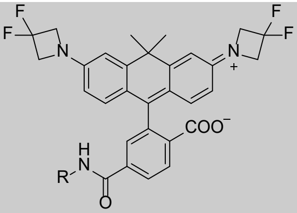	JF_{585}-CA	585<br>565/25	609<br>625/35	[16]<br>Janelia
**22**	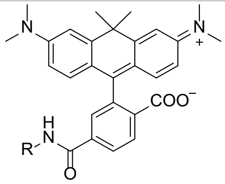	CPY-CA	606<br>610/20	626<br>680/30	[23]
**23**	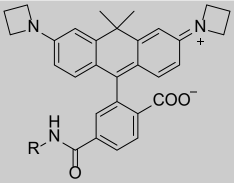	JF_{608}-CA	608<br>610/20	631<br>680/30	[4]
**24**	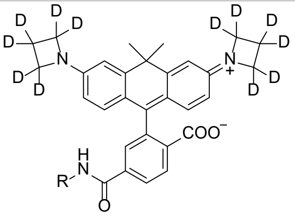	JFX_{608}-CA	608<br>610/20	628<br>680/30	[17]<br>Janelia

**25**	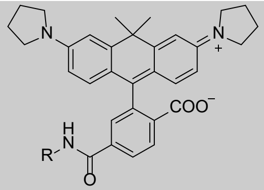	CRhp-CA	613<br>610/20	633<br>680/30	[17]<br>Janelia
**26**	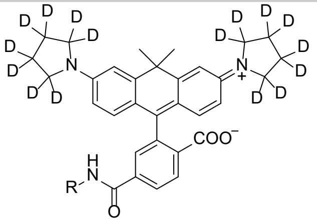	JFX_{612}-CA	612<br>610/20	633<br>680/30	[17]<br>Janelia
**27**	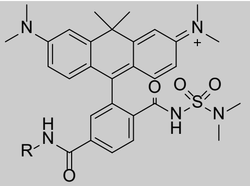	MaP618-CA	618[a]<br>610/20	635[a]<br>680/30	[21]
**28**	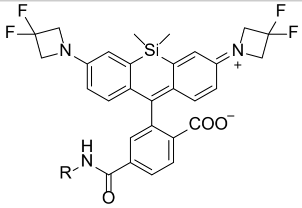	JF_{614}-CA	622[a]<br>620/20	640[a]<br>680/30	[24]<br>Janelia
**29**	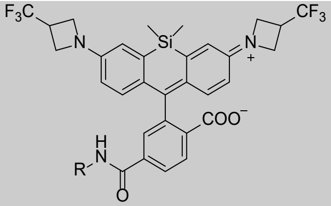	JF_{626}-CA	634[a]<br>620/20	647[a]<br>680/30	[24]<br>Janelia
**30**	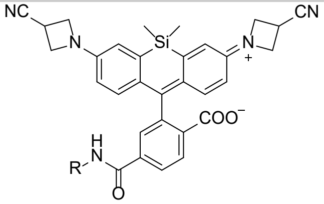	JF_{629}-CA	638[a]<br>620/20	655[a]<br>680/30	[24]<br>Janelia
**31**	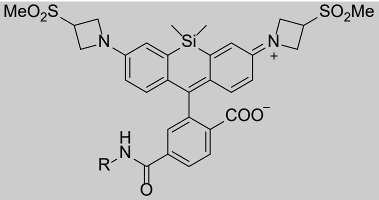	JF_{630}-CA	633 [a]<br>620/20	657[a]<br>680/30	[24]<br>Janelia
**32**	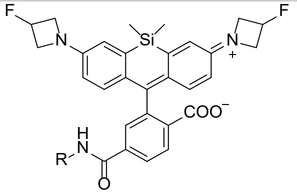	JF_{635}-CA	635<br>620/20	652<br>680/30	[16]<br>Janelia

**33**	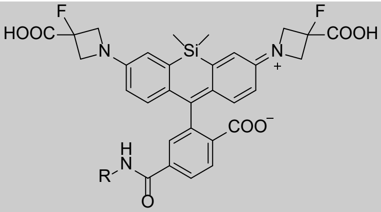	JF$_{635}$i-CA	640[a]<br>620/20	656[a]<br>680/30	[25]<br>Janelia
**34**	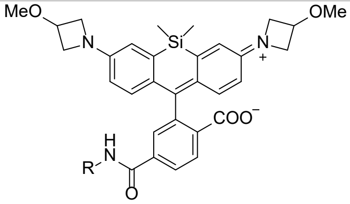	JF$_{639}$-CA	645<br>620/20	658<br>680/30	[24]<br>Janelia
**35**	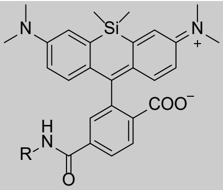	SiR-CA	643<br>620/20	662<br>680/30	[26]
**36**	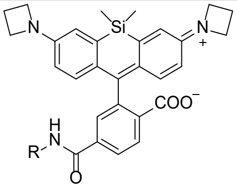	JF$_{646}$-CA	646<br>620/20	664<br>680/30	[4]
**37**	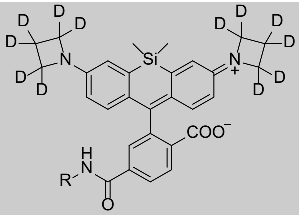	JFX$_{646}$-CA	645<br>620/20	662<br>680/30	[17]<br>Janelia
**38**	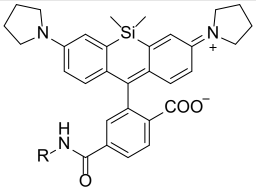	SiRhp-CA	652<br>620/20	668<br>680/30	[17]<br>Janelia
**39**	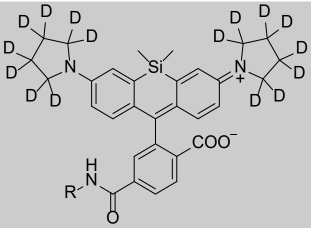	JFX$_{650}$-CA	650<br>620/20	667<br>680/30	[17]<br>Janelia
**40**	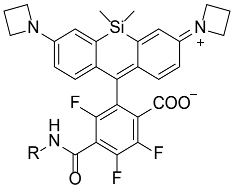	JF$_{669}$-CA	669<br>640/30	682<br>740/30	[20]<br>Janelia

**41**	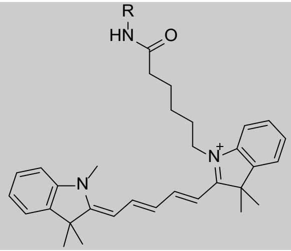	Cyanine5-CA	649<br>640/30	666<br>740/30	See methods
**42**	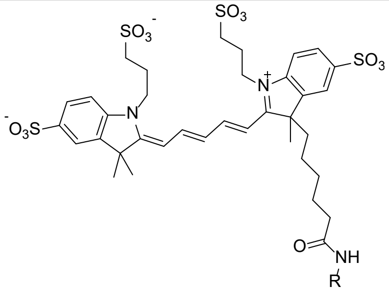	Alexa 647-CA	651<br>640/30	672<br>740/30	ThermoFisher[b]
**43**	No structure given	Alexa 660-CA	663<br>640/30	690<br>740/30	Promega
**44**	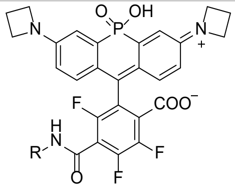	JF_{690}-CA	690<br>640/30	707<br>740/30	[20]<br>Janelia
**45**	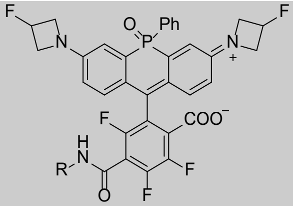	JF_{711}-CA	711<br>640/30	732<br>740/30	[20]<br>Janelia
**46**	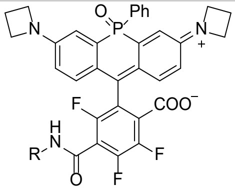	JF_{722}-CA	722<br>640/30	743<br>740/30	[20]<br>Janelia

Supplementary Table S5: Comparison of the fluorescence intensity of different fluorophores on the three HaloTag variants relative to HaloTag7. Fluorescence intensity changes are given in % in comparison to HaloTag7 ($\Delta I = (I_{var} - I_{HT7}) \cdot I_{HT7}^{-1}$, mean±s.d., *N* = 2 replicates each 4 samples for HaloTag9 and mean±s.e.m, *N* = 4 samples from 1 replicate for all other variants; unless otherwise stated). D_{50} values are given for selected fluorophores (mean±s.e.m., *N* = 11 samples). $K_{L\text{-}Z}$ literature values and their reference are given. * Not significant (one sided t-test, α = 5%, DF = 6). NA not applicable, ND not determined.

**Fluorophore**	**HT9**		**HT10 (Q165W)**		**HT11 (M175W)**		**D_{50}**	**$K_{L\text{-}Z}$**	
	Δ*I* [%]	s.d. [%]	Δ*I* [%]	s.e.m. [%]	Δ*I* [%]	s.e.m. [%]			
**Coumarin-CA**	−0.7*	2.5	−14.2	1.8	−16.0	1.6	NA	NA	
**Alexa 488-CA**	−2.7*	1.5	−55.1	2.1	−54.9	1.8			
**Fluorescein-CA**	0.4*	1.6	−13.6	2.7	−0.2*	1.5*	NA	NA	
**R110-CA**	−1*	6	−85.9	0.3	−62.3	0.7			
**JF$_{525}$-CA**	4.1*	2.4	−91.1	0.3	−74.4	0.8	38.67±0.23	0.068	[16]
**JF$_{536}$-CA**	−1.2*	0.3	−91.0	0.6	−49.2	1.8	22.3±0.3	1.0	[16]
**TMR-CA**	9.7	0.6	−73.7	0.8	−40.0	1.9	12.5±0.5		
**JF$_{549}$-CA**	−0.2*	0.5	−77.6	1.3	−35	4		3.5	[16]
**JFX$_{549}$-CA**	−0.1*	0.4	−77.7	0.8	−35	4		3.03	[17]
**JF$_{549}$i-CA**	0.6*	1.0	−86.5	0.4	−30.9	1.9			
**JF$_{552}$-CA**	3.4*	2.5	−91.16	0.23	−39.3	2.1		0.70	[20]
**Rhp-CA**	2.2*	0.5	−75.2	1.3	−28	3		4.50	[17]
**JFX$_{554}$-CA**	0.1*	0.5	−74.9	0.4	−29.3	1.1		4.97	[17]
**JF$_{559}$-CA**	3.6*	1.5	−95.22	0.06	−29.4	1.3		6.22	[20]
**JF$_{571}$-CA**	1.3*	1.1	−91.68	0.20	−16.9	2.4		7.93	[20]
**MaP555-CA**	12.2	1.7	−72	3	−21*	9	55.8±0.3		
**Cy3-CA**	40	10	−20.5	1.4	1.7*	2.1	NA	NA	
**JF$_{570}$-CA**	−0*	6	−74.9	0.6	−32.6	0.8		2.24	[20]
**JF$_{593}$-CA**	−8	4	−76.9	0.5	−19.4	1.6		6.06	[20]
**VO-CA**	42	6	−52.7	1.3	−29.7	1.7	NA	NA	
**JF$_{585}$-CA**	8.6	0.8	−95.62	0.12	−88.3	0.4	56.3±1.5	<0.0001	[16]
**CPY-CA**	11.6	0.4	−46.3	2.3	−36	3	35.0±0.5		
**JF$_{608}$-CA**	−3.4*	1.6	−43.2	1.8	−45.4	1.5		0.091	[16]
**JFX$_{608}$-CA**	−5.8*	0.9	−42.9	0.9	−20.3	1.0		0.14	[17]
**CRhp-CA**	−3.4*	1.0	−18	4	−4*	5*		0.81	[17]
**JFX$_{612}$-CA**	−7.7*	1.0	−28.1	1.6	−5.9*	2.3*		0.88	[17]
**MaP618-CA**	31	4	−38.7	1.3	−17.9	1.9	61±7		
**JF$_{614}$-CA**	102	11	−81.6	0.6	−76.8	0.7	>78		
**JF$_{626}$-CA**	62	14	−74.8	1.5	−56	3			
**JF$_{629}$-CA**	51	8	−82.0	0.3	−79.2	0.3			
**JF$_{630}$-CA**	20	4	−81.3	0.6	−68.4	1.1			
**JF$_{635}$-CA (*N* = 4 rep)**	28.4	2.5	−81.3	0.9	−63.5	1.5	>78	<0.0001	[16]
**JF$_{635}$i-CA**	26.4	1.5	−87.5	0.5	−70.6	0.4			
**JF$_{639}$-CA**	14	6	−65.5	1.0	−41.2	1.9			
**SiR-CA (*N* = 4, 3 rep)**	19.9	0.5	−52.9	1.2	−44.8	0.8	60.5±0.8	0.0034	[27]
**JF$_{646}$-CA (*N* = 4 rep)**	7.1*	0.8	−36	6	−36	6	55.2±0.3	0.0014	[16]
**JFX$_{646}$-CA**	4.5*	0.3	−36.9	1.7	−27.4	2.1		0.0013	[17]
**SiRp-CA**	4.4*	2.5	−18.4	1.8	−32.7	1.7		0.0013	[17]
**JFX$_{650}$-CA**	3.8*	0.6	−19.2	1.2	−32.7	0.6		0.0015	[17]
**JF$_{669}$-CA**	3*	3	−41	3	−14	3		0.262	[20]
**Cy5-CA (*N* = 4 rep)**	−3.2*	1.2	−10.3	2.5	−11.4	2.2	NA	NA	
**Alexa 647-CA (*N* = 4 rep)**	1.9*	1.2	2*	5*	4*	5*	NA	NA	
**Alexa 660-CA (*N* = 4 rep)**	1.2*	1.3	−3*	4*	−15	4	NA	NA	
**JF$_{690}$-CA**	−0.9*	0.6	−66.4	0.8	−16.6	1.8		2.90	[20]
**JF$_{711}$-CA**	−12.1*	1.7	−85.2	0.3	−89.7	0.2		<0.001	[20]
**JF$_{722}$-CA**	13.3	1.0	−77.4	0.9	−70.8	1.0		0.11	[20]

Supplementary Table S6: Quantum yields (ϕ) of fluorophores on HaloTags. Quantum yields of different fluorophores reacted with HaloTag7, HaloTag9, HaloTag10, HaloTag11, or in activity buffer are given (mean±s.e.m., *N* = 3 samples). ND not determined. * Low absorbance.

**Fluorophore**	**HaloTag7 [%]**		**HaloTag9 [%]**		**HaloTag10 [%]**		**HaloTag11 [%]**		**Buffer [%]**	
	**ϕ**	**s.e.m.**	**ϕ**	**s.e.m.**	**ϕ**	**s.e.m.**	**ϕ**	**s.e.m.**	**ϕ**	**s.e.m.**
**JF_{722}-CA**	12.37	0.18	13.07	0.11	3.03	0.07	3.00	0.05	11.50	0.22
**SiR-CA**	55.3	0.5	51.7	0.3	24.63	0.10	28.5	0.09	32.6	0.5
**JF_{646}-CA**	61.83	0.24	60.93	0.17	33.00	0.19	41.57	0.18	61*	3*
**JFX_{646}-CA**	72.53	0.17	70.47	0.05	33.67	0.17	45.67	0.18	62*	3*
**JF_{635}-CA**	67.50	0.17	67.00	0.12	8.90	0.09	28.2	0.4	53*	8*
**JF_{635i}-CA**	65.57	0.11	65.83	0.05	6.37	0.03	19.07	0.07	58.5	0.5
**JF_{614}-CA**	75.63	0.28	73.4	1.0	2.8	0.4	10.6	0.3	ND	ND
**CPY-CA**	71.67	0.22	64.17	0.23	29.53	0.12	27.30	0.12	49.4	1.2
**JF_{608}-CA**	79.47	0.10	77.30	0.17	40.67	0.07	40.33	0.05	66.07	0.10
**JFX_{608}-CA**	70.97	0.10	70.07	0.19	36.03	0.07	39.73	0.03	64.4	0.3
**MaP618-CA**	68.07	0.24	58.50	0.09	26.93	0.07	24.17	0.12	0.7	0.6
**JF_{585}-CA**	85.23	0.07	80.23	0.24	2.89	0.05	10.70	0.05	79.7	1.0
**VO-CA**	75.2	0.4	71.6	0.3	40.3	0.3	63.4	0.7	39.60	0.17
**TMR-CA**	61.2	0.4	56.00	0.08	12.53	0.07	34.23	0.17	43.43	0.10
**JF_{549}-CA**	86.27	0.12	84.60	0.16	16.53	0.21	57.43	0.14	84.80	0.9
**JFX_{549}-CA**	88.57	0.19	86.63	0.24	16.27	0.14	59.20	0.05	88.6	0.3
**MaP555-CA**	61.17	0.24	54.13	0.21	11.47	0.14	34.23	0.20	45*	5*
**JF_{525}-CA**	91.2	0.3	80.30	0.19	5.20	0.09	17.83	0.11	87.7*	2.2*
**Cy3-CA**	18.87	0.05	17.60	0.08	11.77	0.03	17.90	0.12	3.93	0.03

Supplementary Table S7: Extinction coefficients (ε) of fluorophores on HaloTags. Extinction coefficients of different fluorophores reacted with HaloTag7, HaloTag9, HaloTag10, HaloTag11, or in activity buffer with 0.1% SDS (mean±s.e.m., *N* = 6 samples, except HaloTag10-TMR *N* = 5). ND not determined.[1]Larger error given from two independent measurements. [2]Literature values from references [4,21,24,26].

**Fluorophore**	**HaloTag7 [M^{-1} cm^{-1}]**		**HaloTag9 [M^{-1} cm^{-1}]**		**HaloTag10 [M^{-1} cm^{-1}]**		**HaloTag11 [M^{-1} cm^{-1}]**		**Buffer+ SDS [M^{-1} cm^{-1}]**	
	**ε**	**s.e.m.**	**ε**	**s.e.m.**	**ε**	**s.e.m.**	**ε**	**s.e.m.**	**ε**	**s.e.m.**
**SiR-CA**	167,000	11,000[1]	167,000	3,000	153,400	1,900	146,000	5,000	100,000[2]	7,000
**JF_{614}-CA**	7,000[2]	500[1]	19,360	260	8,500	1,100	8,800	1,100	ND	ND
**CPY-CA**	122,000	1,800[1]	124,000	800	125,000	3,000	128,000	4,000	121,000[2]	500
**MaP618-CA**	107,000[2]	1,900[1]	127,900	1,600	111,100	2,600	119,000	5,000	ND	ND
**TMR-CA**	82,000	5,000[1]	82,700	300	92,000	6,000	111,000	9,000	87,000[2]	300
**MaP555-CA**	94,100	1,000[1]	96,500	500	100,000	3,000	119,000	8,000	92,000[2]	1,100

Supplementary Table S8: Intensity weighted fluorescence lifetimes (τ) of fluorophores on HaloTags. Fluorescence lifetimes of different fluorophores reacted with HaloTag7, HaloTag9, HaloTag10, or HaloTag11 as measured by FLIM (mean±s.e.m., *N* = 5-8 FOVs from 3 biological replicates). Unless otherwise stated all mono-exponential fits. ^{2} bi-exponential ^{3} tri-exponential.

**Variant**	**HaloTag7 [ns]**			**HaloTag9 [ns]**			**HaloTag10 Q165W [ns]**			**HaloTag11 M175W [ns]**		
	τ	s.e.m.	*N*	τ	s.e.m.	*N*	τ	s.e.m.	*N*	τ	s.e.m.	*N*
**SiR-CA**	3.26	0.01	6	3.45	0.01	6	1.68^2	0.02	6	2.22^2	0.03	6
**JF_{614}-CA**	3.93	0.02	6	3.94	0.03	8	1.05^3	0.14	5	1.33^2	0.03	5
**CPY-CA**	3.19	0.00	8	3.66	0.02	7	1.93^2	0.09	6	2.51^2	0.03	6
**MaP618-CA**	3.05	0.01	6	3.71	0.01	7	1.62^2	0.01	6	2.14^3	0.03	6
**TMR-CA**	2.41	0.01	6	2.80	0.01	6	1.34^3	0.06	6	1.97^2	0.02	6
**MaP555-CA**	2.33	0.01	7	2.78	0.01	6	1.09^3	0.07	6	1.94^2	0.04	6

Supplementary Table S9: Comparison of fluorophore properties on HaloTag9 and HaloTag7. Changes in fluorescence intensity ($\Delta I = (I_{HT9} - I_{HT7}) \cdot I_{HT7}^{-1}$, N = 2 replicates each 4 samples, unless otherwise stated) together with the changes in quantum yield ($\Delta\phi = (\phi_{HT9} - \phi_{HT7}) \cdot \phi_{HT7}^{-1}$, N = 3 samples), changes in extinction coefficient ($\Delta\varepsilon = (\varepsilon_{HT9} - \varepsilon_{HT7}) \cdot \varepsilon_{HT7}^{-1}$, N = 6 samples), and changes in fluorescence lifetime ($\Delta\tau = (\tau_{HT9} - \tau_{HT7}) \cdot \tau_{HT7}^{-1}$, N = 6–8 samples). ND not determined, * Not significant (one sided t-test, α = 5%, DF = 6, 4, 10, 10-12).

**Fluorophore**	**Δ*I*±s.d. [%]**	**Δϕ±s.e.m. [%]**	**Δε±s.e.m. [%]**	**Δτ±s.e.m. [%]**
**JF_{722}-CA**	13.3±1.0	−5.4±1.6*	ND	ND
**SiR-CA (*N* = 4)**	19.9±0.5	7.0±1.2	−0.1±2.3*	5.8±0.5
**JF_{646}-CA (*N* = 4)**	7.1±0.8*	1.5±0.5*	ND	ND
**JFX_{646}-CA**	4.5±0.3*	2.93±0.25	ND	ND
**JF_{635}-CA (*N* = 4)**	28.4±2.5	0.7±0.3*	ND	ND
**JF_{635}i-CA**	26.4±1.5	−0.4±0.4*	ND	ND
**JF_{614}-CA**	102±11	3.0±1.4*	177±17	0.0±0.8*
**CPY-CA**	11.6±0.4	11.7±0.5	1.3±1.0*	14.5±0.5
**JF_{608}-CA**	−3.4±1.6*	2.8±0.3	ND	ND
**JFX_{608}-CA**	−5.8±0.9*	1.3±0.3	ND	ND
**MaP618-CA**	31±4	16.4±0.4	19.5±1.8	21.4±0.6
**JF_{585}-CA**	8.6±0.8	6.2±0.3	ND	ND
**VO-CA**	42±6	5.1±0.6	ND	ND
**TMR-CA**	9.7±0.6	9.2±0.7	1.5±0.7*	16.1±0.8
**JF_{549}-CA**	−0.2±0.5*	1.97±0.24	ND	ND
**JFX_{549}-CA**	−0.1±0.4*	2.2±0.4	ND	ND
**MaP555-CA**	12.2±1.7	13.0±0.6	2.6±0.8*	19.3±0.6
**JF_{525}-CA**	4.1±2.4*	13.6±0.5	ND	ND
**Cy3-CA**	40±10	7.2±0.6	ND	ND

Supplementary Table S10: Comparison of fluorophore properties on HaloTag10 (HaloTag7-Q165W) and HaloTag7. Changes in fluorescence intensity ($\Delta I = (I_{HT10} - I_{HT7}) \cdot I_{HT7}^{-1}$, N = 4 samples from 1 replicate, unless otherwise stated) together with the changes in quantum yield ($\Delta\phi = (\phi_{HT10} - \phi_{HT7}) \cdot \phi_{HT7}^{-1}$, N = 3 samples), changes in extinction coefficient ($\Delta\varepsilon = (\varepsilon_{HT10} - \varepsilon_{HT7}) \cdot \varepsilon_{HT7}^{-1}$, N = 6 samples), and changes in fluorescence lifetime ($\Delta\tau = (\tau_{HT10} - \tau_{HT7}) \cdot \tau_{HT7}^{-1}$, N = 6–8 samples). ND not determined, * Not significant (one sided t-test, α = 5%, DF = 6, 4, 10, 10-12).

Fluorophore	Δ*I*±s.e.m. [%]	Δϕ±s.e.m. [%]	Δε±s.e.m. [%]	Δτ±s.e.m. [%]
**JF_{722}-CA**	−77.4±0.9	−76.8±0.6	ND	ND
**SiR-CA**	−52.9±1.2	−52.4±0.4	−8±6*	−48.32±0.23
**JF_{646}-CA**	−36±6	−45.9±0.4	ND	ND
**JFX_{646}-CA**	−36.9±1.7	−52.22±0.24	ND	ND
**JF_{635}-CA**	−81.3±0.9	−86.72±0.14	ND	ND
**JF_{635}i-CA**	−87.5±0.5	−90.33±0.04	ND	ND
**JF_{614}-CA**	−81.6±0.6	−96.1±0.5	21±19	−73±4
**CPY-CA**	−46.3±2.3	−53.97±0.25	2.3±2.7*	−39±3
**JF_{608}-CA**	−43.2±1.8	−47.39±0.15	ND	ND
**JFX_{608}-CA**	−42.9±0.9	−48.57±0.17	ND	ND
**MaP618-CA**	−38.7±1.3	−53.96±0.14	4±3*	−47.0±0.4
**JF_{585}-CA**	−95.62±0.12	−96.51±0.06	ND	ND
**VO-CA**	−52.7±1.3	−43.6±0.5	ND	ND
**TMR-CA**	−73.7±0.8	−77.62±0.13	12±10*	−44.7±2.6
**JF_{549}-CA**	−77.6±1.3	−80.46±0.25	ND	ND
**JFX_{549}-CA**	−77.7±0.8	−81.22±0.17	ND	ND
**MaP555-CA**	−72±3	−78.8±0.3	6±4	−53±3
**JF_{525}-CA**	−91.1±0.3	−93.52±0.12	ND	ND
**Cy3-CA**	−20.5±1.4	−33.1±0.4	ND	ND

Supplementary Table S11: Comparison of fluorophore properties on HaloTag11 (HaloTag7-M175W) and HaloTag7. Changes in fluorescence intensity ($\Delta I = (I_{HT11} - I_{HT7}) \cdot I_{HT7}^{-1}$, N = 4 samples from 1 replicate, unless otherwise stated) together with the changes in quantum yield ($\Delta\phi = (\phi_{HT11} - \phi_{HT7}) \cdot \phi_{HT7}^{-1}$, N = 3 samples), changes in extinction coefficient ($\Delta\varepsilon = (\varepsilon_{HT11} - \varepsilon_{HT7}) \cdot \varepsilon_{HT7}^{-1}$, N = 6 samples), and changes in fluorescence lifetime ($\Delta\tau = (\tau_{HT11} - \tau_{HT7}) \cdot \tau_{HT7}^{-1}$, N = 6–8 samples). ND not determined, * Not significant (one sided t-test, α = 5%, DF = 6, 4, 10, 10-12).

Fluorophore HT11 M175W	Δ*I*±s.e.m. [%]	Δϕ±s.e.m. [%]	Δε±s.e.m. [%]	Δτ±s.e.m. [%]
**JF_{722}-CA**	−70.8±1.0	−77.0±0.4	ND	ND
**SiR-CA**	−44.8±0.8	−44.9±0.4	−13±7*	−32.0±0.8
**JF_{646}-CA**	−36±6	−31.8±0.4	ND	ND
**JFX_{646}-CA**	−27.4±2.1	−35.19±0.26	ND	ND
**JF_{635}-CA**	−63.5±1.5	−58.0±0.7	ND	ND
**JF_{635}i-CA**	−70.6±0.4	−71.04±0.11	ND	ND
**JF_{614}-CA**	−76.8±0.7	−85.6±0.5	25±18	−66.1±0.8
**CPY-CA**	−36±3	−57.45±0.25	5±3	−21.3±0.9
**JF_{608}-CA**	−45.4±1.5	−47.82±0.13	ND	ND
**JFX_{608}-CA**	−20.3±1.0	−43.29±0.16	ND	ND
**MaP618-CA**	−17.9±1.9	−58.69±0.21	11±5	−30.0±0.9
**JF_{585}-CA**	−88.3±0.4	−86.66±0.07	ND	ND
**VO-CA**	−29.7±1.7	−11.4±1.1	ND	ND
**TMR-CA**	−40.0±1.9	−38.9±0.3	37±13	−18.4±0.8
**JF_{549}-CA**	−35±4	−32.11±0.21	ND	ND
**JFX_{549}-CA**	−35±4	−31.67±0.20	ND	ND
**MaP555-CA**	−21±9*	−36.8±0.4	27±9	−16.6±1.6
**JF_{525}-CA**	−74.4±0.8	−77.79±0.15	ND	ND
**Cy3-CA**	−1.7±2.1	1.7±0.9*	ND	ND

Supplementary Table S12: Structural analysis of TMR-labeled HaloTags. The following parameters are given: the (average) root-mean-square deviation of the alpha carbons of ($RMSD_{\alpha C}$), the (average) root-mean square displacement of the xanthene core ($RMSD_{xanth}$) and the rhodamine excluding the amide bond ($RMSD_{rhod}$), the (average) root-mean square displacement of three residues of the neighboring monomer (W228, S232, and P233) ($RMSD_{pack}$) comparing HaloTag9 with HaloTag7, the (average) dihedral angle between the xanthene and the appended aromatic ring ($\varphi_{Ar\text{-}Ar}$), the (average) tilt angle of the C9–C7'a bond out of the xanthene plane (γ, Supplementary Fig. S8). For HaloTag10 chain C was not taken into account as two TMR conformations were modeled. Mean±s.d.. [a] Conformation 1. [b] Conformation 2 (Supplementary Fig. S1). NA not applicable.

Variant	Monomers	$RMSD_{\alpha C}$ [Å]	$RMSD_{xanth}$ [Å]	$RMSD_{rhod}$ [Å]	$RMSD_{pack}$ [Å]	$\varphi_{Ar\text{-}Ar}$ [°]	γ [°]
**HaloTag7**	1	-	-	-	-	119.0	8.7
**HaloTag9**	2	0.204±0.015	1.82±0.16	1.57±0.14	0.85±0.13	122.32±0.21	4.45±0.11
**HaloTag10**	6	0.220±0.017	4.3±1.7	3.6±1.4	NA	111±4	3.9±1.2
**HaloTag11**	4	0.278±0.020	7.96±0.12	6.68±0.10	NA	−110.5±1.6	2.85±0.28
**6U32**	1	0.266	-	-	-	104.4[a] 100.9[b]	15.2[a] 1.3[b]
**TMR open**	-	-	-	-	-	94.3	5.6
**TMR closed**	-	-	-	-	-	115.1	37.8

Supplementary Table S13: Comparison of the packing interface in HaloTag7-TMR and HaloTag9-TMR. Distances between different amino acid residues and TMR at the interface between two proteins as indicated in Supplementary Fig. S1 and S6. The lengths of the hydrogen bonds COO^-–W228(NH) and COO^-–S232(OH) as well as the distance from the xanthene core to S232 barley change between HaloTag7 and HaloTag9. The distances between the amino acids W228 and P233 and the xanthene core however changed going from HaloTag7 to HaloTag9. Mean±s.d., *N* = 2 monomers.

Variant	COO^-–W228(NH) [Å]	COO^-–S232(OH) [Å]	Xanthene–W228(Cδ) [Å]	Xanthene–S232(Cα) [Å]	Xanthene–P233(Cδ) [Å]
**HaloTag9**	3.0±0.0	2.8±0.0	3.7±0.0	4.3±0.0	3.55±0.07
**HaloTag7**	2.9	2.8	3.3	4.3	3.9

Supplementary Table S14. Data collection and refinement statistics for the crystal structure of HaloTag9-TMR, HaloTag10-TMR, and HaloTag11-TMR. Values in parentheses are for the highest resolution shell.

	**HaloTag7-Q165H-P174R-TMR**<br>**HaloTag9**<br>**6ZVY**	**HaloTag7-Q165W-TMR**<br>**HaloTag10**<br>**7PCX**	**HaloTag7-M177W-TMR**<br>**HaloTag11**<br>**7PCW**
**Data collection**			
Space group	*P*1	$P12_11$	*P*1
Unit-cell parameters			
a, b, c (Å)	44.15, 47.34, 78.47	74.96, 149.15, 79.92	44.32, 76.56, 85.76
α, β, γ (°)	97.67, 90.19, 113.54	90.00, 117.97, 90.00	68.40, 79.81, 79.02
Radiation source	PXII-X10SA, SLS	PXII-X10SA, SLS	PXII-X10SA, SLS
Wavelength (Å)	0.99988	1.00000	1.00000
Temperature (K)	100	100	100
Resolution range (Å)	50-1.40 (1.50-1.40)	50-1.40 (1.50-1.40)	50-2.30 (2.40-2.30)
No. of observed reflections	189598 (25609)	1028622 (185418)	116244 (13560)
No. of unique reflections	99388 (14156)	300540 (56041)	43477 (5246)
Multiplicity	1.91 (1.81)	3.42 (3.31)	2.67 (2.58)
Completeness (%)	87.5 (66.7)	98.9 (98.9)	95.6 (96.1)
R_{merge} (%)	2.6 (12.5)	3.1 (20.6)	9.8 (46.7)
$\langle I/\sigma(I) \rangle$	17.82 (5.57)	22.96 (6.09)	8.44 (2.80)
$CC_{1/2}$ (%)[#]	99.9 (97.1)	99.9 (95.7)	99.3 (76.9)
Wilson B (Å^2)	20.83	18.45	35.74
**Refinement**			
Molecules per a.u.	2	6	4
No. of reflections	99379	300480	43471
No. of reflections in test set	4970	15024	2174
Resolution range (Å)	40.40-1.40	39.86-1.40	40.36-2.30
No. of non-hydrogen atoms			
Protein	4730	14237	9406
Ligand/ion	114	356	180
Water	648	1159	125
Total	5492	15752	9711
R (%)	16.21	17.55	19.08
R_{free} (%)	18.00	19.82	24.26
RMS deviations from ideal			
bonds (Å)	0.006	0.006	0.002
angles (°)	0.882	0.910	0.576
B-factors (Å^2)			
Protein	15.59	12.78	29.64
Ligand/ion	16.65	16.97	29.41
Water	26.66	20.35	24.48
Average	16.92	13.43	29.57
Ramachandran statistics (%)			
favored regions	96.2	96.7	96.4
allowed regions	3.8	3.3	3.6
disallowed regions	0	0	0
Clashscore	1.16	1.05	2.67

Supplementary Table S15: Comparison of intensity weighted fluorescence lifetimes (τ) of MaP618-CA and MaP55-CA on HaloTags fused to different protein of interest. Fluorescence lifetimes was measured by FLIM (mean±s.e.m., *N* = 4 FOVs from 2 biological replicates, except cytosol *N* = see Supplementary Table S8). Unless otherwise stated all mono-exponential fits. [2] bi-exponential [3] tri-exponential.

	**Fluorophore**	**HaloTag7 [ns]**		**HaloTag9 [ns]**		**HaloTag10 [ns]**		**HaloTag11 [ns]**	
		τ	**s.e.m.**	τ	**s.e.m.**	τ	**s.e.m.**	τ	**s.e.m.**
**MaP618-CA**	**H2B**	3.13	0.05	3.71	0.01	1.65[2]	0.01	2.27[3]	0.02
	**LMNB1**	3.08	0.01	3.64	0.02	1.66[2]	0.02	2.53[3]	0.06
	**Cytosol**	3.05	0.01	3.71	0.01	1.62[2]	0.01	2.14[3]	0.03
	**NES**	3.12	0.01	3.73	0.01	1.69[2]	0.02	2.28[3]	0.01
	**Tomm20**	3.08	0.01	3.69	0.03	1.76[2]	0.01	2.22[3]	0.01
	**COX8**	3.05	0.02	3.62	0.01	1.69[2]	0.01	2.46[3]	0.04
	**CalR-KDEL**	3.06	0.01	3.71	0.01	1.69[2]	0.02	2.02[3]	0.06
	**β4Gal-T1**	3.03	0.01	3.64	0.02	1.64[2]	0.03	2.24[3]	0.13
	**LAMP1**	3.06	0.01	3.65	0.01	1.66[2]	0.01	2.11[3]	0.04
	**SKL**	3.05	0.01	3.71	0.02	1.61[2]	0.00	2.12[3]	0.01
	**Lyn11**	3.06	0.01	3.66	0.01	1.64[2]	0.02	2.08[3]	0.06
	**Ig-κ-PDGF**	3.00	0.01	3.60	0.02	1.57[2]	0.02	1.78[3]	0.07
	**CEP41**	3.32	0.02	3.51	0.05	2.02[3]	0.01	2.55[3]	0.05
	**Lifeact**	3.08	0.00	3.40	0.19	1.63[2]	0.01	2.12[3]	0.05
	**Average**	3.08	0.02	3.64	0.02	1.68	0.03	2.21	0.06
**MaP555-CA**	**H2B**	2.41	0.01	2.86	0.01	1.40[3]	0.07	1.97[2]	0.01
	**LMNB1**	2.38	0.00	2.77	0.01	1.52[3]	0.03	2.08[2]	0.03
	**Cytosol**	2.33	0.01	2.78	0.01	1.09[3]	0.07	1.94[2]	0.04
	**NES**	2.41[2]	0.01	2.85[2]	0.01	1.35[3]	0.02	1.99[2]	0.01
	**Tomm20**	2.38	0.00	2.81	0.01	1.56[3]	0.02	1.96[2]	0.00
	**COX8**	2.40[2]	0.02	2.77	0.02	1.43[3]	0.05	1.98[2]	0.02
	**CalR-KDEL**	2.36	0.00	2.82	0.01	1.16[3]	0.10	1.88[2]	0.01
	**β4Gal-T1**	2.35	0.03	2.67	0.02	1.34[3]	0.07	2.15[2]	0.15
	**LAMP1**	2.38	0.00	2.78	0.00	1.66[3]	0.18	1.90[2]	0.02
	**SKL**	2.37	0.01	2.81	0.01	1.21[3]	0.04	1.91[2]	0.00
	**Lyn11**	2.37[2]	0.01	2.79[2]	0.01	1.19[3]	0.03	1.90[3]	0.01
	**Ig-κ-PDGF**	2.31[2]	0.01	2.78	0.00	0.87[3]	0.01	1.79[3]	0.02
	**CEP41**	2.51[2]	0.02	2.75[2]	0.03	1.66[3]	0.06	2.16[2]	0.04
	**Lifeact**	2.36[2]	0.01	2.85[2]	0.02	1.26[3]	0.01	1.96[3]	0.01
	**Average**	2.38	0.01	2.79	0.01	1.34	0.06	1.97	0.03

Supplementary Table S16: Brightness comparison of different fluorophores on new HaloTag variants relative to HaloTag7 *in cellulo*. Live-cell confocal microscopy was performed on U-2 OS cells expressing HaloTag7, HaloTag9, HaloTag10, or HaloTag11 co-translationally with EGFP (T2A)[28] labeled with SiR-CA, JF_{635}-CA, JF_{629}-CA, JF_{626}-CA, JF_{614}-CA, CPY-CA, MaP618-CA, TMR-CA, and MaP555-CA. Sums of z-stacks were analyzed with regard to their fluorophore and EGFP fluorescence intensities. Mean±s.e.m., *N* = number of total cells analyzed, from three independent preparations. * Not significant (one sided t-test, α = 5%, DF = 238-250).

Fluorophore	Variant	*N*	Δ*I* [%]	s.e.m. [%]
SiR-CA	HaloTag7	120	0.0	0.7
	HaloTag9 (Q165H and P174R)	121	−0.3*	0.5
	HaloTag10 (Q165W)	120	−80.4	0.5
	HaloTag11 (M175W)	121	−52.8	0.6
JF_{635}-CA	HaloTag7	120	0.0	0.6
	HaloTag9 (Q165H and P174R)	121	12.8	0.6
	HaloTag10 (Q165W)	120	−96.2	0.1
	HaloTag11 (M175W)	120	−79.39	0.13
JF_{629}-CA	HaloTag7	129	0.0	0.6
	HaloTag9 (Q165H and P174R)	123	9.7	0.8
	HaloTag10 (Q165W)	114	−96.16	0.13
	HaloTag11 (M175W)	117	−90.60	0.12
JF_{626}-CA	HaloTag7	121	0.0	0.6
	HaloTag9 (Q165H and P174R)	120	6.2	0.6
	HaloTag10 (Q165W)	120	−96.50	0.10
	HaloTag11 (M175W)	120	−79.84	0.16
JF_{614}-CA	HaloTag7	120	0.0	0.9
	HaloTag9 (Q165H and P174R)	124	25.3	1.0
	HaloTag10 (Q165W)	120	−94.32	0.25
	HaloTag11 (M175W)	120	−88.26	0.20
CPY-CA	HaloTag7	120	0.0	0.6
	HaloTag9 (Q165H and P174R)	120	14.4	1.4
	HaloTag10 (Q165W)	120	−62.9	1.0
	HaloTag11 (M175W)	120	−54.2	0.6
MaP618-CA	HaloTag7	120	0.0	0.7
	HaloTag9 (Q165H and P174R)	120	37.6	0.8
	HaloTag10 (Q165W)	120	−81.0	0.6
	HaloTag11 (M175W)	121	−67.88	0.20
TMR-CA	HaloTag7	120	0.0	0.8
	HaloTag9 (Q165H and P174R)	120	8.2	0.7
	HaloTag10 (Q165W)	120	−84.8	0.4
	HaloTag11 (M175W)	121	−40.3	0.7
MaP555-CA	HaloTag7	121	0.0	0.6
	HaloTag9 (Q165H and P174R)	120	14.8	0.5
	HaloTag10 (Q165W)	120	−90.26	0.22
	HaloTag11 (M175W)	120	−46.7	0.3

Supplementary Table S17: Photostability of HaloTags assessed by confocal microscopy. The difference in normalized fluorescence intensity in comparison to HaloTag7 after 480 photobleaching frames ($\Delta_{bleach} = (I_{var\text{-}norm} - I_{HT7\text{-}norm}) \cdot I_{HT7\text{-}norm}^{-1}$, mean and 95% confidence interval, N = all from two independent preparations) and the difference in normalized fluorescence intensity corrected for the difference in brightness ($\Delta\Delta_{bleach}$, mean and 95% confidence interval (CI)) is given. In addition, laser powers used for z-stack measurements and for bleaching are indicated.

HaloTag9 was less photostable than HaloTag7 but the differences were rather small (−7.3%—28.1%). In addition, the absolute fluorescence signal of JF_{614}-CA and MaP618-CA on HaloTag9 after photobleaching would still be higher than on HaloTag7 ($Corr\Delta_{bleach}>0$). In order to reach the presented Δ_{bleach} for the six fluorophores on HaloTag9 one could measure 1,800 (SiR-CA, TMR-CA, and MaP555-CA) 700 (JF_{614}-CA), 3,800 (CPY-CA), or 2,500 (MaP618-CA) z-stacks. HaloTag10 and HaloTag11 were more photostable than HaloTag7. * Not significant (one sided t-test, α = 5%, DF = 42-49).

**Fluorophore**<br>z-stack power<br>Bleaching power	**Variant**	**N [nuclei]**	**Δ_{bleach} [%]**	**95CI [%]**	**$\Delta\Delta_{bleach}$ [%]**	**$\Delta\Delta$95CI [%]**
**SiR-CA**<br>1.6 µW<br>71.3 µW<br>630 nm	**HaloTag7**	24	0.0	1.5	0.0	2.4
	**HaloTag9 (Q165H and P174R)**	24	−10.2	1.7	−10.5	2.2
	**HaloTag10 (Q165W)**	22	5.0	1.6	−79.5	1.2
	**HaloTag11 (M175W)**	24	16.6	1.6	−44.9	1.7
**JF_{614}-CA**<br>4.5 µW<br>78.0 µW<br>614 nm	**HaloTag7**	24	0.0	1.9	0.0	3.2
	**HaloTag9 (Q165H and P174R)**	24	−7.3	1.6	**16.2**	3.4
	**HaloTag10 (Q165W)**	23	15.1	1.6	−93.5	0.6
	**HaloTag11 (M175W)**	20	11.3	1.7	−86.9	0.5
**CPY-CA**<br>0.8 µW<br>78.0 µW<br>614 nm	**HaloTag7**	25	0.0	1.4	0.0	2.2
	**HaloTag9 (Q165H and P174R)**	23	−11.9	2.1	0.8	3.7
	**HaloTag10 (Q165W)**	24	−1.5*	3.7	−63.5	2.4
	**HaloTag11 (M175W)**	22	15.0	1.9	−47.4	1.8
**MaP618-CA**<br>1.3 µW<br>78.0 µW<br>614 nm	**HaloTag7**	26	0.0	2.4	0.0	3.0
	**HaloTag9 (Q165H and P174R)**	24	−10.0	2.7	**23.9**	4.3
	**HaloTag10 (Q165W)**	24	4.4*	2.4	−80.2	1.3
	**HaloTag11 (M175W)**	25	17.9	2.4	−62.1	1.0
**TMR-CA**<br>0.92 µW<br>42.4 µW<br>555 nm	**HaloTag7**	25	0.0	3.8	0.0	4.4
	**HaloTag9 (Q165H and P174R)**	23	−23.9	3.2	−17.6	3.8
	**HaloTag10 (Q165W)**	22	11.6	3.4	−83.1	1.1
	**HaloTag11 (M175W)**	22	5.6*	3.8	−37.0	2.9
**MaP555-CA**<br>0.92 µW<br>42.4 µW<br>555 nm	**HaloTag7**	26	0.0	1.8	0.0	2.5
	**HaloTag9 (Q165H and P174R)**	24	−28.1	2.6	−17.4	3.2
	**HaloTag10 (Q165W)**	25	16.2	1.9	−88.7	0.6
	**HaloTag11 (M175W)**	24	12.9	1.8	−39.8	1.4

Supplementary Table S18: Plasmids used and generated as well as the stable cell lines derived thereof. * Plasmid published in [29].

Name	Addgene#	Plasmid	Gene	Entry Plasmid(s) Addgene#	Stable cell lines
**pET51b(+)_HaloTag9-EGFP**	-	pET51b(+)	HaloTag9	167266[30]	-
**pET51b(+)_HaloTag9**	169324	pET51b(+)	HaloTag9	167266[30]	-
**pET51b(+)_HaloTag10**	175520	pET51b(+)	HaloTag10	167266[30]	-
**pET51b(+)_HaloTag11**	175519	pET51b(+)	HaloTag11	167266[30]	-
**pCDNA5/FRT/TO_HaloTag7_T2A_EGFP**	169325	pCDNA5/FRT/TO	HaloTag7 and EGFP	135444[29]	U-2 OS Flp-In TREx
**pCDNA5/FRT/TO_HaloTag9_T2A_EGFP**	169326	pCDNA5/FRT/TO	HaloTag9 and EGFP	135444[29]	U-2 OS Flp-In TREx
**pCDNA5/FRT/TO_HaloTag10_T2A_EGFP**		pCDNA5/FRT/TO	HaloTag10 and EGFP	135444[29]	U-2 OS Flp-In TREx
**pCDNA5/FRT/TO_HaloTag11_T2A_EGFP**		pCDNA5/FRT/TO	HaloTag11 and EGFP	135444[29]	U-2 OS Flp-In TREx
**pCDNA5/FRT/TO_CEP41-HaloTag7_T2A_EGFP**	169327	pCDNA5/FRT/TO	CEP41-HaloTag7 and EGFP	135446[29]	-
**pCDNA5/FRT/TO_CEP41-HaloTag9_T2A_EGFP**	169328	pCDNA5/FRT/TO	CEP41-HaloTag9 and EGFP	135446[29]	-
**pCDNA5/FRT/TO_CEP41-HaloTag10_T2A_EGFP**		pCDNA5/FRT/TO	CEP41-HaloTag10 and EGFP	135446[29]	-
**pCDNA5/FRT/TO_CEP41-HaloTag11_T2A_EGFP**		pCDNA5/FRT/TO	CEP41-HaloTag11 and EGFP	135446[29]	-
**pCDNA5/FRT/TO_H2B-HaloTag7_T2A_EGFP***	-	pCDNA5/FRT/TO	H2B-HaloTag7 and EGFP	-	U-2 OS Flp-In TREx
**pCDNA5/FRT/TO_H2B-HaloTag9_T2A_EGFP**	169332	pCDNA5/FRT/TO	H2B-HaloTag9 and EGFP	135444[29]	U-2 OS Flp-In TREx
**pCDNA5/FRT/TO_H2B-HaloTag10_T2A_EGFP**	175521	pCDNA5/FRT/TO	H2B-HaloTag10 and EGFP	135444[29]	-
**pCDNA5/FRT/TO_H2B-HaloTag11_T2A_EGFP**	175522	pCDNA5/FRT/TO	H2B-HaloTag11 and EGFP	135444[29]	-
**pCDNA5/FRT/TO_TOMM20-HaloTag7_T2A_EGFP***	-	pCDNA5/FRT/TO	TOMM20-HaloTag7 and EGFP	-	-
**pCDNA5/FRT/TO_TOMM20-HaloTag9_T2A_EGFP**	169333	pCDNA5/FRT/TO	TOMM20-HaloTag9 and EGFP	135443[29]	-
**pCDNA5/FRT/TO_TOMM20-HaloTag10_T2A_EGFP**		pCDNA5/FRT/TO	TOMM20-HaloTag10 and EGFP	135443[29]	-
**pCDNA5/FRT/TO_TOMM20-HaloTag11_T2A_EGFP**		pCDNA5/FRT/TO	TOMM20-HaloTag11 and EGFP	135443[29]	-
**pCDNA5/FRT/TO_CEP41-HaloTag7***	-	pCDNA5/FRT/TO	CEP41-HaloTag7	-	U-2 OS Flp-In TREx
**pCDNA5/FRT/TO_CEP41-HaloTag9**	169331	pCDNA5/FRT/TO	CEP41-HaloTag9	135446[29]	U-2 OS Flp-In TREx
**pCDNA5/FRT/TO_CEP41-HaloTag10**		pCDNA5/FRT/TO	CEP41-HaloTag10	135446[29]	-
**pCDNA5/FRT/TO_CEP41-HaloTag11**		pCDNA5/FRT/TO	CEP41-HaloTag11	135446[29]	U-2 OS Flp-In TREx
**pCDNA5/FRT/TO_H2B-HaloTag7**	169329	pCDNA5/FRT/TO	H2B-HaloTag7	135444[29]	U-2 OS Flp-In TREx
**pCDNA5/FRT/TO_H2B-HaloTag9**	169334	pCDNA5/FRT/TO	H2B-HaloTag9	135444[29]	U-2 OS Flp-In TREx

**pCDNA5/FRT/TO_H2B-HaloTag10**		pCDNA5/FRT/TO	H2B-HaloTag10	135444[29]	U-2 OS Flp-In TREx
**pCDNA5/FRT/TO_H2B-HaloTag11**		pCDNA5/FRT/TO	H2B-HaloTag11	135444[29]	U-2 OS Flp-In TREx
**pCDNA5/FRT/TO_TOMM20-HaloTag7**	169330	pCDNA5/FRT/TO	TOMM20-HaloTag7	135443[29]	U-2 OS Flp-In TREx
**pCDNA5/FRT/TO_TOMM20-HaloTag9**	169335	pCDNA5/FRT/TO	TOMM20-HaloTag9	135443[29]	U-2 OS Flp-In TREx
**pCDNA5/FRT/TO_TOMM20-HaloTag10**		pCDNA5/FRT/TO	TOMM20-HaloTag10	135443[29]	-
**pCDNA5/FRT/TO_TOMM20-HaloTag11**		pCDNA5/FRT/TO	TOMM20-HaloTag11	135443[29]	U-2 OS Flp-In TREx
**pCDNA5/FRT/TO_HaloTag7-LMNB1**	175528	pCDNA5/FRT/TO	HaloTag7-LMNB1	55069	-
**pCDNA5/FRT/TO_HaloTag9-LMNB1**	175538	pCDNA5/FRT/TO	HaloTag9-LMNB1	HaloTag7-LMNB1	-
**pCDNA5/FRT/TO_HaloTag10-LMNB1**		pCDNA5/FRT/TO	HaloTag10-LMNB1	HaloTag7-LMNB1	-
**pCDNA5/FRT/TO_HaloTag11-LMNB1**		pCDNA5/FRT/TO	HaloTag11-LMNB1	HaloTag7-LMNB1	-
**pCDNA5/FRT/TO_NES-HaloTag7**	175527	pCDNA5/FRT/TO	NES-HaloTag7	101061[31]	-
**pCDNA5/FRT/TO_NES-HaloTag9**	175537	pCDNA5/FRT/TO	NES-HaloTag9	NES-HaloTag7	-
**pCDNA5/FRT/TO_NES-HaloTag10**		pCDNA5/FRT/TO	NES-HaloTag10	NES-HaloTag7	-
**pCDNA5/FRT/TO_NES-HaloTag11**		pCDNA5/FRT/TO	NES-HaloTag11	NES-HaloTag7	-
**pCDNA5/FRT/TO_COX8-HaloTag7**	175529	pCDNA5/FRT/TO	COX8-HaloTag7	113916[32]	-
**pCDNA5/FRT/TO_COX8-HaloTag9**	175539	pCDNA5/FRT/TO	COX8-HaloTag9	COX8-HaloTag7	-
**pCDNA5/FRT/TO_COX8-HaloTag10**		pCDNA5/FRT/TO	COX8-HaloTag10	COX8-HaloTag7	-
**pCDNA5/FRT/TO_COX8-HaloTag11**		pCDNA5/FRT/TO	COX8-HaloTag11	COX8-HaloTag7	-
**pCDNA5/FRT/TO_CalR-HaloTag7-KDEL**	175530	pCDNA5/FRT/TO	CalR-HaloTag7-KDEL	synthetic	-
**pCDNA5/FRT/TO_CalR-HaloTag9-KDEL**	175540	pCDNA5/FRT/TO	CalR-HaloTag9-KDEL	CalR-HaloTag7-KDEL	-
**pCDNA5/FRT/TO_CalR-HaloTag10-KDEL**		pCDNA5/FRT/TO	CalR-HaloTag10-KDEL	CalR-HaloTag7-KDEL	-
**pCDNA5/FRT/TO_CalR-HaloTag11-KDEL**		pCDNA5/FRT/TO	CalR-HaloTag11-KDEL	CalR-HaloTag7-KDEL	-
**pCDNA5/FRT/TO_β4Gal-T1-HaloTag7**	175536	pCDNA5/FRT/TO	β4Gal-T1-HaloTag7	36205[33]	-
**pCDNA5/FRT/TO_β4Gal-T1-HaloTag9**	175546	pCDNA5/FRT/TO	β4Gal-T1-HaloTag9	β4Gal-T1-HaloTag7	-
**pCDNA5/FRT/TO_β4Gal-T1-HaloTag10**		pCDNA5/FRT/TO	β4Gal-T1-HaloTag10	β4Gal-T1-HaloTag7	-
**pCDNA5/FRT/TO_β4Gal-T1-HaloTag11**		pCDNA5/FRT/TO	β4Gal-T1-HaloTag11	β4Gal-T1-HaloTag7	-
**pCDNA5/FRT/TO_LAMP1-HaloTag7**	175531	pCDNA5/FRT/TO	LAMP1-HaloTag7	34831[34]	-
**pCDNA5/FRT/TO_LAMP1-HaloTag9**	175541	pCDNA5/FRT/TO	LAMP1-HaloTag9	LAMP1-HaloTag7	-
**pCDNA5/FRT/TO_LAMP1-HaloTag10**		pCDNA5/FRT/TO	LAMP1-HaloTag10	LAMP1-HaloTag7	-
**pCDNA5/FRT/TO_LAMP1-HaloTag11**		pCDNA5/FRT/TO	LAMP1-HaloTag11	LAMP1-HaloTag7	-
**pCDNA5/FRT/TO_HaloTag7-SKL**	175532	pCDNA5/FRT/TO	HaloTag7-SKL	Synthetic	-
**pCDNA5/FRT/TO_HaloTag9-SKL**	175542	pCDNA5/FRT/TO	HaloTag9-SKL	HaloTag7-SKL	-

**pCDNA5/FRT/TO_HaloTag10-SKL**		pCDNA5/FRT/TO	HaloTag10-SKL	HaloTag7-SKL	-
**pCDNA5/FRT/TO_HaloTag11-SKL**		pCDNA5/FRT/TO	HaloTag11-SKL	HaloTag7-SKL	-
**pCDNA5/FRT/TO_Lyn11-HaloTag7**	175533	pCDNA5/FRT/TO	Lyn11-HaloTag7	Synthetic	-
**pCDNA5/FRT/TO_Lyn11-HaloTag9**	175543	pCDNA5/FRT/TO	Lyn11-HaloTag9	Lyn11-HaloTag7	-
**pCDNA5/FRT/TO_Lyn11-HaloTag10**		pCDNA5/FRT/TO	Lyn11-HaloTag10	Lyn11-HaloTag7	-
**pCDNA5/FRT/TO_Lyn11-HaloTag11**		pCDNA5/FRT/TO	Lyn11-HaloTag11	Lyn11-HaloTag7	-
**pCDNA5/FRT/TO_Ig-κ-HaloTag7-PDGFR**	175534	pCDNA5/FRT/TO	Ig-κ-HaloTag7-PDGFR	[35]	-
**pCDNA5/FRT/TO_Ig-κ-HaloTag9-PDGFR**	175544	pCDNA5/FRT/TO	Ig-κ-HaloTag9-PDGFR	Ig-κ-HaloTag7-PDGFR	-
**pCDNA5/FRT/TO_Ig-κ-HaloTag10-PDGFR**		pCDNA5/FRT/TO	Ig-κ-HaloTag10-PDGFR	Ig-κ-HaloTag7-PDGFR	-
**pCDNA5/FRT/TO_Ig-κ-HaloTag11-PDGFR**		pCDNA5/FRT/TO	Ig-κ-HaloTag11-PDGFR	Ig-κ-HaloTag7-PDGFR	-
**pCDNA5/FRT/TO_Lifeact-HaloTag7**	175535	pCDNA5/FRT/TO	Lifeact-HaloTag7	Synthetic	-
**pCDNA5/FRT/TO_Lifeact-HaloTag9**	175545	pCDNA5/FRT/TO	Lifeact-HaloTag9	Lifeact-HaloTag7	-
**pCDNA5/FRT/TO_Lifeact-HaloTag10**		pCDNA5/FRT/TO	Lifeact-HaloTag10	Lifeact-HaloTag7	-
**pCDNA5/FRT/TO_Lifeact-HaloTag11**		pCDNA5/FRT/TO	Lifeact-HaloTag11	Lifeact-HaloTag7	-
**pCDNA5/FRT/TO_H2B-HaloTag7_T2A_Tomm20-HaloTag9**	175547	pCDNA5/FRT/TO	H2B-HaloTag7 and Tomm20-HaloTag9	135444[29]	U-2 OS Flp-In TREx
**pCDNA5/FRT/TO_H2B-HaloTag10_T2A_Tomm20-HaloTag9**	175525	pCDNA5/FRT/TO	H2B-HaloTag10 and Tomm20-HaloTag9	H2B-HaloTag7 T2A Tomm20-HaloTag9	U-2 OS Flp-In TREx
**pCDNA5/FRT/TO_β4Gal-T1-HaloTag10_T2A_EGFP**		pCDNA5/FRT/TO	β4Gal-T1-HaloTag10 and EGFP	β4Gal-T1-HaloTag10	-
**pCDNA5/FRT/TO_β4Gal-T1-HaloTag11_T2A_EGFP**		pCDNA5/FRT/TO	β4Gal-T1-HaloTag11 and EGFP	β4Gal-T1-HaloTag11	-
**pCDNA5/FRT/TO_Lyn11-HaloTag10_T2A_EGFP**		pCDNA5/FRT/TO	Lyn11-HHaloTag10 and EGFP	Lyn11-HHaloTag10	-
**pCDNA5/FRT/TO_Lyn11-HaloTag11_T2A_EGFP**		pCDNA5/FRT/TO	Lyn11-HHaloTag11 and EGFP	Lyn11-HHaloTag11	-
**pCDNA5/FRT/TO_LAMP1-HaloTag10_T2A_EGFP**		pCDNA5/FRT/TO	LAMP1-HaloTag10 and EGFP	LAMP1-HaloTag10	-
**pCDNA5/FRT/TO_LAMP1-HaloTag11_T2A_EGFP**		pCDNA5/FRT/TO	LAMP1-HaloTag11 and EGFP	LAMP1-HaloTag11	-
**pCDNA5/FRT/TO_Lifeact-HaloTag10_T2A_EGFP**		pCDNA5/FRT/TO	Lifeact-HaloTag10 and EGFP	Lifeact-HaloTag10	-
**pCDNA5/FRT/TO_Lifeact-HaloTag11_T2A_EGFP**		pCDNA5/FRT/TO	Lifeact-HaloTag11 and EGFP	Lifeact-HaloTag11	-
**pCDNA5/FRT/TO_Lifeact-HaloTag11_T2A_Tomm20-HaloTag7**	175526	pCDNA5/FRT/TO	Lifeact-HaloTag11 and Tomm20-HaloTag7	H2B-HaloTag7 T2A Tomm20-HaloTag9 and Lifeact-HaloTag7	-

**pCDNA5/FRT/TO_β4Gal-T1-HaloTag7_T2A_Lyn11-SNAPf-tag**	175524	pCDNA5/FRT/TO	β4Gal-T1-HaloTag7 and Lyn11-SNAPf-tag[30]	β4Gal-T1-HaloTag7 167271[30]	U-2 OS Flp-In TREx
**pCDNA5/FRT/TO_LAMP1-HaloTag11_T2A_ Tomm20-HaloTag9**	175523	pCDNA5/FRT/TO	LAMP1-HaloTag11 and Tomm20-HaloTag9	H2B-HaloTag7 T2A Tomm20-HaloTag9 and LAMP1-HaloTag11	-
**pCDNA5/FRT_Fucci(CA)**	169338	pCDNA5/FRT	HaloTag7-Geminin(1/110) and HaloTag9-Cdt(1-100)Cy-	83841[36] 80007[37]	U-2 OS Flp-In TREx
**pCDNA5/FRT_Fucci(SA)**	169336	pCDNA5/FRT	HaloTag7-Geminin(1/110) and HaloTag9-Cdt(30-120)	83841[36]	U-2 OS Flp-In TREx
**pCDNA5/FRT_Fucci(SCA)**	169337	pCDNA5/FRTF	HaloTag7-Geminin(1/110) and HaloTag9-Cdt(1-100)Cy+	83841[36] 80007[37]	U-2 OS Flp-In TREx
**pCAGGS-Raichu-RhoA-CR**	-	pCAGGS	Raichu-RhoA-CR	40258[38]	-

Supplementary Table S19: Fluorescence microscopy data acquisition parameters. Fluorophores refer to CA analogues unless otherwise stated. *See methods for details.

Image	Label	Ligand	Microscope	Excitation [nm]	Pixel dwell time [µs]	Pinhole [Airy Units]	Objective	Pixel size [nm]	Size [pixels]	Emission [nm]	Comment
**Fig. 1F**	HTx	SiR-CA	SP8-FALCON	631	2.09	1	40x1.10 water	569	512x512	680-700	80 MHz
**Fig. 1F**	HTx	JF_{614}-CA	SP8-FALCON	605	2.09	1	40x1.10 water	569	512x512	640-700	80 MHz
**Fig. 1F**	HTx	CPY-CA	SP8-FALCON	595	2.09	1	40x1.10 water	569	512x512	640-660	80 MHz
**Fig. 1F**	HTx	MaP618-CA	SP8-FALCON	595	2.09	1	40x1.10 water	569	512x512	640-660	80 MHz
**Fig. 1F**	HTx	TMR-CA	SP8-FALCON	550	2.09	1	40x1.10 water	569	512x512	570-600	80 MHz
**Fig. 1F**	HTx	MaP555-CA	SP8-FALCON	550	2.09	1	40x1.10 water	569	512x512	570-600	80 MHz
**Fig. 1G**	HTx	MaP618-CA	SP8-FALCON	595	2.09	1	40x1.10 water	569	512x512	640-660	80 MHz
**Fig. 2B**	CEP41-HT9 and Tomm20-HT11	MaP618-CA	SP8-FALCON	615	5.73	1	40x1.10 water	113	688x688	635-700	80 MHz<br>10 line accumu.
**Fig. 2C**	CEP41-HT9 and H2B-HT11	MaP555-CA	SP8-FALCON	550	6.31	1	40x1.10 water	101	624x624	570-600	80 MHz<br>8 line accumu.
**Fig. 2D**	H2B-HT7,<br>Tomm20-HT9,<br>β4Gal-T1-HT11	MaP618-CA	SP8-FALCON	615	6.08	1	40x1.10 water	112	648x648	635-700	80 MHz<br>10 line accumu.
**Fig. 2E**	β4Gal-T1-HT7,<br>Tomm20-HT9<br>LAMP1-HT11	MaP618-CA	SP8-FALCON	615	6.84	1	40x1.10 water	101	576x576	635-700	80 MHz<br>10 line accumu.
**Fig. 2E**	SNAP-tag	MaP555-BG<br>MaP555-Actin<br>MaP555-DN	SP8-FALCON	550	6.84	1	40x1.10 water	101	576x576	570-600	80 MHz<br>10 line accumu.
**Fig. 3A-D**	*LT*-Fucci(CA)	MaP618-CA	SP8-FALCON	615	1.58	5	40x1.10 water	284	1024x1024	640-700	40 MHz<br>4 line accumu.<br>2 pixel binning<br>24 h every 12 min
**Fig. 3E-F**	*LT*-Fucci(CA)	MaP555-CA	SP8-FALCON	550	7.69	5	40x1.10 water	569	512x512	570-620	40 MHz<br>10 line accumu.

**E3A**	HTx	SiR-CA	SP8	630	3.16	1	20x0.75 dry	1138	512x512	650-700	Sum projection
**E3A**	EGFP	-	SP8	489	3.16	1	20x0.75 dry	1138	512x512	510-530	Sum projection
**E3B**	HTx	JF_{635}-CA	SP8	605	3.16	1	20x0.75 dry	1138	512x512	640-700	Sum projection
**E3B**	EGFP	-	SP8	489	3.16	1	20x0.75 dry	1138	512x512	510-530	Sum projection
**E3C**	HTx	JF_{629}-CA	SP8	605	3.16	1	20x0.75 dry	1138	512x512	640-700	Sum projection
**E3C**	EGFP	-	SP8	489	3.16	1	20x0.75 dry	1138	512x512	510-530	Sum projection
**E3D**	HTx	JF_{626}-CA	SP8	605	3.16	1	20x0.75 dry	1138	512x512	640-700	Sum projection
**E3D**	EGFP	-	SP8	489	3.16	1	20x0.75 dry	1138	512x512	510-530	Sum projection
**E3E**	HTx	JF_{614}-CA	SP8	605	3.16	1	20x0.75 dry	1138	512x512	640-700	Sum projection
**E3E**	EGFP	-	SP8	489	3.16	1	20x0.75 dry	1138	512x512	510-530	Sum projection
**E3F**	HTx	CPY-CA	SP8	595	3.16	1	20x0.75 dry	1138	512x512	640-660	Sum projection
**E3F**	EGFP	-	SP8	489	3.16	1	20x0.75 dry	1138	512x512	510-530	Sum projection
**E3G**	HTx	MaP618-CA	SP8	595	3.16	1	20x0.75 dry	1138	512x512	640-660	Sum projection
**E3G**	EGFP	-	SP8	489	3.16	1	20x0.75 dry	1138	512x512	510-530	Sum projection
**E3H**	HTx	TMR-CA	SP8	550	3.16	1	20x0.75 dry	1138	512x512	580-600	Sum projection
**E3H**	EGFP	-	SP8	489	3.16	1	20x0.75 dry	1138	512x512	510-530	Sum projection
**E3I**	HTx	MaP555-CA	SP8	550	3.16	1	20x0.75 dry	1138	512x512	570-600	Sum projection
**E3I**	EGFP	-	SP8	489	3.16	1	20x0.75 dry	1138	512x512	510-530	Sum projection
**E4B**	β4Gal-T1-HTx	MaP618-CA	SP8-FALCON	615	3.16	1	40x1.10 water	142	512x512	635-700	80 MHz<br>500 photons
**E4B**	NES-HTx	MaP555-CA	SP8-FALCON	550	3.16	1	40x1.10 water	142	512x512	570-600	80 MHz<br>500 photons
**E5A-D**	*LT*-Fucci(CA)<br>Raichu-RhoA-CR	MaP618-CA<br>-	SP8-FALCON	615<br>495	7.69	1	40x1.10 water	162	512x512	640-700<br>510-560	40 MHz<br>10 line accumu.<br>2 pixel binning<br>2.5 h every 5 min
**S11A**	H2B-HTx	MaP618-CA	SP8-FALCON	615	3.16	1	40x1.10 water	190	512x512	635-700	80 MHz<br>500 photons
**S11B**	HTx-LAMB1	MaP618-CA	SP8-FALCON	615	12.00	1	40x1.10 water	111	328x328	635-700	80 MHz<br>500 photons
**S11C**	HTx	MaP618-CA	SP8-FALCON	615	3.16	1	40x1.10 water	142	512x512	635-700	80 MHz<br>500 photons
**S11D**	NES-HTx	MaP618-CA	SP8-FALCON	615	3.16	1	40x1.10 water	142	512x512	635-700	80 MHz<br>500 photons
**S11E**	Tomm20-HTx	MaP618-CA	SP8-FALCON	615	3.16	1	40x1.10 water	114	512x512	635-700	80 MHz<br>500 photons

**S11F**	COX8-HTx	MaP618-CA	SP8-FALCON	615	3.16	1	40x1.10 water	190	512x512	635-700	80 MHz<br>500 photons
**S11G**	CalR-HTx-KDEL	MaP618-CA	SP8-FALCON	615	3.16	1	40x1.10 water	114	512x512	635-700	80 MHz<br>500 photons
**S11H**	β4Gal-T1-HTx	MaP618-CA	SP8-FALCON	615	3.16	1	40x1.10 water	142	512x512	635-700	80 MHz<br>500 photons
**S11I**	LAMP1-HTx	MaP618-CA	SP8-FALCON	615	7.69	1	40x1.10 water	114	512x512	635-700	80 MHz<br>500 photons
**S11J**	HTx-SKL	MaP618-CA	SP8-FALCON	615	3.16	1	40x1.10 water	114	512x512	635-700	80 MHz<br>500 photons
**S11K**	Lyn11-HTx	MaP618-CA	SP8-FALCON	615	3.16	1	40x1.10 water	142	512x512	635-700	80 MHz<br>500 photons
**S11L**	Ig-κ-HTx-PDGFR	MaP618-CA	SP8-FALCON	615	3.16	1	40x1.10 water	142	512x512	635-700	80 MHz<br>500 photons
**S11M**	CEP41-HTx	MaP618-CA	SP8-FALCON	615	7.69	1	40x1.10 water	142	512x512	635-700	80 MHz<br>500 photons
**S11N**	Lifeact-HTx	MaP618-CA	SP8-FALCON	615	3.16	1	40x1.10 water	142	512x512	635-700	80 MHz<br>500 photons
**S12A**	H2B-HTx	MaP555-CA	SP8-FALCON	550	3.16	1	40x1.10 water	190	512x512	570-600	80 MHz<br>500 photons
**S12B**	HTx-LAMB1	MaP555-CA	SP8-FALCON	550	10.93	1	40x1.10 water	101	360x360	570-600	80 MHz<br>500 photons
**S12C**	HTx	MaP555-CA	SP8-FALCON	550	3.16	1	40x1.10 water	190	512x512	570-600	80 MHz<br>500 photons
**S12D**	NES-HTx	MaP555-CA	SP8-FALCON	550	3.16	1	40x1.10 water	142	512x512	570-600	80 MHz<br>500 photons
**S12E**	Tomm20-HTx	MaP555-CA	SP8-FALCON	550	3.16	1	40x1.10 water	114	512x512	570-600	80 MHz<br>500 photons
**S12F**	COX8-HTx	MaP555-CA	SP8-FALCON	550	3.16	1	40x1.10 water	142	512x512	570-600	80 MHz<br>500 photons
**S12G**	CalR-HTx-KDEL	MaP555-CA	SP8-FALCON	550	3.16	1	40x1.10 water	142	512x512	570-600	80 MHz<br>500 photons
**S12H**	β4Gal-T1-HTx	MaP555-CA	SP8-FALCON	550	3.16	1	40x1.10 water	114	512x512	570-600	80 MHz<br>500 photons
**S12I**	LAMP1-HTx	MaP555-CA	SP8-FALCON	550	3.16	1	40x1.10 water	114	512x512	570-600	80 MHz<br>500 photons
**S12J**	HTx-SKL	MaP555-CA	SP8-FALCON	550	3.16	1	40x1.10 water	114	512x512	570-600	80 MHz<br>500 photons

**S12K**	Lyn11-HTx	MaP555-CA	SP8-FALCON	550	3.16	1	40x1.10 water	114	512x512	570-600	80 MHz<br>500 photons
**S12L**	Ig-κ-HTx-PDGFR	MaP555-CA	SP8-FALCON	550	3.16	1	40x1.10 water	114	512x512	570-600	80 MHz<br>500 photons
**S12M**	CEP41-HTx	MaP555-CA	SP8-FALCON	550	7.69	1	40x1.10 water	142	512x512	570-600	80 MHz<br>500 photons
**S12N**	Lifeact-HTx	MaP555-CA	SP8-FALCON	550	3.16	1	40x1.10 water	114	512x512	570-600	80 MHz<br>500 photons
**S13A**	H2B-HTx	SiR-CA	SP8	630	0.86	5	40x1.10 water	284	1024x1024	650-700	Sum projection<br>Bleaching
**S13B**	H2B-HTx	JF_{614}-CA	SP8	614	0.86	5	40x1.10 water	284	1024x1024	630-750	Sum projection<br>Bleaching
**S13C**	H2B-HTx	CPY-CA	SP8	614	0.86	5	40x1.10 water	284	1024x1024	630-750	Sum projection<br>Bleaching
**S13D**	H2B-HTx	MaP618-CA	SP8	614	0.86	5	40x1.10 water	284	1024x1024	630-750	Sum projection<br>Bleaching
**S13E**	H2B-HTx	TMR-CA	SP8	555	0.86	5	40x1.10 water	284	1024x1024	570-620	Sum projection<br>Bleaching
**S13F**	H2B-HTx	MaP555-CA	SP8	555	0.86	5	40x1.10 water	284	1024x1024	570-620	Sum projection<br>Bleaching
**S14A**	Tomm20-HT7 or Tomm20-HT9	SiR-CA	Abberior	640	7.00	1	100x1.40 oil	25	680x680	650-725	2 line accumu.<br>STED 3 frame accumu.
**S14B**	Tomm20-HT7 or Tomm20-HT9	MaP618-CA	Abberior	640	7.00	1	100x1.40 oil	25	680x680	650-725	2 line accumu.<br>STED 3 frame accumu.
**S15A**	Tomm20-HT7	MaP618-CA	Abberior	640	7.00	1	100x1.40 oil	25	680x680	650-725	2 line accumu.<br>STED 3 frame accumu.
**S15B**	Tomm20-HT9	MaP618-CA	Abberior	640	7.00	1	100x1.40 oil	25	680x680	650-725	2 line accumu.<br>STED 3 frame accumu.
**S15C**	CEP41-HT7	MaP618-CA	Abberior	640	12.00	1	100x1.40 oil	25	680x680	650-725	2 line accumu.<br>STED 3 frame accumu.
**S15D**	CEP41-HT9	MaP618-CA	Abberior	640	12.00	1	100x1.40 oil	25	680x680	650-725	2 line accumu.<br>STED 3 frame accumu.

**S15E-F**	CEP41-HT7 or CEP41-HT9	MaP618-CA	Abberior	640	12.00	1	100x1.40 oil	25	680x680	650-725	2 line accumu. STED 3 frame accumu.
**S15G-H**	CEP41-HT7 or CEP41-HT9	MaP618-CA	Abberior	640	12.00	1	100x1.40 oil	25	680x680	650-725	2 line accumu. STED 3 frame accumu. PFA fixed
**S16A**	CEP41-HT7	MaP618-CA	Abberior	-	12.00	1	100x1.40 oil	25	680x680	650-725	2 line accumu. STED 3 frame accumu.
**S16B**	CEP41-HT7	MaP618-CA	Abberior	640	12.00	1	100x1.40 oil	25	680x680	650-725	2 line accumu. STED 3 frame accumu.
**S16C**	CEP41-HT9	MaP618-CA	Abberior	-	12.00	1	100x1.40 oil	25	680x680	650-725	2 line accumu. STED 3 frame accumu.
**S16D**	CEP41-HT9	MaP618-CA	Abberior	640	12.00	1	100x1.40 oil	25	680x680	650-725	2 line accumu. STED 3 frame accumu.
**S16E**	CEP41-HT7	SiR-CA	Abberior	-	12.00	1	100x1.40 oil	25	680x680	650-725	2 line accumu. STED 3 frame accumu.
**S16F**	CEP41-HT7	SiR-CA	Abberior	640	12.00	1	100x1.40 oil	25	680x680	650-725	2 line accumu. STED 3 frame accumu.
**S16G**	CEP41-HT9	SiR-CA	Abberior	-	12.00	1	100x1.40 oil	25	680x680	650-725	2 line accumu. STED 3 frame accumu.
**S16H**	CEP41-HT9	SiR-CA	Abberior	640	12.00	1	100x1.40 oil	25	680x680	650-725	2 line accumu. STED 3 frame accumu.
**S17A**	HT7 or HT9	SiR-CA	SP8-FALCON	630	-	1	40x1.10 water	-	-	650-700	FCS
**S17B**	HT7 or HT9	JF_{614}-CA	SP8-FALCON	615	-	1	40x1.10 water	-	-	630-700	FCS
**S17C**	HT7 or HT9	MaP618-CA	SP8-FALCON	615	-	1	40x1.10 water	-	-	630-700	FCS
**S18A**	CEP41-HT7	MaP618-CA	SP8	595	10.36	1	40x1.10 water	110	760x760	640-660	Sum projection
**S18A**	EGFP	-	SP8	489	10.36	1	40x1.10 water	110	760x760	510-530	Sum projection
**S18B**	CEP41-HT9	MaP618-CA	SP8	595	10.36	1	40x1.10 water	110	760x760	640-660	Sum projection
**S18B**	EGFP	-	SP8	489	10.36	1	40x1.10 water	110	760x760	510-530	Sum projection

**S18C**	CEP41-HT7 or CEP41-HT9	MaP618-CA	SP8	615	5.13	1	40x1.10 water	90	768x768	640-700	Sum projection
**S18C**	EGFP	-	SP8	489	5.13	1	40x1.10 water	90	768x768	510-530	Sum projection
**S19A**	H2B-HT7 and Tomm20-HT9	MaP618-CA	SP8-FALCON	615	21.40	1	40x1.10 water	112	520x520	630-700	80 MHz<br>10 line accumu.
**S19B**	H2B-HT7 and Tomm20-HT10	MaP618-CA	SP8-FALCON	615	3.56	1	40x1.10 water	114	456x456	635-700	80 MHz<br>8 line accumu.
**S19C**	H2B-HT7 and Tomm20-HT11	MaP618-CA	SP8-FALCON	615	3.75	1	40x1.10 water	112	432x432	635-700	80 MHz<br>8 line accumu.
**S19D**	H2B-HT9 and Tomm20-HT10	MaP618-CA	SP8-FALCON	615	2.50	1	40x1.10 water	113	648x648	635-700	80 MHz<br>8 line accumu.
**S19E**	H2B-HT9 and Tomm20-HT11	MaP618-CA	SP8-FALCON	615	3.82	1	40x1.10 water	112	424x424	635-700	80 MHz<br>4 line accumu.
**S19F**	H2B-HT10 and Tomm20-HT11	MaP618-CA	SP8-FALCON	615	3.03	1	40x1.10 water	113	536x536	635-700	80 MHz<br>6 line accumu.
**S20A**	Tomm20-HT7 and H2B-HT9	MaP618-CA	SP8-FALCON	615	22.90	1	40x1.10 water	113	344x344	630-700	80 MHz<br>10 line accumu.
**S20B**	Tomm20-HT7 and H2B-HT10	MaP618-CA	SP8-FALCON	615	4.06	1	40x1.10 water	112	400x400	635-700	80 MHz<br>10 line accumu.
**S20C**	Tomm20-HT7 and H2B-HT11	MaP618-CA	SP8-FALCON	615	3.33	1	40x1.10 water	114	488x488	635-700	80 MHz<br>6 line accumu.
**S20D**	Tomm20-HT9 and H2B-HT10	MaP618-CA	SP8-FALCON	615	3.69	1	40x1.10 water	112	440x440	635-700	80 MHz<br>8 line accumu.
**S20E**	Tomm20-HT9 and H2B-HT11	MaP618-CA	SP8-FALCON	615	2.64	1	40x1.10 water	112	616x616	635-700	80 MHz<br>4 line accumu.
**S20F**	Tomm20-HT10 and H2B-HT11	MaP618-CA	SP8-FALCON	615	2.41	1	40x1.10 water	112	672x672	635-700	80 MHz<br>10 line accumu.
**S21A**	CEP41-HT7 and H2B-HT9	MaP618-CA	SP8-FALCON	615	10.70	1	40x1.10 water	112	368x368	630-700	80 MHz<br>10 line accumu.
**S21B**	CEP41-HT7 and H2B-HT10	MaP618-CA	SP8-FALCON	615	7.58	1	40x1.10 water	112	520x520	635-700	80 MHz<br>4 line accumu.
**S21C**	CEP41-HT7 and H2B-HT11	MaP618-CA	SP8-FALCON	615	8.06	1	40x1.10 water	113	488x488	635-700	80 MHz<br>4 line accumu.
**S21D**	CEP41-HT9 and H2B-HT10	MaP618-CA	SP8-FALCON	615	8.79	1	40x1.10 water	112	448x448	635-700	80 MHz<br>2 line accumu.
**S21E**	CEP41-HT9 and H2B-HT11	MaP618-CA	SP8-FALCON	615	7.58	1	40x1.10 water	113	736x736	635-700	80 MHz<br>4 line accumu.

**S21F**	CEP41-HT11 and H2B-HT10	MaP618-CA	SP8-FALCON	615	11.45	1	40x1.10 water	113	344x344	635-700	80 MHz 10 line accumu.
**S22A**	CEP41-HT7 and Tomm20-HT9	MaP618-CA	SP8-FALCON	615	7.58	1	40x1.10 water	112	448x448	630-700	80 MHz 10 line accumu.
**S22B**	CEP41-HT7 and Tomm20-HT10	MaP618-CA	SP8-FALCON	615	5.59	1	40x1.10 water	113	704x704	635-700	80 MHz 10 line accumu.
**S22C**	CEP41-HT7 and Tomm20-HT11	MaP618-CA	SP8-FALCON	615	8.20	1	40x1.10 water	112	480x480	635-700	80 MHz 10 line accumu.
**S22D**	CEP41-HT9 and Tomm20-HT10	MaP618-CA	SP8-FALCON	615	6.23	1	40x1.10 water	114	632x632	635-700	80 MHz 6 line accumu.
**S22E**	CEP41-HT9 and Tomm20-HT11	MaP618-CA	SP8-FALCON	615	5.73	1	40x1.10 water	113	688x688	635-700	80 MHz 10 line accumu.
**S22F**	CEP41-HT11 and Tomm20-HT10	MaP618-CA	SP8-FALCON	615	8.49	1	40x1.10 water	113	464x464	635-700	80 MHz 10 line accumu.
**S23A**	CEP41-HT9 and H2B-HT7	MaP618-CA	SP8-FALCON	615	8.06	1	40x1.10 water	113	488x488	630-700	80 MHz 6 line accumu.
**S23B**	CEP41-HT9 and Tomm20-HT7	MaP618-CA	SP8-FALCON	615	7.58	1	40x1.10 water	113	520x520	635-700	80 MHz 12 line accumu.
**S23C**	CEP41-HT9 and H2B-HT7	MaP555-CA	SP8-FALCON	550	6.92	1	40x1.10 water	100	568x568	570-620	80 MHz 10 line accumu.
**S24A**	Tomm20-HT7 and H2B-HT9	MaP618-CA	SP8-FALCON	615	22.90	1	40x1.10 water	113	344x344	630-700	40 MHz 10 line accumu.
**S24B**	H2B-HT7 and Tomm20-HT9	MaP618-CA	SP8-FALCON	615	21.40	1	40x1.10 water	112	520x520	630-700	40 MHz 10 line accumu.
**S25A**	H2B-HT7 and Tomm20-HT9	MaP555-CA	SP8-FALCON	550	17.59	1	40x1.10 water	100	448x448	570-620	80 MHz 10 line accumu.
**S25B**	H2B-HT7 and Tomm20-HT10	MaP555-CA	SP8-FALCON	550	2.39	1	40x1.10 water	101	680x680	570-600	80 MHz 6 line accumu.
**S25C**	H2B-HT7 and Tomm20-HT11	MaP555-CA	SP8-FALCON	550	3.83	1	40x1.10 water	101	424x424	570-600	80 MHz 8 line accumu.
**S25D**	H2B-HT9 and Tomm20-HT10	MaP555-CA	SP8-FALCON	550	2.09	1	40x1.10 water	101	776x776	570-600	80 MHz 10 line accumu.
**S25E**	H2B-HT9 and Tomm20-HT11	MaP555-CA	SP8-FALCON	550	3.33	1	40x1.10 water	101	488x488	570-600	80 MHz 6 line accumu.
**S25F**	H2B-HT10 and Tomm20-HT11	MaP555-CA	SP8-FALCON	550	3.13	1	40x1.10 water	1010	520x520	570-600	80 MHz 8 line accumu.

**S26A**	Tomm20-HT7 and H2B-HT9	MaP555-CA	SP8-FALCON	550	21.89	1	40x1.10 water	101	360x360	570-620	80 MHz 10 line accumu.
**S26B**	Tomm20-HT7 and H2B-HT10	MaP555-CA	SP8-FALCON	550	3.75	1	40x1.10 water	100	432x432	570-600	80 MHz 8 line accumu.
**S26C**	Tomm20-HT7 and H2B-HT11	MaP555-CA	SP8-FALCON	550	3.44	1	40x1.10 water	100	472x472	570-600	80 MHz 10 line accumu.
**S26D**	Tomm20-HT9 and H2B-HT10	MaP555-CA	SP8-FALCON	550	2.90	1	40x1.10 water	101	560x560	570-600	80 MHz 10 line accumu.
**S26E**	Tomm20-HT9 and H2B-HT11	MaP555-CA	SP8-FALCON	550	2.70	1	40x1.10 water	101	600x600	570-600	80 MHz 4 line accumu.
**S26F**	Tomm20-HT10 and H2B-HT11	MaP555-CA	SP8-FALCON	550	3.50	1	40x1.10 water	101	464x464	570-600	80 MHz 6 line accumu.
**S27A**	CEP41-HT7 and H2B-HT9	MaP555-CA	SP8-FALCON	550	7.94	1	40x1.10 water	101	496x496	570-620	80 MHz 10 line accumu.
**S27B**	CEP41-HT7 and H2B-HT10	MaP555-CA	SP8-FALCON	550	4.78	1	40x1.10 water	101	824x824	570-600	80 MHz 4 line accumu.
**S27C**	CEP41-HT7 and H2B-HT11	MaP555-CA	SP8-FALCON	550	8.49	1	40x1.10 water	100	464x464	570-600	80 MHz 6 line accumu.
**S27D**	CEP41-HT9 and H2B-HT10	MaP555-CA	SP8-FALCON	550	8.64	1	40x1.10 water	101	456x456	570-600	80 MHz 4 line accumu.
**S27E**	CEP41-HT9 and H2B-HT11	MaP555-CA	SP8-FALCON	550	6.31	1	40x1.10 water	101	624x624	570-600	80 MHz 8 line accumu.
**S27F**	CEP41-HT11 and H2B-HT10	MaP555-CA	SP8-FALCON	550	7.81	1	40x1.10 water	101	504x504	570-600	80 MHz 10 line accumu.
**S28A**	CEP41-HT7 and Tomm20-HT9	MaP555-CA	SP8-FALCON	550	7.35	1	40x1.10 water	101	536x536	570-600	80 MHz 10 line accumu.
**S28B**	CEP41-HT7 and Tomm20-HT10	MaP555-CA	SP8-FALCON	550	6.65	1	40x1.10 water	102	608x608	570-600	80 MHz 8 line accumu.
**S28C**	CEP41-HT7 and Tomm20-HT11	MaP555-CA	SP8-FALCON	550	6.93	1	40x1.10 water	101	568x568	570-600	80 MHz 8 line accumu.
**S28D**	CEP41-HT9 and Tomm20-HT10	MaP555-CA	SP8-FALCON	550	5.59	1	40x1.10 water	101	704x704	570-600	80 MHz 10 line accumu.
**S28E**	CEP41-HT9 and Tomm20-HT11	MaP555-CA	SP8-FALCON	550	5.93	1	40x1.10 water	101	664x664	570-600	80 MHz 10 line accumu.
**S28F**	CEP41-HT11 and Tomm20-HT10	MaP555-CA	SP8-FALCON	550	4.39	1	40x1.10 water	102	896x896	570-600	80 MHz 12 line accumu.

**S29A**	Tomm20-HT79 and H2B-HT7	MaP618-CA	SP8-FALCON	615	8.64	1	40x1.10 water	112	456x456	630-700	80 MHz<br>10 line accumu.
**S29B**	Tomm20-HT7 and H2B-HT10	MaP618-CA	SP8-FALCON	615	4.23	1	40x1.10 water	113	384x384	635-700	80 MHz<br>10 line accumu.<br>PFA fixed
**S29C**	Tomm20-HT7 and H2B-HT11	MaP618-CA	SP8-FALCON	615	3.03	1	40x1.10 water	113	536x536	635-700	80 MHz<br>10 line accumu.<br>PFA fixed
**S29D**	Tomm20-HT9 and H2B-HT10	MaP618-CA	SP8-FALCON	615	4.41	1	40x1.10 water	113	368x368	635-700	80 MHz<br>10 line accumu.<br>PFA fixed
**S29E**	Tomm20-HT9 and H2B-HT11	MaP618-CA	SP8-FALCON	615	4.51	1	40x1.10 water	113	360x360	635-700	80 MHz<br>10 line accumu.<br>PFA fixed
**S29F**	Tomm20-HT11 and H2B-HT10	MaP618-CA	SP8-FALCON	615	3.83	1	40x1.10 water	113	424x424	635-700	80 MHz<br>10 line accumu.<br>PFA fixed
**S30A**	Tomm20-HT79 and H2B-HT7	MaP555-CA	SP8-FALCON	550	4.55	1	40x1.10 water	101	864x864	570-620	80 MHz<br>12 line accumu.<br>PFA fixed
**S30B**	Tomm20-HT7 and H2B-HT10	MaP555-CA	SP8-FALCON	550	2.66	1	40x1.10 water	100	608x608	570-600	80 MHz<br>8 line accumu.<br>PFA fixed
**S30C**	Tomm20-HT7 and H2B-HT11	MaP555-CA	SP8-FALCON	550	3.44	1	40x1.10 water	101	472x472	570-600	80 MHz<br>9 line accumu.<br>PFA fixed
**S30D**	Tomm20-HT9 and H2B-HT10	MaP555-CA	SP8-FALCON	550	3.03	1	40x1.10 water	101	536x536	570-600	80 MHz<br>10 line accumu.<br>PFA fixed
**S30E**	Tomm20-HT9 and H2B-HT11	MaP555-CA	SP8-FALCON	550	3.44	1	40x1.10 water	101	472x472	570-600	80 MHz<br>10 line accumu.<br>PFA fixed
**S30F**	Tomm20-HT11 and H2B-HT10	MaP555-CA	SP8-FALCON	550	2.56	1	40x1.10 water	101	384x384	570-600	80 MHz<br>10 line accumu.<br>PFA fixed
**S31A**	H2B-HT7, Tomm20-HT9, β4Gal-T1-HT10	MaP618-CA	SP8-FALCON	615	6.48	1	40x1.10 water	90	608x608	635-700	80 MHz<br>10 line accumu.

**S31B**	H2B-HT7,<br>Tomm20-HT9,<br>Lyn11-HT10	MaP618-CA	SP8-FALCON	615	5.93	1	40x1.10 water	112	664x664	635-700	80 MHz<br>10 line accumu.
**S31C**	H2B-HT7,<br>Tomm20-HT9,<br>LAMP1-HT10	MaP618-CA	SP8-FALCON	615	3.56	1	40x1.10 water	112	456x456	635-700	80 MHz<br>10 line accumu.
**S31D**	H2B-HT7,<br>Tomm20-HT9,<br>Lifeact-HT10	MaP618-CA	SP8-FALCON	615	1.99	1	40x1.10 water	113	816x816	635-700	80 MHz<br>10 line accumu.
**S31E**	H2B-HT7,<br>Tomm20-HT9,<br>β4Gal-T1-HT11	MaP618-CA	SP8-FALCON	615	6.08	1	40x1.10 water	112	648x648	635-700	80 MHz<br>10 line accumu.
**S31F**	H2B-HT7,<br>Tomm20-HT9,<br>Lyn11-HT11	MaP618-CA	SP8-FALCON	615	6.08	1	40x1.10 water	112	648x648	635-700	80 MHz<br>15 line accumu.
**S31G**	H2B-HT7,<br>Tomm20-HT9,<br>LAMP1-HT11	MaP618-CA	SP8-FALCON	615	3.33	1	40x1.10 water	113	488x488	635-700	80 MHz<br>9 line accumu.
**S31H**	H2B-HT7,<br>Tomm20-HT9,<br>Lifeact-HT11	MaP618-CA	SP8-FALCON	615	8.06	1	40x1.10 water	113	488x488	635-700	80 MHz<br>8 line accumu.
**S31I**	Tomm20-HT7,<br>H2B-HT10,<br>Lifeact-HT11	MaP618-CA	SP8-FALCON	615	6.31	1	40x1.10 water	113	624x624	635-700	80 MHz<br>10 line accumu.
**S31J**	Tomm20-HT9,<br>H2B-HT10,<br>Lyn11-HT11	MaP618-CA	SP8-FALCON	615	2.15	1	40x1.10 water	113	752x752	635-700	80 MHz<br>10 line accumu.
**S31K**	Tomm20-HT9,<br>H2B-HT10,<br>Lifeact-HT11	MaP618-CA	SP8-FALCON	615	7.14	1	40x1.10 water	113	552x552	635-700	80 MHz<br>10 line accumu.
**S32A**	H2B-HT7,<br>Tomm20-HT9,<br>Lyn11-HT10	MaP555-CA	SP8-FALCON	550	2.03	1	40x1.10 water	101	800x800	570-600	80 MHz<br>10 line accumu.
**S32B**	H2B-HT7,<br>Tomm20-HT9,<br>Lifeact-HT10	MaP555-CA	SP8-FALCON	550	2.44	1	40x1.10 water	101	664x664	570-600	80 MHz<br>10 line accumu.
**S32C**	H2B-HT7,<br>Tomm20-HT9,	MaP555-CA	SP8-FALCON	550	1.69	1	40x1.10 water	101	960x960	570-600	80 MHz<br>8 line accumu.

	Lyn11-HT11										
**S32D**	H2B-HT7,<br>Tomm20-HT9,<br>Lifeact-HT11	MaP555-CA	SP8-FALCON	550	2.81	1	40x1.10 water	100	576x576	570-600	80 MHz<br>10 line accumu.
**S32E**	Tomm20-HT7,<br>H2B-HT10,<br>Lifeact-HT11	MaP555-CA	SP8-FALCON	550	6.08	1	40x1.10 water	101	648x648	570-600	80 MHz<br>12 line accumu.
**S32F**	Tomm20-HT9,<br>H2B-HT10,<br>Lyn11-HT11	MaP555-CA	SP8-FALCON	550	2.56	1	40x1.10 water	101	632x632	570-600	80 MHz<br>10 line accumu.
**S32G**	Tomm20-HT9,<br>H2B-HT10,<br>Lifeact-HT11	MaP555-CA	SP8-FALCON	550	2.33	1	40x1.10 water	101	696x696	570-600	80 MHz<br>10 line accumu.
**S33A**	H2B-HT7 and<br>Tomm20-HT9	MaP555-CA<br>MaP555-Actin	SP8-FALCON	550	10.94	1	40x1.10 water	101	720x720	570-620	80 MHz<br>10 line accumu.
**S33B**	H2B-HT7 and<br>Tomm20-HT9	MaP555-CA<br>MaP555-Actin	SP8-FALCON	550	13.68	1	40x1.10 water	101	576x576	570-620	80 MHz<br>10 line accumu.
**S33C**	H2B-HT7 and<br>Tomm20-HT9	MaP618-CA<br>MaP618-Actin	SP8-FALCON	615	15.15	1	40x1.10 water	112	520x520	630-700	80 MHz<br>10 line accumu.
**S33D**	H2B-HT7 and<br>Tomm20-HT9	MaP555-CA<br>MaP555-<br>Tubulin	SP8-FALCON	550	6.39	1	40x1.10 water	101	616x616	570-620	80 MHz<br>10 line accumu.
**S33E**	Tomm20-HT9	MaP555-CA<br>MaP555-<br>Tubulin	SP8-FALCON	550	8.64	1	40x1.10 water	100	456x456	570-620	80 MHz<br>10 line accumu.
**S33F**	CEP41-HT7 and<br>Tomm20-HT9	MaP555-CA<br>MaP555-DNA	SP8-FALCON	550	6.31	1	40x1.10 water	102	624x624	570-620	80 MHz<br>10 line accumu.
**S34A**	HTx	MaP618-CA	SP8	*	1.58	1	40x1.10 water	284	1024x1024	*	Spectra
**S34B**	HTx	MaP555-CA	SP8	*	1.58	1	40x1.10 water	284	1024x1024	*	Spectra
**S34C**	-	MaP618-Actin<br>or<br>MaP618-DNA	SP8	*	1.58	1	40x1.10 water	284	1024x1024	*	Spectra
**S34D**	-	MaP555-Actin	SP8	*	3.84	1	40x1.10 water	284	1024x1024	*	Spectra
**S34D**	-	MaP555-<br>Tubulin	SP8	*	3.84	1	40x1.10 water	284	1024x1024	*	Spectra<br>2 line accumu.
**S34D**	-	MaP555-DNA	SP8	*	1.58	1	40x1.10 water	284	1024x1024	*	Spectra
**S34D**	SNAP-tag	MaP555-BG	SP8	*	1.58	1	40x1.10 water	284	1024x1024	*	Spectra

**S35**	H2B-HT7 and Tomm20-HT9	MaP618-CA	SELLARIS 8 STED FALCON	615	1.20	1	86x1.20 water	20	1792x1792	630-760	80 MHz<br>10 line accumu.<br>2 pixel bin (confocal)<br>STED
**S36A**	β4Gal-T1-HT7, Tomm20-HT9 LAMP1-HT11	MaP618-CA	SP8-FALCON	615	5.86	1	40x1.10 water	101	672x672	635-700	80 MHz<br>12 line accumu.
**S36A**	SNAP-tag	MaP555-BG MaP555-Actin MaP555-DN	SP8-FALCON	550	5.86	1	40x1.10 water	101	672x672	570-600	80 MHz<br>12 line accumu.
**S36B**	β4Gal-T1-HT7, Tomm20-HT9 LAMP1-HT11	MaP618-CA	SP8-FALCON	615	6.84	1	40x1.10 water	101	576x576	635-700	80 MHz<br>10 line accumu.
**S36B**	SNAP-tag	MaP555-BG MaP555-Actin MaP555-DN	SP8-FALCON	550	6.84	1	40x1.10 water	101	576x576	570-600	80 MHz<br>10 line accumu.
**S37A**	*LT*-Fucci(CA)	MaP618-CA	SP8-FALCON	615	3.88	1	40x1.10 water	284	1024x1024	640-700	40 MHz<br>4 line accumu.<br>2 pixel binning
**S37B**	*LT*-Fucci(CA)	MaP618-CA	SP8-FALCON	615	1.58	5	40x1.10 water	284	1024x1024	640-700	40 MHz<br>4 line accumu.<br>2 pixel binning<br>24 h every 12 min
**S37C**	*LT*-Fucci(SA)	MaP618-CA	SP8-FALCON	615	1.58	5	40x1.10 water	284	1024x1024	640-700	40 MHz<br>4 line accumu.<br>2 pixel binning<br>24 h every 12 min
**S37D**	*LT*-Fucci(SCA)	MaP618-CA	SP8-FALCON	615	1.58	5	40x1.10 water	284	1024x1024	640-700	40 MHz<br>4 line accumu.<br>2 pixel binning<br>24 h every 12 min
**S38A**	*LT*-Fucci(CA)	MaP618-CA	SP8-FALCON	615	1.58	5	40x1.10 water	284	1024x1024	640-700	40 MHz<br>4 line accumu.<br>2 pixel binning<br>24 h every 12 min
**S38B**	*LT*-Fucci(CA)	MaP618-CA	SELLARIS 8 (FALCON)	615	1.58	1	86x1.20 water	284	1024x1024	630-700	80 MHz<br>10 line accumu.

											TauContrast
**S38C**	*LT*-Fucci(CA)	MaP555-CA	SP8-FALCON	550	7.69	5	40x1.10 water	569	512x512	570-620	40 MHz<br>10 line accumu.

Supplementary Videos

Supplementary Videos 1-3. *LT*-Fucci biosensors over 24 h in living U-2 OS cells.

FastFlim video of U-2 OS cells stably expressing *LT*-Fucci(CA) (**1**), *LT*-Fucci(SA) (**2**), or *LT*-Fucci(SCA) (**3**) after no-wash labeling with MaP618-CA (1 µM). For all biosensors HaloTag9-hCdt is present during G1 and the nuclei will therefore present long average photon arrival times (MaP618-CA: 3.7 ns, orange). During S, G2 and M phase HaloTag7-hGem or a mixture of HaloTag7-hGem and HaloTag9-hCdt is present, resulting in short average photon arrival times (MaP618-CA: 3.1 ns, green) or a gradient of medium average photon arrival times (MaP618-CA: ~3.4 ns, light-green). Scale bar, 50 µm.

Supplementary Methods

Protein sequences

>His-TEV-Halo-EGFP

MHHHHHHHHHHENLYFQGIGTGFPFDPHYVEVLGERMHYVDVGPRDGTPVLFLHGNPTSSYVWRNIIPHVAPTHRCI
APDLIGMGKSDKPDLGYFFDDHVRFMDAFIEALGLEEVVLVIHDWGSALGFHWAKRNPERVKGIAFMEFIRPIPTWDE
WPEFARETFQAFRTTDVGRKLIIDQNVFIEGTLPMGVVRPLTEVEMDHYREPFLNPVDREPLWRFPNELPIAGEPANIV
ALVEEYMDWLHQSPVPKLLFWGTPGVLIPPAEAARLAKSLPNCKAVDIGPGLNLLQEDNPDLIGSEIARWLSTLEIVSKG
EELFTGVVPILVELDGDVNGHKFSVSGEGEGDATYGKLTLKFICTTGKLPVPWPTLVTTLTYGVQCFSRYPDHMKQHDF
FKSAMPEGYVQERTIFFKDDGNYKTRAEVKFEGDTLVNRIELKGIDFKEDGNILGHKLEYNYNSHNVYIMADKQKNGIK
VNFKIRHNIEDGSVQLADHYQQNTPIGDGPVLLPDNHYLSTQSALSKDPNEKRDHMVLLEFVTAAGITLGMDELYK

Red: His-tag, Purple: TEV-cleavage site, Blue: HaloTag7, Black: Sites of mutations, Green: EGFP.

>His-TEV-Halo

MHHHHHHHHHHENLYFQGIGTGFPFDPHYVEVLGERMHYVDVGPRDGTPVLFLHGNPTSSYVWRNIIPHVAPTHRCI
APDLIGMGKSDKPDLGYFFDDHVRFMDAFIEALGLEEVVLVIHDWGSALGFHWAKRNPERVKGIAFMEFIRPIPTWDE
WPEFARETFQAFRTTDVGRKLIIDQNVFIEGTLPMGVVRPLTEVEMDHYREPFLNPVDREPLWRFPNELPIAGEPANIV
ALVEEYMDWLHQSPVPKLLFWGTPGVLIPPAEAARLAKSLPNCKAVDIGPGLNLLQEDNPDLIGSEIARWLSTLEI

Red: His-tag, Purple: TEV-cleavage site, Blue: HaloTag7, Black: Sites of mutations

Synthesis

General considerations All chemical reagents and anhydrous solvents for synthesis were purchased from commercial suppliers (Acros, Merck, Sigma-Aldrich, and TCI) and used without further purification. CA-NHBoc was synthesized according to literature procedures[15]. Alexa647-NHS was purchased from ThermoFisher. Composition of mixed solvents is given by volume ratio (v/v). High-resolution mass spectrometry (HRMS) was performed by the MS-facility of the Max Planck Institute for Medical Research on a Bruker maXis II™ ETD. Liquid chromatography coupled to mass spectrometry (LC-MS) was performed on a Shimadzu MS2020 connected to a Nexera UHPLC system equipped with a Supelco Titan C18 80 Å (1.9 µm, 2.1 x 50 mm). Buffer A: 0.05% HCOOH in H_2O Buffer B: 0.05% HCOOH in ACN. Analytical gradient was from 10% to 90% B within 6 min with 0.5 mL min^{-1} flow. Preparative reverse phase high-performance liquid chromatography (RP-HPLC) was either carried out on a Dionex system equipped with an UltiMate 3000 diode array detector for product visualization on a Supleco Ascentis® C18 column (5 µm, 10 x 250 mm) or on a Supleco Ascentis® C18 column (5 µm, 21.2 x 250 mm). Buffer A: 0.1% TFA in H_2O Buffer

B: ACN. Typical gradient was from 10% to 90% B within 32 min with 4 or 8 mL min^{-1} flow or on a Shimadzu system equipped with an SPD-M20A diode array detector for product visualization and a LCMS-2020 for mass detection on a Shimadzu Shim-pack GIS C18 column (5 µm, 50 x 250 mm). Buffer A: 0.1% FA in H_2O Buffer B: 0.1% FA in ACN. Typical gradient was from 10% to 90% B within 60 min with 50 mL min^{-1} flow.

Cyanine3-CA

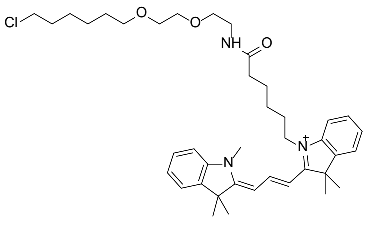

Cyanine3-CA

Cyanine3-COOH was synthesized according to Ueno et al. 2011.[39]
A solution of Cyanine3-COOH (50 mg, 219 µmol, 1.0 equiv.) in dry DMSO (1.0 mL) was treated with DIPEA (229 µL, 1.3 mmol, 6.0 equiv.) and TSTU (92.1 mg, 306 µmol, 1.4 equiv.). The mixture was stirred for 10 min at room temperature. In a separate vial a solution of CA-NHBoc (78.7 mg, 241 µmol, 1.1 eq.) in TFA (500 µL) was shaken for 20 min. The solution was evaporated and dried. The residue was taken up in DMSO (500 µL) and added to the other mixture together with DIPEA (76 µL, 438 mmol, 2.0 equiv.). The mixture was shaken for 30 min and then acidified with TFA (150 µL). RP-HPLC (50 mL min^{-1}, 10% to 90% B in 60 min) gave Cyanine3-CA (62 mg, 43%) as a red solid.
HRMS (ESI): calc. for $C_{40}H_{57}ClN_3O_3^+$ $[M]^+$: 662.4083; found 661.4084.

Cyanine5-CA

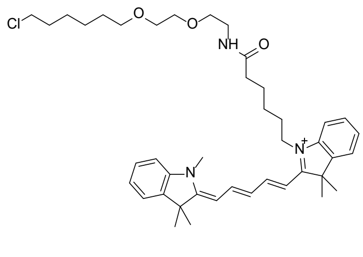

Cyanine5-CA

Cyanine5-COOH was synthesized according to Ueno et al. 2010.[39]
A solution of Cyanine5-COOH (100 mg, 207 µmol, 1.0 equiv.) in dry DMSO (2.0 mL) was treated with DIPEA (205 µL, 1.24 mmol, 6.0 equiv.) and TSTU (87.1 mg, 289 µmol, 1.4 equiv.). The mixture was stirred for 10 min at room temperature. In a separate vial a solution of CA-NHBoc (73.5 mg, 227 µmol, 1.1 eq.) in TFA (500 µL) was shaken for 20 min. The solution was evaporated and dried. The residue was taken up in DMSO (500 µL) and added to the other mixture together with DIPEA (68 µL, 414 mmol, 2.0 equiv.). The mixture was shaken for 30 min and then acidified with TFA (150 µL). RP-HPLC (50 mL min^{-1}, 10% to 90% B in 60 min) gave Cyanine5-CA (98 mg, 69%) as a blue solid.
HRMS (ESI): calc. for $C_{42}H_{59}ClN_3O_3^+$ $[M]^+$: 688.4239; found 688.4239.

Alexa647-CA

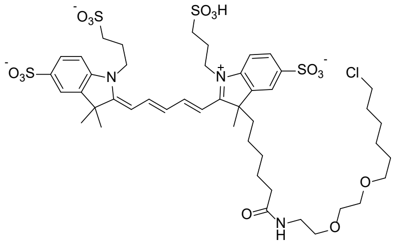

A solution of Alex647-NHS (0.3 mg, 0.3 µmol, 1.0 equiv.) in dry DMSO (150 µL) was treated with DIPEA (0.12 µL, 0.9 µmole, 6.0 equiv.). The mixture was stirred for 10 min at room temperature. In a separate vial a solution of CA-NHBoc (0.38 mg, 1.1 µmol, 3.6 eq.) in TFA (50 µL) was shaken for 20 min. The solution was evaporated and dried. The residue was taken up in DMSO (50 µL) and added to the other mixture together with DIPEA (68 µL, 414 mmol, 2.0 equiv.). The mixture was shaken for 30 min and then acidified with TFA (150 µL). RP-HPLC (8 mL min^{-1}, 5% to 60% B in 32 min) gave Alexa647-CA as a blue solid, which was taken up in 40 µL DMSO (1.98 mM, 25%).
HRMS (ESI): calc. for $C_{46}H_{64}ClN_3O_{15}S_4^{2-}$ $[M]^+$: 530.6460; found 530.6449.

References

1. Berro, A. J. & Schreiter, E. R. Crystal structure of HaloTag bound to tetramethylrhodamine-HaloTag ligand. (2019). doi:10.2210/pdb6u32/pdb
2. Savarese, M. *et al.* Fluorescence lifetimes and quantum yields of rhodamine derivatives: New insights from theory and experiment. *J. Phys. Chem. A* **116**, 7491–7497 (2012).
3. Savarese, M. *et al.* Non-radiative decay paths in rhodamines: New theoretical insights. *Phys. Chem. Chem. Phys.* **16**, 20681–20688 (2014).
4. Grimm, J. B. *et al.* A general method to improve fluorophores for live-cell and single-molecule microscopy. *Nat. Methods* **12**, 244–250 (2015).
5. Marmé, N., Knemeyer, J. P., Sauer, M. & Wolfrum, J. Inter- and Intramolecular Fluorescence Quenching of Organic Dyes by Tryptophan. *Bioconjug. Chem.* **14**, 1133–1139 (2003).
6. Casey, J. R., Grinstein, S. & Orlowski, J. Sensors and regulators of intracellular pH. *Nat. Rev. Mol. Cell Biol.* **11**, 50–61 (2010).
7. Baker, N. A., Sept, D., Joseph, S., Holst, M. J. & McCammon, J. A. Electrostatics of nanosystems: Application to microtubules and the ribosome. *Proc. Natl. Acad. Sci. U. S. A.* **98**, 10037–10041 (2001).
8. Jurrus, E. *et al.* Improvements to the APBS biomolecular solvation software suite. *Protein*

Sci. **27**, 112–128 (2018).

9. Uno, S. *et al.* A spontaneously blinking fluorophore based on intramolecular spirocyclization for live-cell super-resolution imaging. *Nat. Chem.* **6**, 681–689 (2014).
10. Belov, V. N., Bossi, M. L., Fölling, J., Boyarskiy, V. P. & Hell, S. W. Rhodamine Spiroamides for Multicolor Single-Molecule Switching Fluorescent Nanoscopy. *Chem. - A Eur. J.* **15**, 10762–10776 (2009).
11. Eggeling, C., Widengren, J., Rigler, R. & Seidel, C. A. M. Photobleaching of Fluorescent Dyes under Conditions Used for Single-Molecule Detection: Evidence of Two-Step Photolysis. *Anal. Chem.* **70**, 2651–2659 (1998).
12. Roberti, M. J. *et al.* TauSense: a fluorescence lifetime-based tool set for everyday imaging. *Nat. Methods* (2020).
13. Lambert, T. J. FPbase: a community-editable fluorescent protein database. *Nat. Methods* **16**, 277–278 (2019).
14. Shirmanova, M. V *et al.* FUCCI - Red : a single - color cell cycle indicator for fluorescence lifetime imaging. *Cell. Mol. Life Sci.* **78**, 3467–3476 (2021).
15. Los, G. V *et al.* HaloTag: A Novel Protein Labeling Technology for Cell Imaging and Protein Analysis. *ACS Chem. Biol.* **3**, 373–382 (2008).
16. Grimm, J. B. *et al.* A general method to fine-tune fluorophores for live-cell and in vivo imaging. *Nat. Methods* **14**, 987–994 (2017).
17. Grimm, J. B. *et al.* A General Method to Improve Fluorophores Using Deuterated Auxochromes. *JACS AU* (2020). doi:10.1101/2020.08.17.250027
18. Hoffman, D. P. *et al.* Correlative three-dimensional super-resolution and block-face electron microscopy of whole vitreously frozen cells. *Science* **367**, eaaz5357 (2020).
19. Grimm, J. B., Brown, T. A., Tkachuk, A. N. & Lavis, L. D. General Synthetic Method for Si-Fluoresceins and Si-Rhodamines. *ACS Cent. Sci.* **3**, 975–985 (2017).
20. Grimm, J. B. *et al.* A general method to optimize and functionalize red-shifted rhodamine dyes. *Nat. Methods* **17**, 815–821 (2020).
21. Wang, L. *et al.* A general strategy to develop cell permeable and fluorogenic probes for multicolour nanoscopy. *Nat. Chem.* **12**, 165–172 (2020).

22. Grimm, J. B., Gruber, T. D., Ortiz, G., Brown, T. A. & Lavis, L. D. Virginia Orange: A Versatile, Red-Shifted Fluorescein Scaffold for Single- And Dual-Input Fluorogenic Probes. *Bioconjug. Chem.* **27**, 474–480 (2016).

23. Butkevich, A. N. *et al.* Fluorescent Rhodamines and Fluorogenic Carbopyronines for Super-Resolution STED Microscopy in Living Cells. *Angew. Chem. Int. Ed.* **55**, 3290–3294 (2016).

24. Deo, C. *et al.* The HaloTag as a general scaffold for far-red tunable chemigenetic indicators. *Nat. Chem. Biol.* **17**, 718–723 (2021).

25. Jonker, C. T. H. *et al.* Accurate measurement of fast endocytic recycling kinetics in real time. *J. Cell Sci.* **133**, jcs231225 (2020).

26. Lukinavičius, G. *et al.* A near-infrared fluorophore for live-cell super-resolution microscopy of cellular proteins. *Nat. Chem.* **5**, 132–139 (2013).

27. Zheng, Q. *et al.* Rational Design of Fluorogenic and Spontaneously Blinking Labels for Super-Resolution Imaging. *ACS Cent. Sci.* **5**, 1602–1613 (2019).

28. Liu, Z. *et al.* Systematic comparison of 2A peptides for cloning multi-genes in a polycistronic vector. *Sci. Rep.* **7**, 2193 (2017).

29. Frei, M. S. *et al.* Photoactivation of silicon rhodamines via a light-induced protonation. *Nat. Commun.* **10**, 4580 (2019).

30. Wilhelm, J. *et al.* Kinetic and Structural Characterization of the Self-Labeling Protein Tags HaloTag7, SNAP-tag and CLIP-tag. *Biochemistry* (2021). doi:10.1021/acs.biochem.1c00258

31. Moeyaert, B. *et al.* Improved methods for marking active neuron populations. *Nat. Commun.* **9**, 4440 (2018).

32. Sallin, O. *et al.* Semisynthetic biosensors for mapping cellular concentrations of nicotinamide adenine dinucleotides. *Elife* **7**, e32638 (2018).

33. Goedhart, J. *et al.* Structure-guided evolution of cyan fluorescent proteins towards a quantum yield of 93%. *Nat. Commun.* **3**, 751 (2012).

34. Falcón-Pérez, J. M., Nazarian, R., Sabatti, C. & Dell'Angelica, E. C. Distribution and dynamics of Lamp1-containing endocytic organelles in fibroblasts deficient in BLOC-3. *J.*

Cell Sci. **118**, 5243–5255 (2005).

35. Brun, M. A. *et al.* A semisynthetic fluorescent sensor protein for glutamate. *J. Am. Chem. Soc.* **134**, 7676–7678 (2012).

36. Bajar, B. T. *et al.* Fluorescent indicators for simultaneous reporting of all four cell cycle phases. *Nat. Methods* **13**, 993–996 (2016).

37. Shcherbakova, D. M. *et al.* Bright monomeric near-infrared fluorescent proteins as tags and biosensors for multiscale imaging. *Nat. Commun.* **7**, 12405 (2016).

38. Lam, A. J. *et al.* Improving FRET dynamic range with bright green and red fluorescent proteins. *Nat. Methods* **9**, 1005–1012 (2012).

39. Ueno, Y. *et al.* Encapsulated energy-transfer cassettes with extremely well resolved fluorescent outputs. *J. Am. Chem. Soc.* **133**, 51–55 (2011).
