# Supplementary material for: Engineered HaloTag variants for fluorescence lifetime multiplexing
Source: Nat Methods. 2021 Dec 16;19(1):65–70. doi: 10.1038/s41592-021-01341-x (PMC8748199; doi:10.1038/s41592-021-01341-x)
Supplement: Supplementary file 2 — Reporting Summary [file 41592_2021_1341_MOESM2_ESM.pdf]

nature research

Corresponding author(s): Kai Johnsson

Last updated by author(s): Oct 19, 2021

Reporting Summary

Nature Research wishes to improve the reproducibility of the work that we publish. This form provides structure for consistency and transparency in reporting. For further information on Nature Research policies, see our Editorial Policies and the Editorial Policy Checklist.

Statistics

For all statistical analyses, confirm that the following items are present in the figure legend, table legend, main text, or Methods section.

n/a	Confirmed	
☐	☒	The exact sample size (*n*) for each experimental group/condition, given as a discrete number and unit of measurement
☐	☒	A statement on whether measurements were taken from distinct samples or whether the same sample was measured repeatedly
☐	☒	The statistical test(s) used AND whether they are one- or two-sided<br>*Only common tests should be described solely by name; describe more complex techniques in the Methods section.*
☒	☐	A description of all covariates tested
☐	☒	A description of any assumptions or corrections, such as tests of normality and adjustment for multiple comparisons
☐	☒	A full description of the statistical parameters including central tendency (e.g. means) or other basic estimates (e.g. regression coefficient) AND variation (e.g. standard deviation) or associated estimates of uncertainty (e.g. confidence intervals)
☐	☒	For null hypothesis testing, the test statistic (e.g. *F*, *t*, *r*) with confidence intervals, effect sizes, degrees of freedom and *P* value noted<br>*Give P values as exact values whenever suitable.*
☒	☐	For Bayesian analysis, information on the choice of priors and Markov chain Monte Carlo settings
☒	☐	For hierarchical and complex designs, identification of the appropriate level for tests and full reporting of outcomes
☒	☐	Estimates of effect sizes (e.g. Cohen's *d*, Pearson's *r*), indicating how they were calculated

Our web collection on statistics for biologists contains articles on many of the points above.

Software and code

Policy information about availability of computer code

Data collection	NMR: Bruker TopSpin 3.5 and Bruker TopSpin 3.6<br>HRMS: Bruker otofControl 4.1, Bruker Hystar 4.1 SR2 software<br>Plate reader: Tecan Sparkcontrol Method Editor V.2.2<br>Quantaurus: Hamamatsu Quantaurus - QY Plus 4.2.0<br>Structure optimization and analysis: Gaussian 16 and Schroedinger Maestro 12.3.013<br>Microscopy: Leica LAS X 3.5.7.23225(Confocal) and LASX FLIM/FCS 3.5.6, Imspector 16.3.13030 (STED), LAS X 4.2.0 (FLIM-STED)<br>Stopped Flow: Bio-Kine32 4.80<br>Thermostability: R.ThermControl v.2.12
Data analysis	General data analysis: Origin Pro 2018b b9.5.5.409, R version 3.6.0, Microsoft Excel 2016 (16.0.5122.1000)<br>Kinetic analysis: Dynafit 4<br>Image anlaysis: Image J 1.53c, SymPhoTime 64 V 2.6 , LLeica LAS X 3.5.7.23225(Confocal) and LASX FLIM/FCS 3.5.6, LAS X 4.2.0 (FLIM-phasor analysis)<br>Analysis of modeled structures: Avogadro 1.2.0<br>Chemical synthesis: Bruker DataAnalysis 4.4 SR1.<br>X-ray crystallography: XDS (VERSION Mar 15, 2019 BUILT=20190315 and VERSION Jan 31, 2020 BUILT=20200131), Refmac5 (versions 5.8.0258), Phaser (version 2.8.2 and 2.8.3), Coot (version 0.8.9.2), PHENIX (versions 1.15.2-3472 and 1.17.1-3660) and the MolProbity implemented therein (version 4.4), PyMOL version 2.1.1 and APBS electrostatics plugin (3.0.0), PDB2PQR web service (3.1.0)<br>Thermostability: PR.ThermControl v2.3.1

For manuscripts utilizing custom algorithms or software that are central to the research but not yet described in published literature, software must be made available to editors and reviewers. We strongly encourage code deposition in a community repository (e.g. GitHub). See the Nature Research guidelines for submitting code & software for further information.

Data

Policy information about availability of data

All manuscripts must include a data availability statement. This statement should provide the following information, where applicable:

- Accession codes, unique identifiers, or web links for publicly available datasets
- A list of figures that have associated raw data
- A description of any restrictions on data availability

Plasmids encoding HaloTag variants and fusions thereof have been deposited on Addgene. Accession codes can be found in Supplementary Table S18. The X-ray crystal structures of HaloTag9-TMR, HaloTag10-TMR, and HaloTag11-TMR have been deposited to the PDB with deposition codes 6ZVY, 7PCX, and 7PCW. HaloTag7-TMR is available on the PDB with deposition code 6Y7A. Correspondence and requests for materials should be addressed to K.J.. The data supporting the findings of this study are available within the paper and its Supplementary Information and are available from the corresponding author upon reasonable request.

Field-specific reporting

Please select the one below that is the best fit for your research. If you are not sure, read the appropriate sections before making your selection.

☒ Life sciences ☐ Behavioural & social sciences ☐ Ecological, evolutionary & environmental sciences

For a reference copy of the document with all sections, see nature.com/documents/nr-reporting-summary-flat.pdf

Life sciences study design

All studies must disclose on these points even when the disclosure is negative.

Sample size	Sample size was based on experience in prior studies or samples were acquired until a clear trend was evident.
Data exclusions	No data was excluded
Replication	In vitro measurements were performed in triplicates or as indicated. Microscopy experiments were performed on a minimum of two different sample preparations and different field of views. All replicates were successful.
Randomization	Randomization was not necessary for the presented experiments, as the data is not relevant to a clinical trial.
Blinding	Blinding was not necessary for the presented experiments, as the data is not relevant to a clinical trial.

Reporting for specific materials, systems and methods

We require information from authors about some types of materials, experimental systems and methods used in many studies. Here, indicate whether each material, system or method listed is relevant to your study. If you are not sure if a list item applies to your research, read the appropriate section before selecting a response.

Materials & experimental systems

n/a	Involved in the study
☒	☐ Antibodies
☐	☒ Eukaryotic cell lines
☒	☐ Palaeontology and archaeology
☒	☐ Animals and other organisms
☒	☐ Human research participants
☒	☐ Clinical data
☒	☐ Dual use research of concern

Methods

n/a	Involved in the study
☒	☐ ChIP-seq
☒	☐ Flow cytometry
☒	☐ MRI-based neuroimaging

Eukaryotic cell lines

Policy information about cell lines

Cell line source(s)	U-2 OS provided by ATCC HTB-96.<br>U-2 OS FlpIn TREx cell line was generated based on reported work (Molecular and Cellular Biology 2006, 26 (12), 4642-4651) and described in the method in supporting information.
Authentication	Cell lines were not further authenticated.
Mycoplasma contamination	Cell lines have been tested and are negative.

Commonly misidentified lines<br>(See ICLAC register)	Not applicable as no commonly misidentified cell lines were used.
